# Synthesis of 3-(Quinazolin-4-yl)propionic Acids via an Acid-Catalyzed Rearrangement of 4-Oxobutyronitriles

**DOI:** 10.3390/ijms27093903

**Published:** 2026-04-28

**Authors:** Nicolai A. Aksenov, Alexander E. Kurlikov, Alexander P. Barbolin, Polina S. Karaseva, Milena M. Baziyants, Elizabeth A. Glotova, Igor A. Kurenkov, Dmitrii A. Aksenov, Alexander V. Aksenov

**Affiliations:** Department of Organic Chemistry, North Caucasus Federal University, 1a Pushkin St., 355017 Stavropol, Russia; vip.kurlikov@mail.ru (A.E.K.); aleksander.barbolin@gmail.com (A.P.B.); karasevapolina0000@gmail.com (P.S.K.); baziyants.milena@yandex.ru (M.M.B.); lisabethglotova@yandex.ru (E.A.G.); kurenkman@icloud.com (I.A.K.); daksenov@ncfu.ru (D.A.A.); aaksenov@ncfu.ru (A.V.A.)

**Keywords:** cascade transformations, recyclization, formylation, quinazolines, pyrrolidine-2-ones, 3-cyanoketones

## Abstract

4-(2-Aminophenyl)-4-oxobutyronitriles in the presence of formic acid (HCOOH) and *p*-toluenesulfonic acid (TsOH) undergo an unusual rearrangement providing access to a range of 3-(quinazolin-4-yl)propionic acids that have been poorly represented in the literature, with only a few isolated examples known to date. The reaction demonstrates a new route to the quinazoline core through transfer of the nitrile group nitrogen, mediated through the formation of a pyrrolidine cycle. The availability of 4-oxobutyronitrile precursors—2′-aminochacones—provides high variability of substituents in the required positions of product. The transformation is general and can be extended to the preparation of 2-substituted 3-(quinazolin-4-yl)propionic acids through preliminary acylation or to the synthesis of 4-(quinazolin-4-yl)butyric acids.

## 1. Introduction

The assembly of the quinazoline core represents an important task in the total synthesis of various compounds with practically significant properties [1,2,3,4,5]. Serving as the target structure in the above-mentioned transformations, quinazolines are also important precursors in the synthesis of other heterocyclic systems, providing access to indoles [6,7,8], benzodiazepines [8,9,10,11,12], isoxazoles [13], as well as a wide range of fused systems [14,15,16].

**Scheme 1:**
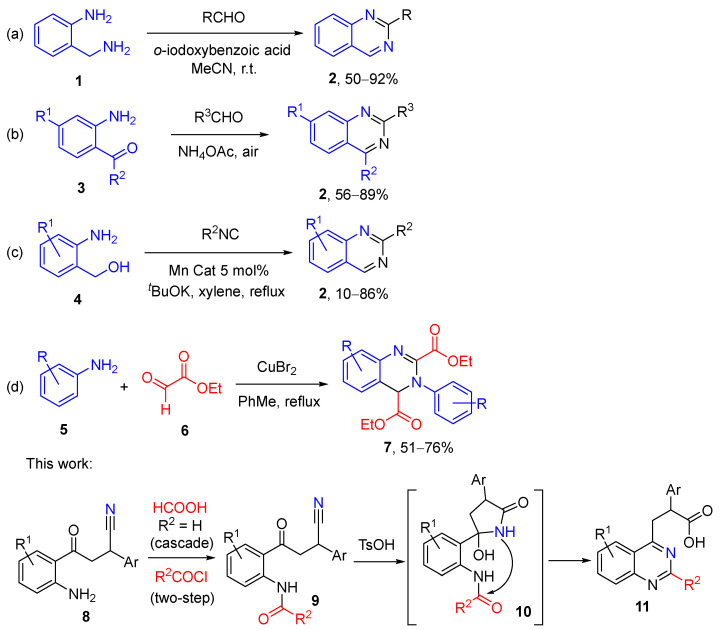
General routes to quinazoline core. (**a**) Cyclization of 2-(aminomethyl)anilines with aldehydes [19]; (**b**) approach based on aminoacetophenones [22]; (**c**) oxidative *in situ* generation of formylanilines [24]; (**d**) cyclization of anilines via C–C bond formation with ethyl glyoxylate [27].

Given the interest in this scaffold, investigating new synthetic methodologies for the quinazoline core remains an important task, which has been reflected in reviews of recent years [2,3,17,18]. Intuitively most obvious methods involve the cyclization of 2-(aminomethyl)anilines **1** with aldehydes [19,20] or carboxylic acid derivatives [21] in the presence of oxidizing agents (Scheme 1a). Similar approaches involve the transformation of azomethines obtained from 2-formyl- and 2-acylanilines **3** [22,23] (Scheme 1b). Additionally, the literature describes numerous transformations that rely on the *in situ* generation of these key starting materials through metal-catalyzed reactions [24,25,26] (Scheme 1c). Finally, methods based on cyclization through C-C bond formation are more uncommon but were presented as well [27] (Scheme 1d). Despite the variety of approaches presented, there are no reports in the literature on the use of accessible and easily functionalized 2′-aminochalcones and their Michael addition adducts as building blocks for the quinazoline core. At the same time, the closest methods, based on 2′-aminoacetophenones **3** [22,23], rely on the use of external nitrogen sources, completely ignoring the internal potential of the substrate for intramolecular cyclization without excess reagents. In this work, we wish to present a method for the preparation of 3-(quinazolin-4-yl)propionic acids **11** through effective cascade transformation of *N*-formyl(acyl)-4-(2-aminophenyl)-4-oxobutyronitriles **9**. Mechanistically, this process begins with the formation of a 5-hydroxypyrrolidin-2-one **10**, which undergoes an unusual intramolecular capture of the lactam nitrogen by the amide or formamide carbonyl. Acylation of 4-oxobutyronitriles **8** requires an additional synthetic step, while formylation with formic acid can be integrated into a general cascade process. 

## 2. Results and Discussion

**Scheme 2:**
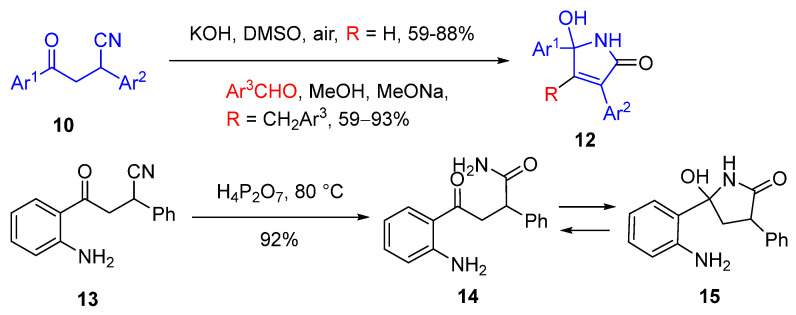
Cyclization of 3-cyanoketones to pyrrolines and pyrrolidines.

We have previously found that 3-cyanoketones **10** [28] in an alkaline medium in the presence of air can be involved to oxidative cyclization yielding corresponding 5-hydroxypyrrolin-2-ones **12** [29]. Similarly, 4-benzyl-substituted products (**12**, R = CH_2_Ar) are formed when the oxidizing agent was replaced with benzaldehydes [30]. Meanwhile, the nitrile group also undergoes hydrolysis in the presence of pyrophosphoric acid (80% P_2_O_5_ polyphosphoric acid), yielding the corresponding butyramides **14** [31]. This work is based on the assumption that the latter exist in equilibrium with 5-hydroxypyrrolidin-2-ones **15**, which can be intercepted by interaction with an internal electrophile (Scheme 2).

Anilines in the presence of hot formic acid or formate buffer undergo a formylation reaction [32]. The resulting formamide functional group is suitable for intercepting the 5-hydroxypyrrolidin-2-one presumably formed in the reaction medium, similar to methods shown previously [22]. Since pyrophosphoric acid is incompatible with formic acid (HCOOH) [33], we began the optimization of conditions with oxalic acid (H_2_C_2_O_4_), which has a comparable pKa. Indeed, boiling of cyanoketone **8a** in formic acid led to the desired reaction product **11aa** with a yield of 19% (Table 1, row 1). Replacing oxalic acid with methanesulfonic acid (MsOH) only slightly reduced the yield of the product, likely due to strong protonation of the amino group, which diverted a significant portion of **8a** from the main reaction pathway (Table 1, row 2), while a catalytic amount of magnesium perchlorate (Mg(ClO_4_)_2_), which has a high affinity for the carbonyl group [34], gave a yield of 65% (Table 1, row 3). Next, we decided to test microwave activation (MW). There are reports in the literature about the increased efficiency of some organic reactions due to more uniform heating of the solution [35,36]. Initially, we repeated the experiment shown in row 1 at an elevated temperature, which allowed for an increase in yield to 73% (Table 1, row 4). Further variation of conditions with H_2_C_2_O_4_ did not lead to any improvement (Table 1, rows 5–7); therefore, we decided to change the catalyst to *p*-toluenesulfonic acid (TsOH), which allowed us to obtain the best result (Table 1, row 8). Reducing the amount of formic acid significantly suppressed the formation of the target quinazoline (Table 1, row 9). Next, we tested a series of acids such as CCl_3_COOH, CF_3_COOH, BF_3_·Et_2_O, as well as the use of pure HCOOH, which did not allow for an increase in yield (Table 1, rows 10–13). We assume that the optimality of TsOH results from a combination of moderate acidity and chemical inertness toward the components of the mixture.

**Scheme 3:**
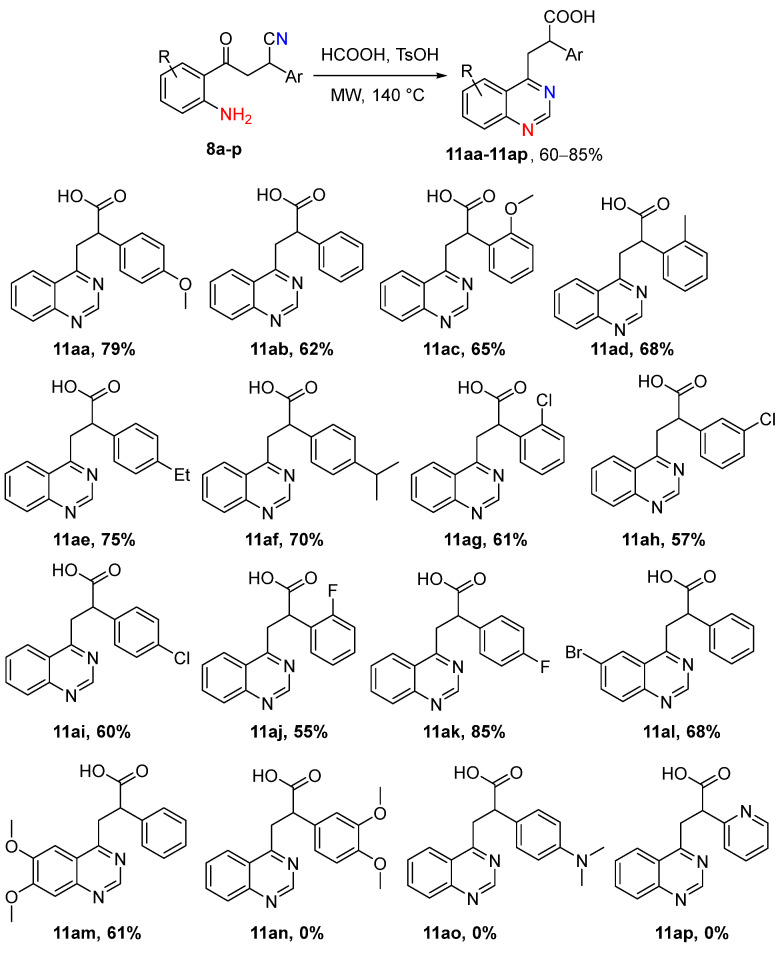
Scope of 3-(quinazolin-4-yl)propionic acids **10** with free position 2.

Next, we have prepared a small library of 3-(quinazolin-4-yl)propionic acids **11** with free second position. Compared to the parent compound **11ab**, the introduction of electron-donating substituents at the para-position of the aryl moiety results in higher yields of products **11aa**, **11ae**, and **11af**. The presence of halogens at the meta- and para-positions has virtually no effect on the reaction outcome, similar to the introduction of substituents at the ortho-position. It is worth noting that the ortho-methoxy derivative **11ac** reacted with a significantly lower yield than its para-substituted analogue **11aa**. Introduction of bromine at the 6-position of the quinazoline (**11al**) gives a slightly higher yield, and the presence of two methoxy groups (**11am**) does not have a significant effect. Compounds **11an**, **11ao**, and **11ap** could not be obtained by the general procedure. In the case of **11an**, we assume high reactivity of the dimethoxy-substituted aryl moiety in Friedel–Crafts-type side reactions, whereas the presence of basic functional groups in **11ao** and **11ap** likely blocks the main reaction pathway (Scheme 3).

**Figure 1:**
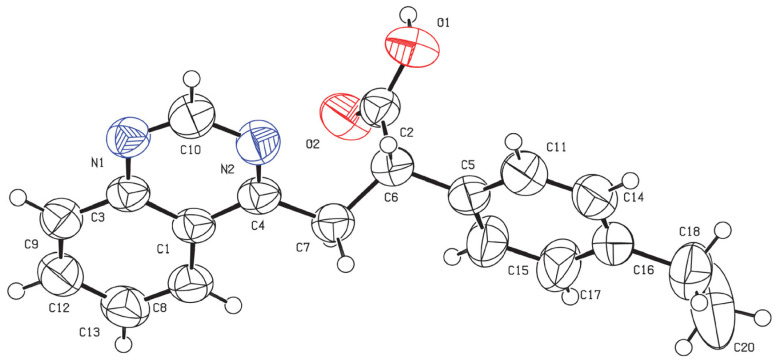
ORTEP plot of **11ae** (CCDC # 2536851) showing atom numbering scheme and 50% probability thermal ellipsoids.

Compared to previously described methods based on the cyclocondensation of aminoacetophenones [23] or the contraction of an indole ring [37,38], this method gives a higher yield of quinazolylpropionic acids. It is also worth noting that the presented studies [23,37,38] provide only a few examples of such compounds, while the use of 3-cyanoketones allows for a high variability of substituents. The NMR spectra of the compounds contain characteristic signals of the CH-CH_2_ diastereotopic system, which appear as three doublets of doublets in the area of 3.5–4.5 ppm (see Supplementary Materials, Figures S40–S65). Also, the proton in the second position appears as expected at around 9 ppm, and the carboxyl proton is detected as a singlet with a shift of 12 ppm, which, depending on the concentration, the presence of moisture, or the electronic structure, varies from practically merging with the baseline to a sharp, elongated peak. Combined with high-resolution mass spectra, these observations left no doubt about the obtained structure; however, for greater certainty, the structure of compound **10ae** was ambiguously confirmed by single-crystal X-ray crystallography (Figure 1). The crystal structure of **10ae** reveals a flattened molecular geometry. The orientation of the quinazoline and aryl rings is characterized by a slight twist, with an interplanar angle of 14.70(7)° and a centroid-to-centroid distance of 6.54 Å. The structure is stabilized by an intermolecular hydrogen bond between the carboxyl group and the N1 atom of the quinazoline ring. The proton is clearly localized on the oxygen atom of the carboxylic moiety, which confirms the absence of a zwitterionic form. The ethyl group is disordered due to thermal motion at room temperature; for clarity, only the major disorder component is shown in the figure; however, the residual thermal motion is still reflected by the enlarged ellipsoids of C20 in this fragment. The bond lengths and valence angles are within their typical ranges and do not require further discussion.

**Scheme 4:**
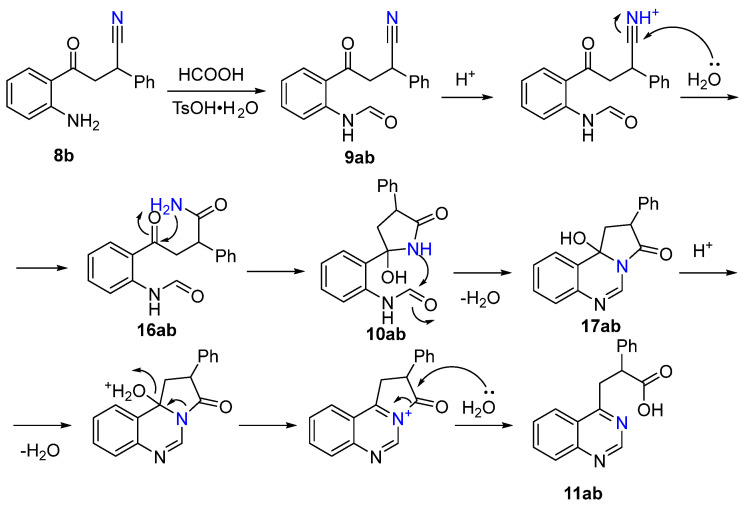
Proposed mechanism of reaction.

The proposed reaction mechanism presented below is based on an example of compound **8b**. First, formylation of the substrate occurs with the formation of formamide **9ab**, which is further hydrolyzed to give amide **16ab**. As stated earlier, the **16ab**–**10ab** equilibrium is thermodynamically unfavorable, remaining shifted toward the starting material **16ab**. Nevertheless, in the presence of an electrophilic formamide group at a short distance, cyclization occurs with the formation of dihydroquinazoline **17ab**, which undergoes ring cleavage upon dehydration to give the target 2-phenyl-3-(quinazolin-4-yl)propionic acid **11ab** (Scheme 4).

**Scheme 5:**
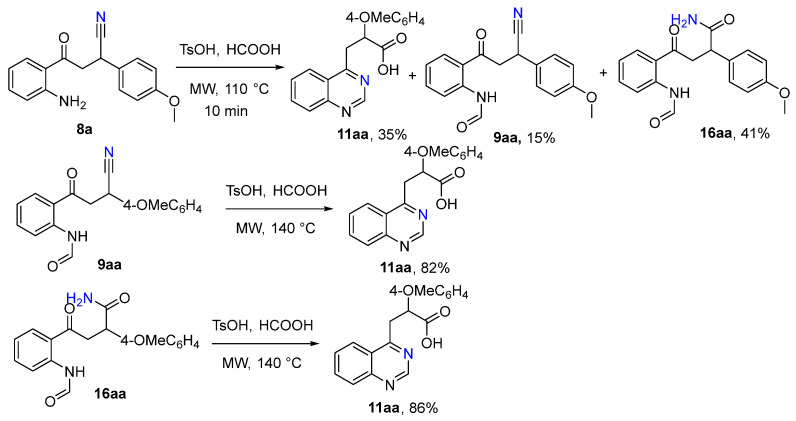
Control experiments.

To prove the reaction mechanism, the transformation was performed at a lower temperature for a period of 10 min, which allowed the isolation of intermediates **9aa** and **16aa** (for NMR spectra, see Supplementary Materials, Figures S83–S86). The detection of compound **9aa** in the reaction mixture unequivocally indicates that formylation is the first step of the process, which occurs due to the difficulty of protonating the nitrile group in the presence of a free amine, and, consequently, the absence of activation for hydrolysis. These were further subjected to standard conditions to give quinazoline **11aa** in yields of 82% and 86%, respectively (Scheme 5).

**Scheme 6:**
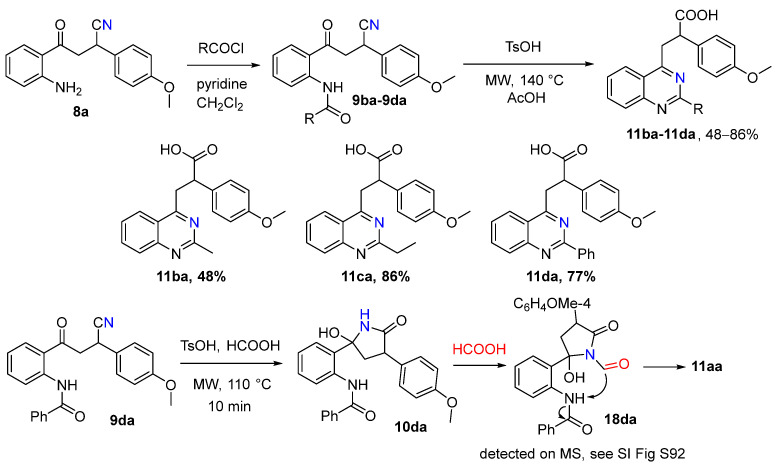
Scope of 3-(quinazolin-4-yl)propionic acids **10** with substituents in position 2.

**Figure 2:**
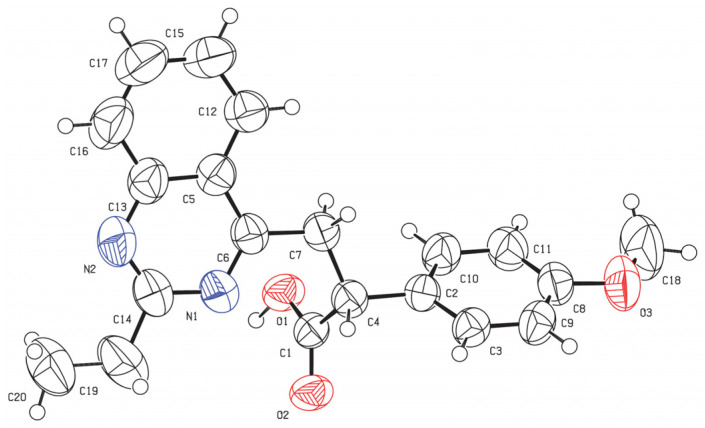
ORTEP plot of **11ca** (CCDC # 2536851) showing atom numbering scheme and 50% probability thermal ellipsoids. The carbon atoms are shown in black, oxygen in red, and nitrogen in blue. Hydrogen atoms are represented by black empty circles.

Having achieved good results with quinazolines with a free second position, we decided to expand the range of quinazolines by preliminary *N*-acylation of cyanoketones **13** (for NMR spectra of **9**, see Supplementary Materials, Figures S22–S27). Reaction of **9ba**–**9da** under standard conditions led to the formation of trace amounts of 2-unsubstituted quinazoline **11aa** probably due to the re-acylation process (for NMR spectra of mixtures, see Supplementary Materials, Figures S86–S91). To avoid this, formic acid was replaced with AcOH. Notably, the 2-methyl derivative **11ba** has a lower output, while the 2-ethyl and 2-phenylquinazolines **11ca** and **11da**, respectively, were obtained in high yield (for NMR spectra, see Supplementary Materials, Figures S66–S71). Reaction of **9da** at a lower temperature enabled the detection of side-intermediate **18da**, responsible for the formation of **11aa** through the alternative cyclization pathway with debenzoylation (for NMR spectra, see Supplementary Materials, Figure S92). This result indirectly confirms the presence of pyrrolidin-2-one **10** in the reaction mixture (Scheme 6). The structure of compound **11ca** was unambiguously confirmed by X-ray diffraction analysis (Figure 2).

**Scheme 7:**
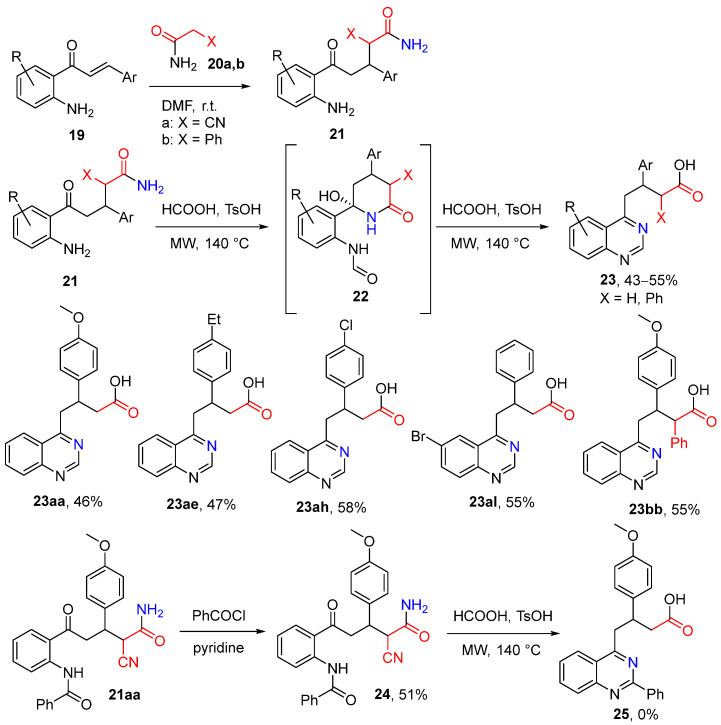
Synthesis of 4-(quinazolin-4-yl)butyric acids **23**.

**Figure 3:**
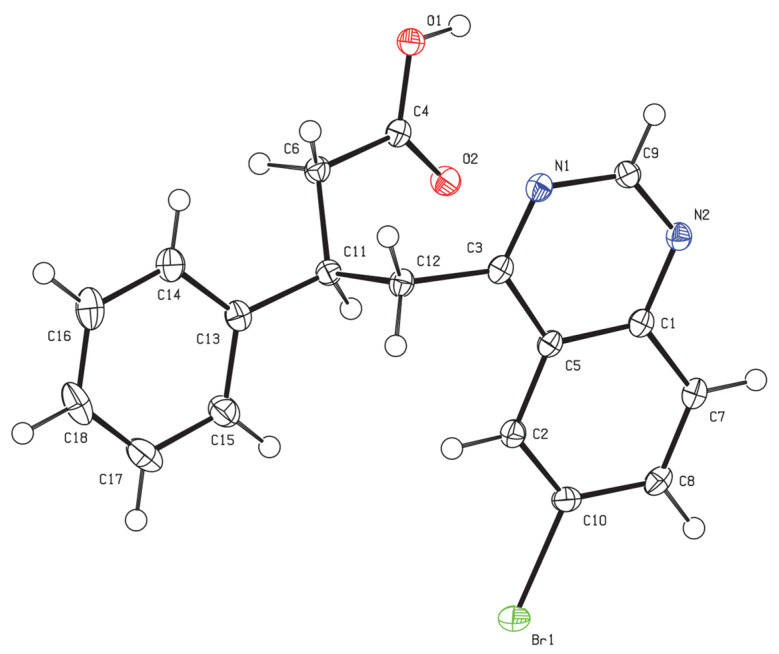
ORTEP plot of **23al** (CCDC # 2536852) showing atom numbering scheme and 50% probability thermal ellipsoids. The carbon atoms are shown in black, oxygen in red, bromine in green, and nitrogen in blue. Hydrogen atoms are represented by black empty circles.

Finally, we tested the possibility of obtaining 4-(quinazolin-4-yl)butyric acids **23**, which should involve the intermediate formation of 6-hydroxypiperidin-2-ones **22**. For this purpose, we synthesized adducts of 2′-aminochalcones **19** with cyanoacetamide **20a** and phenylacetamide **20b**. The best results were obtained without the use of a base at room temperature, while addition in the presence of K_2_CO_3_, DBU, BuOK gave a yield of no more than 20%. It is worth noting that the adducts were formed as a mixture of diastereomers, which is clearly evident from the NMR spectra (see Supplementary Materials, Figures S28–S39). Under standard conditions, the target compounds **23** were formed in moderate yields (for NMR spectra, see Supplementary Materials, Figures S72–S81). It is noteworthy that products **23aa**, **23ae**, **23ah**, and **23al** are formed through reductive decyanation under the action of formic acid. In this case, the influence of the substituents’ electronic effects was not observed due to the remoteness of the aryl fragment. An attempt to obtain the 2-phenyl derivative **25** from the benzoylated adduct **24** was unsuccessful leading only to the degradation of the starting compound, probably due to the low electrophilicity of (Scheme 7). The structure of compound **23al** was unambiguously confirmed by X-ray diffraction analysis (Figure 3).

## 3. Methods and Materials

### 3.1. General Information

NMR spectra, ^1^H, ^13^C, ^19^F, were measured in solutions of CDCl_3_ on Bruker AVANCE-III HD instrument (Bruker BioSpin, Karlsruhe, Germany) at 400, 101, 376 MHz, respectively. Residual solvent signals were used as internal standards (7.26 ppm for ^1^H, and 77.16 ppm for ^13^C nuclei). HRMS spectra were measured on Bruker maXis impact (Bruker Daltonik, Bremen, Germany, electrospray ionization, in MeCN solutions, employing HCO_2_Na–HCO_2_H for calibration). IR spectra were measured on FT-IR spectrometer Shimadzu IRAffinity-1S (Shimadzu Corp., Kyoto, Japan) equipped with an ATR sampling module. Reaction progress, purity of isolated compounds, and R_f_ values were monitored with TLC on ALUGRAM RP-18 W/UV254 plates (Macherey-Nagel GmbH & Co. KG, Düren, Germany). X-ray analysis [39,40,41] was performed on a Rigaku Oxford Diffraction SuperNova dual-source diffractometer (Cu microfocus X-ray source) equipped with an AtlasS2 CCD detector (Wrocław, Poland). Column chromatography was performed on silica gel (32–63 μm, 60 Å pore size, Macherey-Nagel GmbH & Co. KG, Düren, Germany). Melting points were measured with Stuart SMP30 apparatus (Antylia Scientific, Stone, UK). MW-assisted reactions were conducted in G10 vials using an Anton Paar Monowave 300 reactor (Anton Paar GmbH, Austria, Graz, serial number 81552252) employing constant temperature mode with temperature control by IR sensor. Maximum power on the way to the target temperature is 150 watts, while maintaining the temperature is 10 watts. 2-aryl-4-(2-aminophenyl)-4-oxobutyronitriles **8a**–**p** and chalcones **19a**,**e**,**h**,**l** were synthetized by previously described procedure [31]. All other reagents and solvents were purchased from Merck (Darmstadt, Germany) and used as received.

### 3.2. Preparation of 2-(4-Methoxyphenyl)-4-(2-(acylamino)phenyl)-4-oxobutanenitriles ***9*** (General Procedure)

A 10 mL round-bottomed flask was charged with 4-(2-aminophenyl)-2-(4-methoxyphenyl)-4-oxobutanenitrile **8a** (560 mg, 2.00 mmol), CH_2_Cl_2_ (5 mL) and Et_3_N (305 µL, 222 mg, 2.2 mmol). Then, an acylating agent (AcCl, EtCOCl or BzCl, 2.2 mmol) was slowly added and the reaction mixture was stirred at room temperature for 2 h. After consumption of starting material (TLC control), the reaction mixture was diluted with water (40 mL) and extracted with CH_2_Cl_2_ (4 × 40 mL). Combined organic fractions were concentrated *in vacuo* and obtained residue was purified by recrystallization from ethanol.

*N*-(2-(3-cyano-3-(4-methoxyphenyl)propanoyl)phenyl)acetamide (**9ba**): white solid, m.p. 133.3–134.9 °C (EtOH), *R*_f_ 0.33 (EtOAc/hexane, 1:2, *v*/*v*). Yield 489 mg (1.52 mmol, 76%). ^1^H NMR (400 MHz, CDCl_3_) δ 11.46 (s, 1H), 8.75 (d, *J* = 8.5 Hz, 1H), 7.78 (dd, *J* = 8.0, 1.5 Hz, 1H), 7.56 (ddd, *J* = 8.6, 7.2, 1.5 Hz, 1H), 7.33 (d, *J* = 8.7 Hz, 2H), 7.08 (ddd, *J* = 8.3, 7.3, 1.2 Hz, 1H), 6.92 (d, *J* = 8.7 Hz, 2H), 4.42 (dd, *J* = 8.6, 5.4 Hz, 1H), 3.81 (s, 3H), 3.75 (dd, *J* = 17.9, 8.6 Hz, 1H), 3.51 (dd, *J* = 17.9, 5.5 Hz, 1H), 2.25 (s, 3H); ^13^C NMR (101 MHz, CDCl_3_) δ 199.0, 169.7, 159.7, 141.4, 135.9, 130.4, 128.7 (2C), 126.8, 122.5, 121.1, 120.8, 120.5, 114.8 (2C), 55.5, 45.8, 31.4, 25.7; FTIR, v_max_: 3302, 2246, 1753, 1558, 1519, 1252, 1199, 1156 cm^−1^; HRMS (ESI TOF) *m*/*z* calc’d. for C_19_H_18_N_2_NaO_3_ [M + Na]^+^: 345.1210, found: 345.1211 (−0.3 ppm).

*N*-(2-(3-cyano-3-(4-methoxyphenyl)propanoyl)phenyl)propionamide (**9ca**): white solid, m.p. 147.9–150.1 °C (EtOH), *R*_f_ 0.44 (EtOAc/hexane, 1:2, *v*/*v*). Yield 551 mg (1.64 mmol, 82%). ^1^H NMR (400 MHz, CDCl_3_) δ 11.48 (s, 1H), 8.78 (dd, *J* = 8.6, 1.2 Hz, 1H), 7.77 (dd, *J* = 8.1, 1.6 Hz, 1H), 7.56 (ddd, *J* = 8.7, 7.2, 1.5 Hz, 1H), 7.33 (d, *J* = 8.7 Hz, 2H), 7.07 (ddd, *J* = 8.3, 7.3, 1.2 Hz, 1H), 6.91 (d, *J* = 8.7 Hz, 2H), 4.43 (dd, *J* = 8.6, 5.5 Hz, 1H), 3.80 (s, 3H), 3.75 (dd, *J* = 17.9, 8.6 Hz, 1H), 3.51 (dd, *J* = 17.9, 5.5 Hz, 1H), 2.49 (q, *J* = 7.6 Hz, 2H), 1.28 (t, *J* = 7.6 Hz, 3H); ^13^C NMR (101 MHz, CDCl_3_) δ 198.9, 173.5, 159.7, 141.5, 135.9, 130.4, 128.7 (2C), 126.8, 122.4, 121.1, 120.8, 120.6, 114.8 (2C), 55.5, 45.8, 31.8, 31.4, 9.7; FTIR, v_max_: 3292, 2244, 1753, 1583, 1517, 1254, 1187, 1162 cm^−1^; HRMS (ESI TOF) *m*/*z* calc’d. for C_20_H_20_N_2_NaO_3_ [M + Na]^+^: 359.1366, found: 359.1361 (1.5 ppm).

*N*-(2-(3-cyano-3-(4-methoxyphenyl)propanoyl)phenyl)benzamide (**9da**): white solid, m.p. 140.7–142.2 °C (EtOH), *R*_f_ 0.48 (EtOAc/hexane, 1:2, *v*/*v*). Yield 676 mg (1.76 mmol, 88%). ^1^H NMR (400 MHz, CDCl_3_) δ 12.43 (s, 1H), 8.99 (dd, *J* = 8.6, 1.2 Hz, 1H), 8.11–8.02 (m, 2H), 7.84 (dd, *J* = 8.2, 1.6 Hz, 1H), 7.64 (ddd, *J* = 8.7, 7.2, 1.5 Hz, 1H), 7.61–7.52 (m, 3H), 7.39–7.32 (m, 2H), 7.13 (ddd, *J* = 8.2, 7.2, 1.2 Hz, 1H), 6.98–6.82 (m, 2H), 4.49 (dd, *J* = 8.7, 5.4 Hz, 1H), 3.83 (t, *J* = 9.1, 8.5 Hz, 1H), 3.80 (s, 3H), 3.54 (dd, *J* = 17.9, 5.5 Hz, 1H); ^13^C NMR (101 MHz, CDCl_3_) δ 199.4, 166.3, 159.8, 141.8, 136.2, 134.7, 132.3, 130.6, 129.0 (2C), 128.7 (2C), 127.6 (2C), 126.8, 122.8, 121.3, 121.0, 120.9, 114.8 (2C), 55.5, 46.0, 31.5; FTIR, v_max_: 3282, 2238, 1753, 1675, 1560, 1299, 1248, 1197 cm^−1^; HRMS (ESI TOF) *m*/*z* calc’d. for C_24_H_20_N_2_NaO_3_ [M + Na]^+^: 407.1366, found: 407.1358 (2.0 ppm).

### 3.3. Preparation of Michael Adducts ***21*** and ***24*** (General Procedure)

A 25 mL round-bottomed flask was charged with 2′-aminochalcone **19** (5.00 mmol), cyanoacetamide or 2-phenylacetamide **20** (1.2 equiv., 6 mmol), DMF (10 mL) and the reaction mixture was stirred at room temperature for 5 h. After consumption of starting material (TLC control), the reaction mixture was diluted with water (100 mL) and extracted with CH_2_Cl_2_ (4 × 25 mL). Combined organic fractions were washed with water (4 × 50 mL), concentrated *in vacuo* and obtained residue was purified by column chromatography (EtOAc/hexane, 1:1, *v*/*v*).

5-(2-Aminophenyl)-2-cyano-3-(4-methoxyphenyl)-5-oxopentanamide (mixture of diastereomers A + B 1.05:1) (**21aa**): yellow solid, m.p. 157.1–159.6 °C (EtOH), *R*_f_ 0.2 (EtOAc/hexane, 1:1, *v*/*v*). Yield 1.460 g (4.3 mmol, 86%). ^1^H NMR (400 MHz, DMSO-*d*_6_) δ 7.88 (s, 1H, A), 7.83 (s, 1H, B), 7.81 (d, *J* = 8.4 Hz, 1H, B), 7.76 (d, *J* = 7.7 Hz, 1H, A), 7.62 (s, 1H, A), 7.42 (s, 1H, B), 7.33–7.17 (m, 6H, A + B), 7.13 (s, 4H, A + B NH_2_), 6.85 (dd, *J* = 11.0, 8.7 Hz, 4H, A + B), 6.72 (d, *J* = 8.4 Hz, 2H, B), 6.62–6.51 (m, 2H, A + B), 4.08 (d, *J* = 8.8 Hz, 1H, A), 4.02–3.83 (m, 3H, A + B), 3.76 (t, *J* = 10.3, 7.4 Hz, 1H, B), 3.71 (s, 3H, A), 3.70 (s, 3H, B), 3.61 (dd, *J* = 16.7, 10.1 Hz, 1H, A), 3.32 (dd, *J* = 17.0, 3.1 Hz, 1H, B), 3.18 (dd, *J* = 16.7, 4.1 Hz, 1H, A); ^13^C NMR (101 MHz, DMSO-*d*_6_) δ 198.62 (B), 198.57 (A), 165.93 (A), 165.86 (B), 158.3 (A), 158.2 (B), 151.2 (2C, A + B), 134.3 (2C, A + B), 132.0 (B), 131.8 (A), 131.1 (B), 131.0 (A), 129.2 (2C, A), 129.1 (2C, B), 118.0 (B), 117.8 (A), 117.08 (B), 117.06 (A), 116.43 (B), 116.35 (A), 114.49 (B), 114.47 (A), 113.7 (2C, A), 113.6 (2C, B), 55.0 (A + B), 44.41 (B), 44.39 (A), 42.0 (A), 40.7 (B), 40.3 (A), 40.2 (B); FTIR, v_max_: 3358, 3181, 2242, 1702, 1517, 1252, 1162 cm^−1^; HRMS (ESI TOF) *m*/*z* calc’d. for C_19_H_19_N_3_NaO_3_ [M + Na]^+^: 360.1319, found: 360.1321 (−0.6 ppm).

5-(2-Aminophenyl)-2-cyano-3-(4-ethylphenyl)-5-oxopentanamide (mixture of diastereomers A + B 1.2:1) (**21ae**): yellow oil, *R*_f_ 0.34 (EtOAc/hexane, 1:1, *v*/*v*). Yield 1.190 g (3.55 mmol, 71%). ^1^H NMR (400 MHz, DMSO-*d*_6_) δ 7.94 (s, 1H, A), 7.90 (s, 1H, B), 7.83 (dd, *J* = 8.3, 1.5 Hz, 1H, B), 7.79 (dd, *J* = 8.3, 1.5 Hz, 1H, A), 7.66 (s, 1H, A), 7.48 (s, 1H, B), 7.37–7.28 (m, 4H, A + B), 7.27–7.19 (m, 2H, A + B), 7.14 (dd, *J* = 9.7, 8.0 Hz, 4H, A + B), 6.76 (d, *J* = 8.4 Hz, 1H, A), 6.62–6.47 (m, 2H, A + B), 4.15 (d, *J* = 8.8 Hz, 1H, A), 4.10–3.99 (m, 2H, A + B), 3.95 (td, *J* = 9.3, 4.0 Hz, 1H, A), 3.80 (dd, *J* = 17.2, 9.6 Hz, 1H, B), 3.67 (dd, *J* = 16.9, 9.9 Hz, 1H, A), 3.39 (dd, *J* = 17.1, 3.0 Hz, 1H, B), 3.24 (dd, *J* = 17.0, 4.1 Hz, 1H, A), 2.69–2.43 (m, 4H, A + B), 1.21–1.10 (m, 6H, A + B); ^13^C NMR (101 MHz, DMSO-*d*_6_) δ 198.94 (B), 198.91 (A), 166.40 (A), 166.36 (B), 151.60 (B), 151.58 (A), 143.0 (A), 142.9 (B), 138.0 (B), 137.6 (A), 134.8 (2C, A + B), 131.44 (B), 131.41 (A), 128.5 (2C, B), 128.3 (2C, B), 128.2 (2C, A), 128.1 (2C, B), 118.4 (B), 118.3 (A), 117.6 (A), 116.9 (B), 116.8 (A), 115.0 (ZA), 44.8 (B), 44.7 (A), 42.4 (A), 41.1 (A), 41.0 (B), 40.8 (B), 28.2 (2C), 15.83 (B), 15.76 (A); FTIR, v_max_: 3337, 2966, 2253, 1699, 1656, 1617, 1377, 1241 cm^−1^; HRMS (ESI TOF) *m*/*z* calc’d. for C_20_H_21_N_3_NaO_2_ [M + Na]^+^: 358.1526, found: 358.1525 (0.3 ppm).

5-(2-Aminophenyl)-3-(4-chlorophenyl)-2-cyano-5-oxopentanamide (**21ah**): yellow solid, m.p. 161.3–162.1 °C (IPA), *R*_f_ 0.19 (EtOAc/hexane, 1:1, *v*/*v*). Yield 1.418 g (4.15 mmol, 83%). ^1^H NMR (400 MHz, DMSO-*d*_6_) δ 7.85 (s, 1H), 7.79 (d, *J* = 8.2 Hz, 1H), 7.44 (s, 1H), 7.40–7.31 (m, 4H), 7.23 (t, *J* = 7.7 Hz, 1H), 7.12 (s, 2H), 6.71 (d, *J* = 8.4 Hz, 1H), 6.54 (t, *J* = 7.6 Hz, 1H), 4.08–3.90 (m, 2H), 3.78 (dd, *J* = 17.2, 10.1 Hz, 1H), 3.35–3.31 (m, 1H); ^13^C NMR (101 MHz, DMSO-*d*_6_) δ 198.3, 165.5, 151.2, 139.2, 134.4, 131.7, 131.1, 130.0 (2C), 128.2 (2C), 117.7, 117.1, 116.3, 114.5, 43.9, 40.5, 40.2; FTIR, v_max_: 3359, 2938, 2253, 1702, 1637, 1329, 1227 cm^−1^; HRMS (ESI TOF) *m*/*z* calc’d. for C_18_H_16_ClN_3_NaO_2_ [M + Na]^+^: 364.0823, found: 364.0837 (−3.7 ppm).

5-(2-Amino-5-bromophenyl)-2-cyano-5-oxo-3-phenylpentanamide (mixture of diastereomers A + B 1.25:1) (**21al**): yellow oil, *R*_f_ 0.31 (EtOAc/hexane, 1:1, *v*/*v*). Yield 0.853 g (3.7 mmol, 74%). ^1^H NMR (400 MHz, DMSO-*d*_6_) δ 7.93 (d, *J* = 2.4 Hz, 1H, B), 7.90 (s, 1H, A), 7.89 (d, *J* = 2.4 Hz, 1H, A), 7.84 (s, 1H, B), 7.64 (s, 1H, A), 7.41 (s, 1H, B), 7.39–7.15 (m, 15H, A + B), 6.71 (d, *J* = 8.9 Hz, 1H, B), 6.70 (d, *J* = 9.0 Hz, 1H, A), 4.08 (d, *J* = 9.1 Hz, 1H, A), 4.02–3.84 (m, 3H, A + B), 3.80 (dd, *J* = 17.3, 9.4 Hz, 1H, B), 3.68 (dd, *J* = 17.1, 9.9 Hz, 1H, A), 3.38 (dd, *J* = 17.3, 3.3 Hz, 1H, B), 3.22 (dd, *J* = 17.1, 4.0 Hz, 1H, A); ^13^C NMR (101 MHz, DMSO-*d*_6_) δ 197.81 (B), 197.75 (A), 165.8 (A), 165.7 (B), 150.2 (2C, A + B), 140.2 (B), 140.0 (A), 136.7 (2C, A + B), 132.81 (B), 132.76 (A), 128.3 (2C, A), 128.2 (4C, A + B), 128.1 (2C, B), 127.3 (B), 119.41 (B), 119.37 (A), 117.9 (B), 117.7 (A), 117.6 (B), 117.5 (A), 104.60 (B), 104.57 (A), 44.1 (A), 44.0 (B), 41.9 (A), 40.9 (A), 40.8 (B), 40.7 (B); FTIR, v_max_: 3353, 2936, 2245, 1680, 1609, 1544, 1312, 1205 cm^−1^; HRMS (ESI TOF) *m*/*z* calc’d. for C_18_H_16_BrN_3_NaO_2_ [M + Na]^+^: 408.0318, found: 408.0306 (2.9 ppm).

5-(2-Aminophenyl)-5-oxo-2,3-diphenylpentanamide (mixture of diastereomers A + B 4:1) (**21bb**): white solid, m.p. 197.2–199.9 °C (IPA), *R*_f_ 0.14 (EtOAc/hexane, 1:1, *v*/*v*). Yield 1.016 g (2.6 mmol, 52%). ^1^H NMR (400 MHz, DMSO-*d*_6_) δ 7.83 (d, *J* = 7.5 Hz, 1H, A), 7.68 (d, *J* = 2.5 Hz, 1H, A), 7.52 (d, *J* = 7.1 Hz, 2H, B), 7.45 (dd, *J* = 8.3, 1.5 Hz, 1H, B), 7.40–6.99 (m, 19H), 6.92 (d, *J* = 8.7 Hz, 2H, A), 6.76 (d, *J* = 8.6 Hz, 2H, B), 6.68 (d, *J* = 8.2 Hz, 1H, A), 6.62 (d, *J* = 8.3 Hz, 1H, B), 6.58 (d, *J* = 8.6 Hz, 2H, A), 6.53 (ddd, *J* = 8.1, 6.9, 1.2 Hz, 1H, A), 6.47 (s, 1H, B), 6.39 (ddd, *J* = 8.1, 6.8, 1.2 Hz, 1H, B), 3.99–3.77 (m, 4H, A + B), 3.68 (s, 3H, B), 3.57 (s, 3H, A), 3.33 (dd, *J* = 10.5, 5.0 Hz, 1H, A), 3.25 (dd, *J* = 11.2, 5.2 Hz, 1H, B), 3.20 (dd, *J* = 15.4, 2.8 Hz, 1H, A); ^13^C NMR (101 MHz, DMSO-*d*_6_) δ 200.3 (B), 200.0 (A), 174.4 (A), 173.2 (B), 157.4 (B), 157.1 (A), 151.1 (A), 150.9 (B), 139.6 (B), 139.2 (A), 135.0 (B), 134.0 (A), 133.9 (B), 133.7 (A), 131.2 (A), 130.9 (B), 129.4 (2C, B), 129.3 (2C, A), 128.5 (2C, B), 128.42 (2C, A), 128.36 (2C, B), 127.7 (2C, A), 127.0 (B), 126.3 (A), 117.0 (A), 116.9 (B), 116.6 (A), 114.3 (A), 114.1 (B), 113.1 (2C, B), 113.0 (2C, A), 57.18 (B), 57.12 (A), 54.8 (A), 54.7 (B), 44.3 (A), 43.8 (A), 42.9 (B), 42.7 (B); FTIR, v_max_: 3370, 2927, 2240, 1683, 1577, 1252, 1180 cm^−1^; HRMS (ESI TOF) *m*/*z* calc’d. for C_24_H_24_N_2_NaO_3_ [M + Na]^+^: 411.1679, found: 411.1683 (−1.0 ppm).

*N*-(2-(5-amino-4-cyano-3-(4-methoxyphenyl)-5-oxopentanoyl)phenyl)benzamide (mixture of diastereomers A + B 1:1) (**24**): Was synthetized via general method for acylation employing 5-(2-aminophenyl)-2-cyano-3-(4-methoxyphenyl)-5-oxopentanamide (674 mg, 2.00 mmol) and benzoylchloride (508 µL, 616 mg, 2.2 mmol). Light-yellow solid, m.p. 169.5–170.4 °C (EtOH), *R*_f_ 0.13 (EtOAc/hexane, 1:1, *v*/*v*). Yield 1.135 g (1.02 mmol, 51%). ^1^H NMR (400 MHz, DMSO-*d*_6_) δ 11.80 (s, 1H, A), 11.76 (s, 1H, B), 8.59–8.47 (m, 2H, A + B), 8.09 (dd, *J* = 8.1, 1.5 Hz, 1H, A), 8.03 (dd, *J* = 8.0, 1.6 Hz, 1H, B), 7.90–7.82 (m, 5H, A + B), 7.81 (s, 1H, A), 7.40 (s, 1H, A), 7.33–7.21 (m, 6H, A + B), 6.88–6.74 (m, 4H, A + B), 4.11 (d, *J* = 8.6 Hz, 1H, A), 4.00–3.75 (m, 4H, A + B), 3.71–3.68 (m, 1H, B), 3.66 (s, 3H, B), 3.65 (s, 3H, A), 3.47 (dd, *J* = 16.7, 5.0 Hz, 1H, A); ^13^C NMR (101 MHz, DMSO-*d*_6_) δ 202.52 (A), 202.47 (B), 165.7 (B), 165.6 (A), 165.1 (2C, A + B), 158.4 (B), 158.3 (A), 139.11 (A), 139.07 (B), 134.5 (A), 134.4 (A), 134.3 (B), 132.3 (B), 131.6 (A), 131.2 (B), 131.0 (A), 130.9 (B), 129.2 (2C, B), 129.1 (2C,A), 129.0 (4C, A + B), 127.1 (4C, A + B), 124.5 (A), 123.4 (A), 120.9 (B), 120.8 (A), 117.8 (2C, A), 117.7 (2C, B), 113.74 (2C, B), 113.66 (2C, A), 54.93 (B), 54.91 (A), 44.2 (B), 44.1 (A), 43.5 (B), 42.4 (A), 40.4 (A); FTIR, v_max_: 2943, 2244, 1685, 1513, 1449, 1304, 1260, 1180 cm^−1^; HRMS (ESI TOF) *m*/*z* calc’d. for C_26_H_23_N_3_NaO_4_ [M + Na]^+^: 464.1581, found: 464.1592 (−2.5 ppm).

### 3.4. Preparation of 3-(Quinazolin-4-yl)propionic Acids ***11*** and 4-(Quinazolin-4-yl)butyric Acids ***23*** (General Procedure)

A G10 microwave vial was charged with 1 mmol of 4-oxobutyronitrile **8** or 5-oxovaleronitrile **21**, TsOH●H_2_O (190 mg, 1 mmol), and HCOOH (4 mL, 80–85%). The vial was sealed and heated in the microwave apparatus at 140 °C for 30 min. For **11ba**–**da** pre-acylated starting materials **9ba**–**da** were used and HCOOH was replaced with AcOH. After consumption of starting material (TLC control), the reaction mixture was diluted with water (50 mL) and extracted with EtOAc (3 × 25 mL). Combined organic fractions were concentrated *in vacuo* and obtained residue was purified by column chromatography (PhH/EtOAc/AcOH, 15:4:1, *v*/*v*).

2-(4-Methoxyphenyl)-3-(quinazolin-4-yl)propanoic acid (**11aa**): white solid, m.p. 184.1–186.8 °C, *R*_f_ 0.37 (PhH/EtOAc/AcOH, 15:4:1, *v*/*v*). Yield 243 mg (0.79 mmol, 79%). ^1^H NMR (400 MHz, DMSO-*d*_6_) δ 12.31 (s, 1H), 9.18 (s, 1H), 8.38 (d, *J* = 8.4 Hz, 1H), 7.98 (d, *J* = 4.0 Hz, 2H), 7.80–7.67 (m, 1H), 7.37 (d, *J* = 8.7 Hz, 2H), 6.91 (d, *J* = 8.7 Hz, 2H), 4.45 (dd, *J* = 10.3, 4.5 Hz, 1H), 4.13 (dd, *J* = 17.1, 10.3 Hz, 1H), 3.73 (s, 3H), 3.53 (dd, *J* = 17.1, 4.6 Hz, 1H); ^13^C NMR (101 MHz, DMSO-*d*_6_) δ 174.6, 168.9, 158.4, 154.0, 148.9, 134.1, 131.2, 129.1 (2C), 128.3, 128.0, 125.3, 123.5, 114.0 (2C), 55.1, 47.1, 36.6; FTIR, v_max_: 1749, 1716, 1634, 1558, 1439, 1244 cm^−1^; HRMS (ESI TOF) *m*/*z* calc’d. for C_18_H_15_N_2_Na_2_O_3_ [M-H + 2Na]^+^: 353.0873, found: 353.0881 (−2.4 ppm).

2-Phenyl-3-(quinazolin-4-yl)propanoic acid (**11ab**): white solid, m.p. 189.9–191.5 °C, *R*_f_ 0.39 (PhH/EtOAc/AcOH, 15:4:1, *v*/*v*). Yield 173 mg (0.62 mmol, 62%). ^1^H NMR (400 MHz, DMSO-*d*_6_) δ 12.35 (s, 1H), 9.18 (s, 1H), 8.39 (d, *J* = 8.4 Hz, 1H), 7.99 (d, *J* = 3.8 Hz, 2H), 7.78–7.66 (m, 1H), 7.45 (d, *J* = 7.3 Hz, 2H), 7.36 (t, *J* = 7.5 Hz, 2H), 7.28 (t, *J* = 7.3 Hz, 1H), 4.50 (dd, *J* = 10.3, 4.4 Hz, 1H), 4.16 (dd, *J* = 17.2, 10.4 Hz, 1H), 3.57 (dd, *J* = 17.1, 4.5 Hz, 1H); ^13^C NMR (101 MHz, DMSO-*d*_6_) δ 174.3, 168.8, 154.0, 148.9, 139.4, 134.1, 128.7 (2C), 128.3, 128.1 (2C), 128.0, 127.2, 125.3, 123.5, 48.0, 36.5; FTIR, v_max_: 1717, 1558, 1498, 1455, 1271, 1196 cm^−1^; HRMS (ESI TOF) *m*/*z* calc’d. for C_17_H_13_N_2_Na_2_O_2_ [M-H + 2Na]^+^: 323.0767, found: 323.0771 (−1.3 ppm).

2-(2-Methoxyphenyl)-3-(quinazolin-4-yl)propanoic acid (**11ac**): white solid, m.p. 197.9–200.6 °C, *R*_f_ 0.31 (PhH/EtOAc/AcOH, 15:4:1, *v*/*v*). Yield 200 mg (0.65 mmol, 65%). ^1^H NMR (400 MHz, DMSO-*d*_6_) δ 12.28 (s, 1H), 9.17 (s, 1H), 8.30 (d, *J* = 8.3 Hz, 1H), 8.02–7.95 (m, 2H), 7.75–7.65 (m, 1H), 7.25 (t, *J* = 7.6 Hz, 2H), 6.99 (d, *J* = 8.2 Hz, 1H), 6.92 (td, *J* = 7.5, 1.1 Hz, 1H), 4.86 (dd, *J* = 9.7, 4.9 Hz, 1H), 4.02 (dd, *J* = 16.6, 9.7 Hz, 1H), 3.73 (s, 3H), 3.44 (dd, *J* = 16.6, 4.9 Hz, 1H); ^13^C NMR (101 MHz, DMSO-*d*_6_) δ 174.5, 169.1, 156.5, 154.0, 148.9, 134.0, 128.4, 128.3, 128.2, 127.9, 127.6, 125.2, 123.5, 120.6, 111.3, 55.5, 41.6, 35.8; FTIR, v_max_: 1699, 1495, 1247, 1203, 1050 cm^−1^; HRMS (ESI TOF) *m*/*z* calc’d. for C_18_H_15_N_2_Na_2_O_3_ [M-H + 2Na]^+^: 353.0873, found: 353.0866 (1.9 ppm).

3-(Quinazolin-4-yl)-2-(*o*-tolyl)propanoic acid (**11ad**): white solid, m.p. 228.5–233.9 °C, *R*_f_ 0.43 (PhH/EtOAc/AcOH, 15:4:1, *v*/*v*). Yield 198 mg (0.68 mmol, 68%). ^1^H NMR (400 MHz, DMSO-*d*_6_) δ 12.36 (s, 1H), 9.19 (s, 1H), 8.38 (d, *J* = 8.4 Hz, 1H), 7.99 (d, *J* = 4.0 Hz, 2H), 7.82–7.64 (m, 1H), 7.47–7.33 (m, 1H), 7.25–7.09 (m, 3H), 4.78 (dd, *J* = 10.0, 4.5 Hz, 1H), 4.11 (dd, *J* = 17.2, 10.0 Hz, 1H), 3.52 (dd, *J* = 17.2, 4.6 Hz, 1H), 2.39 (s, 3H); ^13^C NMR (101 MHz, DMSO-*d*_6_) δ 174.6, 168.9, 154.0, 148.9, 137.9, 136.0, 134.1, 130.5, 128.3, 127.9, 127.0, 126.8, 126.3, 125.3, 123.5, 43.6, 35.9, 19.4; FTIR, v_max_: 1717, 1652, 1558, 1419, 1268, 1160 cm^−1^; HRMS (ESI TOF) *m*/*z* calc’d. for C_18_H_15_N_2_Na_2_O_2_ [M-H + 2Na]^+^: 337.0923, found: 337.0920 (0.9 ppm).

2-(4-Ethylphenyl)-3-(quinazolin-4-yl)propanoic acid (**11ae**): white solid, m.p. 208.4–209.9 °C, *R*_f_ 0.44 (PhH/EtOAc/AcOH, 15:4:1, *v*/*v*). Yield 230 mg (0.75 mmol, 75%). ^1^H NMR (400 MHz, DMSO-*d*_6_) δ 12.34 (s, 1H), 9.18 (s, 1H), 8.37 (d, *J* = 8.4 Hz, 1H), 8.09–7.91 (m, 2H), 7.83–7.59 (m, 1H), 7.36 (d, *J* = 8.1 Hz, 2H), 7.19 (d, *J* = 7.9 Hz, 2H), 4.48 (dd, *J* = 10.4, 4.3 Hz, 1H), 4.15 (dd, *J* = 17.2, 10.5 Hz, 1H), 3.54 (dd, *J* = 17.2, 4.4 Hz, 1H), 2.57 (q, *J* = 7.6 Hz, 2H), 1.16 (t, *J* = 7.6 Hz, 3H).; ^13^C NMR (101 MHz, DMSO-*d*_6_) δ 174.5, 168.8, 154.0, 148.9, 142.7, 136.6, 134.1, 128.3, 128.1 (2C), 128.0 (3C), 125.2, 123.5, 47.5, 36.5, 27.9, 15.7; FTIR, v_max_: 1685, 1558, 1498, 1436, 1314, 1239 cm^−1^; HRMS (ESI TOF) *m*/*z* calc’d. for C_19_H_17_N_2_Na_2_O_2_ [M-H + 2Na]^+^: 351.1080, found: 351.1077 (0.8 ppm).

2-(4-Isopropylphenyl)-3-(quinazolin-4-yl)propanoic acid (**11af**): white solid, m.p. 210.2–214.4 °C, *R*_f_ 0.47 (PhH/EtOAc/AcOH, 15:4:1, *v*/*v*). Yield 227 mg (0.7 mmol, 70%). ^1^H NMR (400 MHz, DMSO-*d*_6_) δ 11.99 (s, 1H), 8.82 (s, 1H), 8.01 (d, *J* = 8.4 Hz, 1H), 7.63 (d, *J* = 4.0 Hz, 2H), 7.44–7.28 (m, 1H), 7.01 (d, *J* = 7.7 Hz, 2H), 6.87 (d, *J* = 7.7 Hz, 2H), 4.11 (dd, *J* = 10.6, 4.3 Hz, 1H), 3.79 (dd, *J* = 17.2, 10.6 Hz, 1H), 3.18 (dd, *J* = 17.3, 4.3 Hz, 1H), 2.21–2.07 (m, 1H), 0.83 (s, 3H), 0.82 (s, 3H); ^13^C NMR (101 MHz, DMSO-*d*_6_) δ 174.4, 168.8, 154.0, 148.9, 147.3, 136.7, 134.1, 128.3, 128.0 (2C), 126.6 (2C), 125.3, 123.6, 47.6, 36.6, 33.2, 24.0, 23.9 (2C); FTIR, v_max_: 2957, 1736, 1652, 1558, 1506, 1261 cm^−1^; HRMS (ESI TOF) *m*/*z* calc’d. for C_20_H_19_N_2_Na_2_O_2_ [M-H + 2Na]^+^: 365.1236, found: 365.1233 (0.8 ppm).

2-(2-Chlorophenyl)-3-(quinazolin-4-yl)propanoic acid (**11ag**): gray solid, m.p. 221.3–222.9 °C, *R*_f_ 0.41 (PhH/EtOAc/AcOH, 15:4:1, *v*/*v*). Yield 189 mg (0.61 mmol, 61%). ^1^H NMR (400 MHz, DMSO-*d*_6_) δ 12.65 (s, 1H), 9.19 (s, 1H), 8.35 (d, *J* = 8.4 Hz, 1H), 8.01–7.96 (m, 2H), 7.76–7.69 (m, 1H), 7.52 (dd, *J* = 7.6, 1.8 Hz, 1H), 7.47 (dd, *J* = 7.8, 1.5 Hz, 1H), 7.36 (td, *J* = 7.5, 1.5 Hz, 1H), 7.30 (td, *J* = 7.6, 1.8 Hz, 1H), 5.05 (dd, *J* = 9.7, 4.8 Hz, 1H), 4.14 (dd, *J* = 17.0, 9.7 Hz, 1H), 3.56 (dd, *J* = 16.9, 4.9 Hz, 1H); ^13^C NMR (101 MHz, DMSO-*d*_6_) δ 173.6, 168.4, 154.0, 149.0, 137.1, 134.2, 133.2, 129.7, 129.2, 129.0, 128.4, 128.0, 127.7, 125.2 123.5, 44.7, 35.6; FTIR, v_max_: 2993, 1657, 153, 1508, 1453, 1235 cm^−1^; HRMS (ESI TOF) *m*/*z* calc’d. for C_17_H_12_ClN_2_Na_2_O_2_ [M-H + 2Na]^+^: 357.0377, found: 357.0387 (−2.8 ppm).

2-(3-Chlorophenyl)-3-(quinazolin-4-yl)propanoic acid (**11ah**): white solid, m.p. 194.8–200.3 °C, *R*_f_ 0.4 (PhH/EtOAc/AcOH, 15:4:1, *v*/*v*). Yield 179 mg (0.57 mmol, 57%). ^1^H NMR (400 MHz, DMSO-*d*_6_) δ 12.55 (s, 1H), 9.18 (s, 1H), 8.43 (d, *J* = 8.4 Hz, 1H), 8.04–7.91 (m, 2H), 7.79–7.68 (m, 1H), 7.55 (s, 1H), 7.43 (td, *J* = 7.3, 3.7 Hz, 1H), 7.37 (dd, *J* = 11.4, 7.7 Hz, 2H), 4.55 (dd, *J* = 10.3, 4.4 Hz, 1H), 4.19 (dd, *J* = 17.3, 10.4 Hz, 1H), 3.61 (dd, *J* = 17.2, 4.5 Hz, 1H); ^13^C NMR (101 MHz, DMSO-*d*_6_) δ 173.9, 168.5, 153.9, 148.9, 141.7, 134.2, 133.2, 130.5, 128.3, 128.0 (2C), 127.3, 127.0, 125.4, 123.5, 47.5, 36.1; FTIR, v_max_: 1702, 1582, 1497, 1353, 1263, 1175 cm^−1^; HRMS (ESI TOF) *m*/*z* calc’d. for C_17_H_12_ClN_2_Na_2_O_2_ [M-H + 2Na]^+^: 357.0377, found: 357.0368 (2.5 ppm).

2-(4-Chlorophenyl)-3-(quinazolin-4-yl)propanoic acid (**11ai**): white solid, m.p. 172.8–181.5 °C, *R*_f_ 0.37 (PhH/EtOAc/AcOH, 15:4:1, *v*/*v*). Yield 186 mg (0.6 mmol, 60%). ^1^H NMR (400 MHz, DMSO-*d*_6_) δ 12.51 (s, 1H), 9.17 (s, 1H), 8.40 (d, *J* = 8.3 Hz, 1H), 7.99 (d, *J* = 3.9 Hz, 2H), 7.82–7.67 (m, 1H), 7.49 (d, *J* = 8.3 Hz, 2H), 7.42 (d, *J* = 8.3 Hz, 2H), 4.53 (dd, *J* = 10.2, 4.7 Hz, 1H), 4.16 (dd, *J* = 17.2, 10.2 Hz, 1H), 3.59 (dd, *J* = 17.2, 4.7 Hz, 1H); ^13^C NMR (101 MHz, DMSO-*d*_6_) δ 174.0, 168.5, 153.9, 148.9, 138.3, 134.1, 131.9, 130.0 (2C), 128.6 (2C), 128.3, 128.0, 125.2, 123.4, 47.2, 36.2; FTIR, v_max_: 1712, 1568, 1486, 1419, 1238, 1166 cm^−1^; HRMS (ESI TOF) *m*/*z* calc’d. for C_17_H_12_ClN_2_Na_2_O_2_ [M-H + 2Na]^+^: 357.0377, found: 357.0384 (−2.0 ppm).

2-(2-Fluorophenyl)-3-(quinazolin-4-yl)propanoic acid (**11aj**): white solid, m.p. 197.5–200.2 °C, *R*_f_ 0.38 (PhH/EtOAc/AcOH, 15:4:1, *v*/*v*). Yield 162 mg (0.55 mmol, 55%). ^1^H NMR (400 MHz, DMSO-*d*_6_) δ 12.61 (s, 1H), 9.17 (s, 1H), 8.37 (d, *J* = 8.4 Hz, 1H), 8.01–7.91 (m, 2H), 7.82–7.60 (m, 1H), 7.55–7.41 (m, 1H), 7.39–7.24 (m, 1H), 7.26–7.10 (m, 2H), 4.83 (dd, *J* = 9.4, 5.3 Hz, 1H), 4.18 (dd, *J* = 16.9, 9.5 Hz, 1H), 3.58 (dd, *J* = 16.9, 5.4 Hz, 1H); ^13^C NMR (101 MHz, DMSO-*d*_6_) δ 173.5, 168.5, 160.1 (d, *J* = 244.7 Hz), 154.0, 149.0, 134.2, 129.6 (d, *J* = 3.9 Hz), 129.2 (d, *J* = 8.3 Hz), 128.4, 128.0, 126.4 (d, *J* = 15.0 Hz), 125.2, 124.7 (d, *J* = 3.3 Hz), 123.5, 115.6 (d, *J* = 22.1 Hz), 41.0 (d, *J* = 1.9 Hz), 35.2; ^19^F NMR (376 MHz, DMSO) δ −117.70; FTIR, v_max_: 1716, 1560, 1494, 1419, 129, 1260, 1240 cm^−1^; HRMS (ESI TOF) *m*/*z* calc’d. for C_17_H_12_FN_2_Na_2_O_2_ [M-H + 2Na]^+^: 341.0673, found: 341.0672 (0.1 ppm).

2-(4-Fluorophenyl)-3-(quinazolin-4-yl)propanoic acid (**11ak**): white solid, m.p. 178.1–179.5 °C, *R*_f_ 0.37 (PhH/EtOAc/AcOH, 15:4:1, *v*/*v*). Yield 252 mg (0.85 mmol, 85%). ^1^H NMR (400 MHz, DMSO-*d*_6_) δ 12.50 (s, 1H), 9.18 (s, 1H), 8.40 (d, *J* = 8.4 Hz, 1H), 7.99 (d, *J* = 4.0 Hz, 2H), 7.82–7.64 (m, 1H), 7.57–7.44 (m, 2H), 7.19 (t, *J* = 8.9 Hz, 2H), 4.54 (dd, *J* = 10.3, 4.6 Hz, 1H), 4.17 (dd, *J* = 17.2, 10.3 Hz, 1H), 3.58 (dd, *J* = 17.1, 4.6 Hz, 1H); ^13^C NMR (101 MHz, DMSO-*d*_6_) δ 174.3, 168.6, 161.4 (d, *J* = 243.0 Hz), 154.0, 148.9, 135.5 (d, *J* = 3.1 Hz), 134.1, 130.0 (2C, d, *J* = 8.1 Hz), 128.3, 128.0, 125.3, 123.5, 115.4 (2C, d, *J* = 21.2 Hz), 47.1, 36.4; ^19^F NMR (376 MHz, DMSO) δ −115.59; FTIR, v_max_: 1716, 1683, 1558, 1458, 1238, 1174 cm^−1^; HRMS (ESI TOF) *m*/*z* calc’d. for C_17_H_12_FN_2_Na_2_O_2_ [M-H + 2Na]^+^: 341.0673, found: 341.0682 (−2.8 ppm).

3-(6-Bromoquinazolin-4-yl)-2-phenylpropanoic acid (**11al**): white solid, m.p. 211.3–212.5 °C, *R*_f_ 0.54 (PhH/EtOAc/AcOH, 15:4:1, *v*/*v*). Yield 244 mg (0.68 mmol, 68%). ^1^H NMR (400 MHz, DMSO-*d*_6_) δ 12.39 (s, 1H), 9.22 (s, 1H), 8.66 (d, *J* = 2.1 Hz, 1H), 8.09 (dd, *J* = 8.9, 2.2 Hz, 1H), 7.92 (d, *J* = 8.9 Hz, 1H), 7.55–7.44 (m, 2H), 7.36 (dd, *J* = 8.3, 6.7 Hz, 2H), 7.31–7.18 (m, 1H), 4.49 (dd, *J* = 10.6, 4.2 Hz, 1H), 4.21 (dd, *J* = 17.4, 10.6 Hz, 1H), 3.53 (dd, *J* = 17.4, 4.3 Hz, 1H); ^13^C NMR (101 MHz, DMSO-*d*_6_) δ 174.3, 168.5, 154.3, 147.6, 139.2, 137.3, 130.6, 128.6 (2C), 128.2 (2C), 127.7, 127.2, 124.6, 120.9, 47.9, 36.4; FTIR, v_max_: 1733, 1558, 1540, 1453, 1419, 1250, 1145 cm^−1^; HRMS (ESI TOF) *m*/*z* calc’d. for C_17_H_12_BrN_2_Na_2_O_2_ [M-H + 2Na]^+^: 400.9872, found: 400.9881 (−2.2 ppm).

3-(6,7-Dimethoxyquinazolin-4-yl)-2-phenylpropanoic acid (**11am**): white solid, m.p. 193.4–196.5 °C, *R*_f_ 0.16 (PhH/EtOAc/AcOH, 15:4:1, *v*/*v*). Yield 206 mg (0.61 mmol, 61%). ^1^H NMR (400 MHz, DMSO-*d*_6_) δ 12.35 (s, 1H), 8.96 (s, 1H), 7.48–7.41 (m, 3H), 7.35 (t, *J* = 7.5 Hz, 2H), 7.31 (s, 1H), 7.30–7.23 (m, 1H), 4.46 (dd, *J* = 10.2, 4.5 Hz, 1H), 4.03 (dd, *J* = 17.1, 10.3 Hz, 1H), 3.96 (s, 3H), 3.95 (s, 3H), 3.49 (dd, *J* = 17.1, 4.6 Hz, 1H); ^13^C NMR (101 MHz, DMSO-*d*_6_) δ 174.5, 165.0, 155.4, 152.6, 149.9, 146.9, 139.6, 128.6 (2C), 128.1 (2C), 127.1, 119.0, 106.7, 102.5, 56.09, 56.06, 48.1, 36.6; FTIR, v_max_: 1718, 1634, 1556, 1455, 1416, 1238, 1174 cm^−1^; HRMS (ESI TOF) *m*/*z* calc’d. for C_19_H_17_N_2_Na_2_O_4_ [M-H + 2Na]^+^: 383.0978, found: 383.0987 (−2.3 ppm).

2-(4-Methoxyphenyl)-3-(2-methylquinazolin-4-yl)propanoic acid (**11ba**): Was prepared according to general procedure with AcOH instead of HCOOH. White solid, decomposition 198.2 °C, *R*_f_ 0.31 (PhH/EtOAc/AcOH, 15:4:1, *v*/*v*). Yield 155 mg (0.48 mmol, 48%). ^1^H NMR (400 MHz, DMSO-*d*_6_) δ 12.29 (s, 1H), 8.30 (dd, *J* = 8.4, 1.2 Hz, 1H), 7.95–7.84 (m, 2H), 7.63 (ddd, *J* = 8.3, 6.6, 1.5 Hz, 1H), 7.36 (d, *J* = 8.7 Hz, 1H), 6.90 (d, *J* = 8.7 Hz, 1H), 4.41 (dd, *J* = 10.1, 4.7 Hz, 1H), 4.06 (dd, *J* = 16.9, 10.2 Hz, 1H), 3.73 (s, 3H), 3.49 (dd, *J* = 17.0, 4.8 Hz, 1H); ^13^C NMR (101 MHz, DMSO-*d*_6_) δ 174.7, 168.6, 162.5, 158.4, 149.4, 133.9, 131.3, 129.1 (2C), 127.7, 126.9, 125.1, 121.4, 114.0 (2C), 55.1, 47.2, 36.5, 26.1; FTIR, v_max_: 1699, 1576, 1251, 1113 cm^−1^; HRMS (ESI TOF) *m*/*z* calc’d. for C_19_H_17_N_2_Na_2_O_3_ [M-H + 2Na]^+^: 367.1029, found: 367.1034 (−1.5 ppm).

3-(2-Ethylquinazolin-4-yl)-2-(4-methoxyphenyl)propanoic acid (**11ca**): Was prepared according to general procedure with AcOH instead of HCOOH. White solid, m.p. 172.1–173.9 °C, *R*_f_ 0.49 (PhH/EtOAc/AcOH, 15:4:1, *v*/*v*). Yield 290 mg (0.86 mmol, 86%). ^1^H NMR (400 MHz, DMSO-*d*_6_) δ 12.28 (s, 1H), 8.30 (d, *J* = 8.3 Hz, 1H), 8.03–7.82 (m, 2H), 7.63 (ddd, *J* = 8.3, 6.1, 2.0 Hz, 1H), 7.37 (d, *J* = 8.7 Hz, 2H), 6.90 (d, *J* = 8.7 Hz, 1H), 4.42 (dd, *J* = 10.2, 4.7 Hz, 1H), 4.06 (dd, *J* = 17.1, 10.2 Hz, 1H), 3.73 (s, 3H), 3.53 (dd, *J* = 17.2, 4.8 Hz, 1H), 2.98 (qd, *J* = 7.4, 2.8 Hz, 2H), 1.35 (t, *J* = 7.6 Hz, 3H); ^13^C NMR (101 MHz, DMSO-*d*_6_) δ 174.7, 168.6, 166.4, 158.4, 149.3, 133.8, 131.3, 129.1 (2C), 127.9, 126.9, 125.0, 121.6, 114.0 (2C), 55.1, 47.1, 36.6, 32.3, 12.5; FTIR, v_max_: 1701, 1560, 1508, 1420, 1247, 1113 cm^−1^; HRMS (ESI TOF) *m*/*z* calc’d. for C_20_H_19_N_2_Na_2_O_3_ [M-H + 2Na]^+^: 381.1186, found: 381.1187 (−0.4 ppm).

2-(4-Methoxyphenyl)-3-(2-phenylquinazolin-4-yl)propanoic acid (**11da**): white solid, m.p. 210.4–222.6 °C, *R*_f_ 0.68 (PhH/EtOAc/AcOH, 28:4.5:1, *v*/*v*). Yield 295 mg (0.77 mmol, 77%). ^1^H NMR (400 MHz, DMSO-*d*_6_) δ 12.41 (s, 1H), 8.74–8.57 (m, 2H), 8.36 (d, *J* = 8.3 Hz, 1H), 8.04 (d, *J* = 8.8 Hz, 1H), 8.02–7.93 (m, 1H), 7.69 (t, *J* = 7.5 Hz, 1H), 7.61–7.51 (m, 3H), 7.45 (d, *J* = 8.7 Hz, 2H), 6.95 (d, *J* = 8.7 Hz, 2H), 4.54 (dd, *J* = 10.8, 4.0 Hz, 1H), 4.17 (dd, *J* = 17.6, 10.8 Hz, 1H), 3.75 (s, 3H), 3.66 (dd, *J* = 17.7, 4.2 Hz, 1H); ^13^C NMR (101 MHz, DMSO-*d*_6_) δ 174.9, 169.0, 158.5, 158.5, 149.5, 137.6, 134.3, 131.3, 130.8, 129.1 (2C), 128.7 (2C), 128.6 (2C), 128.3, 127.6, 125.2, 122.1, 114.1 (2C), 55.1, 46.9, 37.1; FTIR, v_max_: 1733, 1560, 1508,1250, 1178, 1030 cm^−1^; HRMS (ESI TOF) *m*/*z* calc’d. for C_24_H_19_N_2_Na_2_O_3_ [M-H + 2Na]^+^: 429.1186, found: 429.1193 (−1.6 ppm).

3-(4-Methoxyphenyl)-4-(quinazolin-4-yl)butanoic acid (**23aa**): white solid, m.p. 152.1–153.9 °C (PhH), *R*_f_ 0.19 (PhH/EtOAc/AcOH, 15:4:1, *v*/*v*). Yield 149 mg (0.46 mmol, 46%). ^1^H NMR (400 MHz, DMSO-*d*_6_) δ 12.09 (s, 1H), 9.13 (s, 1H), 8.32 (d, *J* = 8.4 Hz, 1H), 7.94 (d, *J* = 3.9 Hz, 2H), 7.69 (dq, *J* = 8.3, 4.5, 4.1 Hz, 1H), 7.15 (d, *J* = 8.6 Hz, 2H), 6.72 (d, *J* = 8.6 Hz, 2H), 3.88–3.71 (m, 1H), 3.64 (s, 3H), 3.58 (d, *J* = 7.5 Hz, 2H), 2.76 (dd, *J* = 15.8, 6.4 Hz, 1H), 2.64 (dd, *J* = 15.8, 8.6 Hz, 1H); ^13^C NMR (101 MHz, DMSO-*d*_6_) δ 173.1, 169.6, 157.7, 154.1, 149.3 135.3, 133.9, 128.5 (2C), 128.4, 127.8, 125.4, 123.8, 113.5 (2C), 54.9, 40.6, 40.3, 39.8; FTIR, v_max_: 1708, 1609, 1513, 1412, 1304, 1252, 1180 cm^−1^; HRMS (ESI TOF) *m*/*z* calc’d. for C_19_H_17_N_2_Na_2_O_3_ [M-H + 2Na]^+^: 367.1029, found: 367.1038 (−2.3 ppm).

3-(4-Ethylphenyl)-4-(quinazolin-4-yl)butanoic acid (**23ae**): white solid, m.p. 156.7–158.1 (PhH) °C, *R*_f_ 0.26 (PhH/EtOAc/AcOH, 15:4:1, *v*/*v*). Yield 151 mg (0.47 mmol, 47%). ^1^H NMR (400 MHz, DMSO-*d*_6_) δ 12.11 (s, 1H), 9.14 (s, 1H), 8.31 (d, *J* = 8.4 Hz, 1H), 7.95 (d, *J* = 3.9 Hz, 2H), 7.69 (dq, *J* = 8.3, 4.5, 4.1 Hz, 1H), 7.16 (d, *J* = 8.0 Hz, 2H), 7.01 (d, *J* = 7.9 Hz, 2H), 3.95–3.74 (m, 1H), 3.59 (d, *J* = 7.5 Hz, 2H), 2.77 (dd, *J* = 15.9, 6.3 Hz, 1H), 2.67 (dd, *J* = 15.9, 8.6 Hz, 1H), 2.47 (q, *J* = 7.7 Hz, 2H), 1.08 (t, *J* = 7.6 Hz, 3H); ^13^C NMR (101 MHz, DMSO-*d*_6_) δ 172.9, 169.2, 153.9, 149.1, 141.4, 140.5, 133.7, 128.2, 127.5, 127.3 (2C), 127.2 (2C), 125.1, 123.6, 40.3, 40.1, 39.7, 27.5, 15.3; FTIR, v_max_: 1716, 1499, 1293, 1211, 1117 cm^−1^; HRMS (ESI TOF) *m*/*z* calc’d. for C_20_H_19_N_2_Na_2_O_2_ [M-H + 2Na]^+^: 365.1236, found: 365.1234 (0.7 ppm).

3-(4-Chlorophenyl)-4-(quinazolin-4-yl)butanoic acid (**23ah**): white solid, m.p. 199.4–200.9 °C, *R*_f_ 0.21 (PhH/EtOAc/AcOH, 15:4:1, *v*/*v*). Yield 188 mg (0.58 mmol, 58%). ^1^H NMR (400 MHz, DMSO-*d*_6_) δ 12.15 (s, 1H), 9.12 (s, 1H), 8.34 (d, *J* = 8.4 Hz, 1H), 8.07–7.89 (m, 2H), 7.82–7.60 (m, 1H), 7.27 (d, *J* = 8.6 Hz, 2H), 7.22 (d, *J* = 8.6 Hz, 2H), 3.92–3.77 (m, 1H), 3.72–3.48 (m, 2H), 2.81 (dd, *J* = 16.0, 6.2 Hz, 1H), 2.69 (dd, *J* = 16.0, 8.7 Hz, 1H); ^13^C NMR (101 MHz, DMSO-*d*_6_) δ 172.9, 169.2, 154.1, 149.3, 142.4, 134.0, 130.9, 129.6 (2C), 128.4, 128.0 (2C), 127.8, 125.3, 123.8, 40.3, 40.1, 39.3; FTIR, v_max_: 1706, 1618, 1501, 1412, 1238, 1215 cm^−1^; HRMS (ESI TOF) *m*/*z* calc’d. for C_18_H_14_ClN_2_Na_2_O_2_ [M-H + 2Na]^+^: 371.0534, found: 371.0534 (0.0 ppm).

4-(6-Bromoquinazolin-4-yl)-3-phenylbutanoic acid (**23al**): white solid, m.p. 179.8–181.5 °C, *R*_f_ 0.34 (PhH/EtOAc/AcOH, 15:4:1, *v*/*v*). Yield 205 mg (0.55 mmol, 55%). ^1^H NMR (400 MHz, DMSO-*d*_6_) δ 12.15 (s, 1H), 9.15 (s, 1H), 8.50 (d, *J* = 2.2 Hz, 1H), 8.03 (dd, *J* = 8.9, 2.1 Hz, 1H), 7.85 (d, *J* = 8.9 Hz, 1H), 7.21 (d, *J* = 7.3 Hz, 2H), 7.15 (t, *J* = 7.5 Hz, 2H), 7.07 (t, *J* = 7.1 Hz, 1H), 3.85–3.71 (m, 1H), 3.70–3.54 (m, 2H), 2.83 (dd, *J* = 16.0, 6.7 Hz, 1H), 2.69 (dd, *J* = 16.0, 8.2 Hz, 1H); ^13^C NMR (101 MHz, DMSO-*d*_6_) δ 173.1, 169.0, 154.4, 148.0, 143.2, 137.0, 130.6, 128.1 (2C), 127.7, 127.6 (2C), 126.4, 125.0, 120.6, 41.0, 40.2, 39.7; FTIR, v_max_: 1708, 1570, 1560, 1497, 1207, 1014 cm^−1^; HRMS (ESI TOF) *m*/*z* calc’d. for C_18_H_14_BrN_2_Na_2_O_2_ [M-H + 2Na]^+^: 415.0029, found: 415.0034 (−1.3 ppm).

3-(4-Methoxyphenyl)-2-phenyl-4-(quinazolin-4-yl)butanoic acid (**23bb**): white solid, m.p. 213.2–215.3 °C, *R*_f_ 0.23 (PhH/EtOAc/AcOH, 15:4:1, *v*/*v*). Yield 220 mg (0.55 mmol, 55%). ^1^H NMR (400 MHz, DMSO-*d*_6_) δ 12.71 (s, 1H), 9.05 (s, 1H), 8.33 (d, *J* = 8.4 Hz, 1H), 7.97–7.81 (m, 2H), 7.68 (ddd, *J* = 8.3, 6.0, 2.1 Hz, 1H), 7.21 (d, *J* = 7.1 Hz, 2H), 7.13 (t, *J* = 7.5 Hz, 2H), 7.05 (t, *J* = 7.2 Hz, 1H), 6.86 (d, *J* = 8.4 Hz, 2H), 6.41 (d, *J* = 8.4 Hz, 2H), 4.21–4.02 (m, 2H), 3.80 (dd, *J* = 13.5, 9.8 Hz, 1H), 3.61 (dd, *J* = 13.6, 3.4 Hz, 1H), 3.46 (s, 3H); ^13^C NMR (101 MHz, DMSO-*d*_6_) δ 174.8, 169.5, 157.1, 154.0, 149.2, 138.0, 133.8, 132.1, 129.4 (2C), 128.5 (2C), 128.3, 128.1 (2C), 127.6, 126.8, 125.3, 123.8, 112.9 (2C), 57.1, 54.6, 47.1; FTIR, v_max_: 1703, 1570, 1512, 1280, 1253, 1202 cm^−1^; HRMS (ESI TOF) *m*/*z* calc’d. for C_25_H_21_N_2_Na_2_O_3_ [M-H + 2Na]^+^: 443.1342, found: 443.1354 (−2.6 ppm).

### 3.5. Isolation of Intermediates ***9aa*** and ***16aa*** (Control Experiment)

A G10 microwave vial was charged with 4-(2-aminophenyl)-2-(4-methoxyphenyl)-4-oxobutanenitrile **8a** (280 mg, 1 mmol), TsOH●H_2_O (190 mg, 1 mmol), and HCOOH (4 mL, 80–85%). The vial was sealed and heated in the microwave apparatus at 110 °C for 10 min. After this period of time, the reaction mixture was diluted with water (50 mL) and extracted with EtOAc (3 × 25 mL). The combined organic fractions were concentrated *in vacuo* and obtained residue was purified by column chromatography (gradient: EtOAc/Hexane, 1:2–2:1, *v*/*v*) to give a mixture of **11aa** (70 mg, 35%), **9aa** (46 mg, 15%) and **16aa** (134 mg, 41%). Intermediates **9aa** and **16aa** were converted to quinazoline **11aa** under standard conditions in 82% and 86% yields, respectively.

***N*-(2-(3-Cyano-3-(4-methoxyphenyl)propanoyl)phenyl)formamide (9aa):** white solid, m.p. 94.3–96.5 °C (MeOH), *R*_f_ 0.29 (EtOAc/Hexane, 1:2, *v*/*v*). ^1^H NMR (400 MHz, DMSO-*d*_6_) δ 11.14 (s, 1H), 8.49 (s, 1H), 8.45 (d, *J* = 8.4 Hz, 1H), 8.07 (dd, *J* = 8.0, 1.5 Hz, 1H), 7.68–7.54 (m, 1H), 7.44 (d, *J* = 8.7 Hz, 2H), 7.22 (t, *J* = 7.6 Hz, 1H), 6.97 (d, *J* = 8.7 Hz, 2H), 4.50 (dd, *J* = 9.1, 5.0 Hz, 1H), 4.01 (dd, *J* = 18.4, 9.1 Hz, 1H), 3.75 (s, 3H), 3.71 (dd, *J* = 18.4, 5.2 Hz, 1H); ^13^C NMR (101 MHz, DMSO-*d*_6_) δ 199.8, 161.4, 159.0, 138.5, 134.6, 131.3, 129.0 (2C), 127.5, 123.4, 122.9, 121.6, 121.2, 114.3 (2C), 55.2, 44.2, 30.5; FTIR, v_max_: 3222, 3014, 2242, 1694, 1582, 1511, 1298 cm^−1^; HRMS (ESI TOF) *m*/*z* calc’d. for C_18_H_16_N_2_NaO_3_ [M + Na]^+^: 331.1053, found: 331.1058 (−1.5 ppm).

**4-(2-Formamidophenyl)-2-(4-methoxyphenyl)-4-oxobutanamide (16aa):** white solid, m.p. 141.6–143.0 °C (PhH), *R*_f_ 0.14 (EtOAc/Hexane, 2:1, *v*/*v*). ^1^H NMR (400 MHz, DMSO-*d*_6_) δ 11.17 (s, 1H), 8.50–8.38 (m, 2H), 8.12 (d, *J* = 8.0 Hz, 1H), 7.66–7.56 (m, 1H), 7.54–7.48 (m, 1H), 7.31 (d, *J* = 8.6 Hz, 2H), 7.26–7.17 (m, 1H), 6.88 (d, *J* = 8.7 Hz, 3H), 4.00 (dd, *J* = 10.2, 3.8 Hz, 1H), 3.88 (dd, *J* = 17.7, 10.3 Hz, 1H), 3.72 (s, 3H), 3.19 (dd, *J* = 17.5, 3.8 Hz, 1H); ^13^C NMR (101 MHz, DMSO-*d*_6_) δ 202.4, 174.4, 161.3, 158.2, 138.1, 134.1, 132.4, 131.2, 128.8 (2C), 123.7, 123.4, 121.1, 113.8 (2C), 55.1, 45.6, 43.3; FTIR, v_max_: 3391, 2963, 1699, 1675, 1519, 1246 cm^−1^; HRMS (ESI TOF) *m*/*z* calc’d. for C_18_H_18_N_2_NaO_4_ [M + Na]^+^: 349.1159, found: 349.1164 (−1.5 ppm).

## 4. Conclusions

Thus, an efficient method has been developed for the synthesis of 3-(quinazolin-4-yl)propionic and 4-(quinazolin-4-yl)butyric acid derivatives, based on the cascade cyclization of 3-cyanoketones. During optimization of the reaction conditions, it was found that the most effective catalysts are moderately strong acids, such as oxalic acid or TsOH, as well as the mild Lewis acid Mg(ClO4)_2_, which has a high affinity for the carbonyl group. The use of more aggressive acidic reagents blocks the aniline amino group, preventing the target transformation. The proposed method demonstrated broad applicability to a variety of substrates with respect to substituents at the quinazoline core, the aryl substituent, and the 2-position of the heterocycle. A fundamental limitation of the method was highlighted, demonstrating the incompatibility with basic moieties in the starting compound. A reaction mechanism was proposed, and control experiments were conducted confirming the formation of 5-hydroxypyrrolidin-2-ones, which then undergo intramolecular cyclocondensation involving an amide or formamide group. The structures of the key compounds were confirmed by X-ray diffraction analysis. The presence of a carboxyl group in the resulting compounds provides opportunities for further functionalization via acylation reactions, including Friedel–Crafts, which makes the obtained compounds convenient building blocks for total synthesis.

## Figures and Tables

**Table 1:** Optimization of conditions for rearrangement of 3-cyanoketones to 3-(quinazolin-4-yl)propionic acids. Optimal conditions are marked in bold.

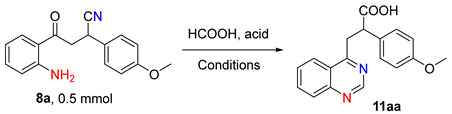					
№	HCOOH, mL	Acid, mmol	Conditions	Time	Yield, %
1	0.5	H_2_C_2_O_4_·H_2_O, 2.5	reflux	3 h	19
2	2	MsOH, 2	reflux	4 h	15
3	2	Mg(ClO_4_)_2_, 0.1	reflux	4 h	65
4	2	H_2_C_2_O_4_·H_2_O, 2.5	MW, 140 °C	30 min	73
5	0.5	H_2_C_2_O_4_·H_2_O, 2	MW, 140 °C	30 min	70
6	2	H_2_C_2_O_4_·H_2_O, 2	MW, 120 °C	1.5 h	64
7	2	H_2_C_2_O_4_·H_2_O, 2	MW, 160 °C	15 min	61
8	2	**TsOH, 0** **.5**	**MW, 140 °C**	**30 min**	**78**
9	0.5	TsOH, 0.5	MW, 140 °C	30 min	55
10	2	CCl_3_COOH, 0.5	MW, 140 °C	30 min	64
11	2	CF_3_COOH, 0.5	MW, 140 °C	30 min	72
12	2	BF_3_·Et_2_O (0.1 mL)	MW, 140 °C	30 min	58
13	2	-	MW, 140 °C	1 h	57

## Data Availability

The original contributions presented in this study are included in the article. Further inquiries can be directed to the corresponding author.

## Supplementary Materials

The following supporting information can be downloaded at: https://www.mdpi.com/article/10.3390/ijms27093903/s1.

## Abbreviations

The following abbreviations are used in this manuscript:

MW	Microwave activation
TsOH	Toluene sulphonic acid
DMF	Dimentylformamide
IPA	Isopropyl alcohol
