# Supplementary material for: Synthesis of 3-(Quinazolin-4-yl)propionic Acids via an Acid-Catalyzed Rearrangement of 4-Oxobutyronitriles
Source: Int J Mol Sci. 2026 Apr 28;27(9):3903. doi: 10.3390/ijms27093903 (PMC13163398; doi:10.3390/ijms27093903)
Supplement: Supplementary file 1 [file ijms-27-03903-s001.zip › ijms-4230029-supplementary.pdf]

Electronic Supplementary Material (ESI)

Synthesis Of 3-(Quinazolin-4-yl)propionic Acids Via An Ac-id-Catalyzed Rearrangement Of 4-Oxobutyronitrirles

Nicolai A. Aksenov *, Alexander E. Kurlikov , Alexander P. Barbolin , Polina S. Karaseva , Milena M. Baziyants, Elizabeth A. Glotova , Igor A. Kurenkov , Dmitrii A. Aksenov and Alexander V. Aksenov

Department of Organic Chemistry, North Caucasus Federal University, 1a Pushkin St., Stavropol 355017, Russian Federation

Supporting Information

^{1}H and ^{13}C NMR spectral charts for 2'-aminochalcones **19**……………………………………………………………………..………..S2
^{1}H and ^{13}C NMR spectral charts for 2-aryl-4-(2-aminophenyl)-4-oxobutironitriles **8**, **9**……………………………..………………..S7
^{1}H and ^{13}C NMR spectral charts for Michael adducts **21, 24**………………………………………………………………………….S29
^{1}H and ^{13}C NMR spectral charts for 3-(quinazolin-4-yl)propanoic acids **11**……………………………………………………….……..S41
^{1}H and ^{13}C NMR spectral charts for 4-(quinazolin-4-yl)butanoic acids **23**……………………………………………………………..S73
^{1}H and ^{13}C NMR spectral charts for *N*-(2-(3-Cyano-3-(4-methoxyphenyl)propanoyl)phenyl)formamide **9aa** ……………………………..S83
^{1}H and ^{13}C NMR spectral charts for 4-(2-Formamidophenyl)-2-(4-methoxyphenyl)-4-oxobutanamide **16aa**..……………………………..S85
^{1}H and ^{13}C NMR spectral charts for for mixture of **11ba** and **11aa**……………………………………...……………………………..S87
^{1}H and ^{13}C NMR spectral charts for for mixture of **11ca** and **11aa**……………………………...……………………………..S89
^{1}H and ^{13}C NMR spectral charts for for mixture of **11da** and **11aa**……………………………...……………………………..S91
HRMS spectral chart for reaction mixture of reaction of **9da** in presence of HCOOH at 110 °C…………………………………………S93
Xray diffraction data for **11ae** ……………………………………………………………………………………………..S94
Xray diffraction data for **11ca** ……………………………………………………………………………………………..S103
Xray diffraction data for **23al** ……………………………………………………………………………………………...S112

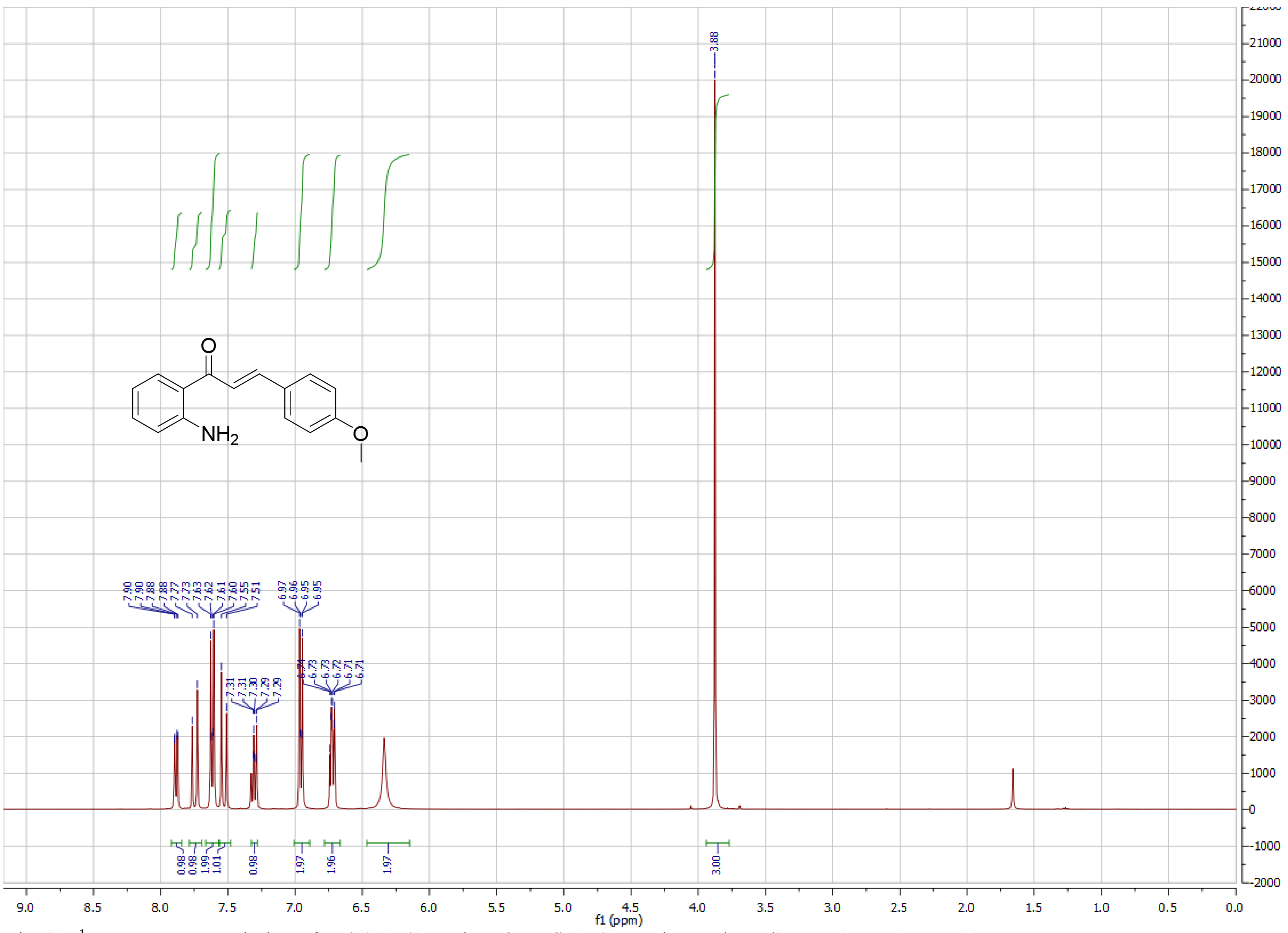

Fig S1. ^{1}H NMR spectral chart for (E)-1-(2-aminophenyl)-3-(4-methoxyphenyl)prop-2-en-1 -one **19a**

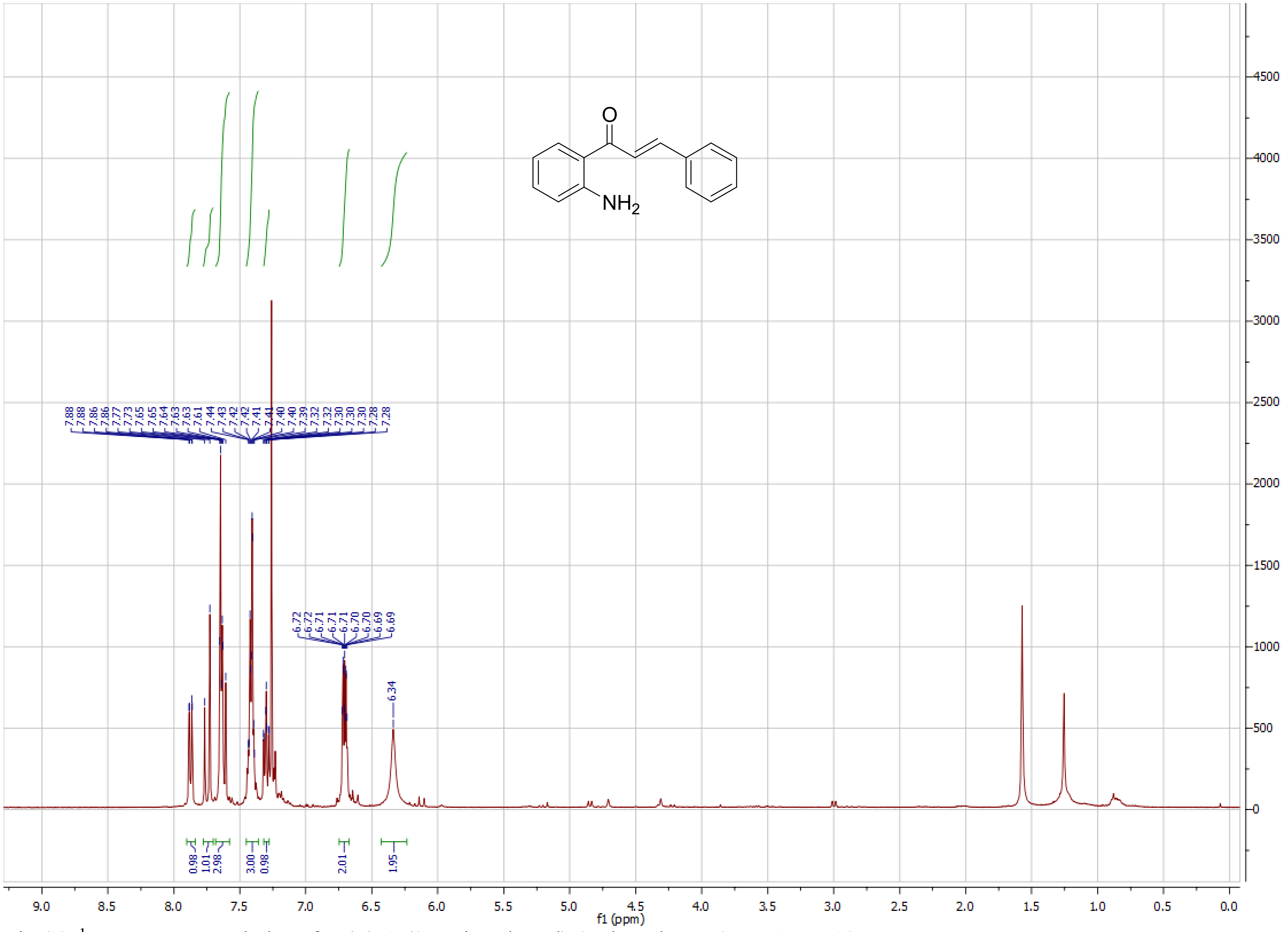

Fig S2. ^{1}H NMR spectral chart for (E)-1-(2-aminophenyl)-3-phenylprop-2-en-1-one **19b**

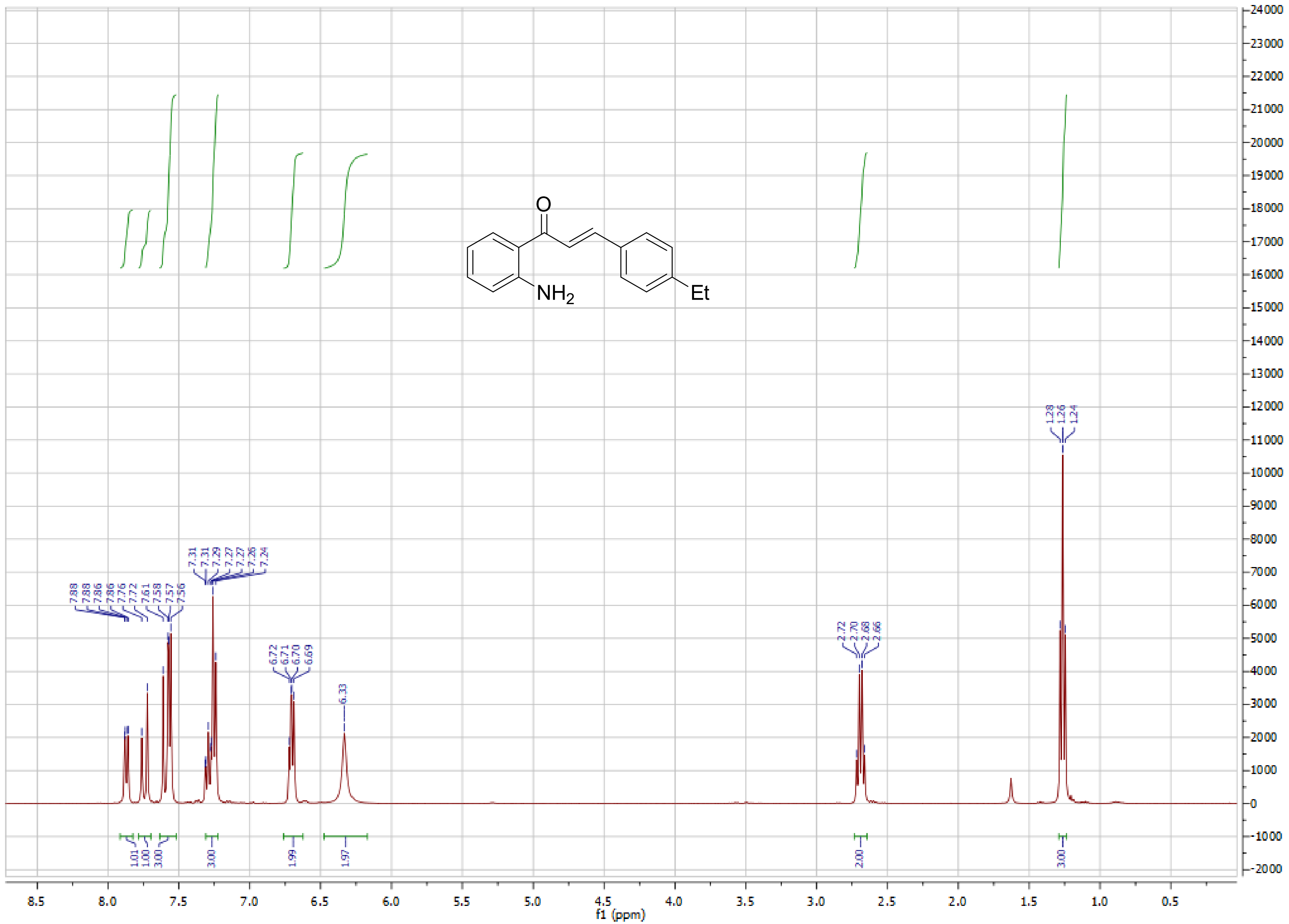

Fig S3. ^{1}H NMR spectral chart for (E)-1-(2-aminophenyl)-3-(4-ethylphenyl)prop-2-en-1-one **19e**

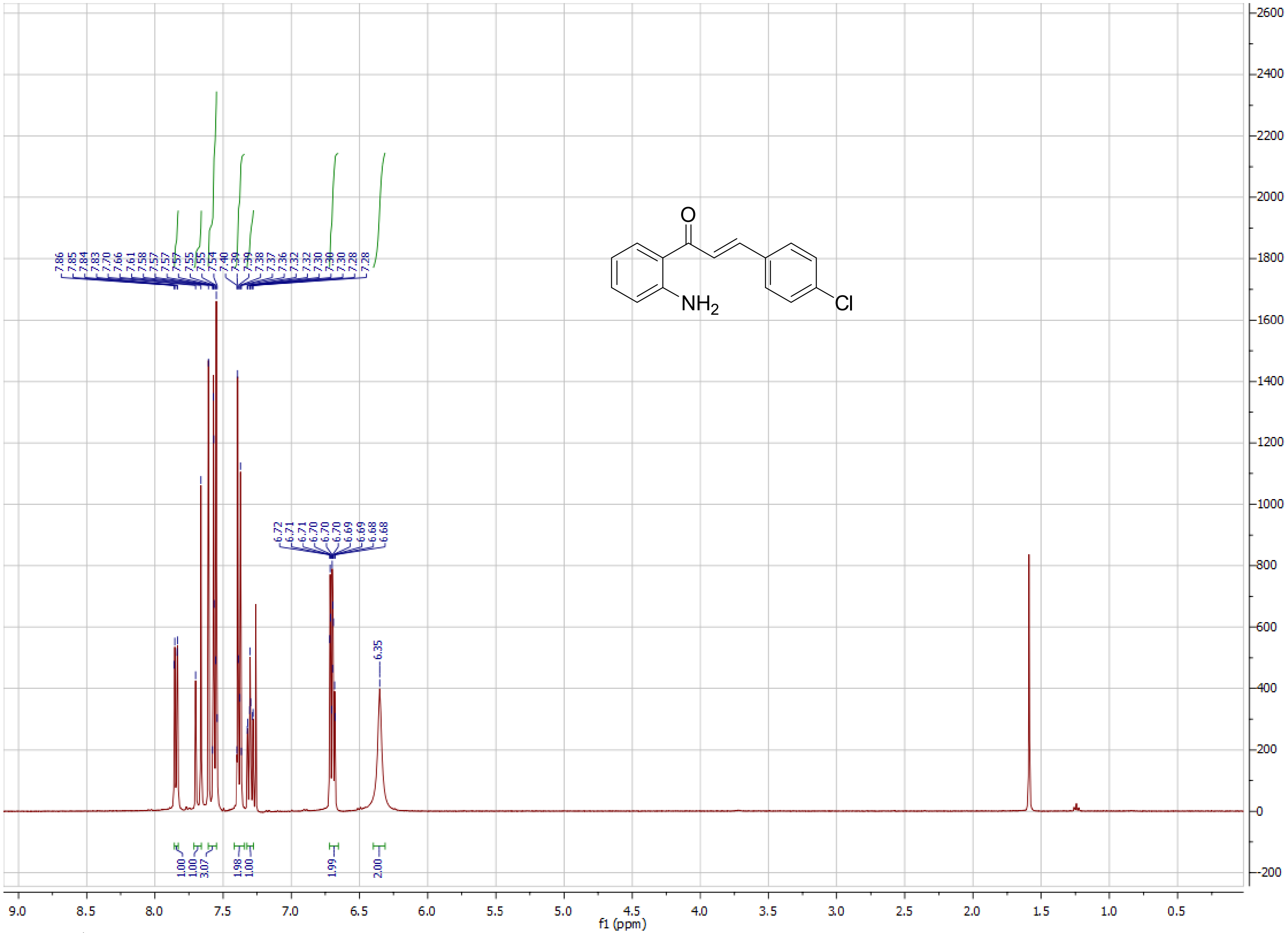

Fig S4. ^{1}H NMR spectral chart for (E)-1-(2-aminophenyl)-3-(4-chloropheny l)prop-2-en-1-one **19h**

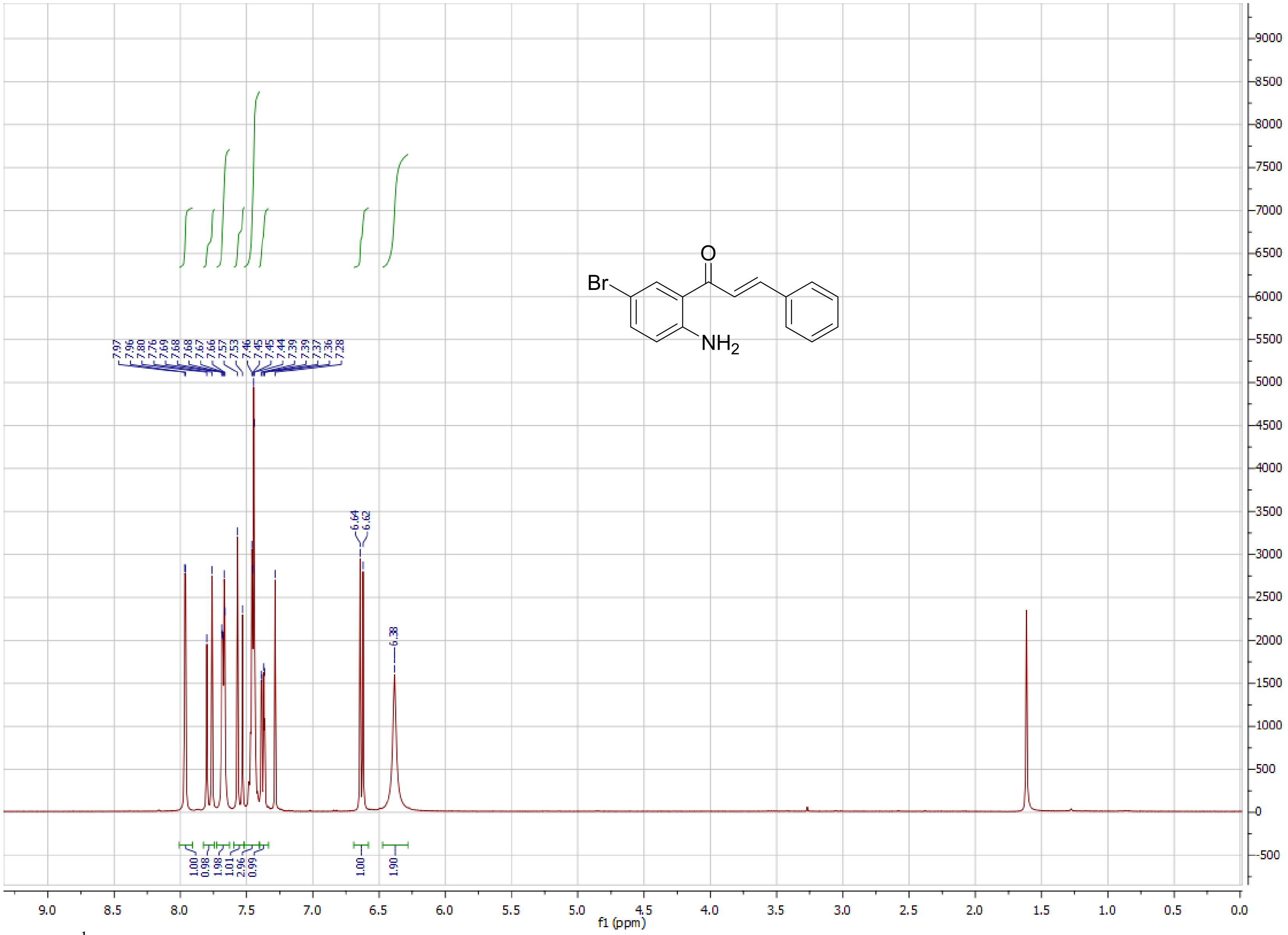

Fig S5. ^{1}H NMR spectral chart for (E)-1-(2-amino-5-bromophenyl)-3-phenyl prop-2-en-1-one **19l**

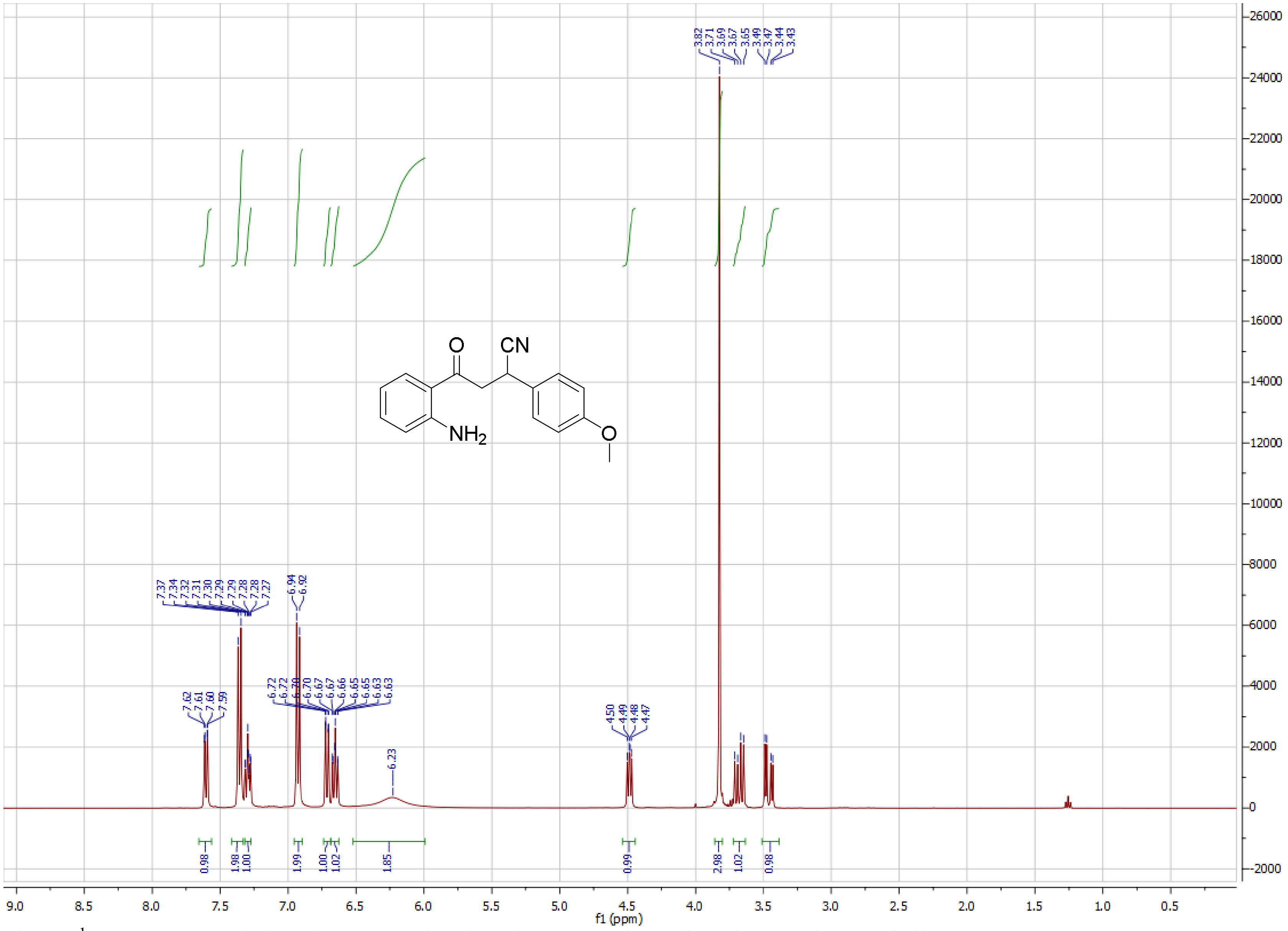

Fig S6. ^{1}H NMR spectral chart for 4-(2-aminophenyl)-2-(4-methoxyphenyl)-4-oxobutanenitrile **13a**

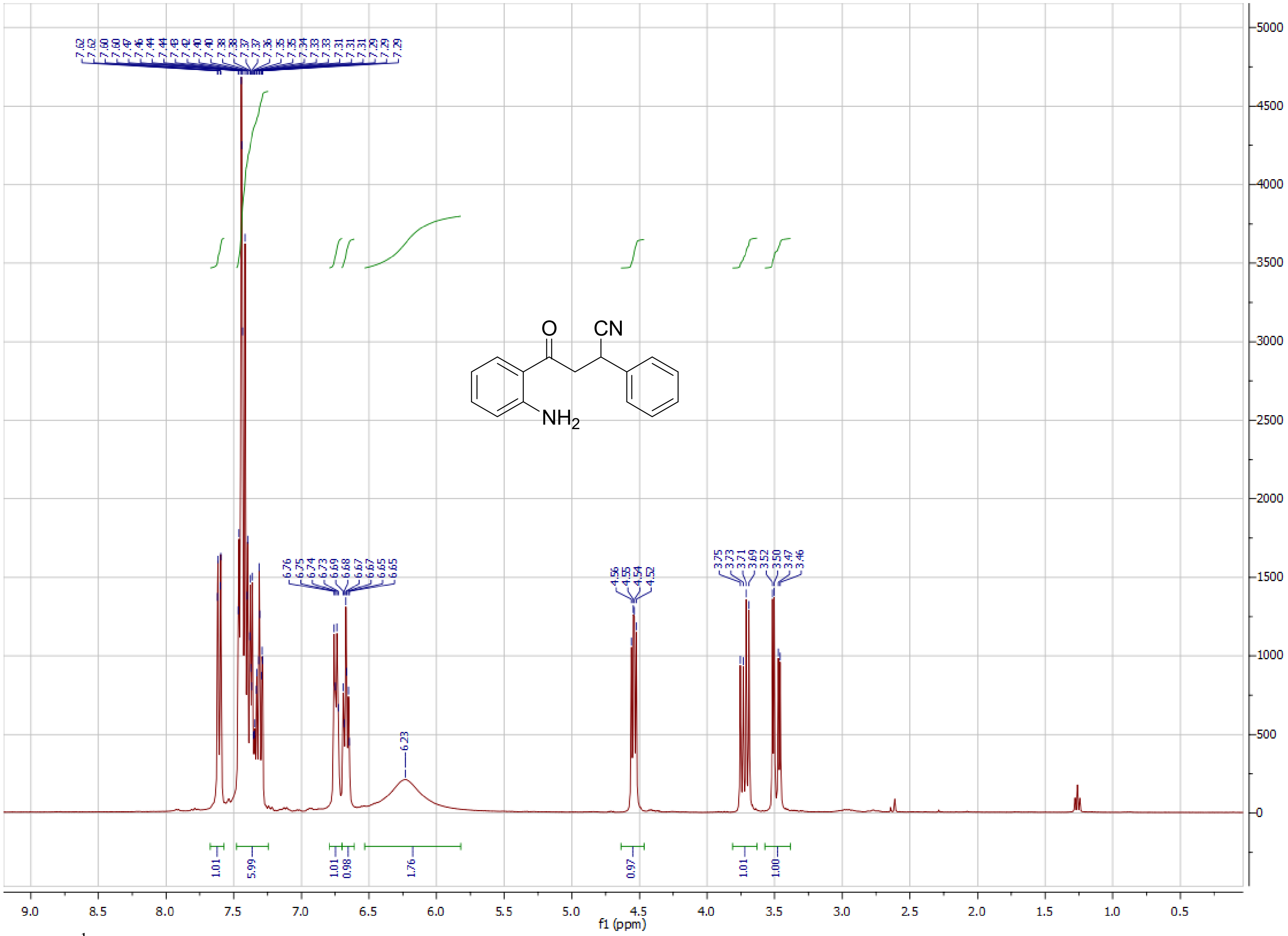

Fig S7. ^{1}H NMR spectral chart for 4-(2-aminophenyl)-4-oxo-2-phenylbutanenitrile **13b**

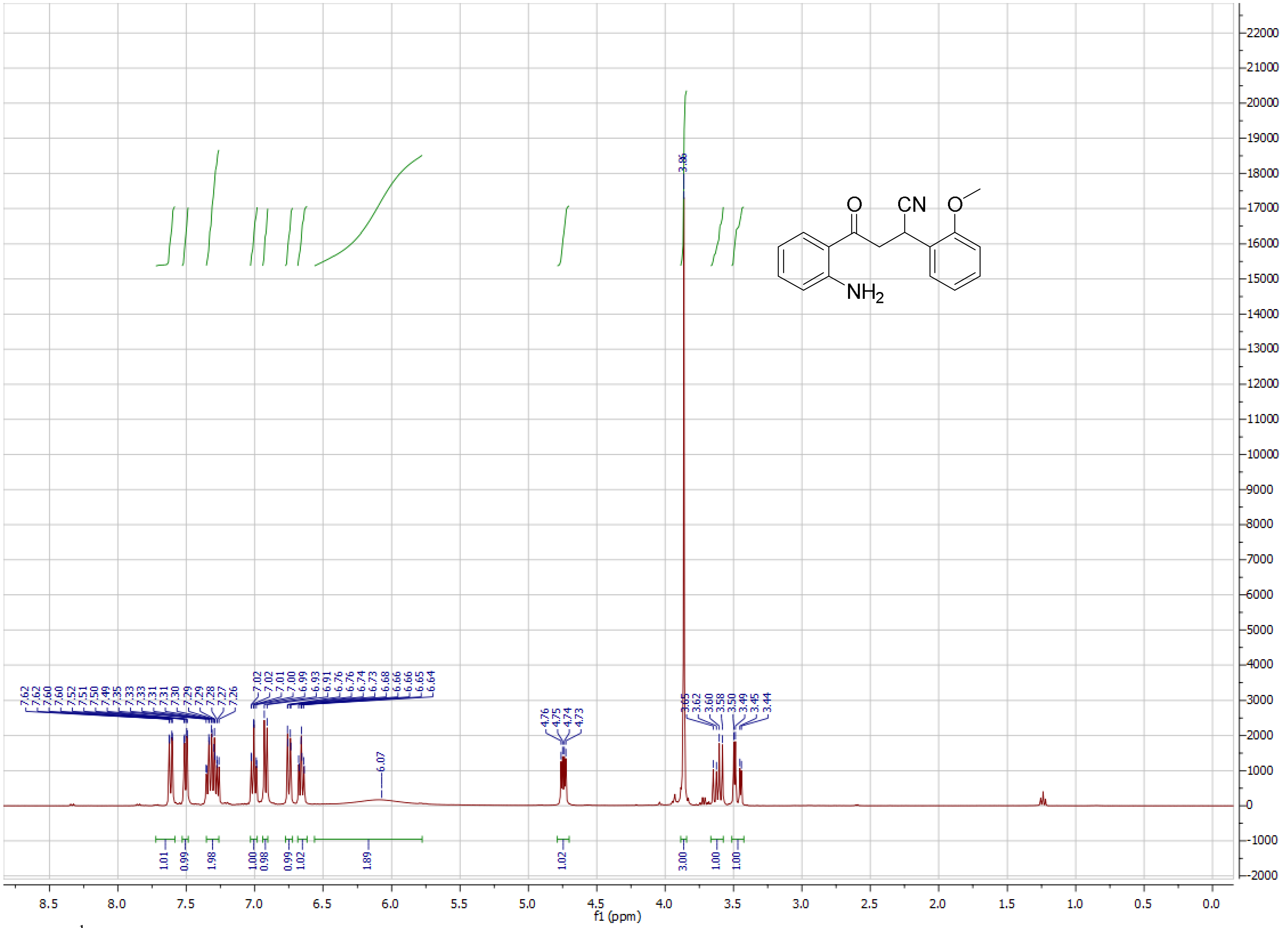

Fig S8. ^{1}H NMR spectral chart for 4-(2-aminophenyl)-2-(2-methoxyphenyl)-4-oxobutanenitrile **13c**

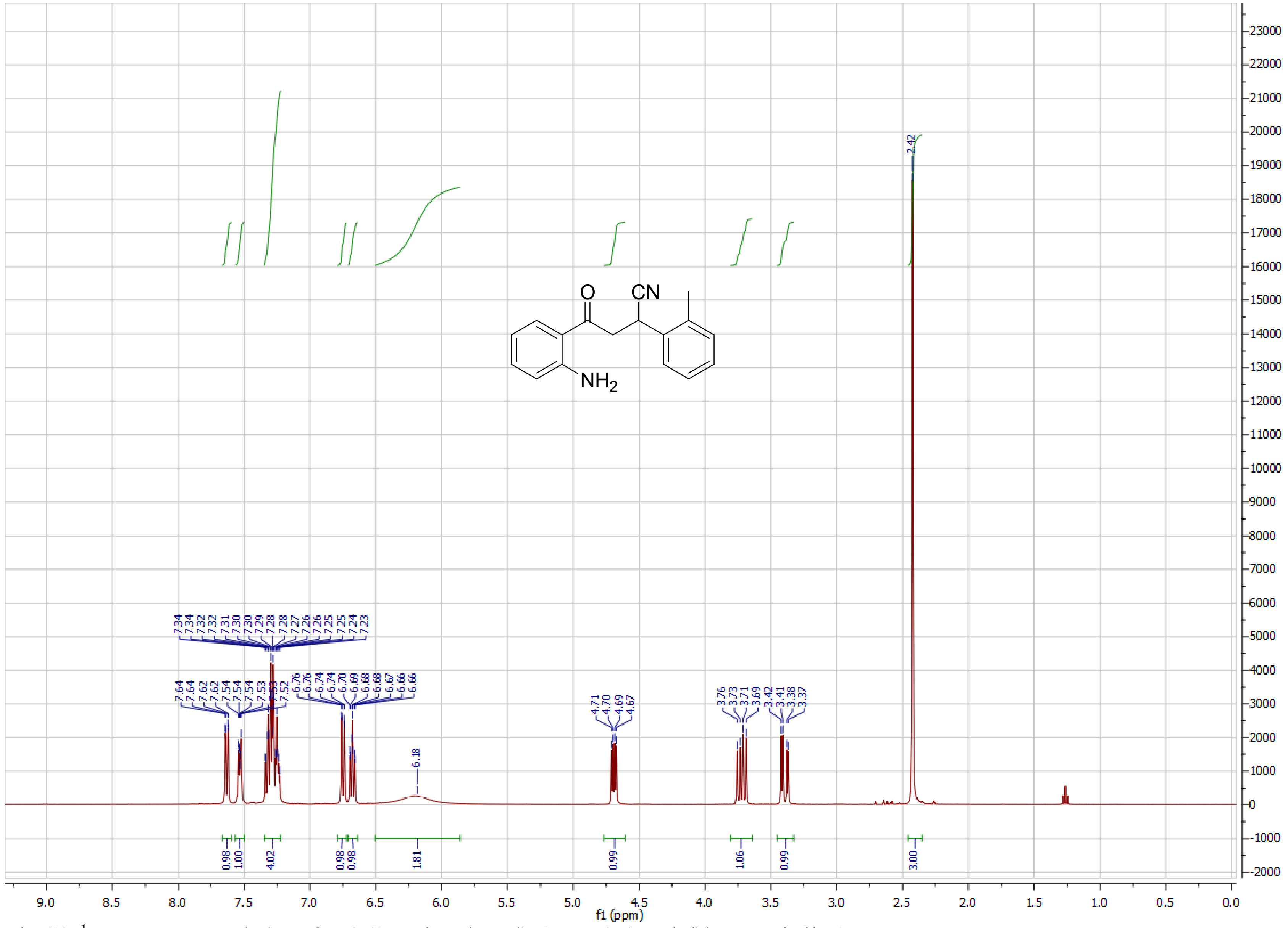

Fig S9. ^{1}H NMR spectral chart for 4-(2-aminophenyl)-4-oxo-2-(o-tolyl)butanenitrile **8d**

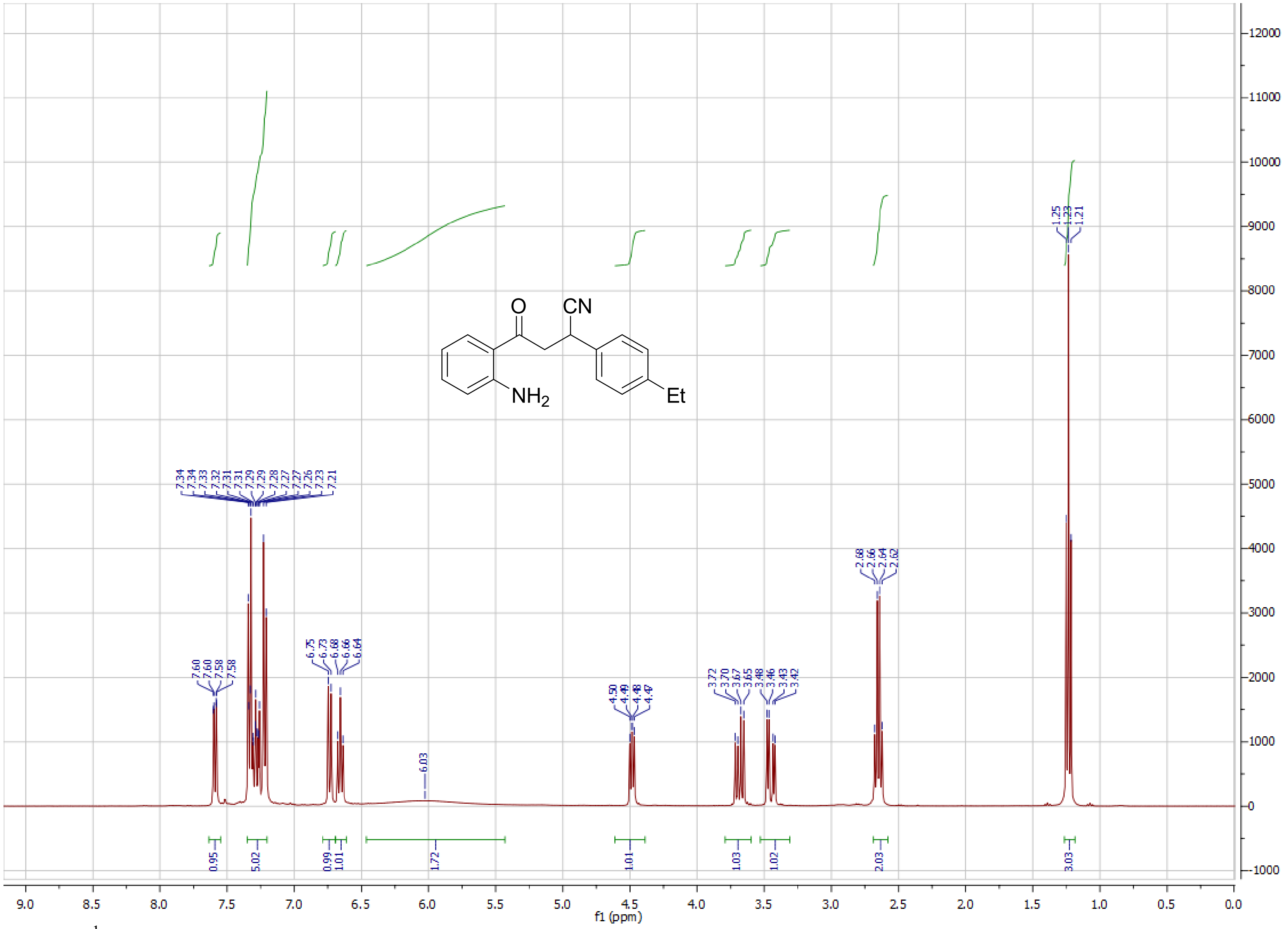

Fig S10. ^{1}H NMR spectral chart for 4-(2-aminophenyl)-2-(4-ethylphenyl)-4-oxobutanenitrile **8e**

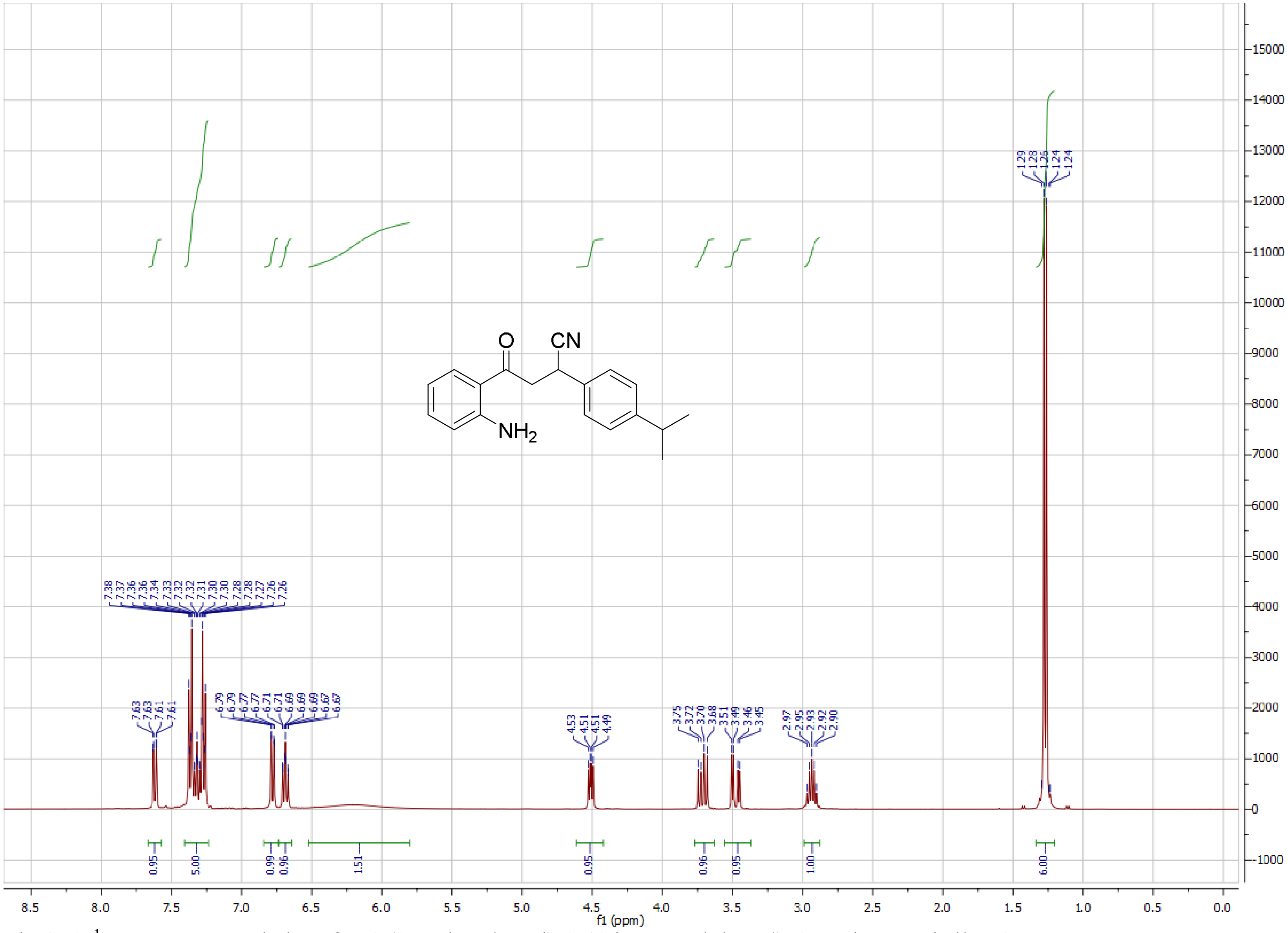

Fig S11. ^{1}H NMR spectral chart for 4-(2-aminophenyl)-2-(4-isopropylphenyl)-4-oxobutanenitrile **8f**

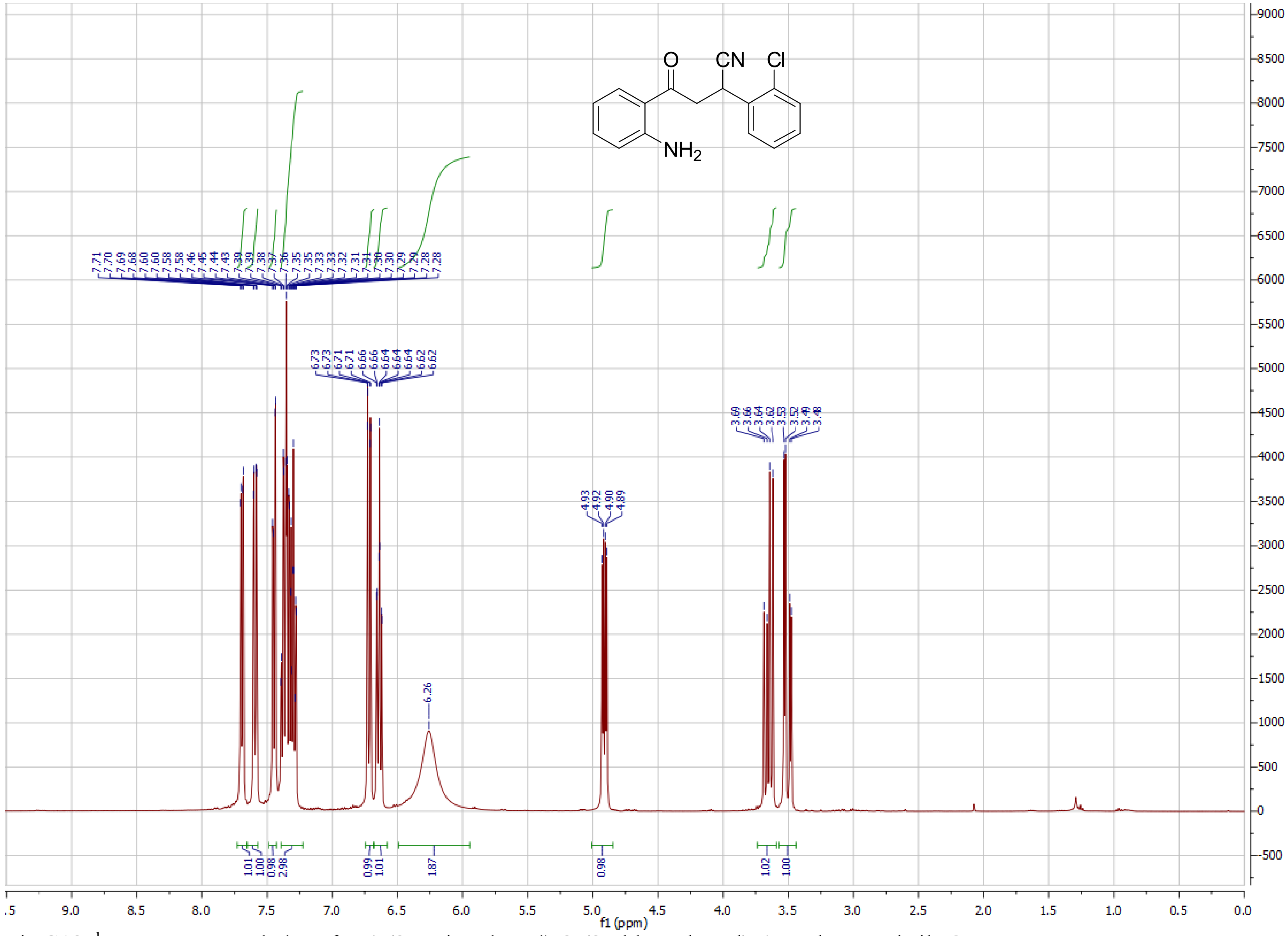

Fig S12. ^{1}H NMR spectral chart for 4-(2-aminophenyl)-2-(2-chlorophenyl)-4-oxobutanenitrile **8g**

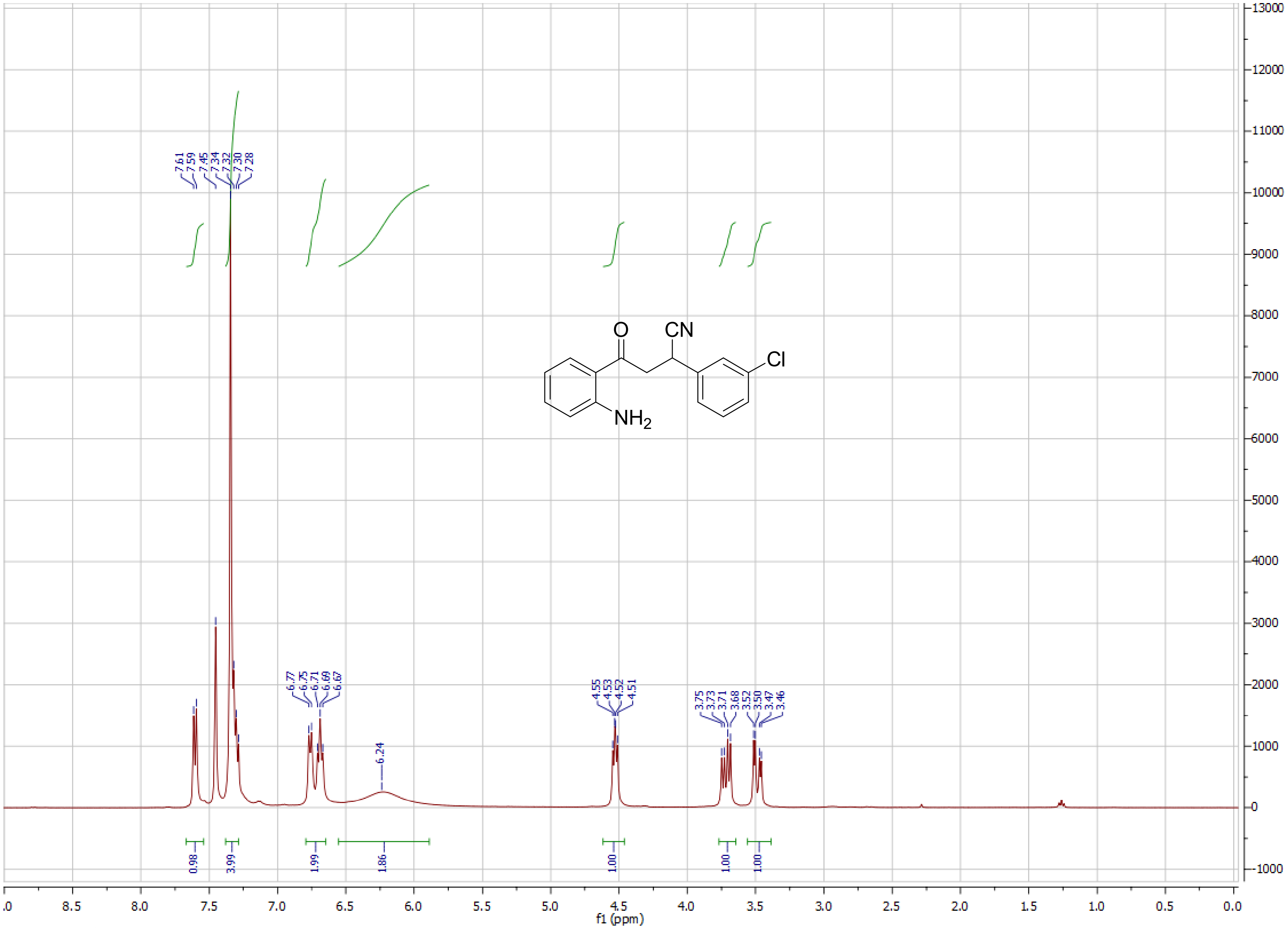

Fig S13. ^{1}H NMR spectral chart for 4-(2-aminophenyl)-2-(3-chlorophenyl)-4-oxobutanenitrile **8h**

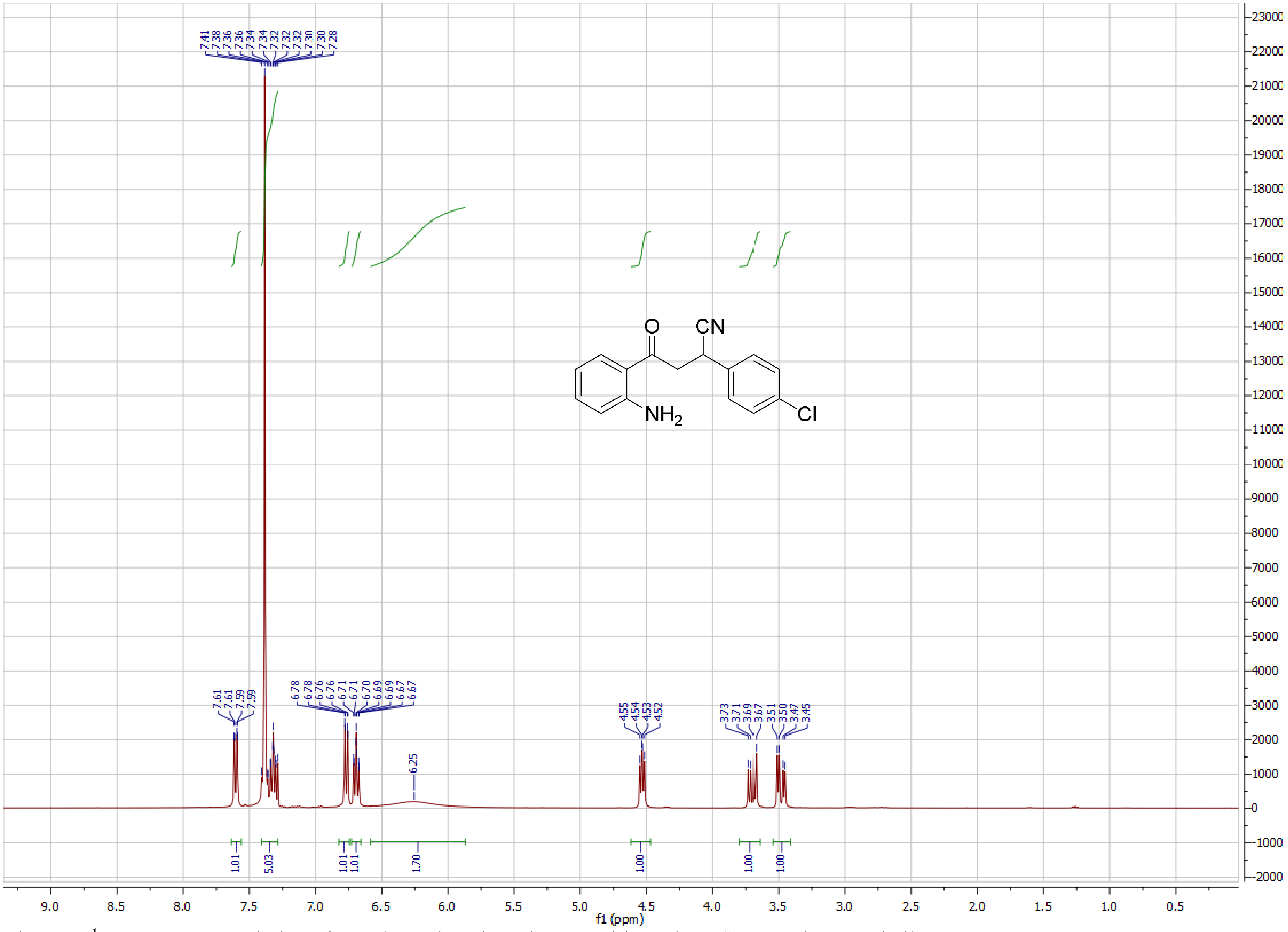

Fig S14. ^{1}H NMR spectral chart for 4-(2-aminophenyl)-2-(4-chlorophenyl)-4-oxobutanenitrile **8i**

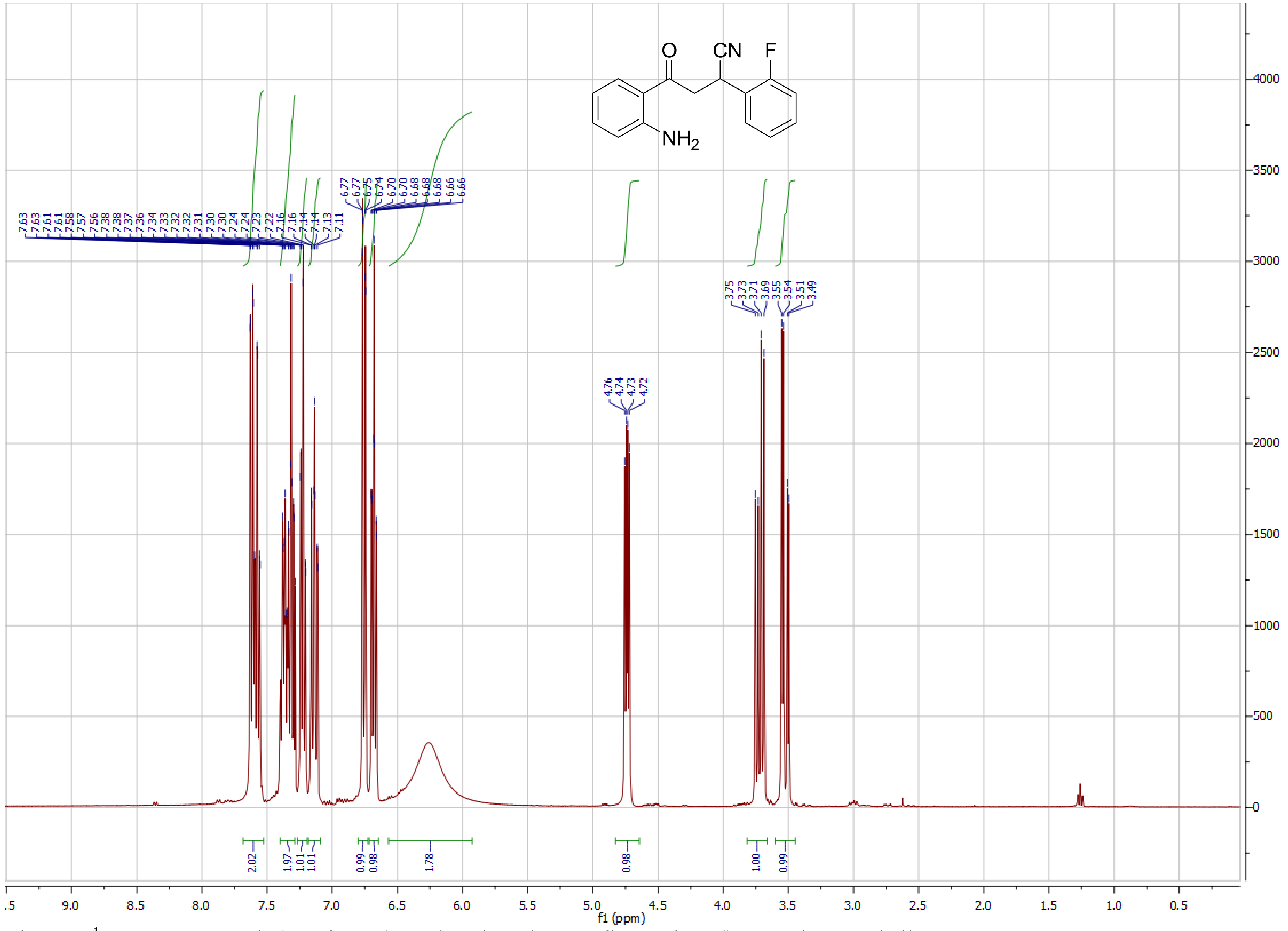

Fig S15. ^{1}H NMR spectral chart for 4-(2-aminophenyl)-2-(2-fluorophenyl)-4-oxobutanenitrile **8j**

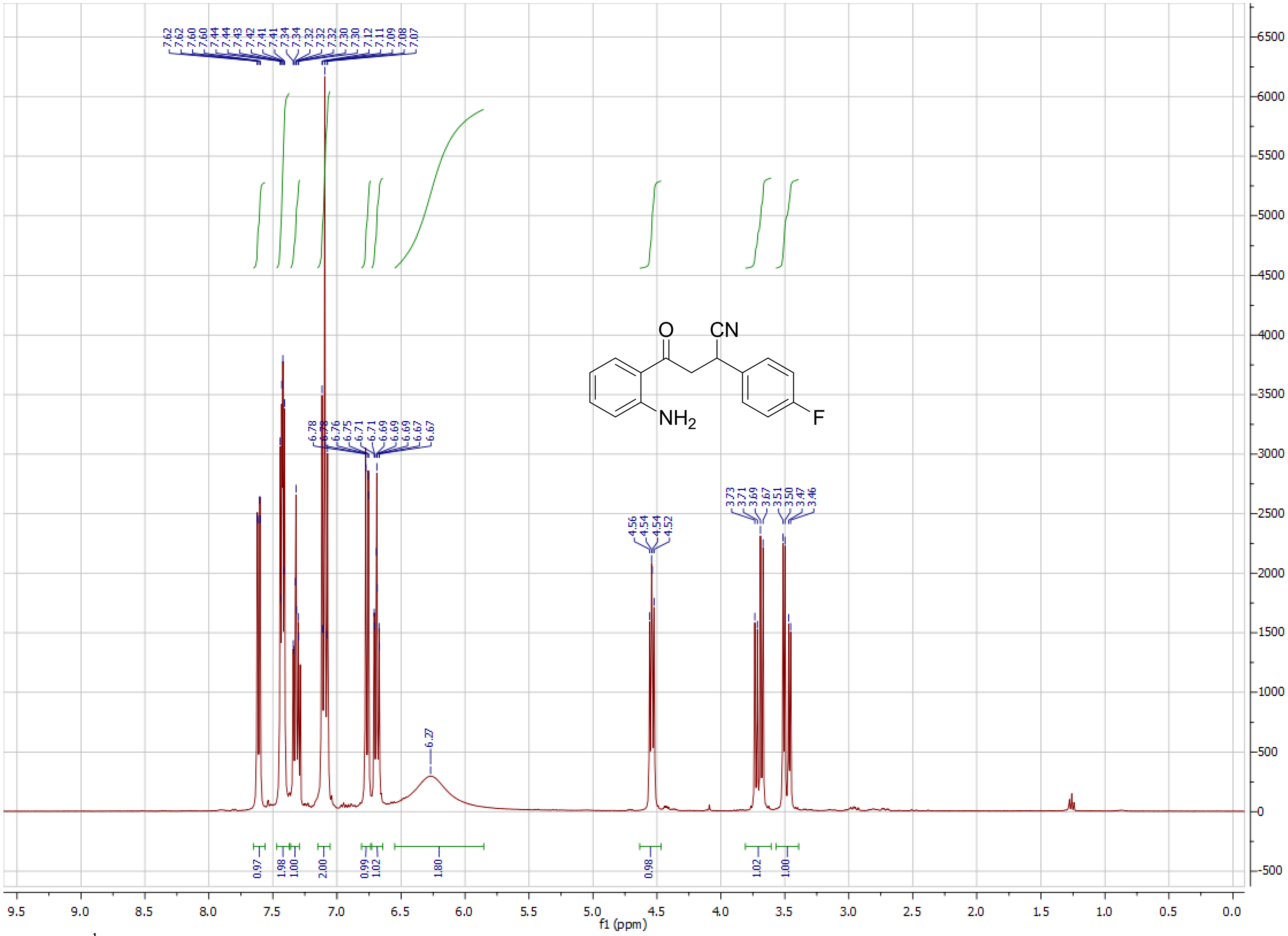

Fig S16. ^{1}H NMR spectral chart for 4-(2-aminophenyl)-2-(4-fluorophenyl)-4-oxobutanenitrile **8k**

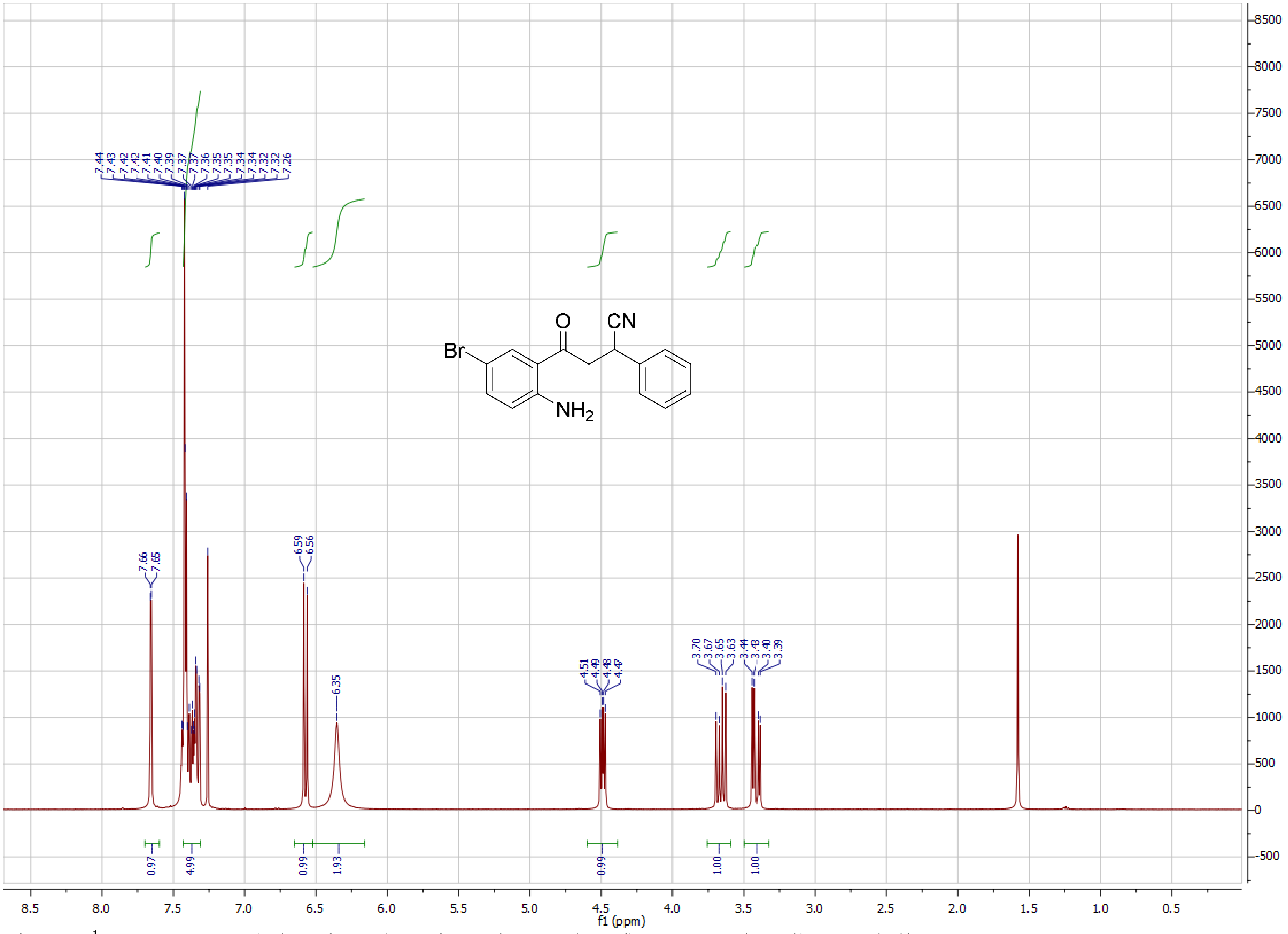

Fig S17. ^{1}H NMR spectral chart for 4-(2-amino-5-bromophenyl)-4-oxo-2-phenylbutanenitrile **8l**

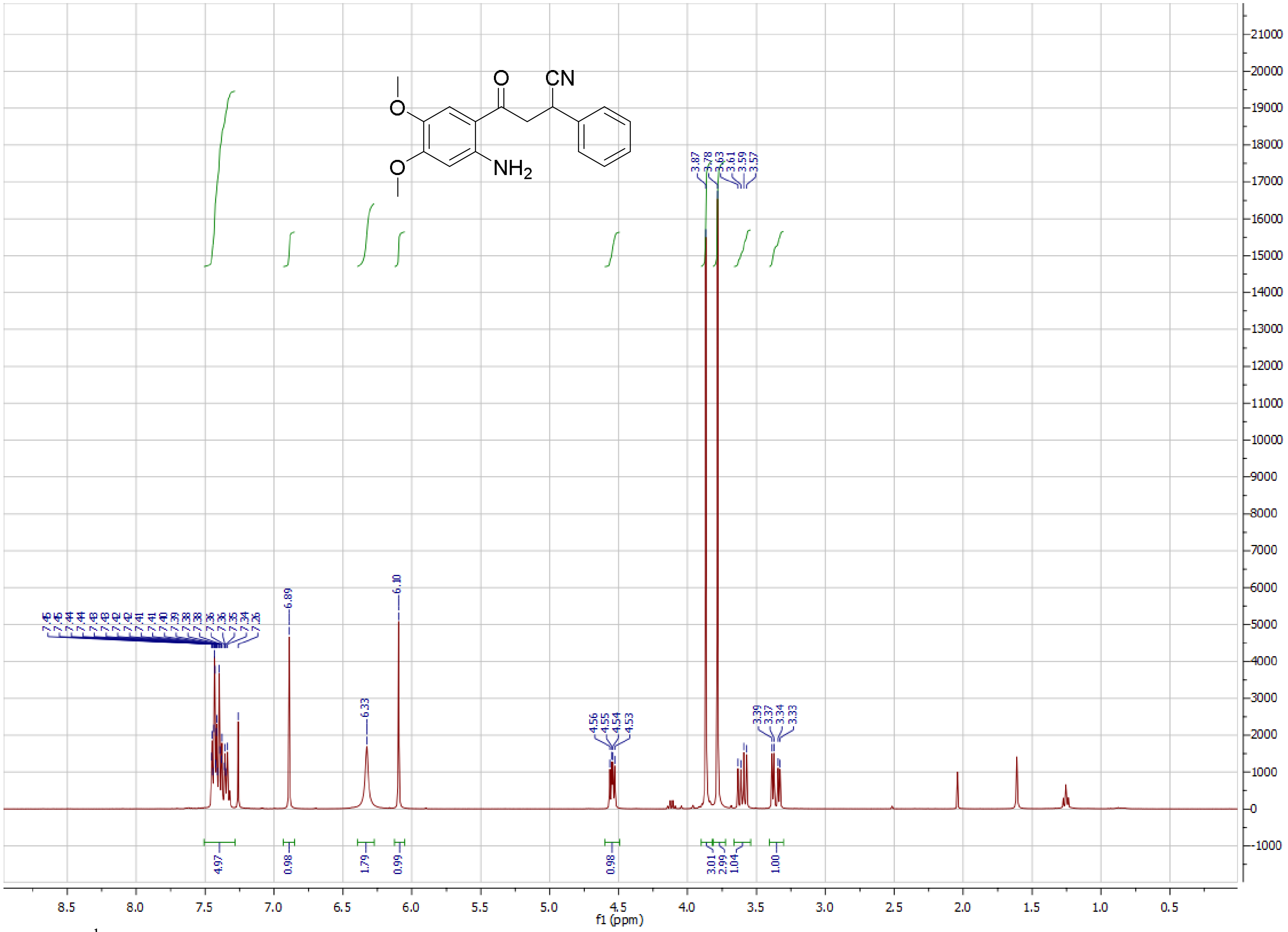

Fig S18. ^{1}H NMR spectral chart for 4-(2-amino-4,5-dimethoxyphenyl)-4-oxo-2-phenylbutanenitrile **8m**

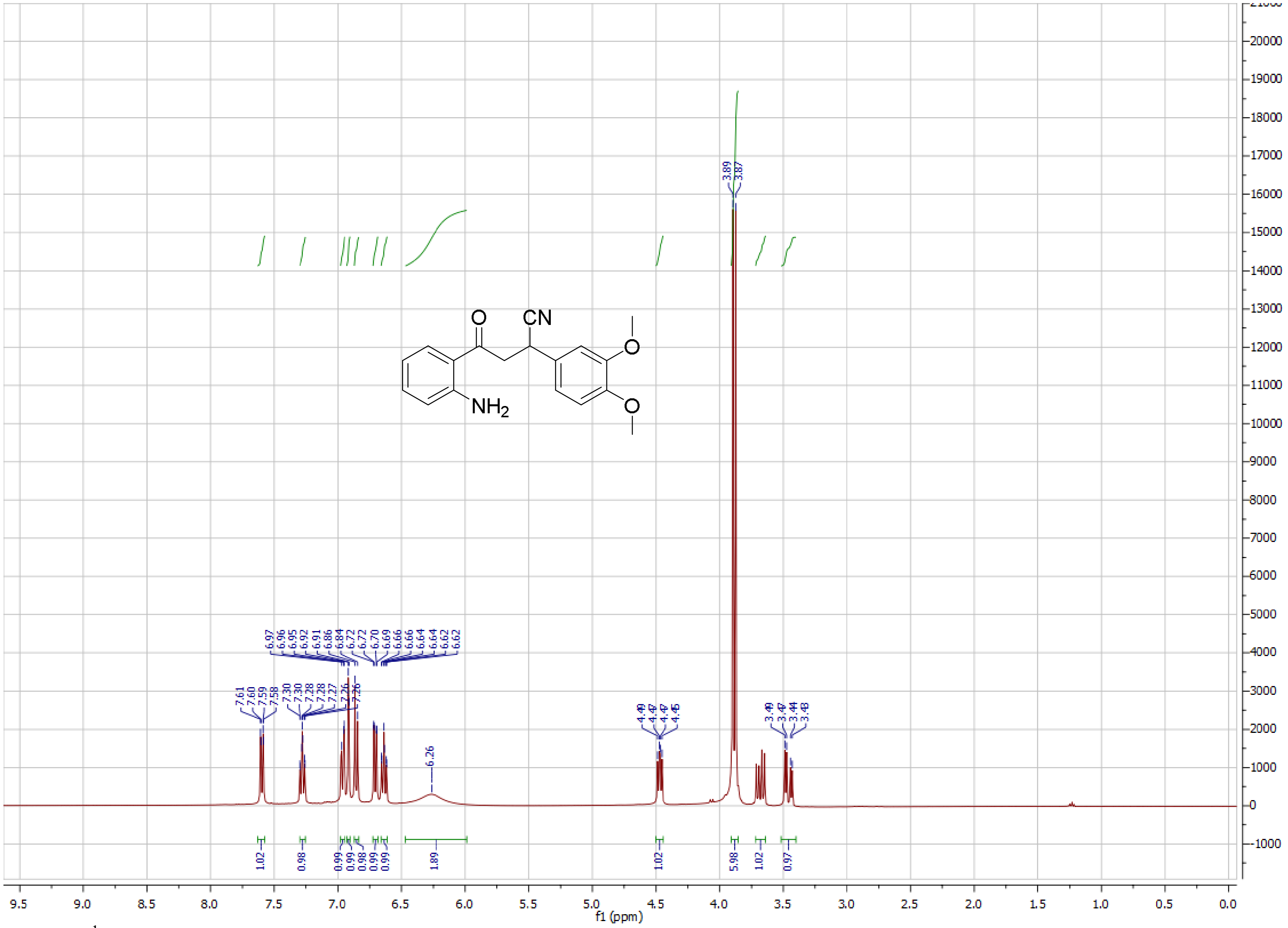

Fig S19. ^{1}H NMR spectral chart for 4-(2-aminophenyl)-2-(3,4-dimethoxyphenyl)-4-oxobutanenitrile **8n**

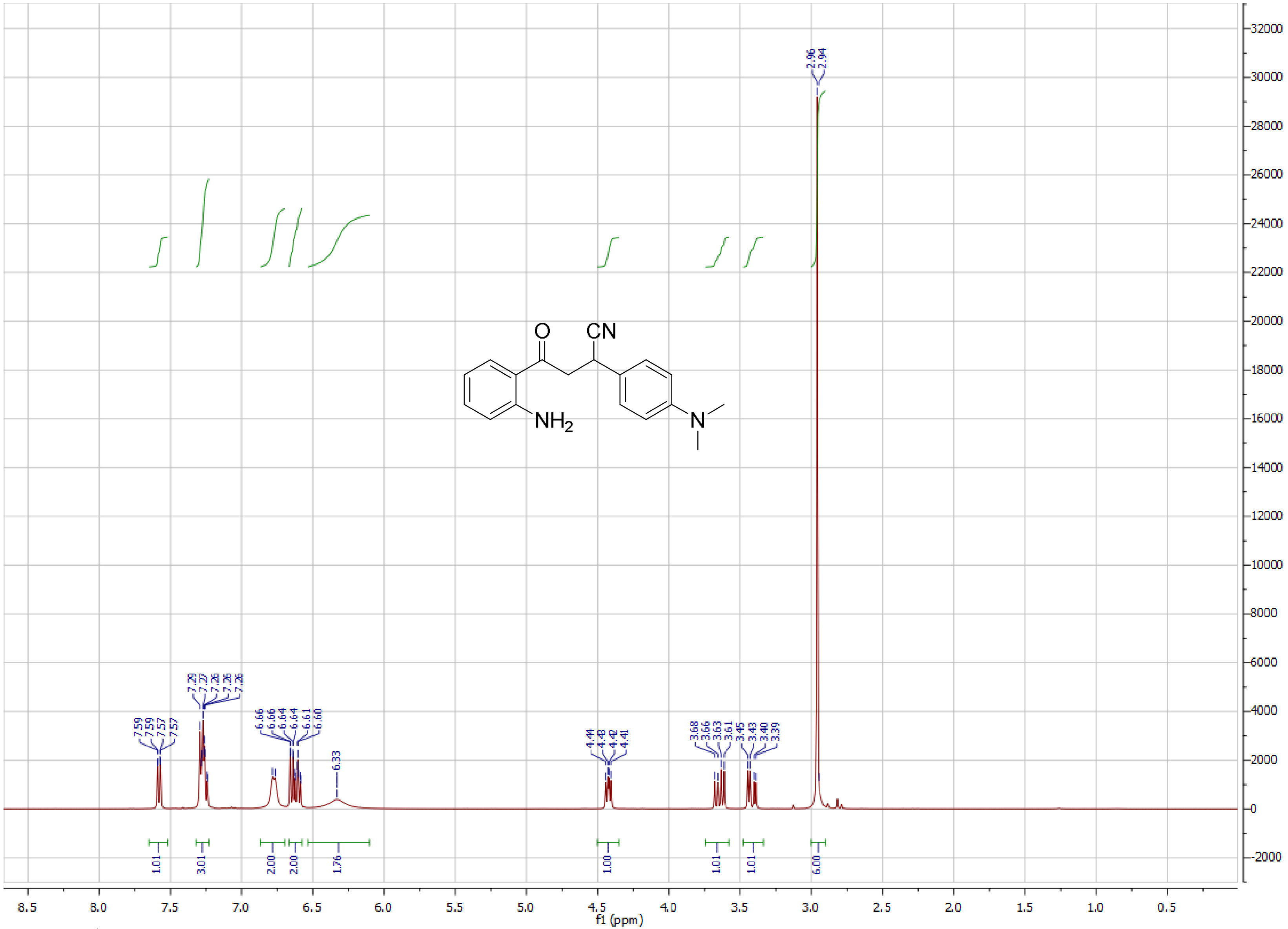

Fig S20. ^{1}H NMR spectral chart for 4-(2-aminophenyl)-2-(4-(dimethylamino)phenyl)-4-oxobutanenitrile **8o**

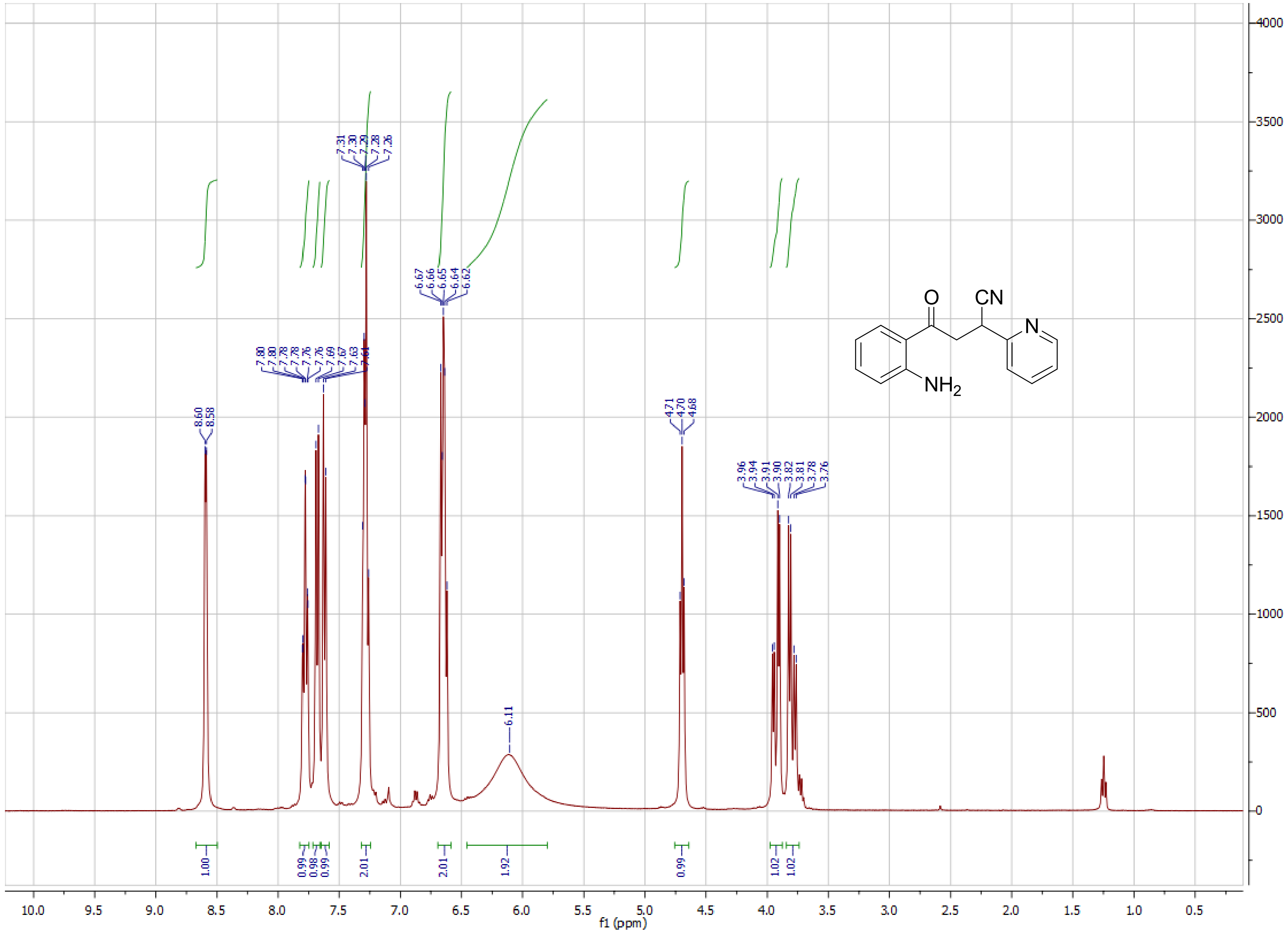

Fig S21. ^{1}H NMR spectral chart for 4-(2-aminophenyl)-4-oxo-2-(pyridin-2-yl)butanenitr ile **8p**

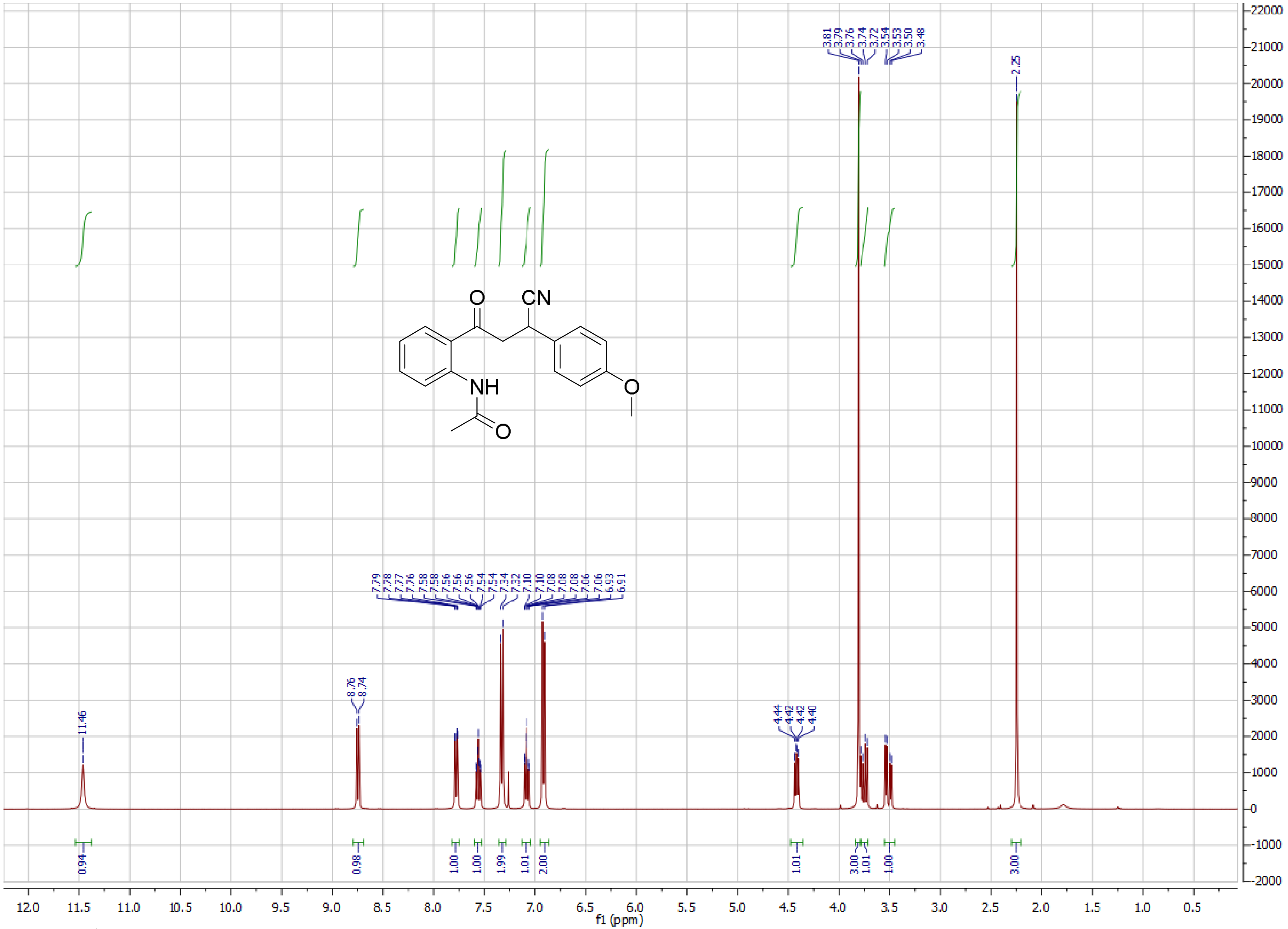

Fig S22. ^{1}H NMR spectral chart for *N*-(2-(3-cyano-3-(4-methoxyphenyl)propanoyl)phenyl)acetamide **9ba**

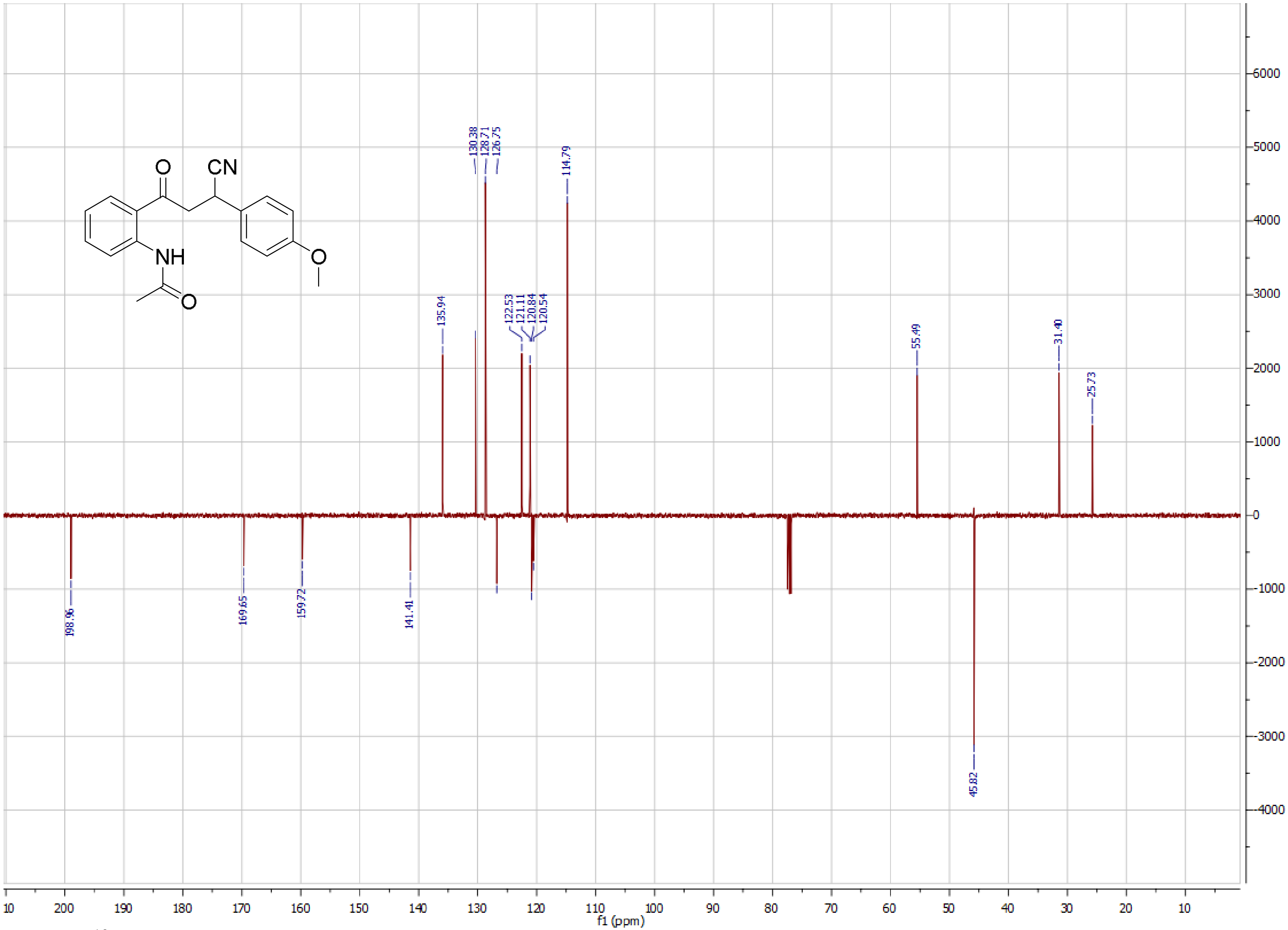

Fig S23. ^{13}C NMR spectral chart for *N*-(2-(3-cyano-3-(4-methoxyphenyl)propanoyl)phenyl)acetamide **9ba**

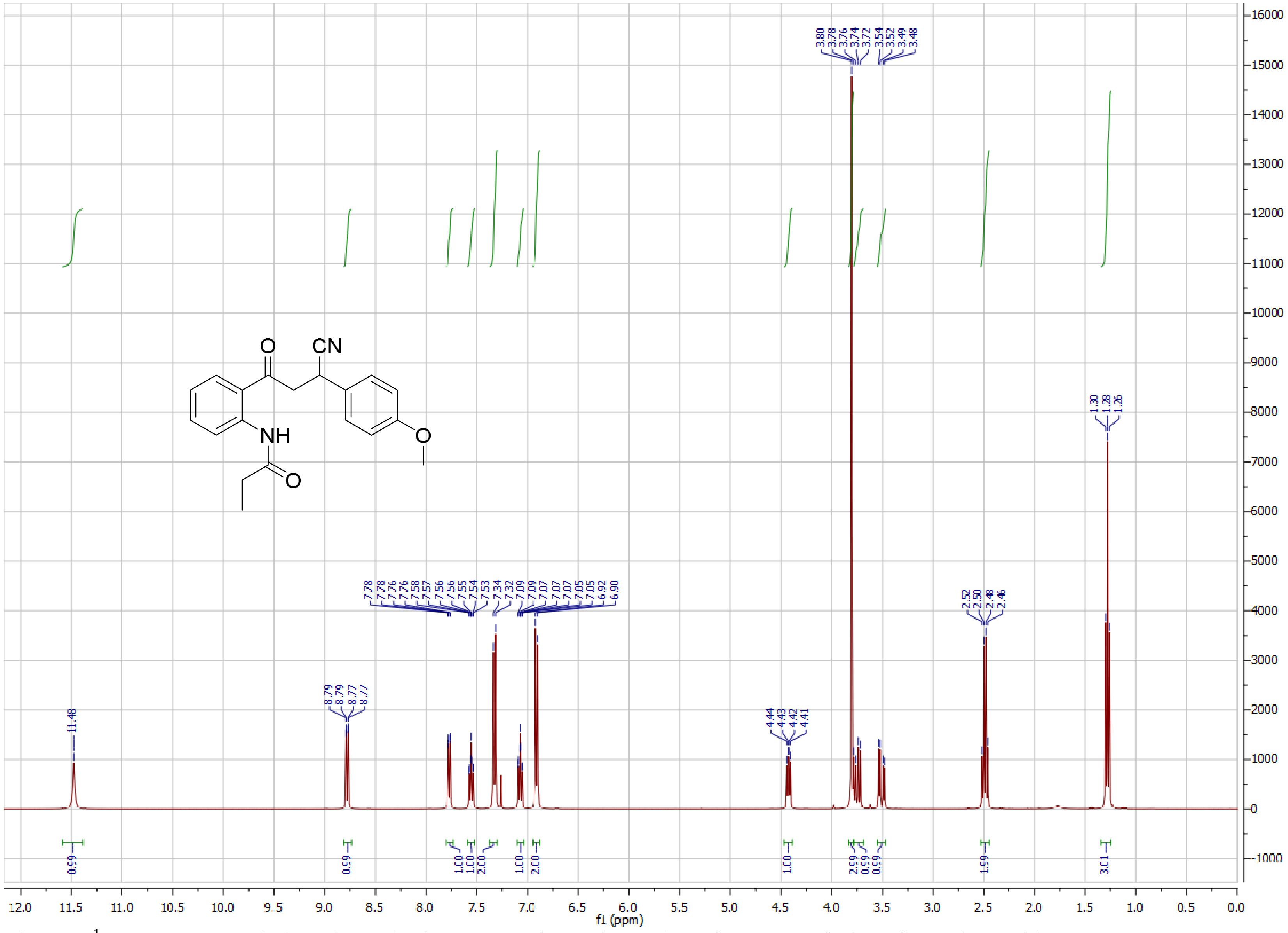

Fig S24. ^{1}H NMR spectral chart for *N*-(2-(3-cyano-3-(4-methoxyphenyl)propanoyl)phenyl)propionamide **9ca**

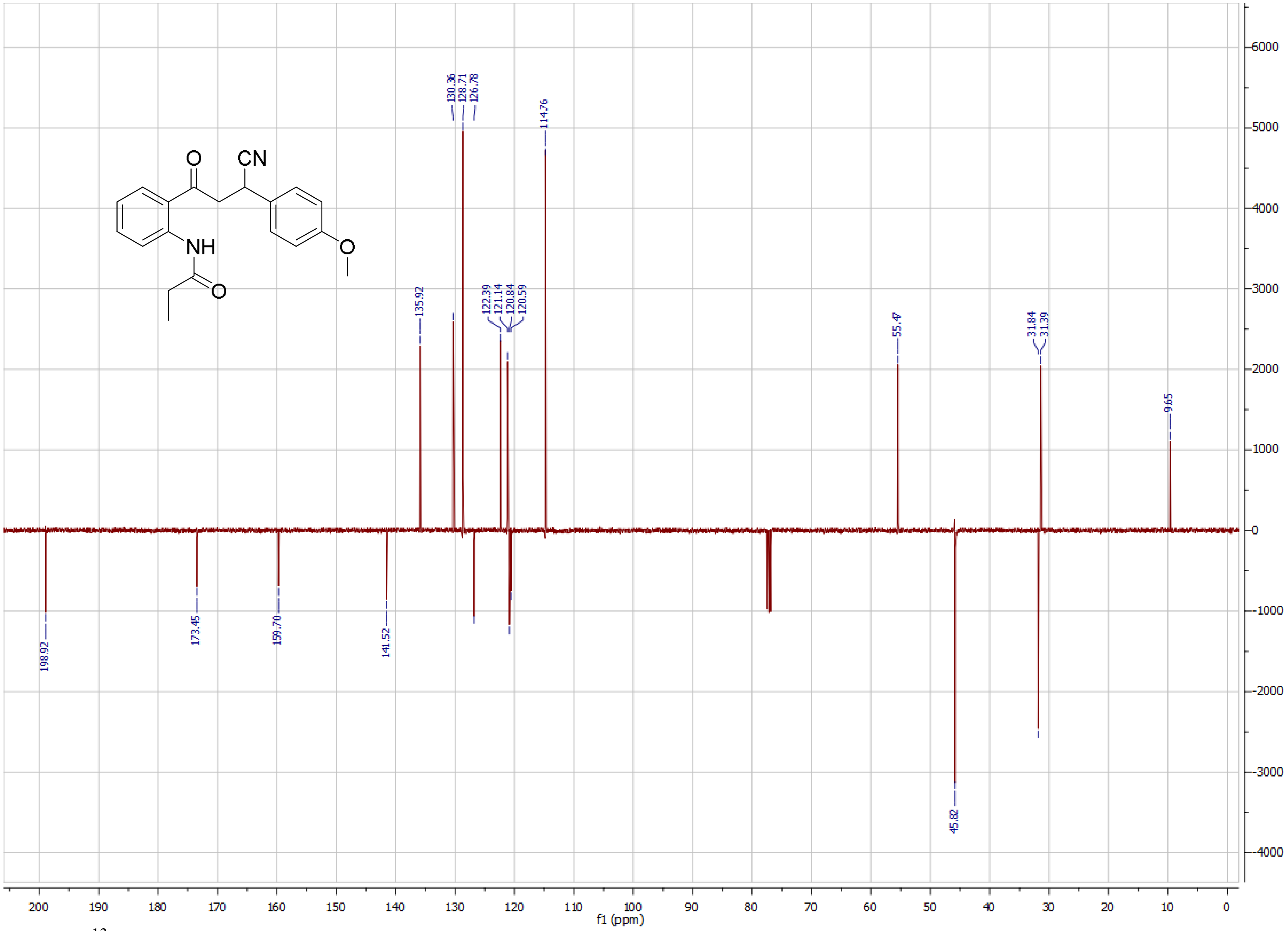

Fig S25. ^{13}C NMR spectral chart for *N*-(2-(3-cyano-3-(4-methoxyphenyl)propanoyl)phenyl)propionamide **9ca**

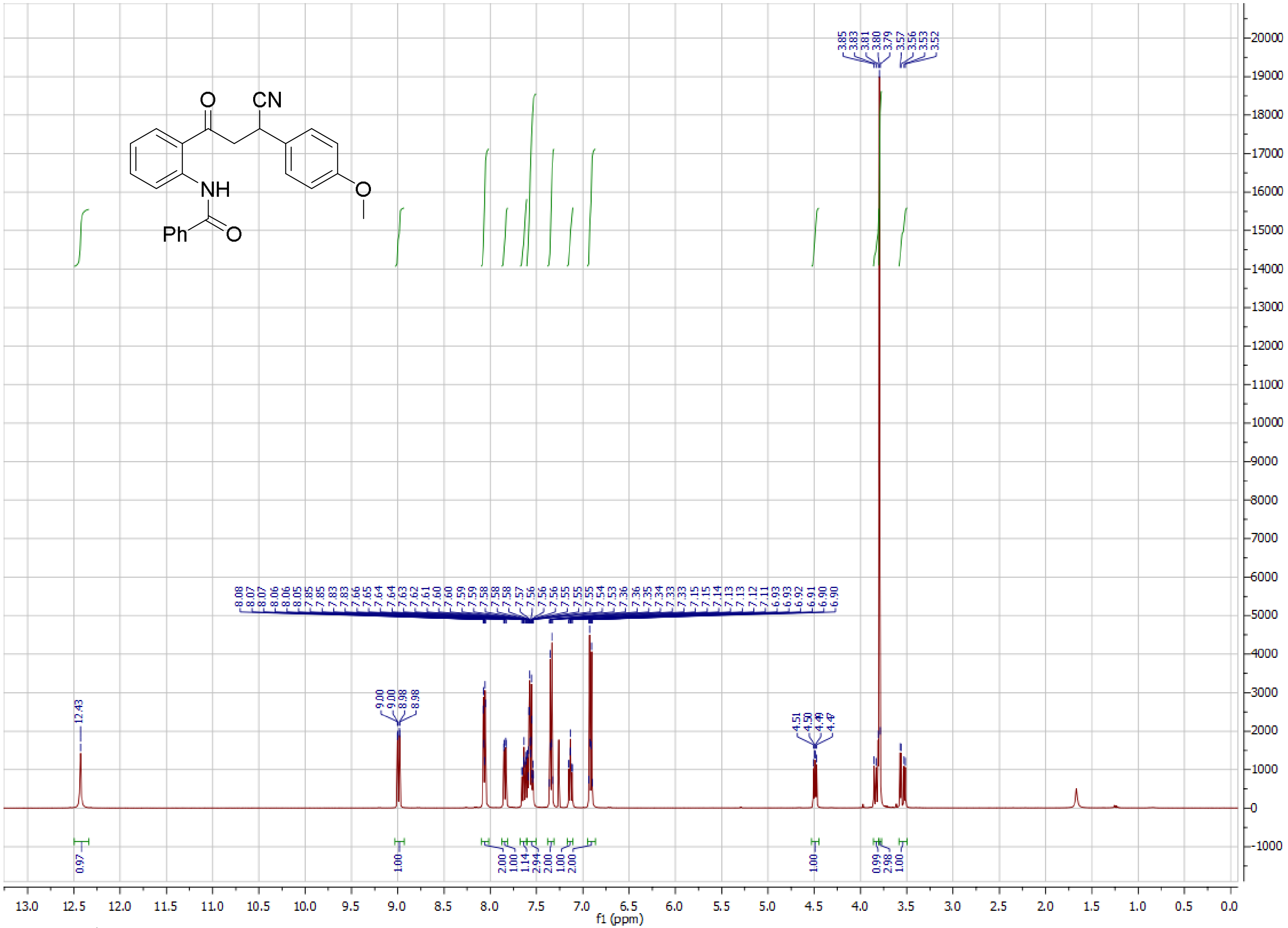

Fig S26. ^{1}H NMR spectral chart for *N*-(2-(3-cyano-3-(4-methoxyphenyl)propanoyl)phenyl)benzamide **9da**

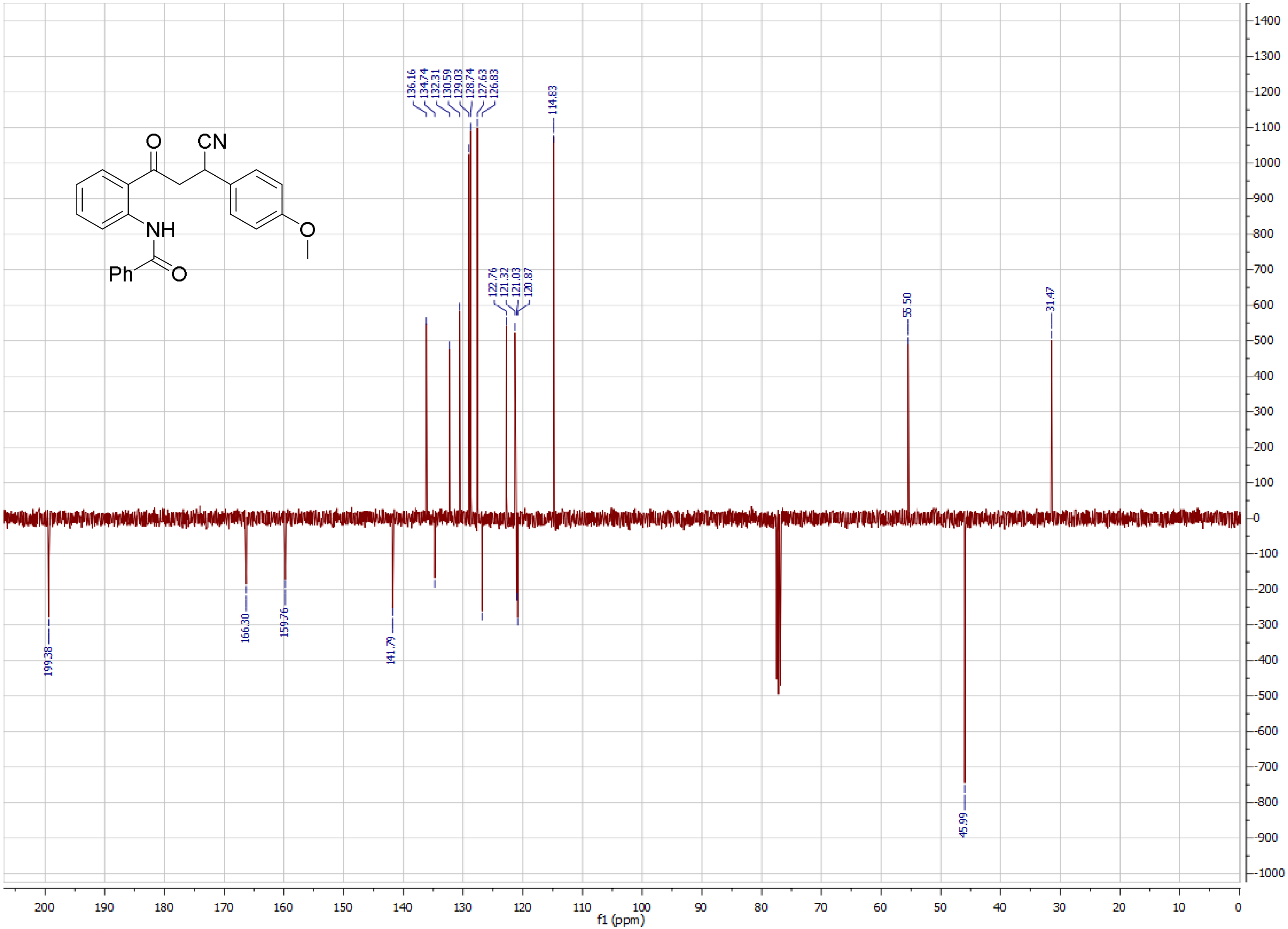

Fig S27. ^{13}C NMR spectral chart for *N*-(2-(3-cyano-3-(4-methoxyphenyl)propanoyl)phenyl)benzamide **9da**

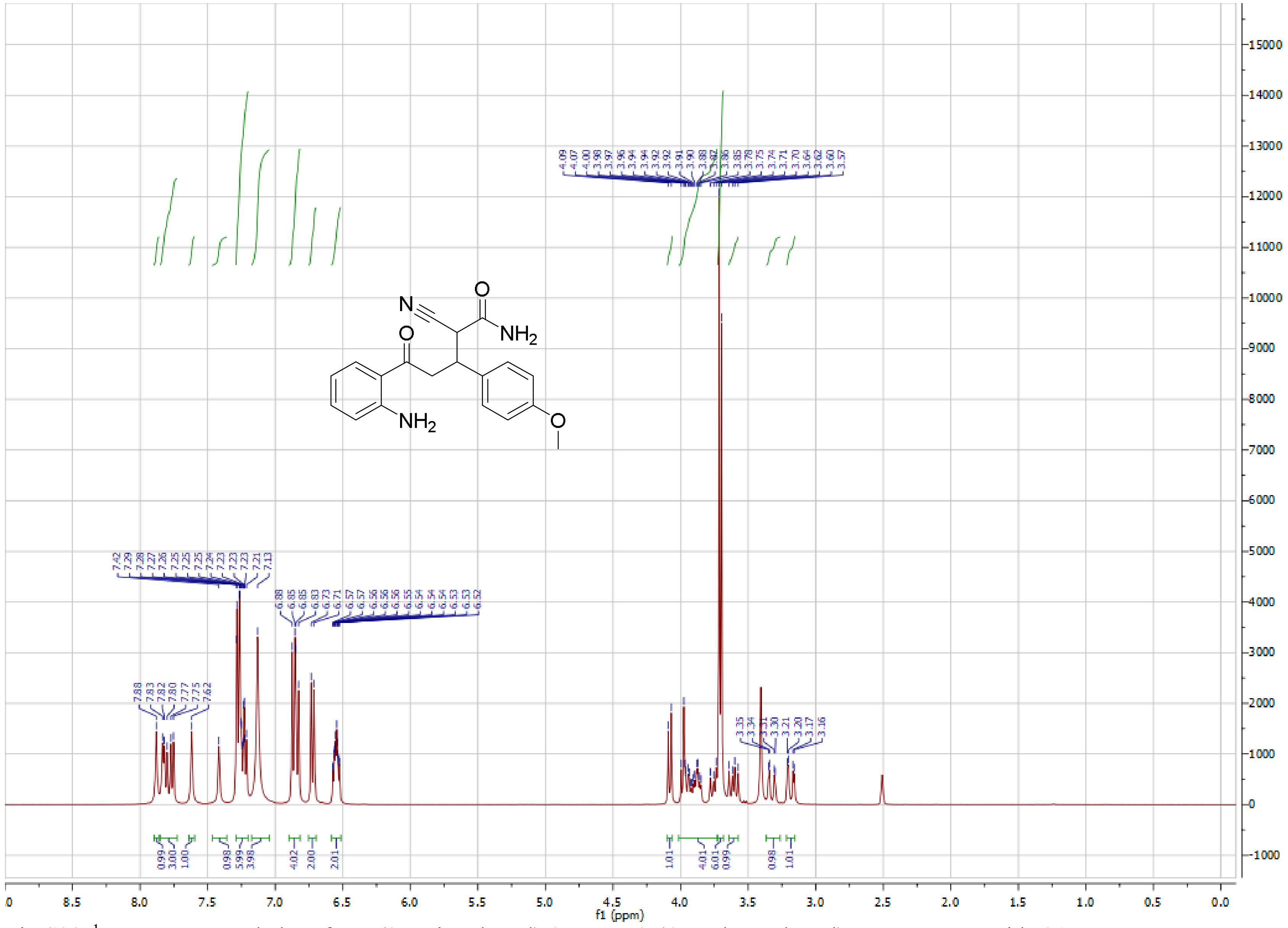

Fig S28. ^{1}H NMR spectral chart for 5-(2-aminophenyl)-2-cyano-3-(4-methoxyphenyl)-5-oxopentanamide **21aa**

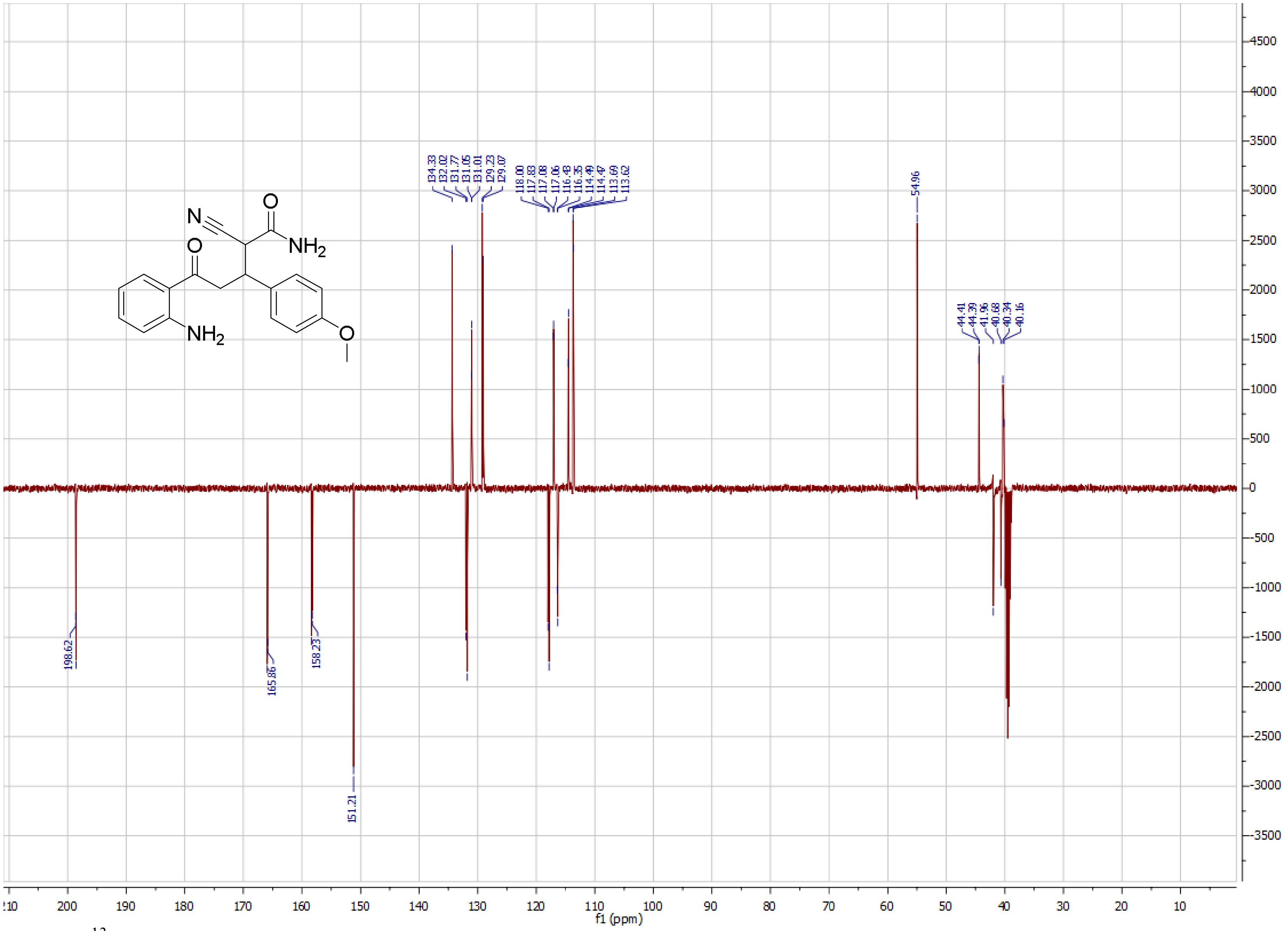

Fig S29. ^{13}C NMR spectral chart for 5-(2-aminophenyl)-2-cyano-3-(4-methoxyphenyl)-5-oxopentanamide **21aa**

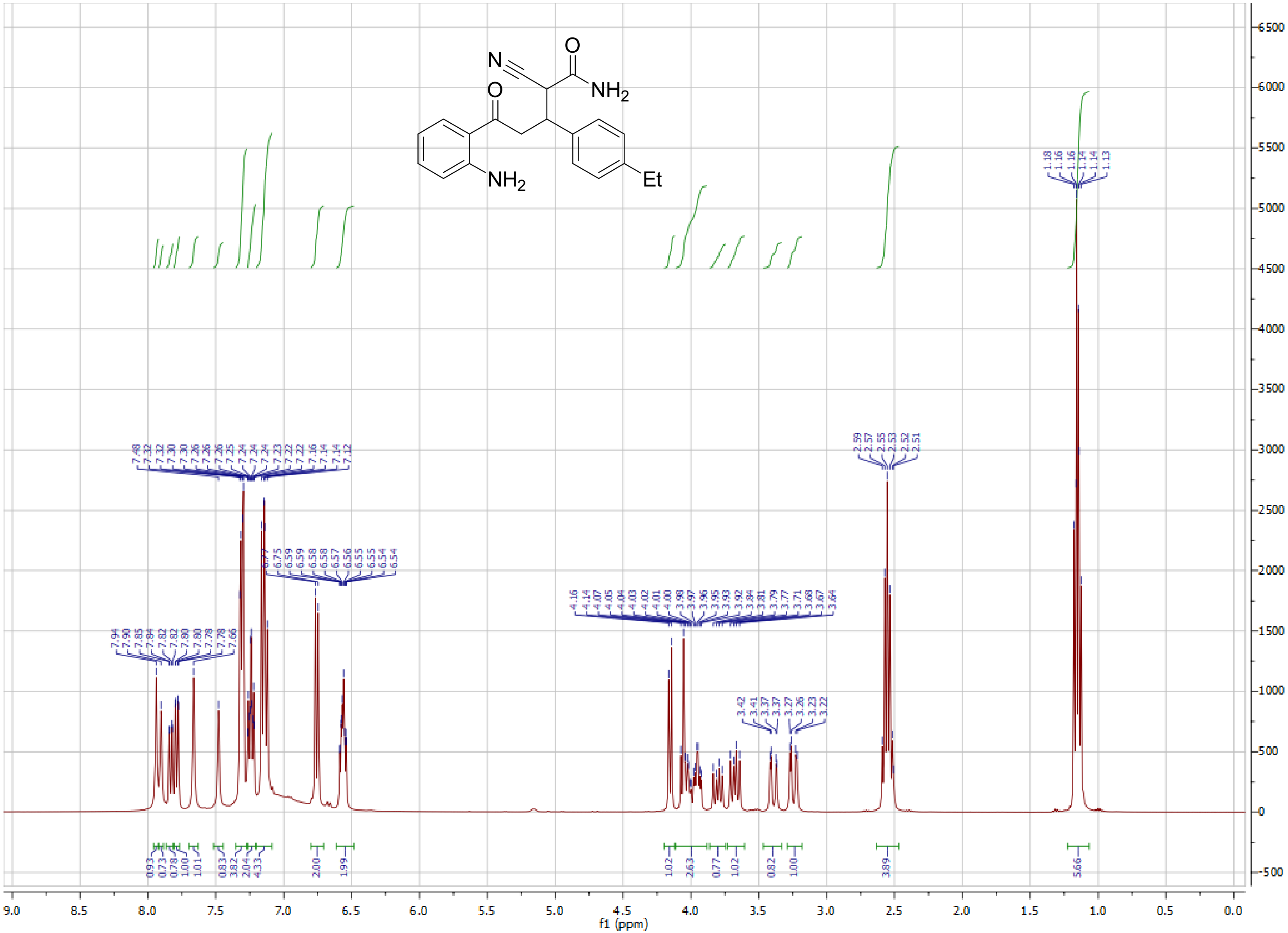

Fig S30. ^{1}H NMR spectral chart for 5-(2-aminophenyl)-2-cyano-3-(4-ethylphenyl)-5-oxopentanamide **21ae**

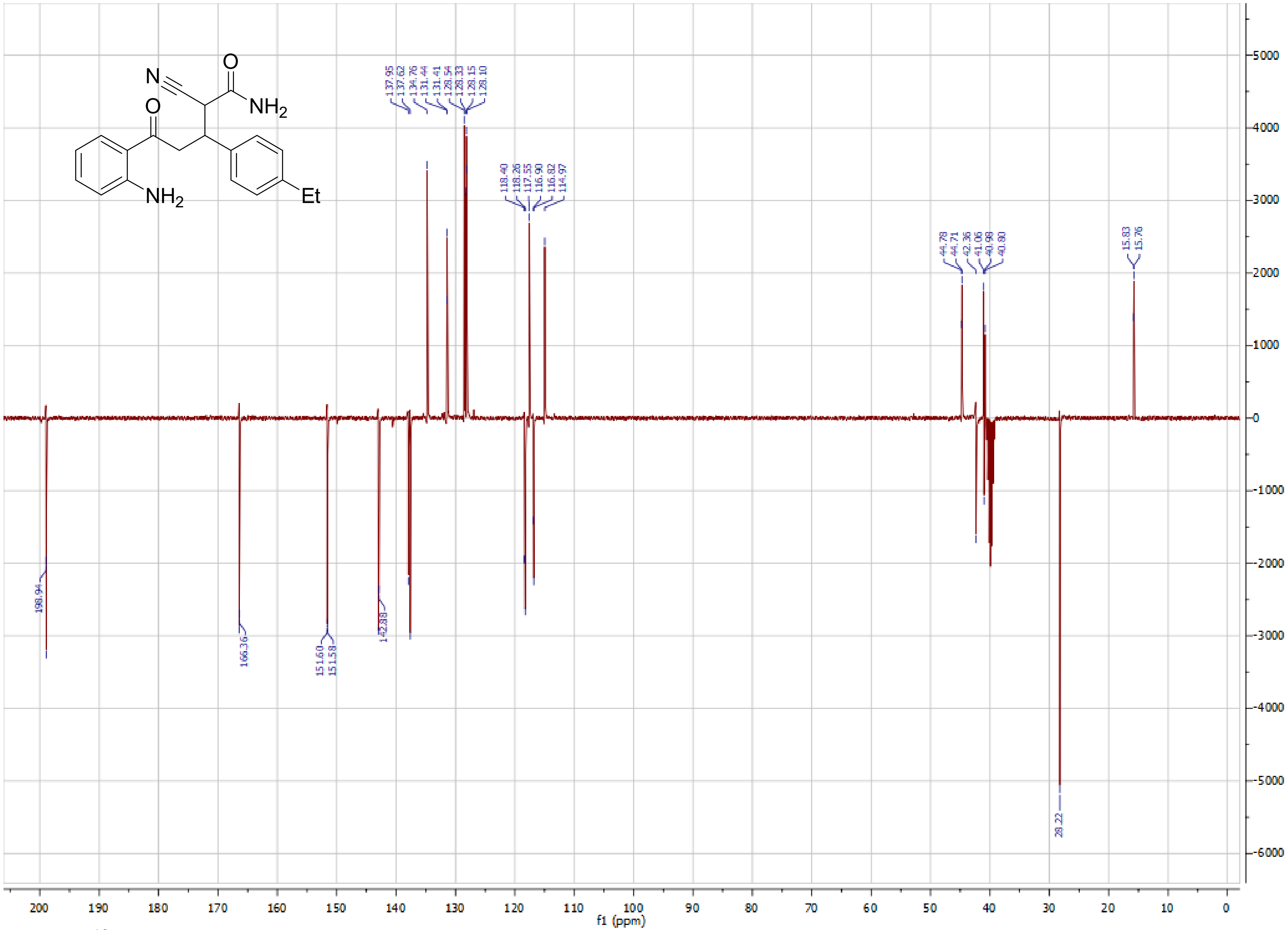

Fig S31. ^{13}C NMR spectral chart for 5-(2-aminophenyl)-2-cyano-3-(4-ethylphenyl)-5-oxopentanamide **21ae**

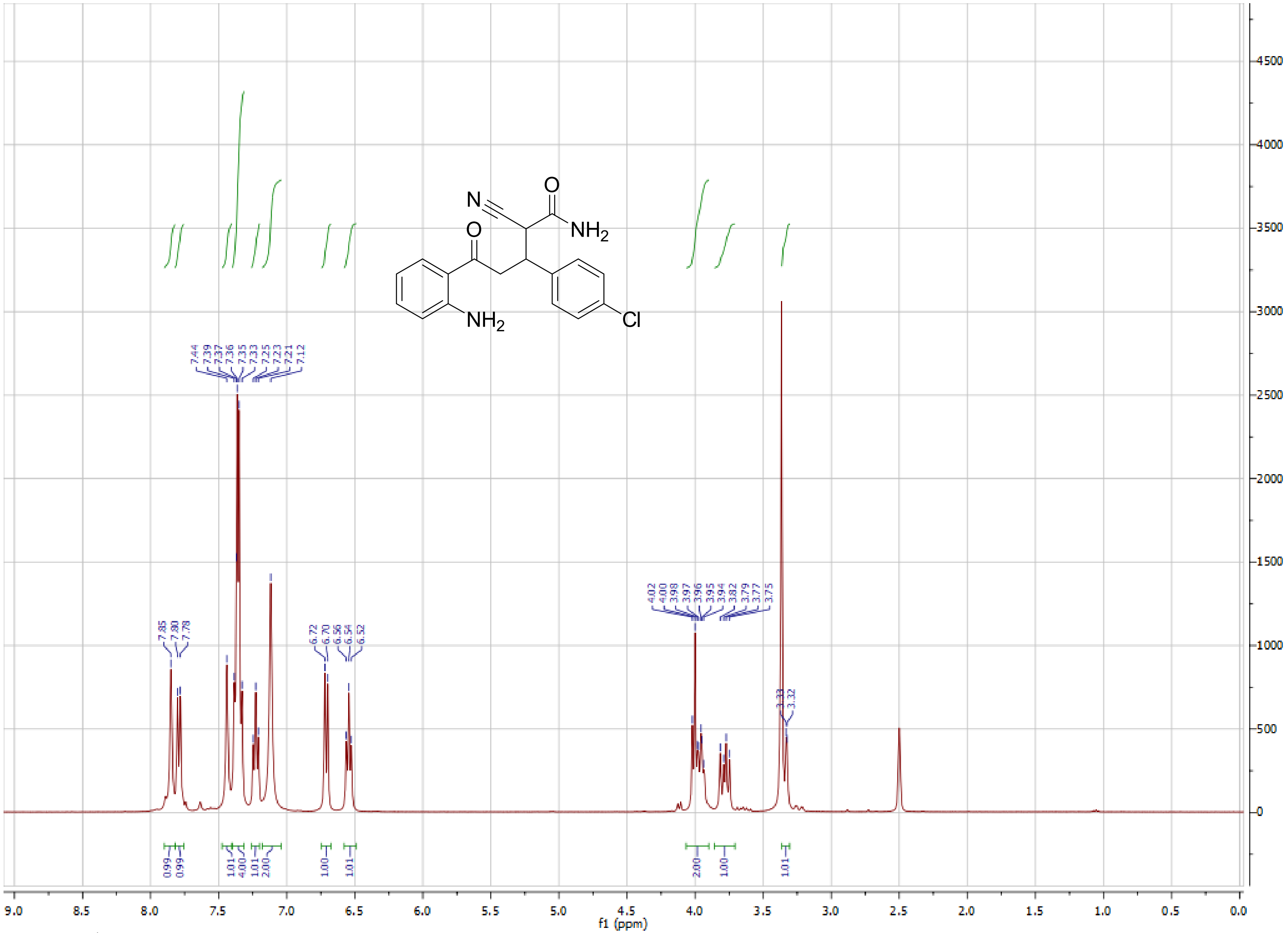

Fig S32. ^{1}H NMR spectral chart for 5-(2-aminophenyl)-3-(4-chlorophenyl)-2-cyano-5-oxopentanamide **21ah**

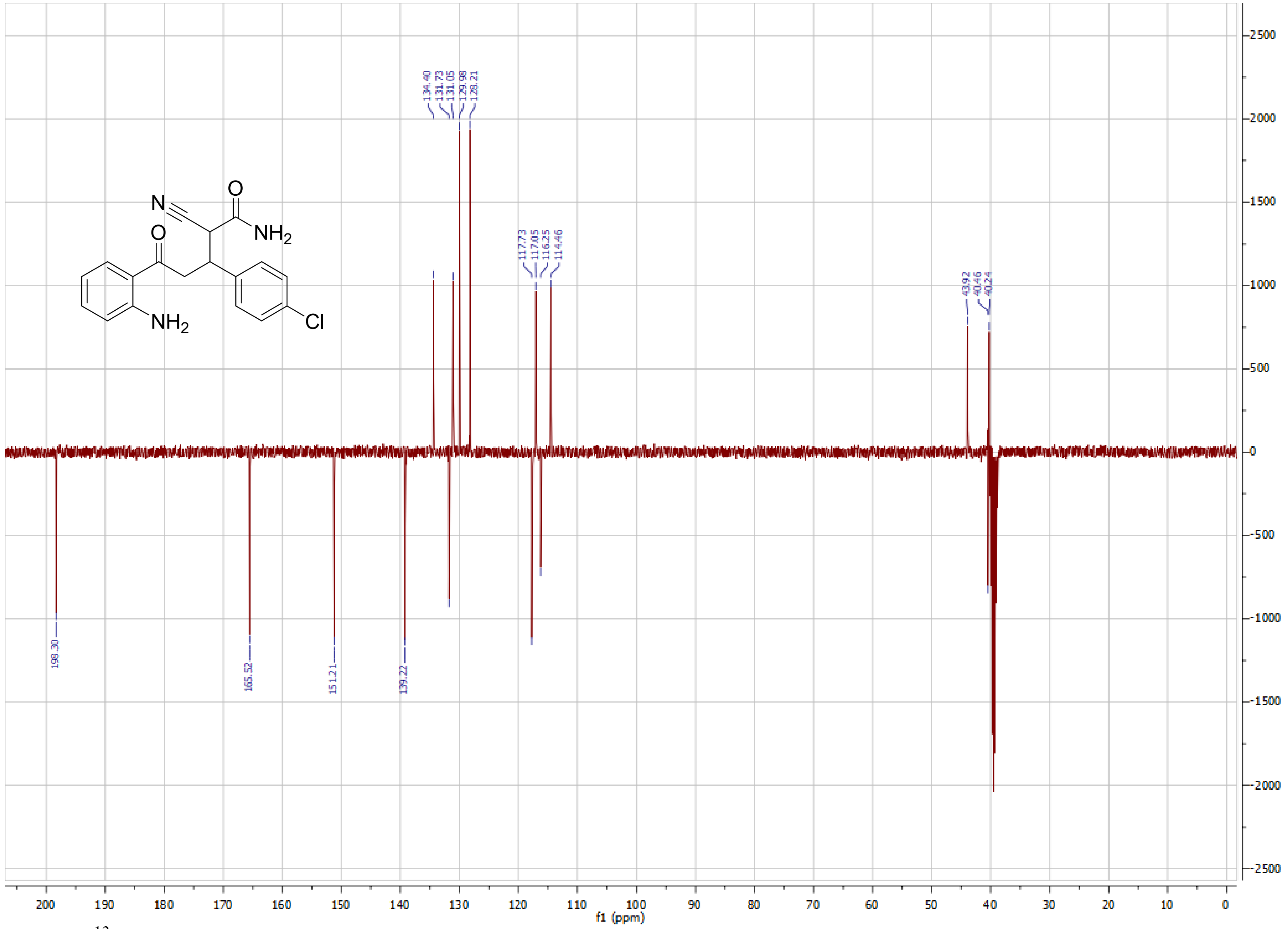

Fig S33. ^{13}C NMR spectral chart for 5-(2-aminophenyl)-3-(4-chlorophenyl)-2-cyano-5-oxopentanamide **21ah**

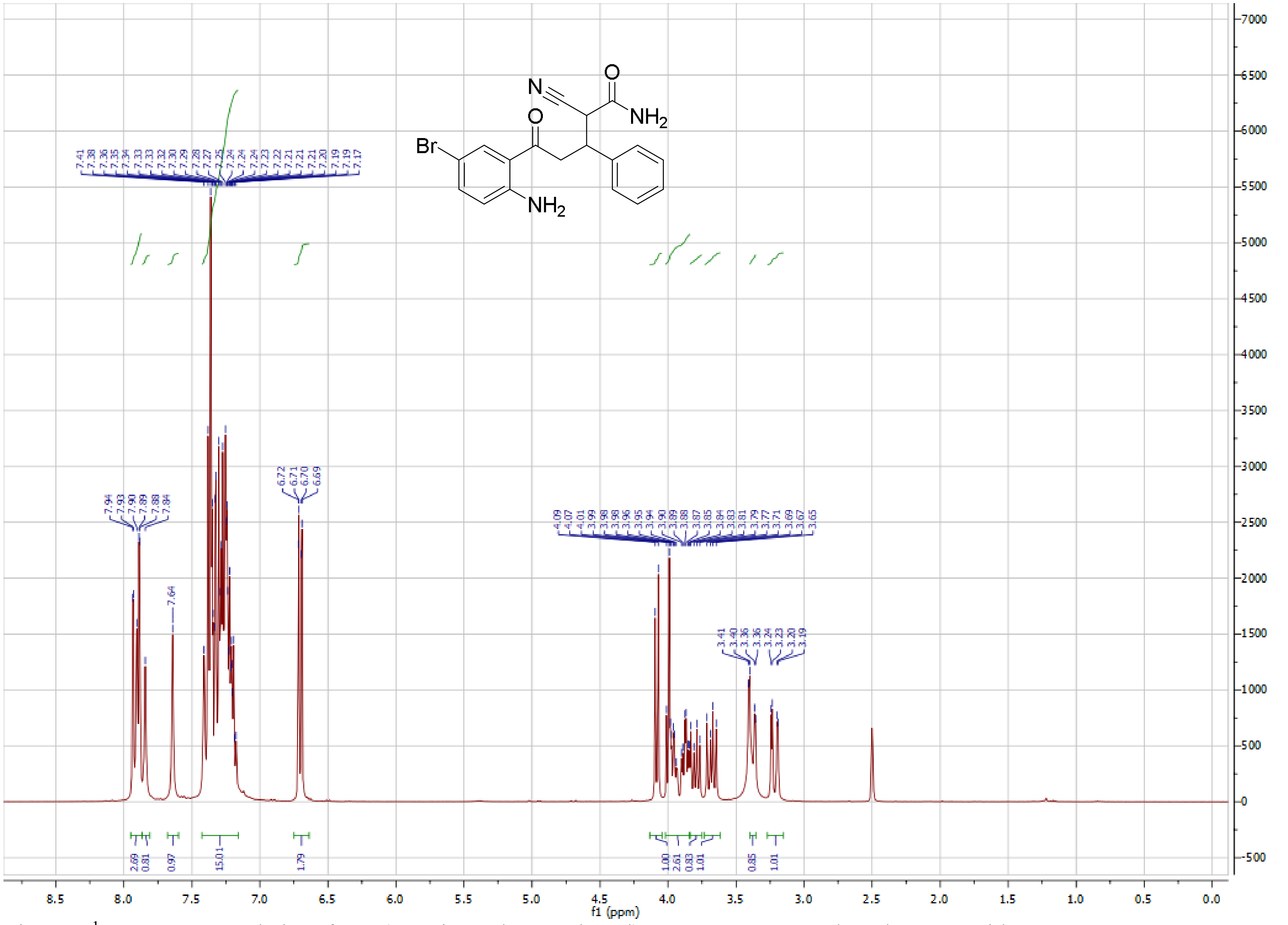

Fig S34. ^{1}H NMR spectral chart for 5-(2-amino-5-bromophenyl)-2-cyano-5-oxo-3-phenylpentanamide **21al**

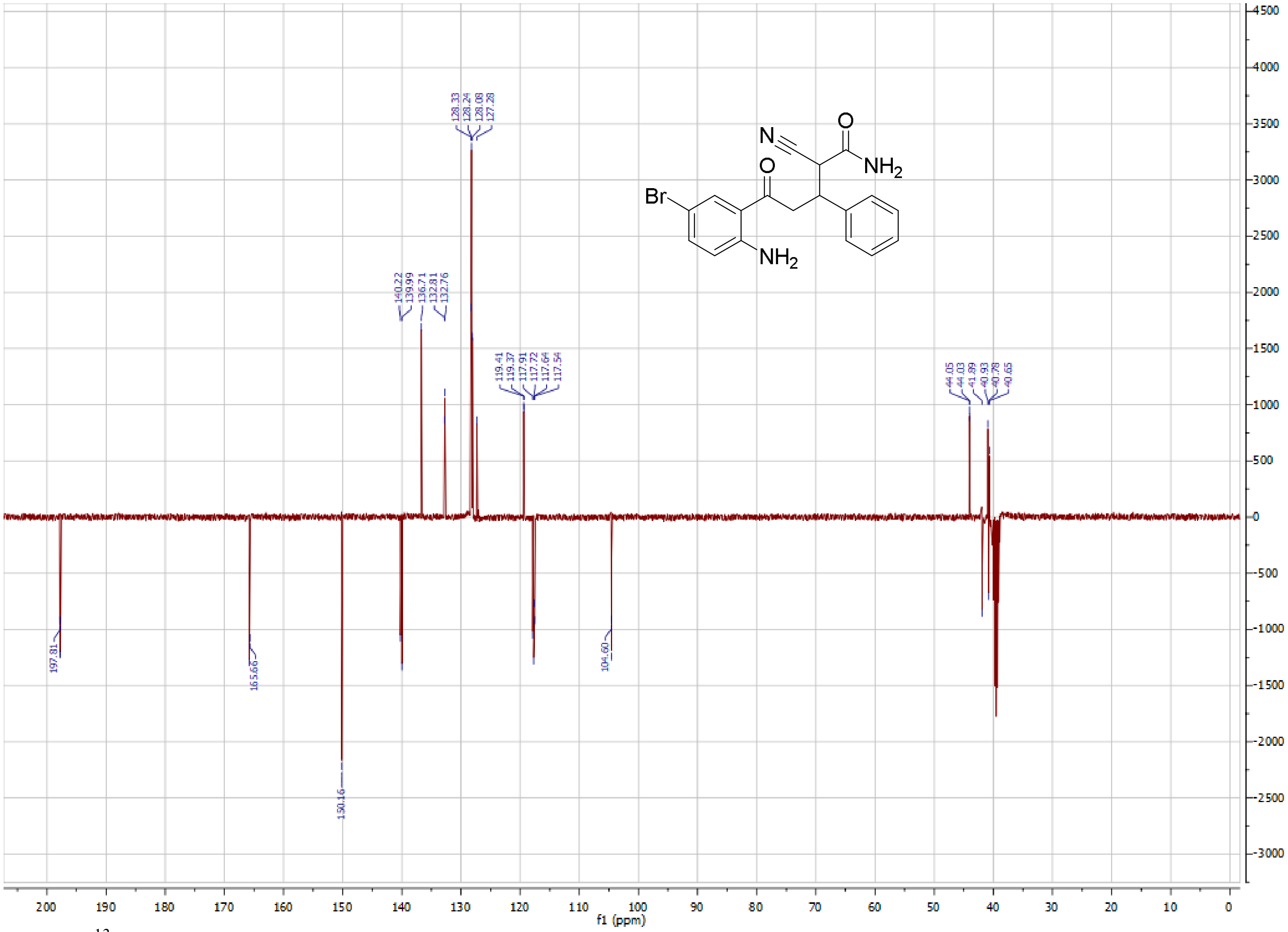

Fig S35. ^{13}C NMR spectral chart for 5-(2-amino-5-bromophenyl)-2-cyano-5-oxo-3-phenylpentanamide **21al**

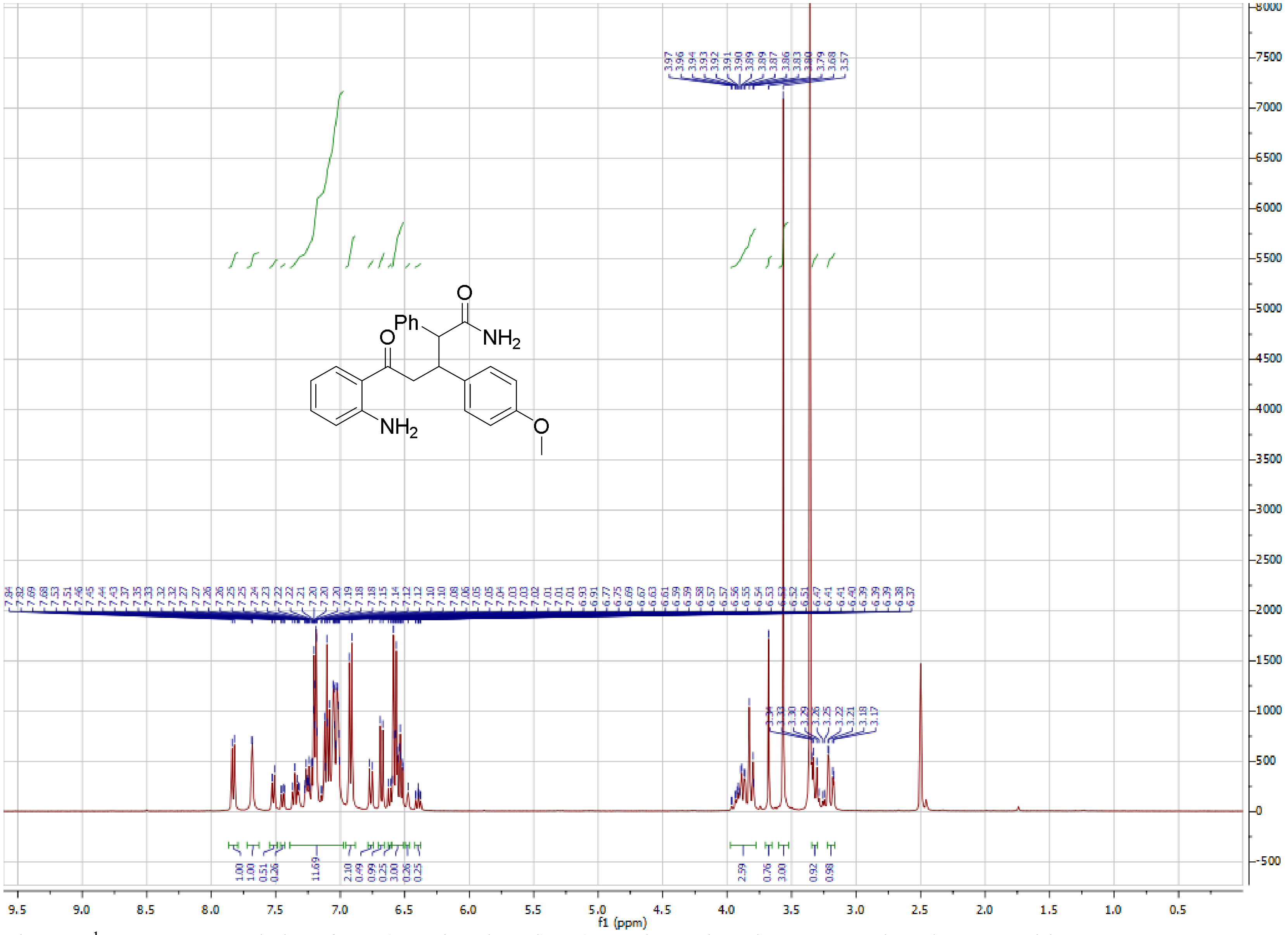

Fig S36. ^{1}H NMR spectral chart for 5-(2-aminophenyl)-3-(4-methoxyphenyl)-5-oxo-2-p henylpentanamide **21bb**

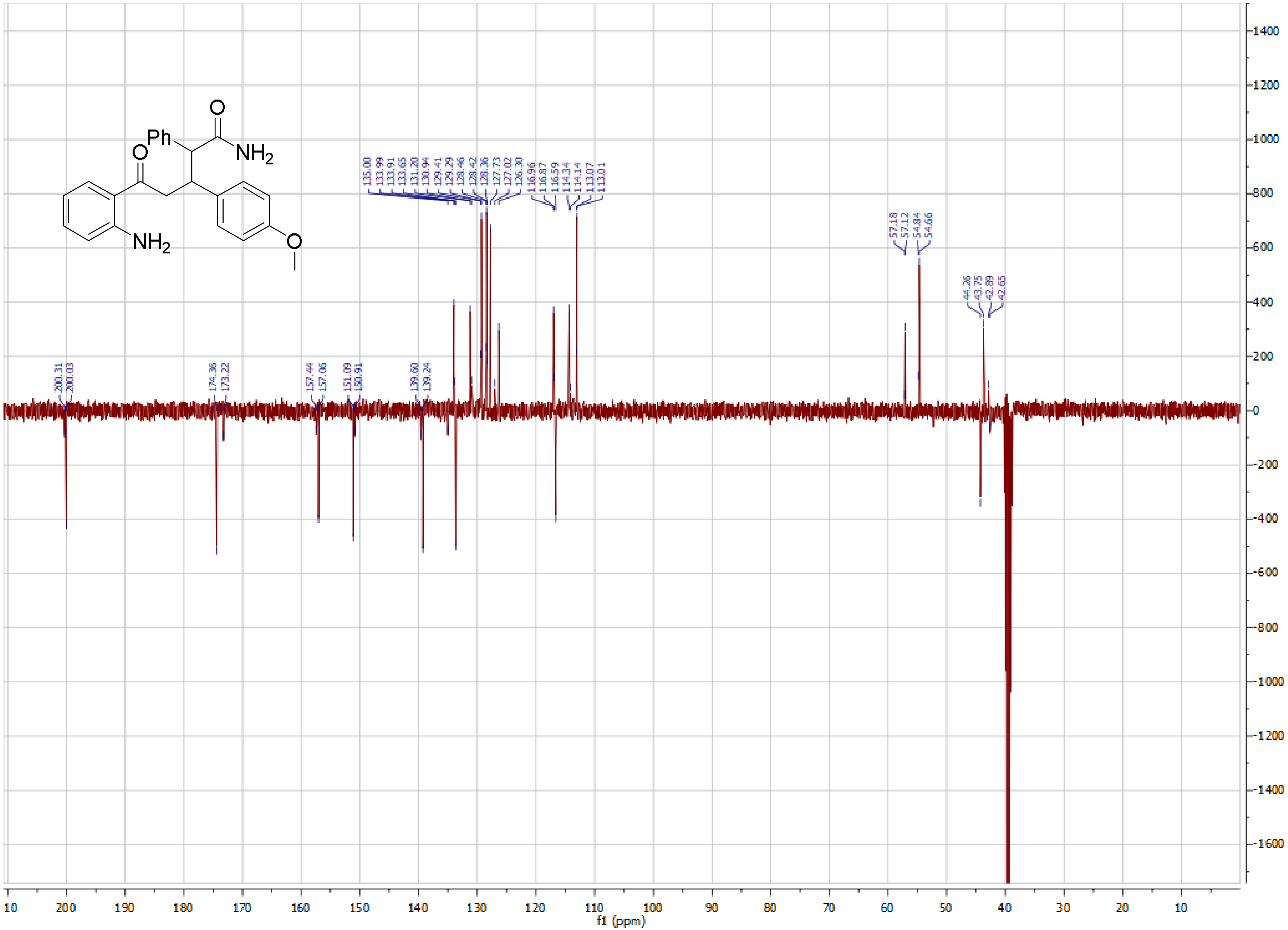

Fig S37. ^{13}C NMR spectral chart for 5-(2-aminophenyl)-3-(4-methoxyphenyl)-5-oxo-2-phenylpentanamide **21bb**

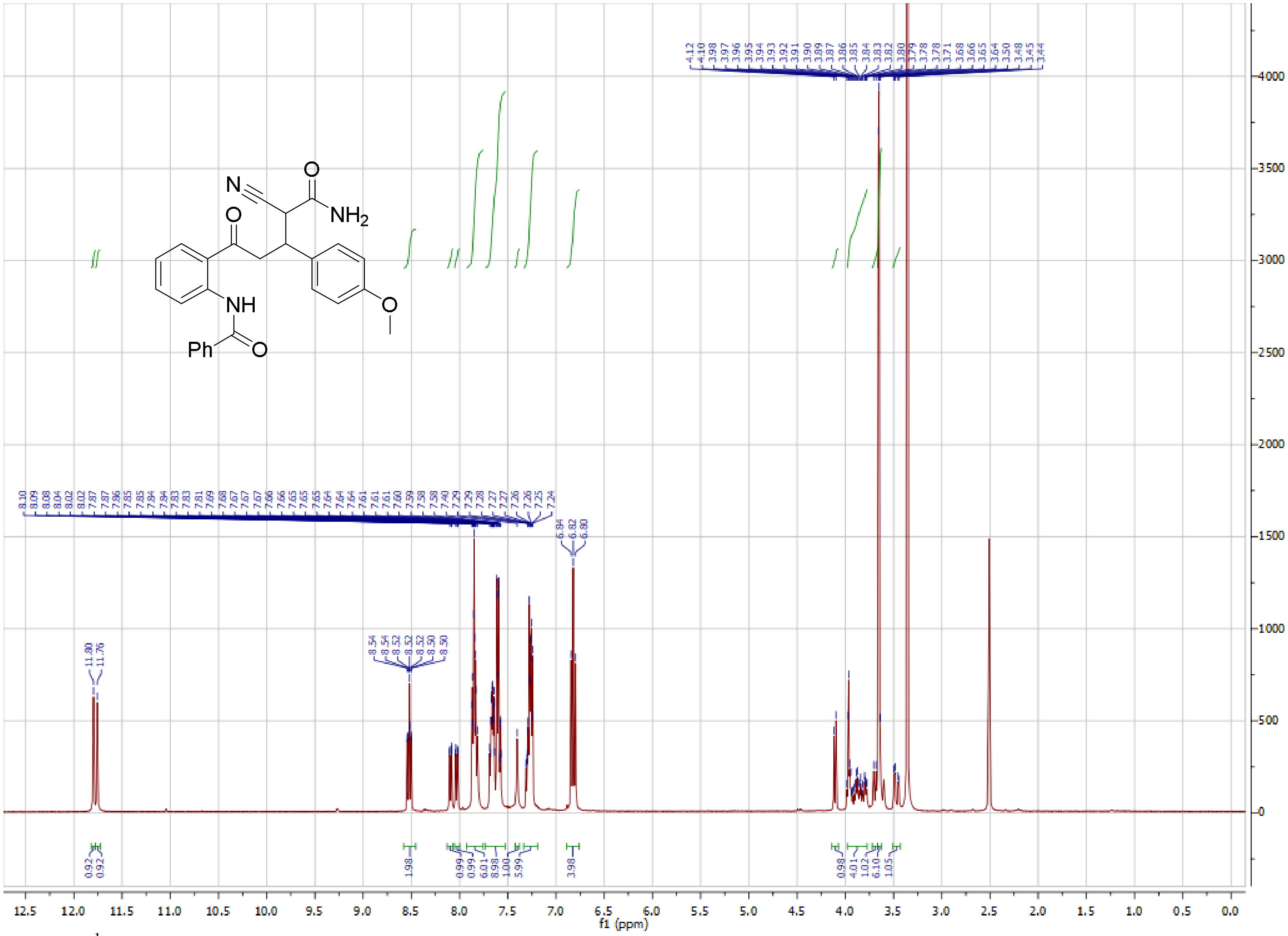

Fig S38. ^{1}H NMR spectral chart for *N*-(2-(5-amino-4-cyano-3-(4-methoxyphenyl)-5-oxopentanoyl)phenyl)benzamid e **24**

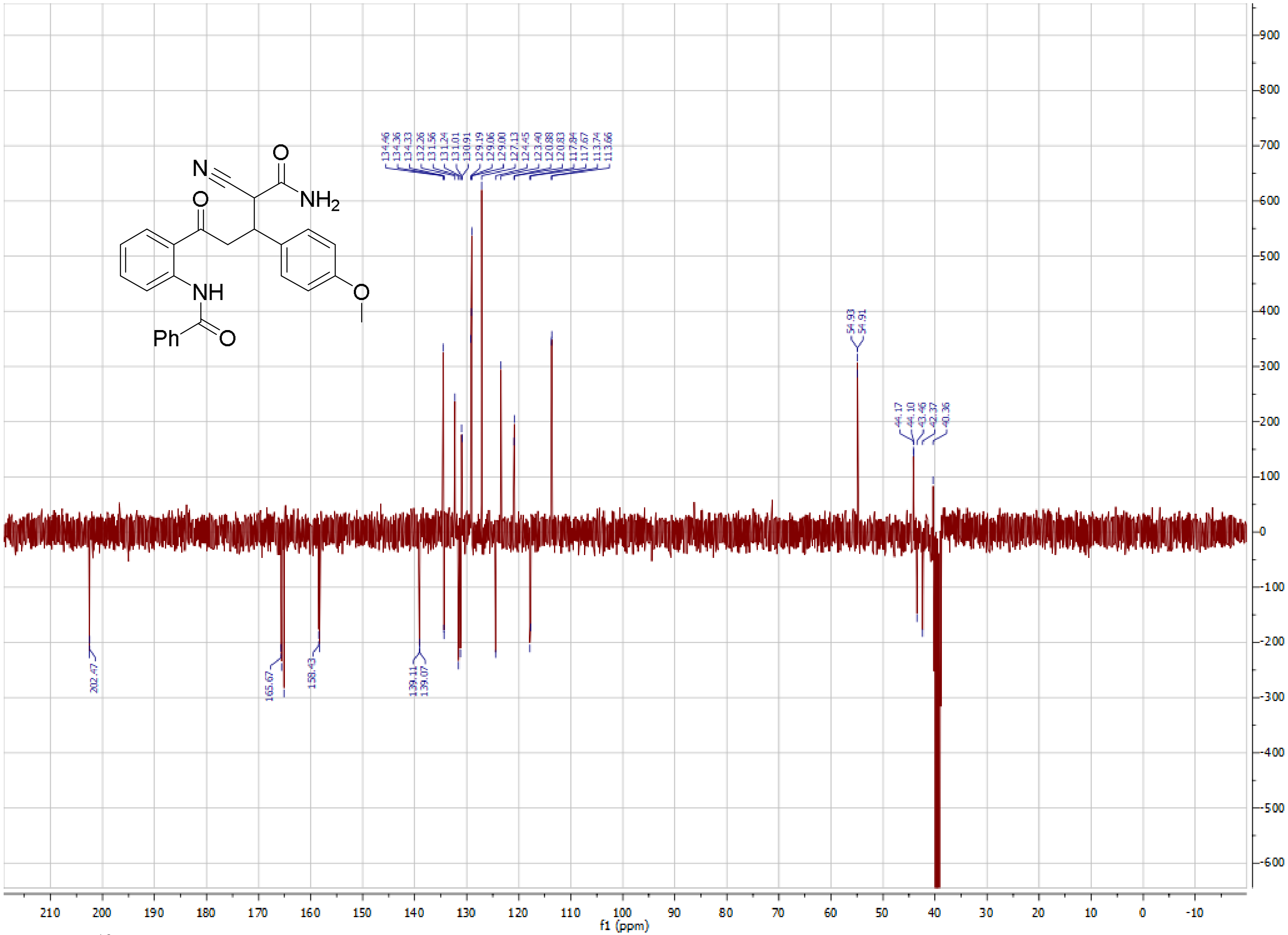

Fig S39. ^{13}C NMR spectral chart for *N*-(2-(5-amino-4-cyano-3-(4-methoxyphenyl)-5-oxopentanoyl)phenyl)benzamide **24**

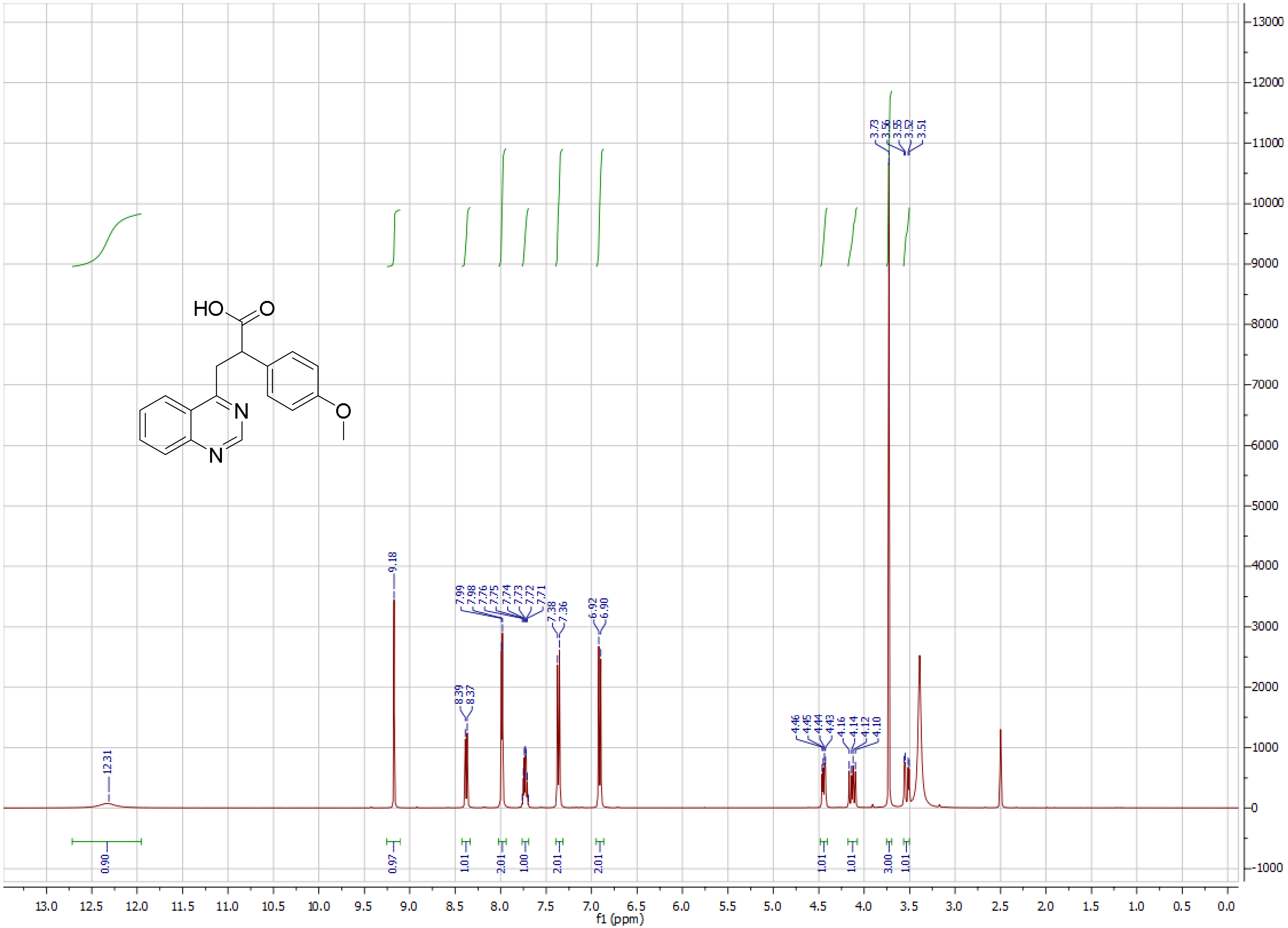

Fig S40. ^{1}H NMR spectral chart for 2-(4-methoxyphen yl)-3-(quinazolin-4-yl)propanoic acid **11aa**

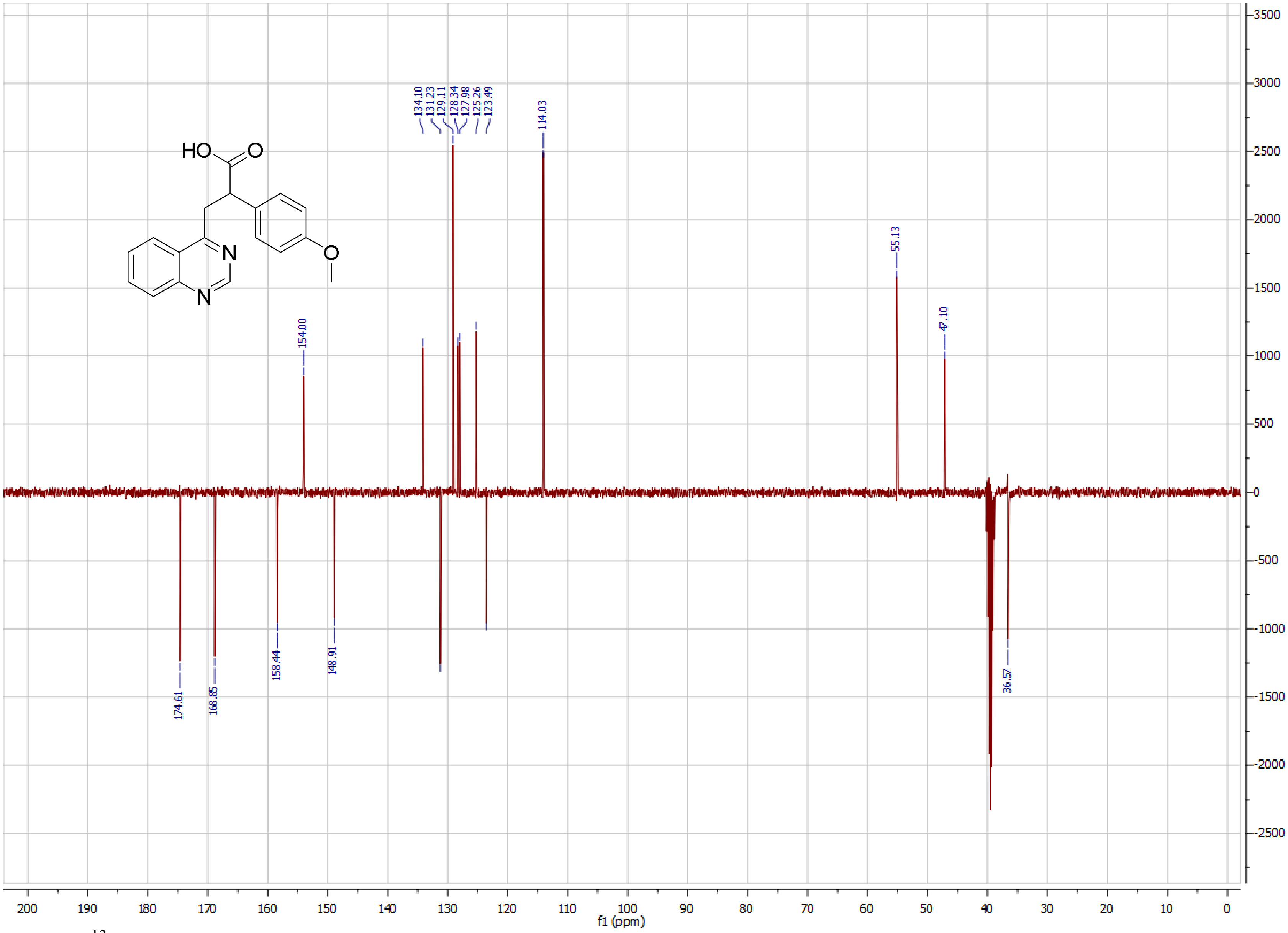

Fig S41. ^{13}C NMR spectral chart for 2-(4-methoxy phenyl)-3-(quinazolin-4-yl)propanoic acid **11aa**

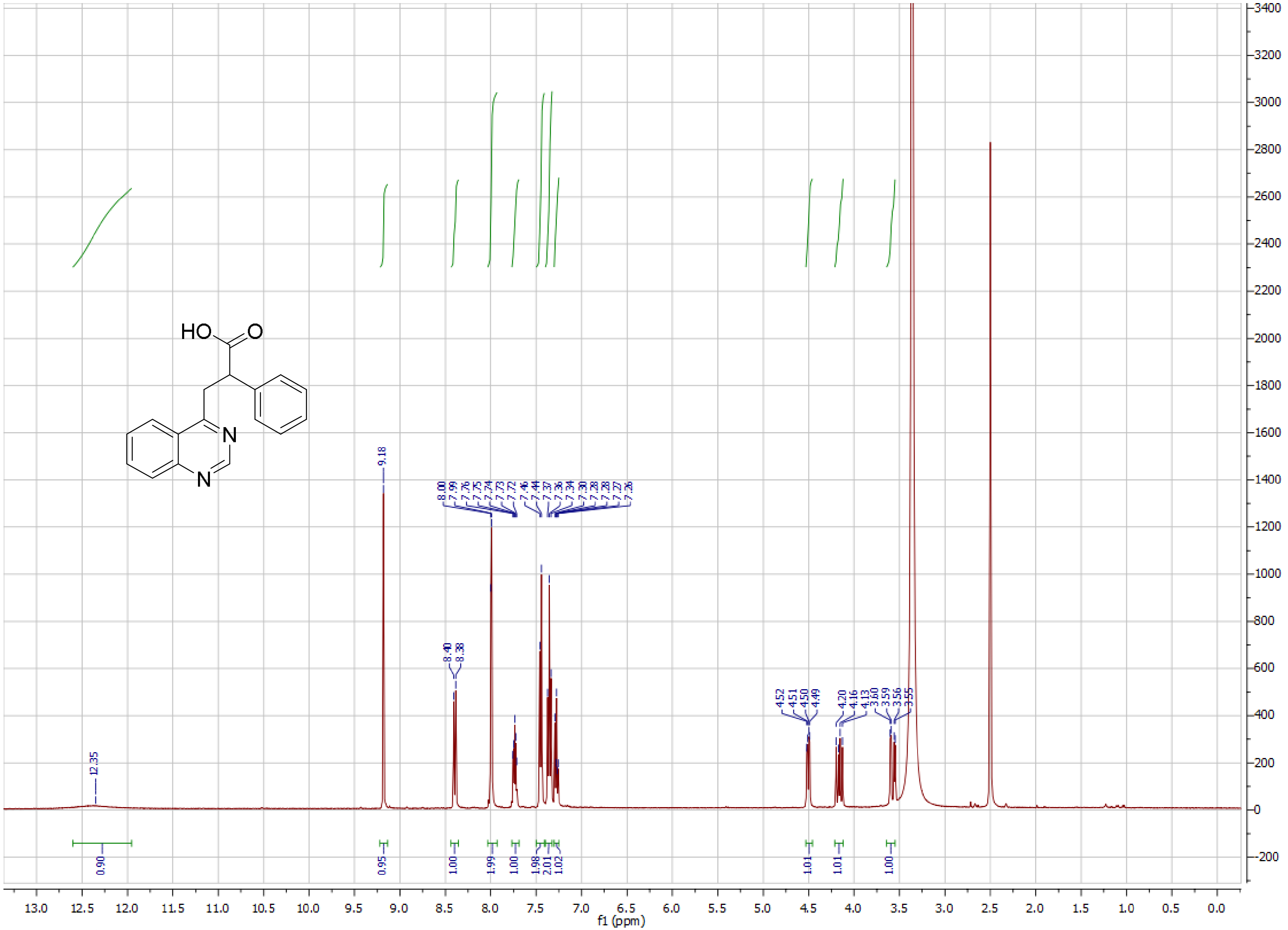

Fig S42. ^{1}H NMR spectral chart for 2-phenyl-3-(q uinazolin-4-yl)propanoic acid **11ab**

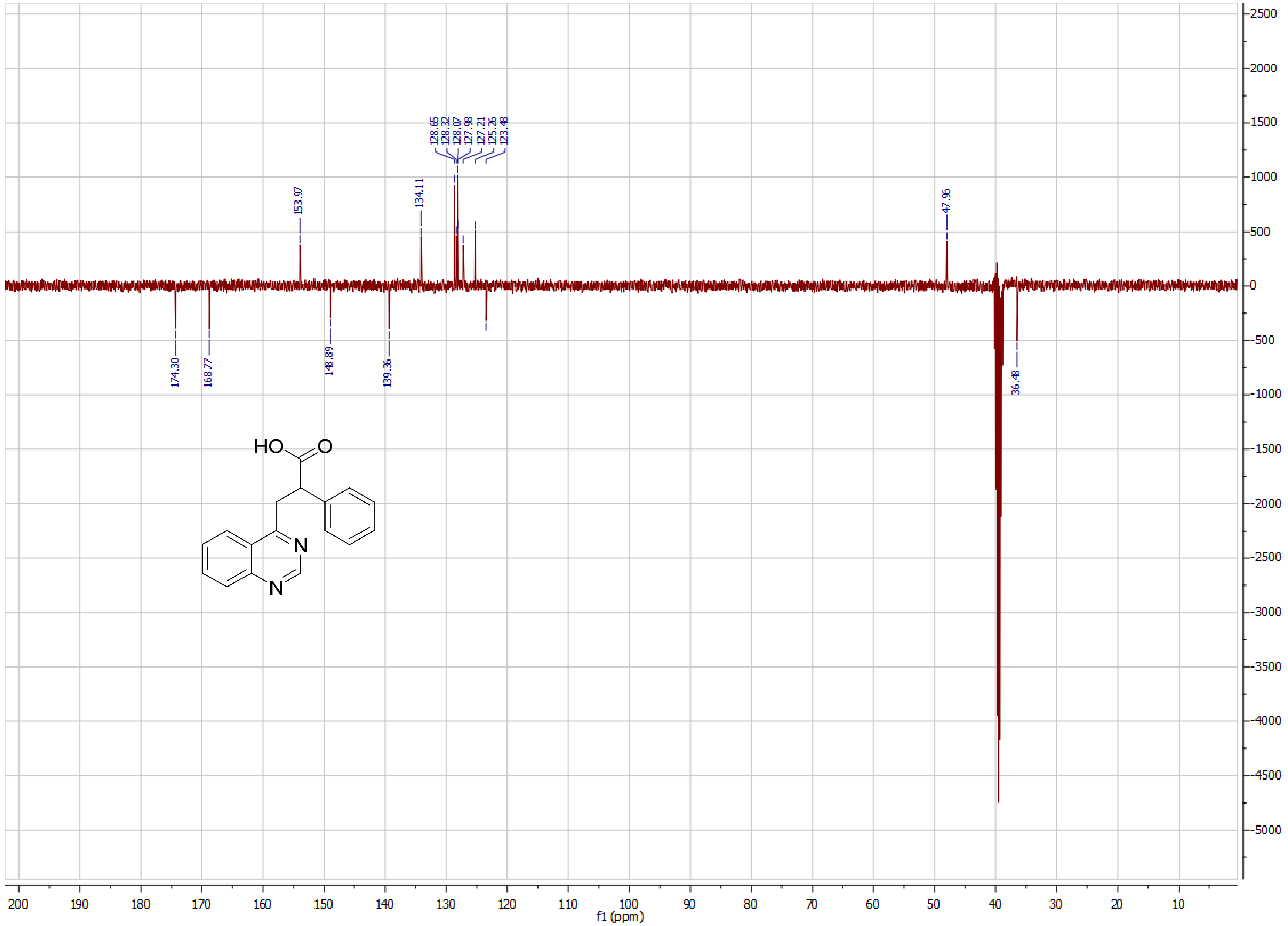

Fig S43. ^{13}C NMR spectral chart for 2-phenyl-3-(quinazolin-4-yl)pro panoic acid **11ab**

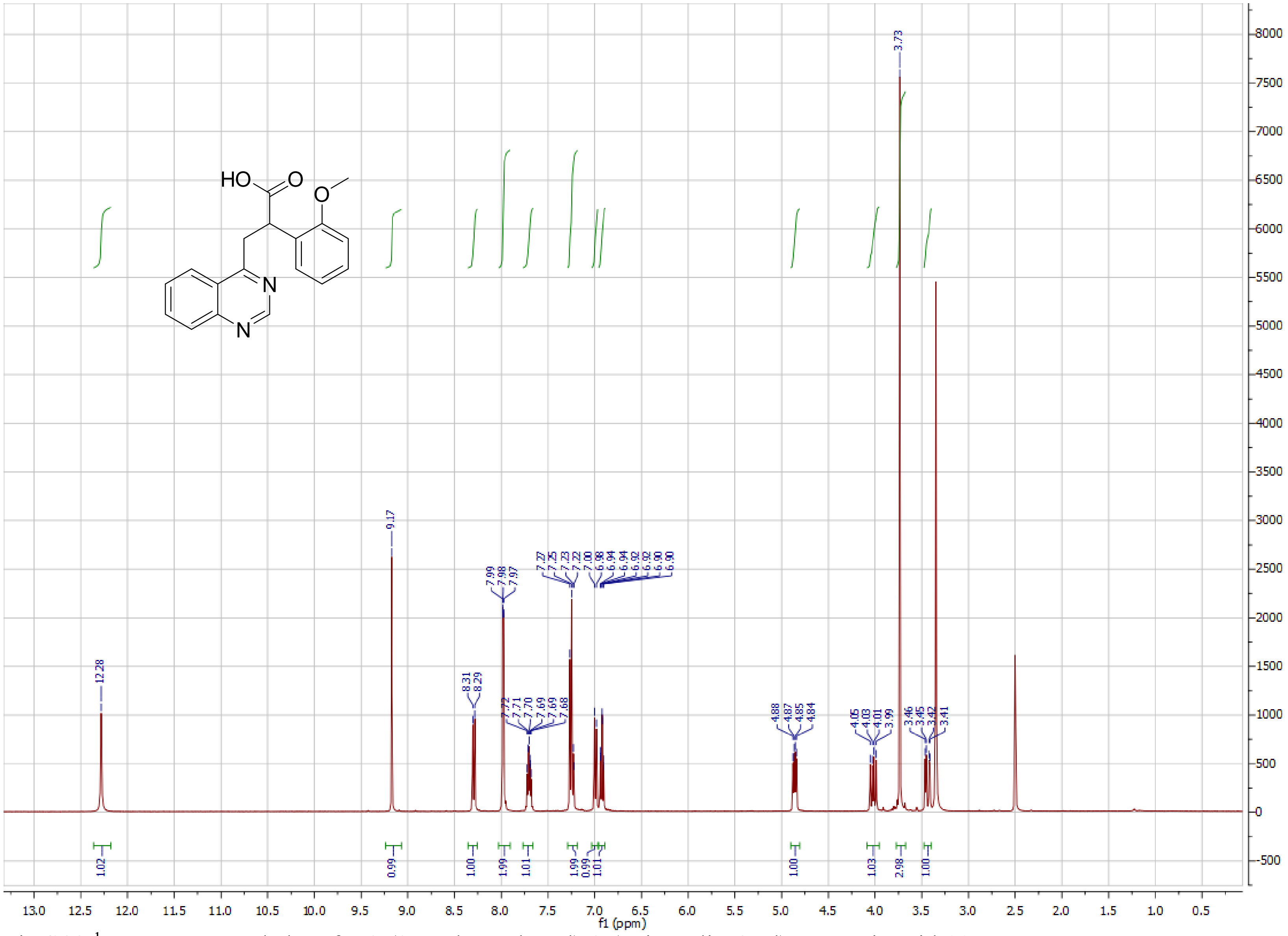

Fig S44. ^{1}H NMR spectral chart for 2-(2-methoxyphenyl)-3-(quinazolin-4-y l)propanoic acid **11ac**

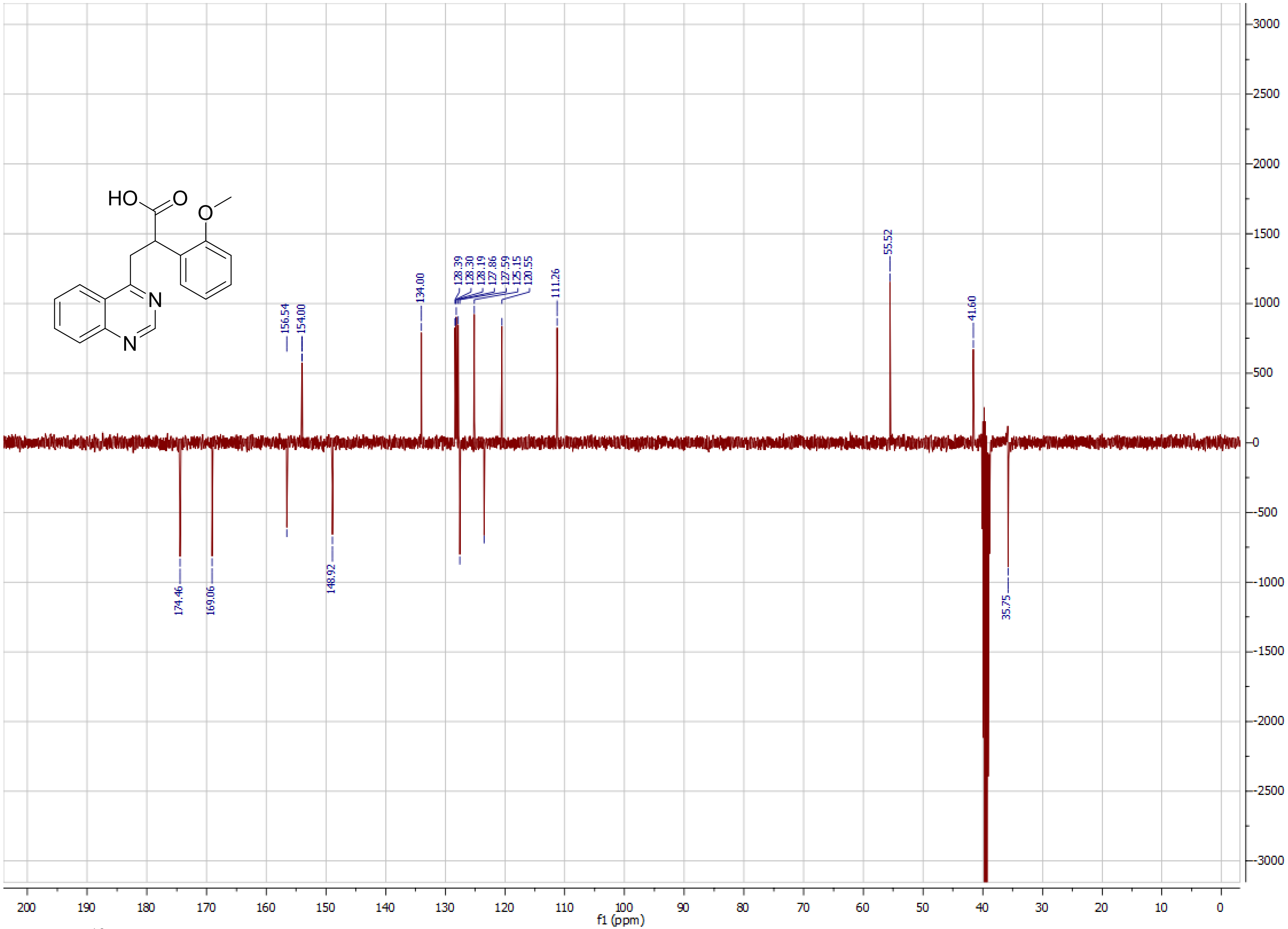

Fig S45. ^{13}C NMR spectral chart for 2-(2-methoxyphen yl)-3-(quinazolin-4-yl)propanoic acid **11ac**

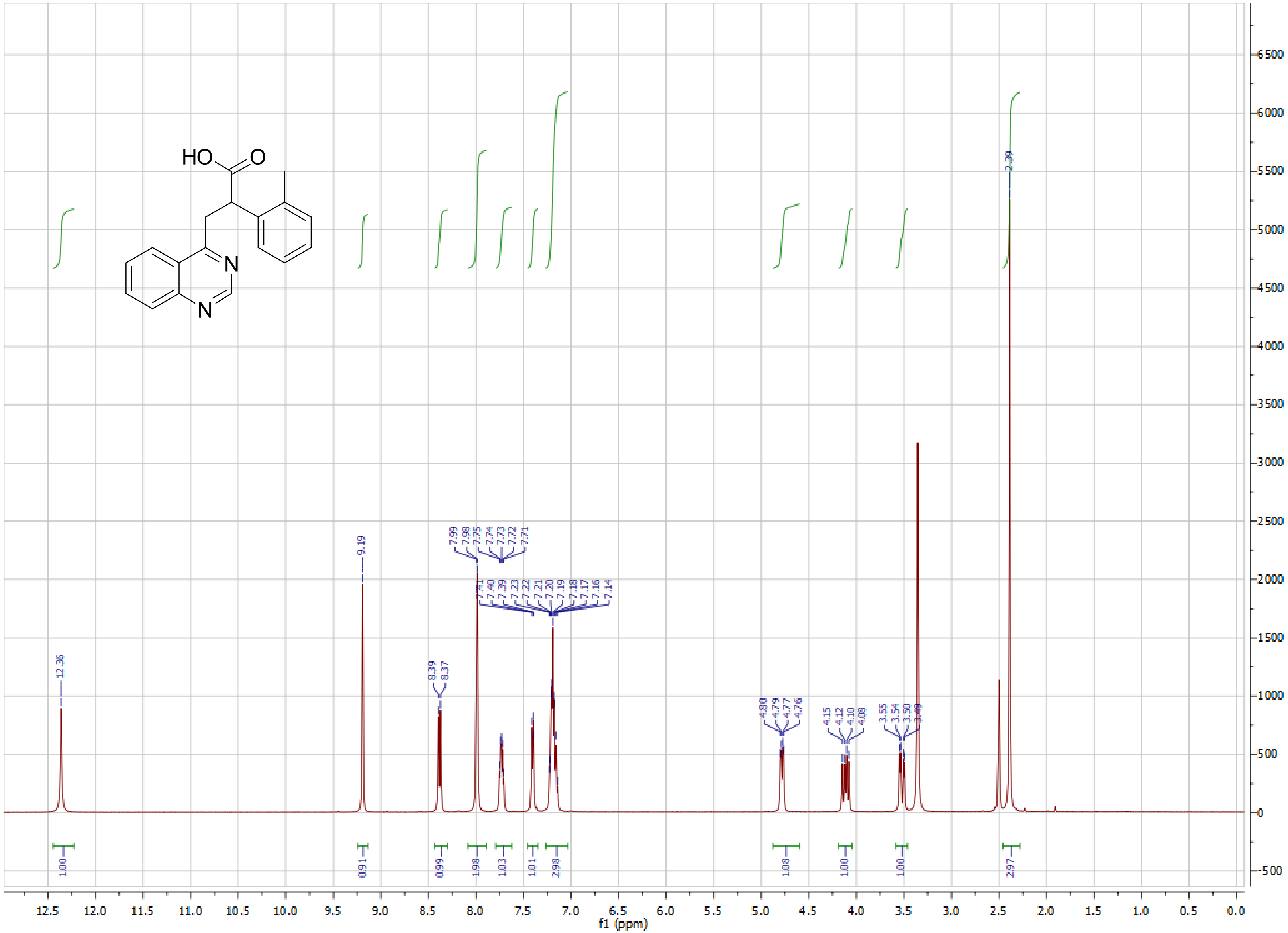

Fig S46. ^{1}H NMR spectral chart for 3-(quinazolin-4-yl)- 2-(o-tolyl)propanoic acid **11ad**

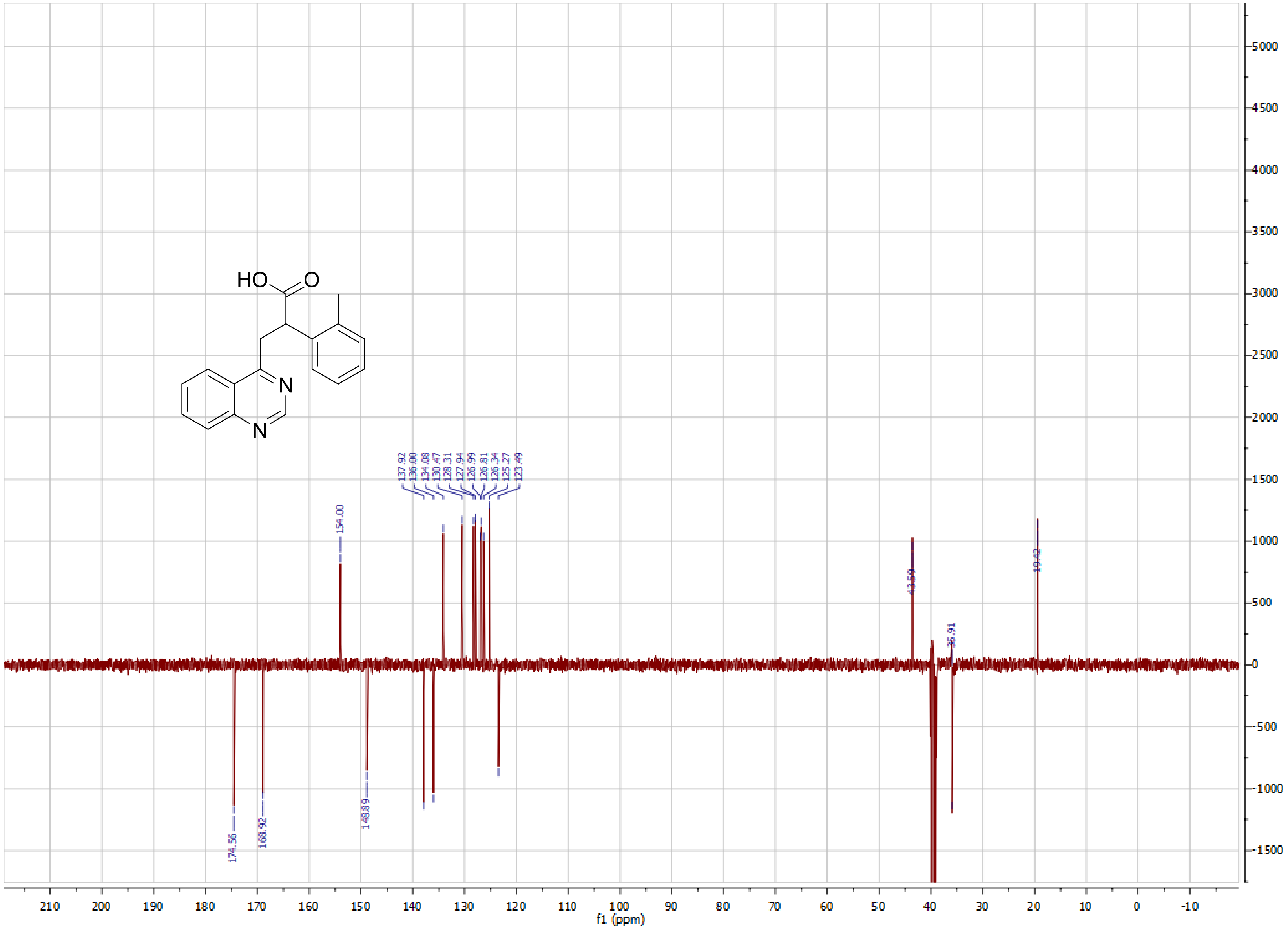

Fig S47. ^{13}C NMR spectral chart for 3-(quinazoli n-4-yl)-2-(o-tolyl)propanoic acid **11ad**

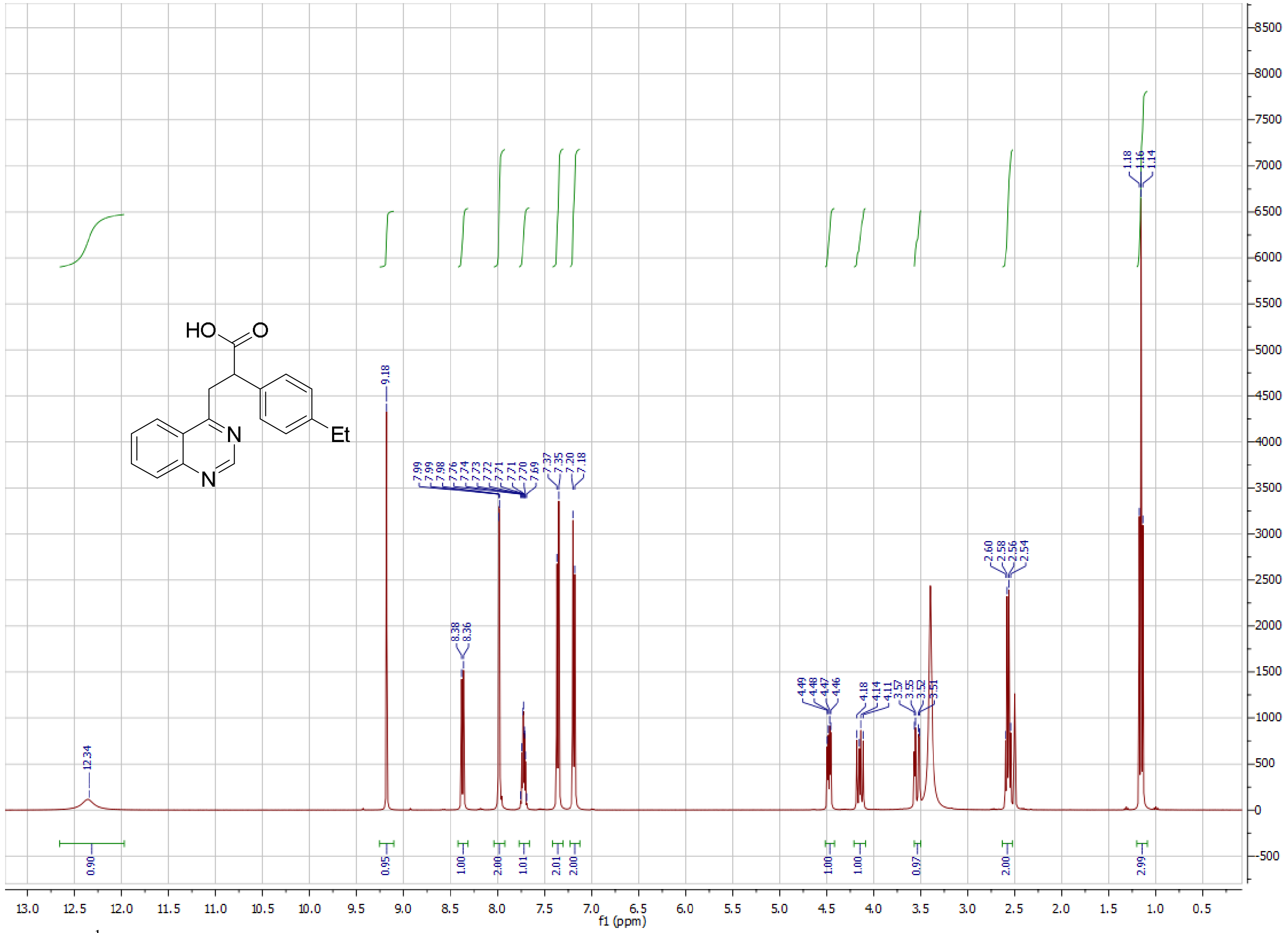

Fig S48. ^{1}H NMR spectral chart for 2-(4-ethylphenyl)-3-(quinaz olin-4-yl)propanoic acid **11ae**

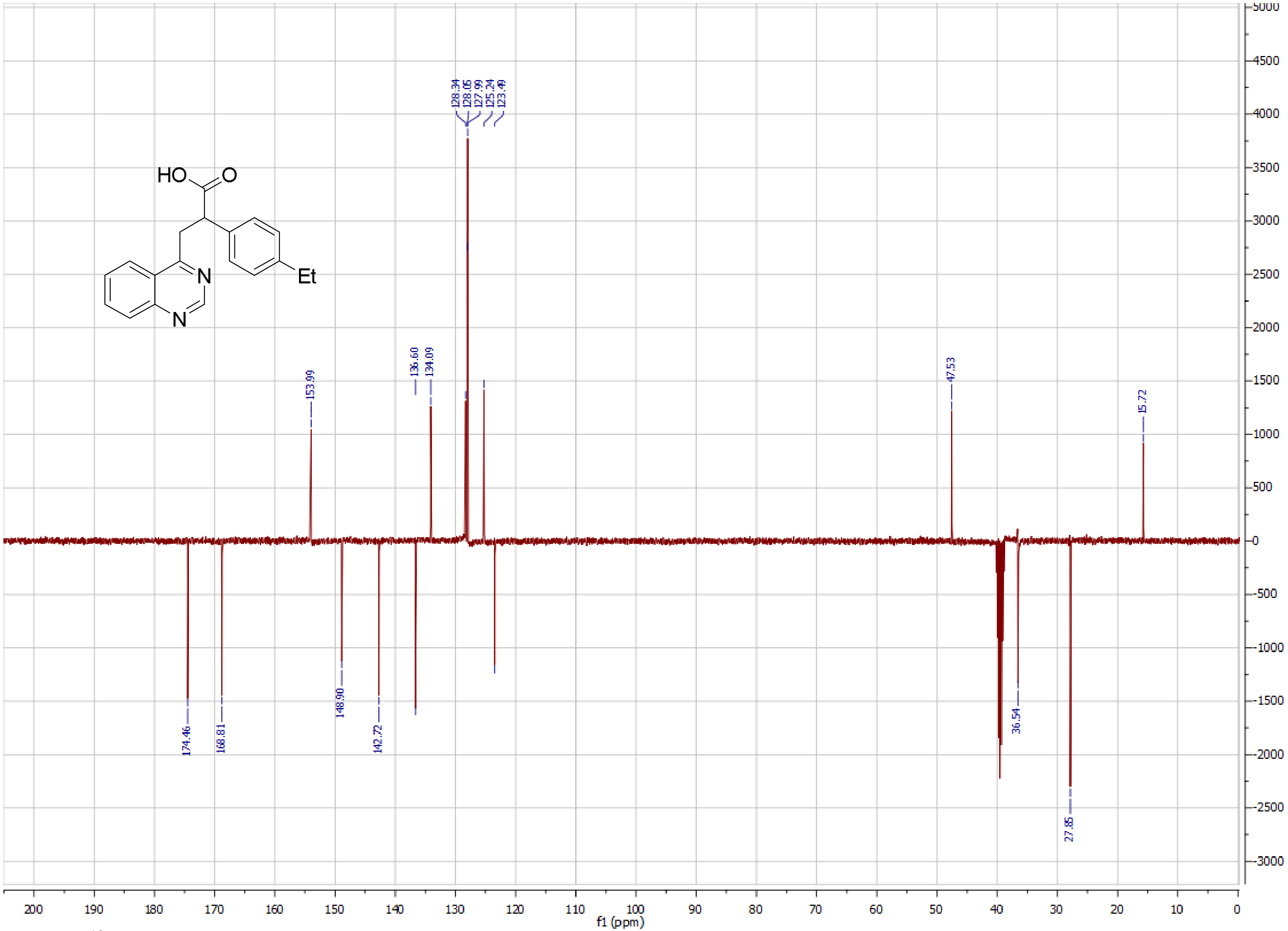

Fig S49. ^{13}C NMR spectral chart for 2-(4-ethylphenyl)-3-(quinazolin-4-yl)propanoic acid **11ae**

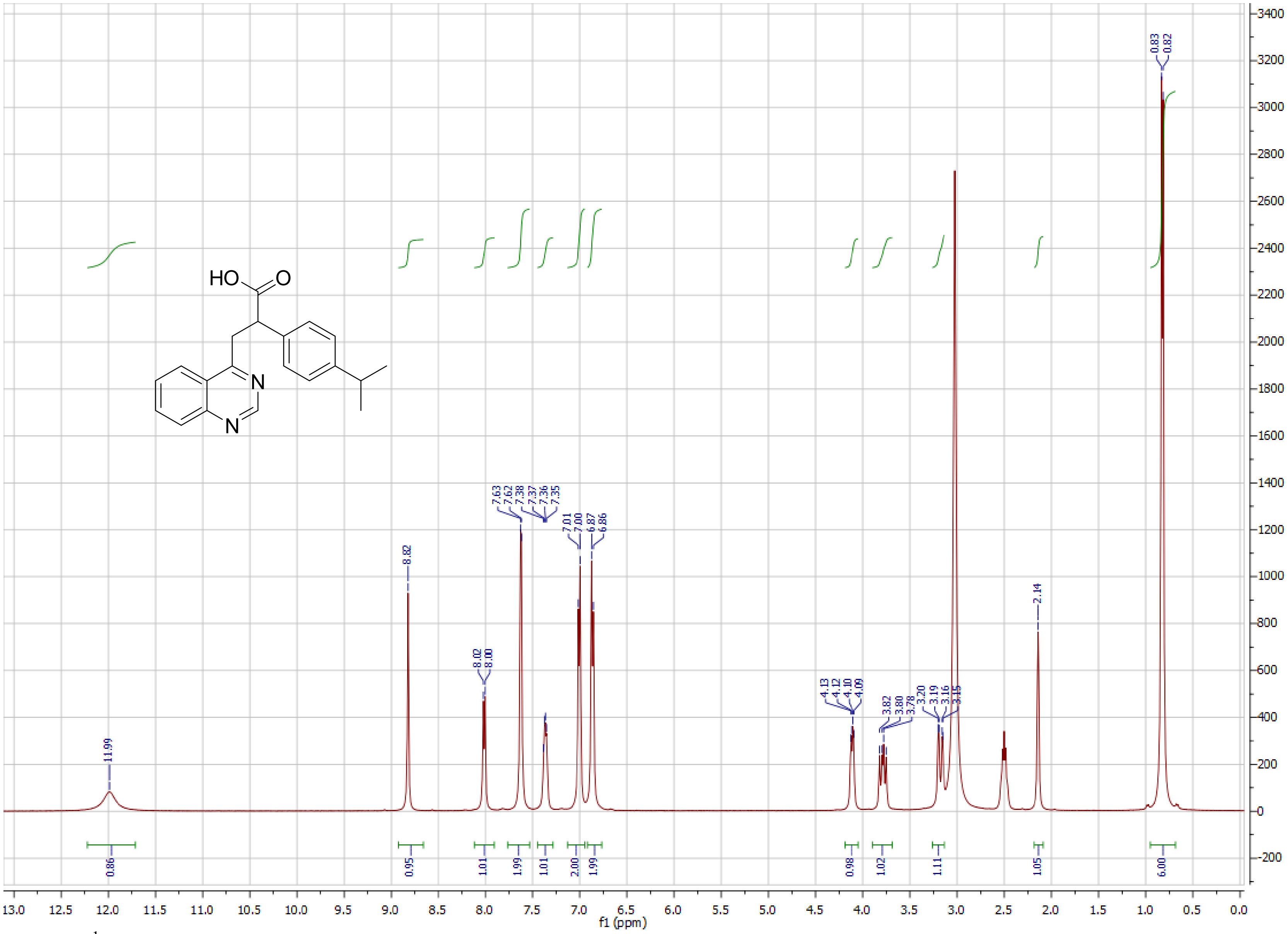

Fig S50. ^{1}H NMR spectral chart for 2-(4-isopro pylphenyl)-3-(quinazolin-4-yl)propanoic acid **11af**

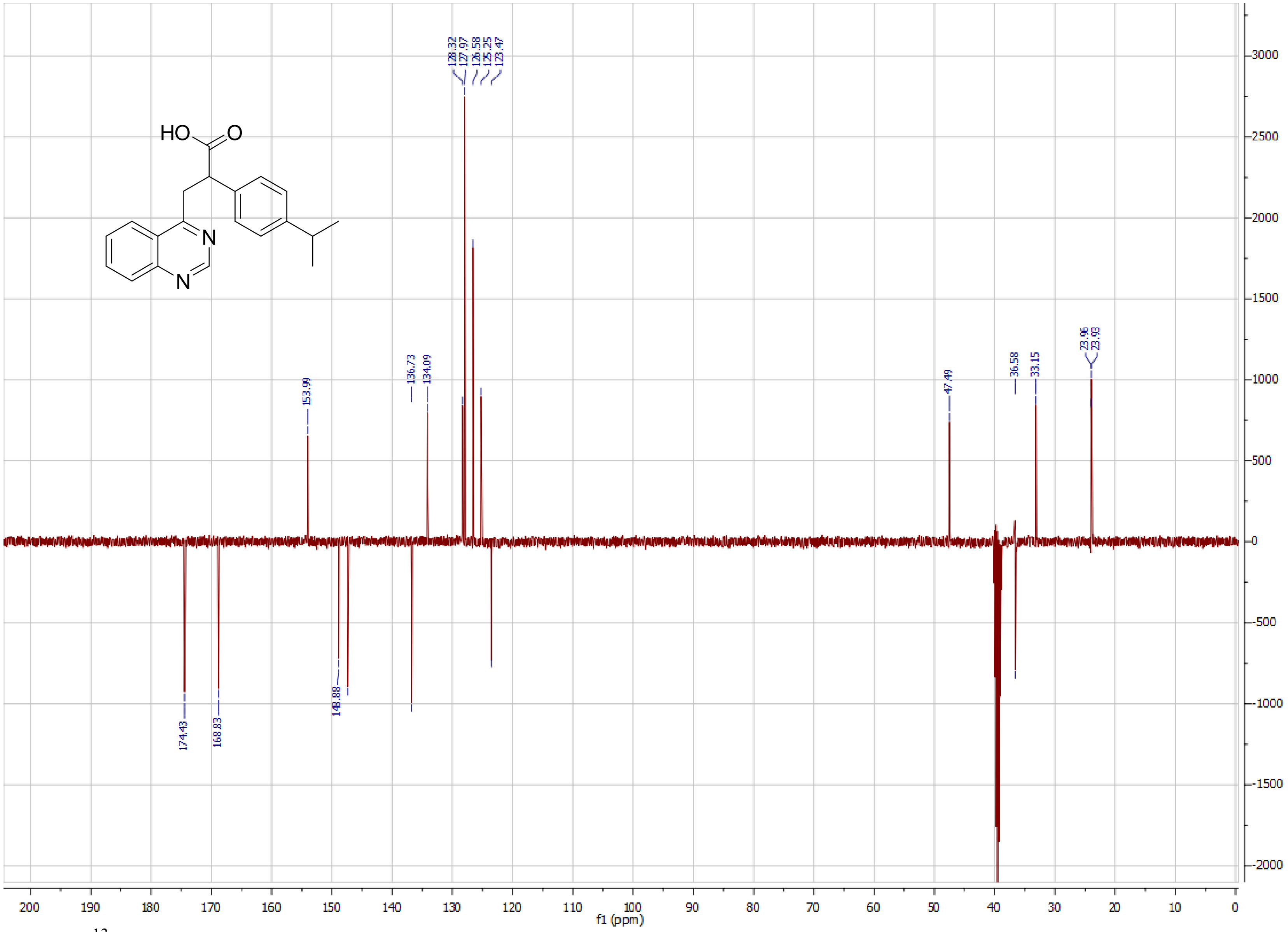

Fig S51. ^{13}C NMR spectral chart f or 2-(4-isopropylphenyl)-3-(quinazolin-4-yl)propanoic acid **11af**

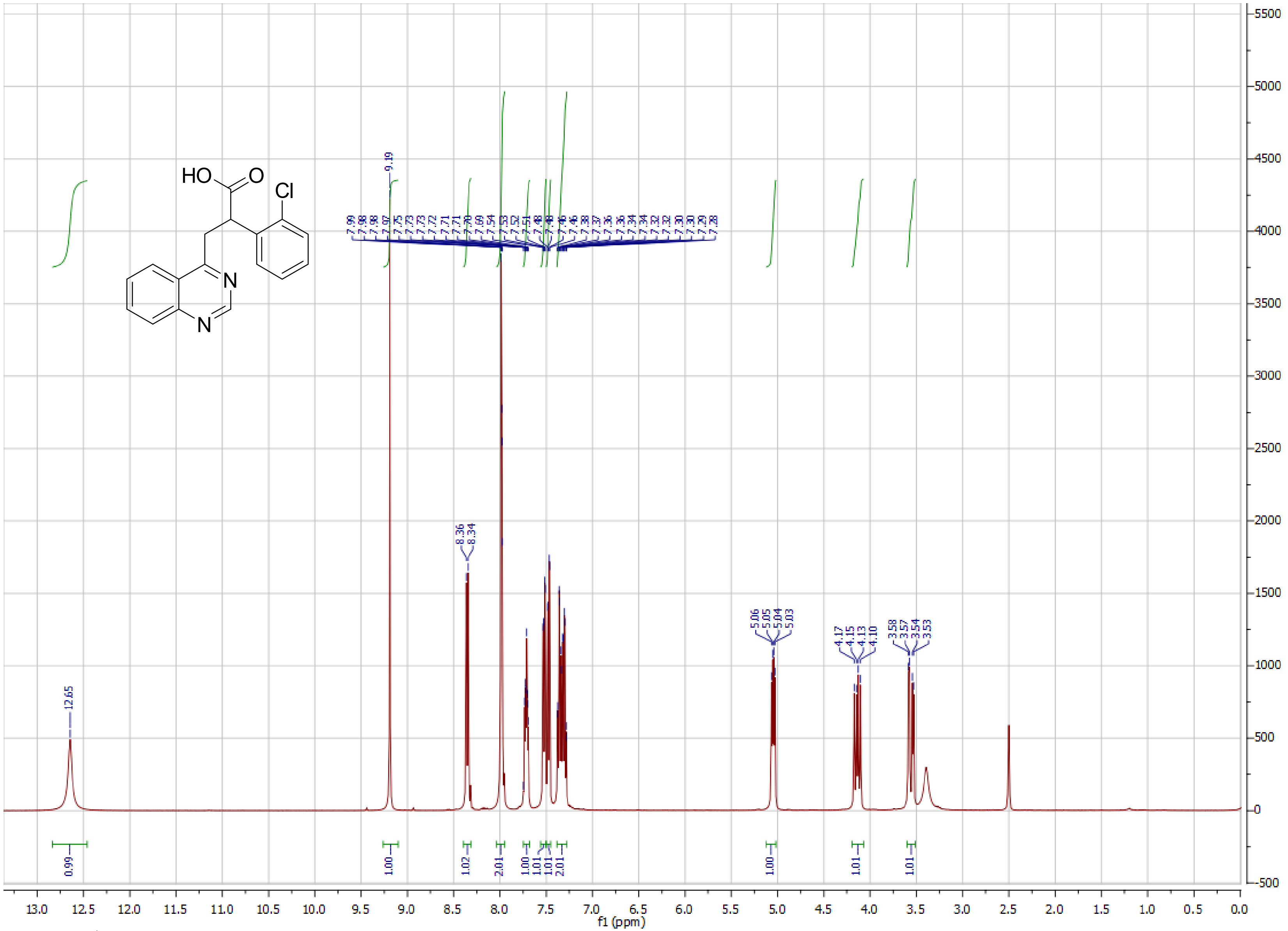

Fig S52. ^{1}H NMR spectral chart for 2-(2-c hlorophenyl)-3-(quinazolin-4-yl)propanoic acid **11ag**

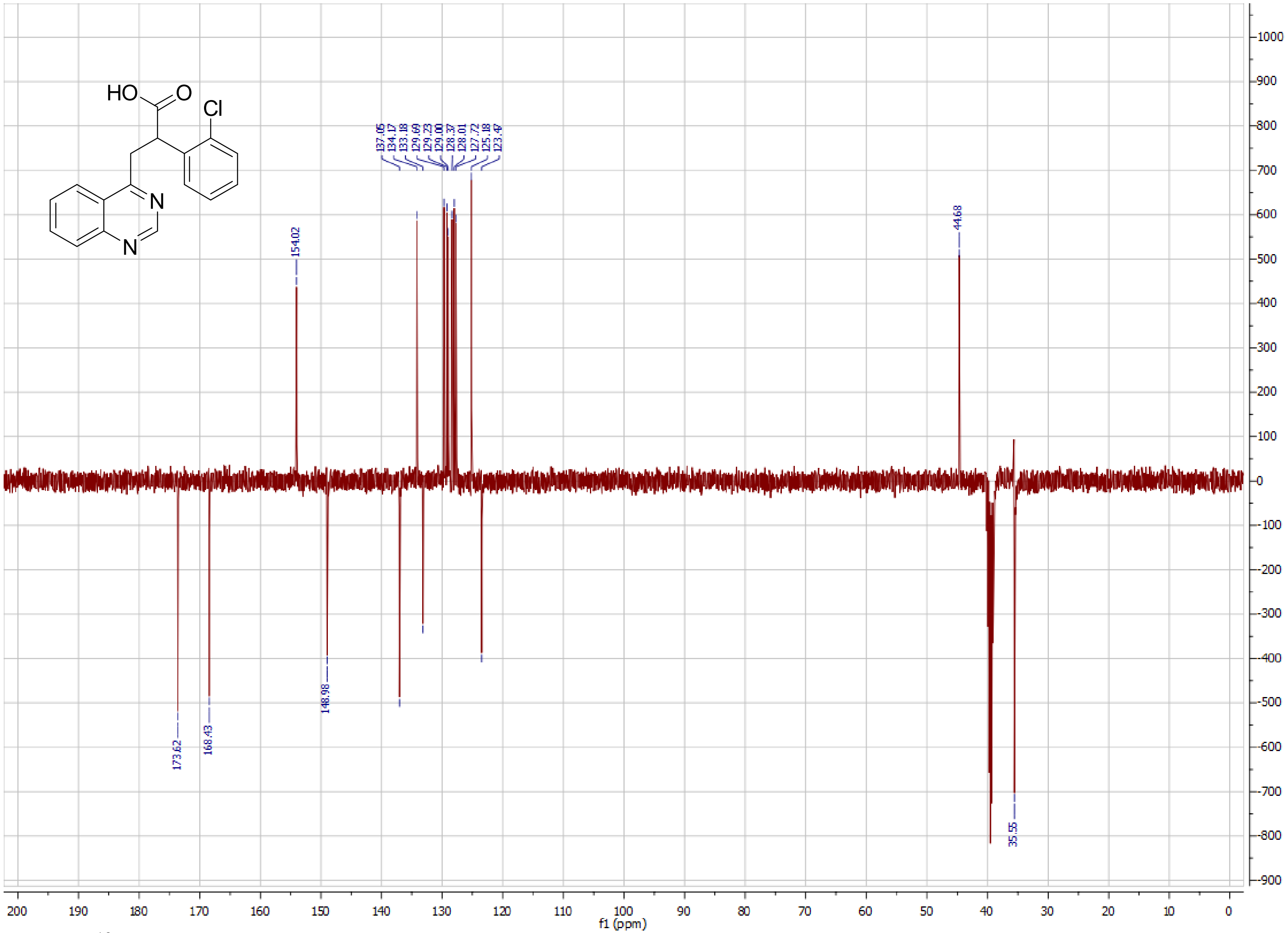

Fig S53. ^{13}C NMR spectral chart for 2-(2-chloro phenyl)-3-(quinazolin-4-yl)propanoic acid **11ag**

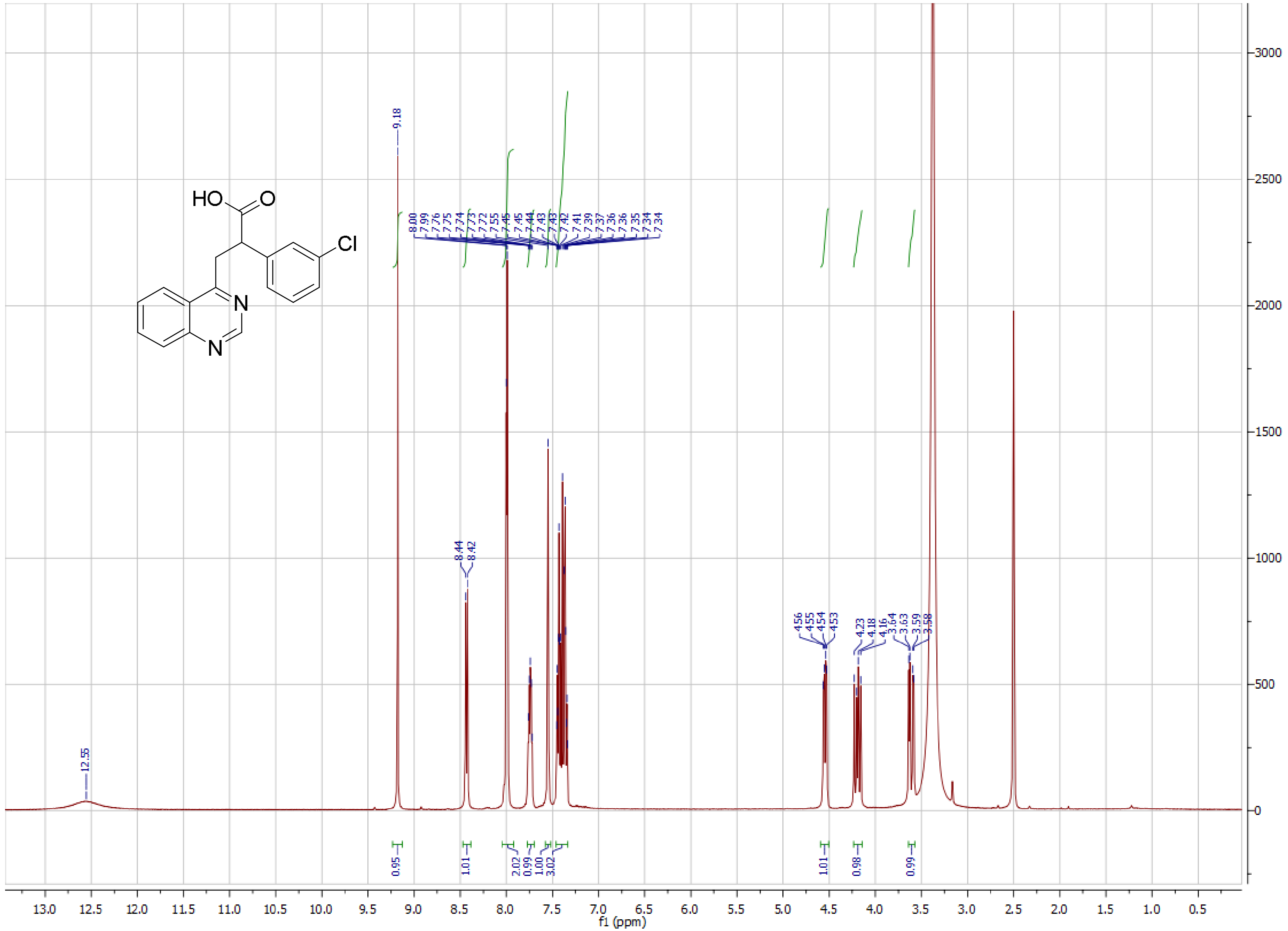

Fig S54. ^{1}H NMR spectral chart for 2-(3-chlorophenyl) -3-(quinazolin-4-yl)propanoic acid **11ah**

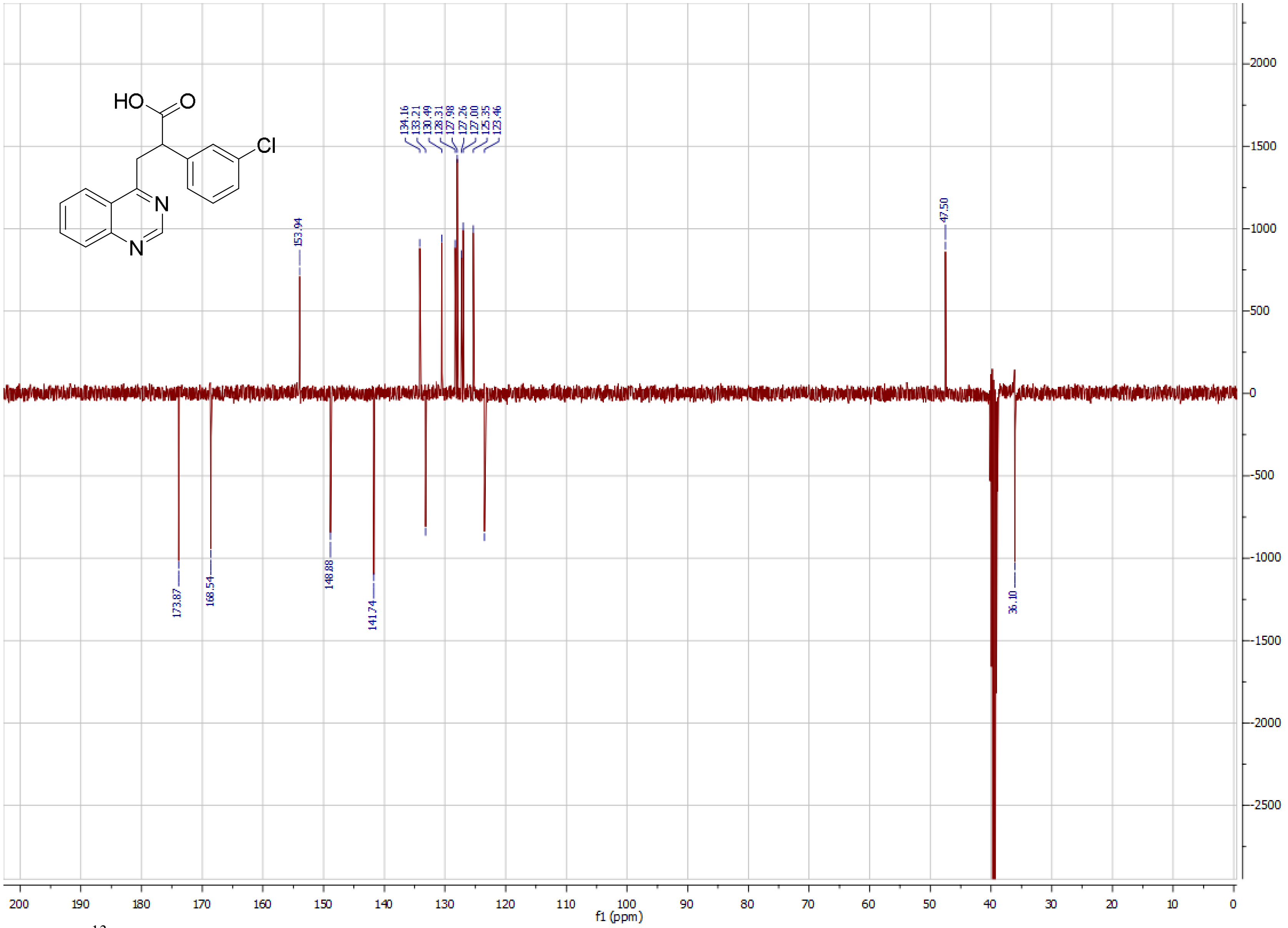

Fig S55. ^{13}C NMR spectral chart for 2-(3-chlorophenyl)-3-(quinazolin-4-yl)propanoic acid **11ah**

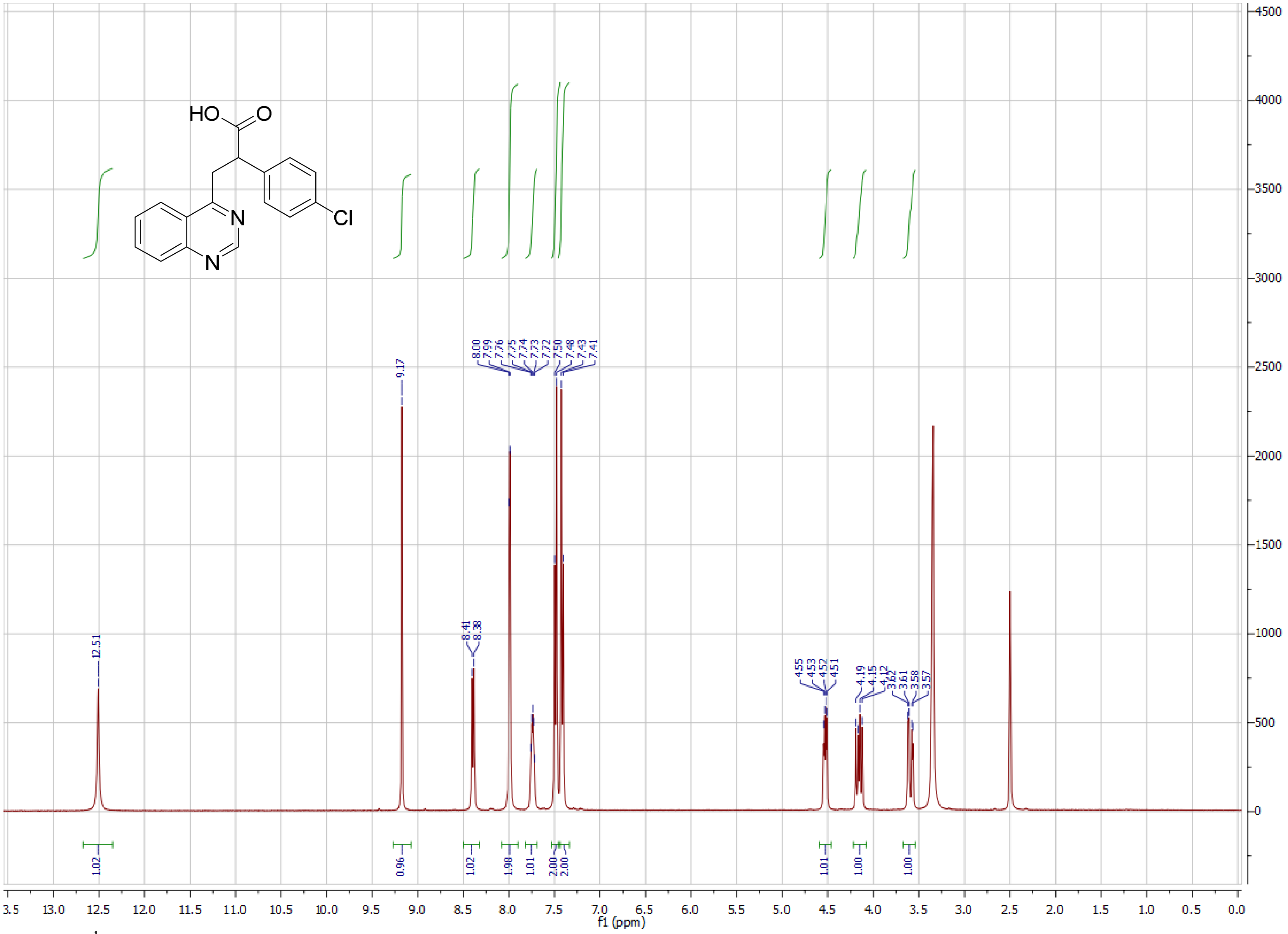

Fig S56. ^{1}H NMR spectral chart for 2-(4- chlorophenyl)-3-(quinazolin-4-yl)propanoic acid **11ai**

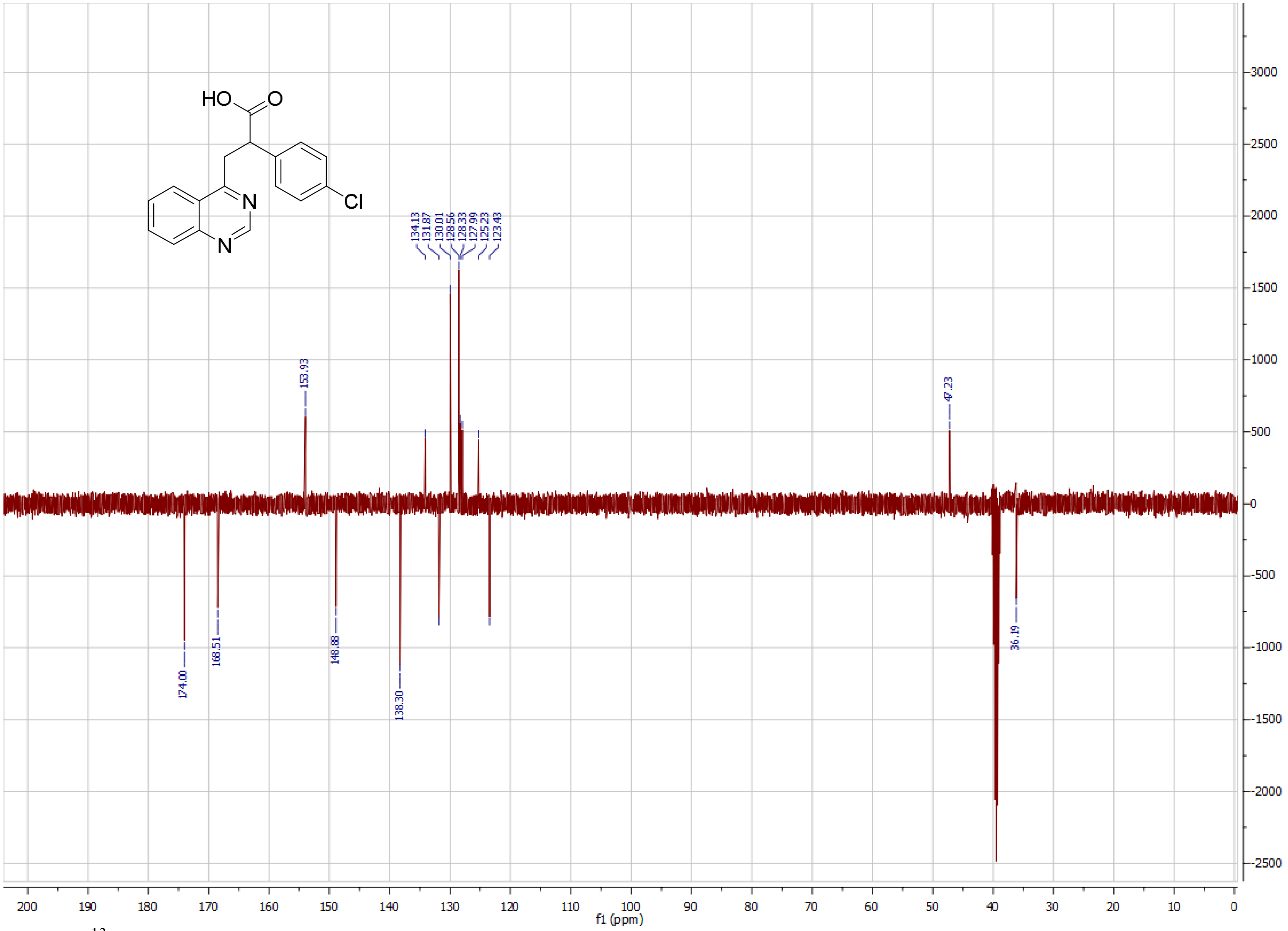

Fig S57. ^{13}C NMR spectral chart for 2-(4-chlorophenyl)-3-(quinazolin-4-yl)propanoic acid **11ai**

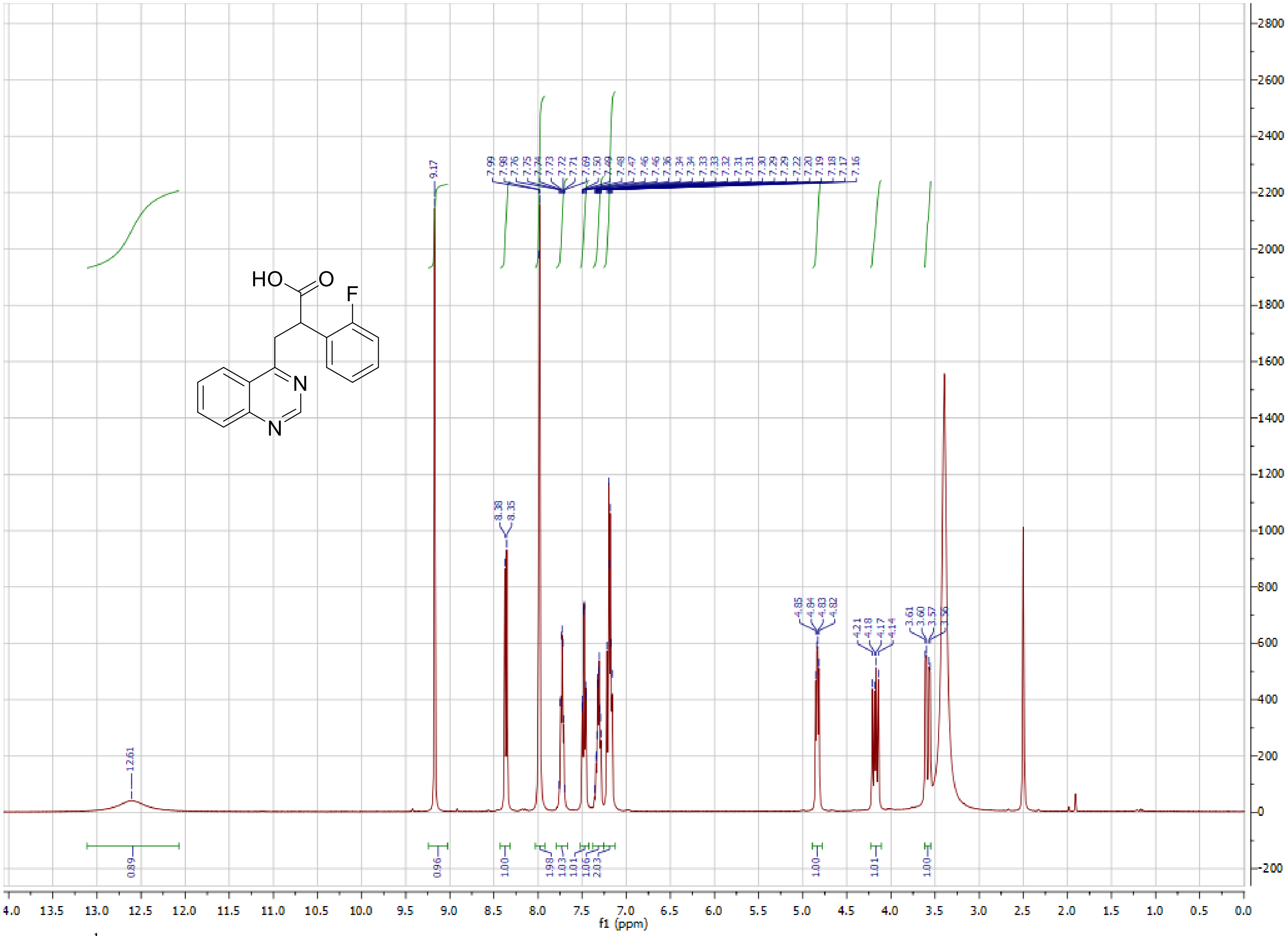

Fig S58. ^{1}H NMR spectral chart for 2-(2-fluoophenyl)-3-(quinazolin-4-yl)propanoic acid **11aj**

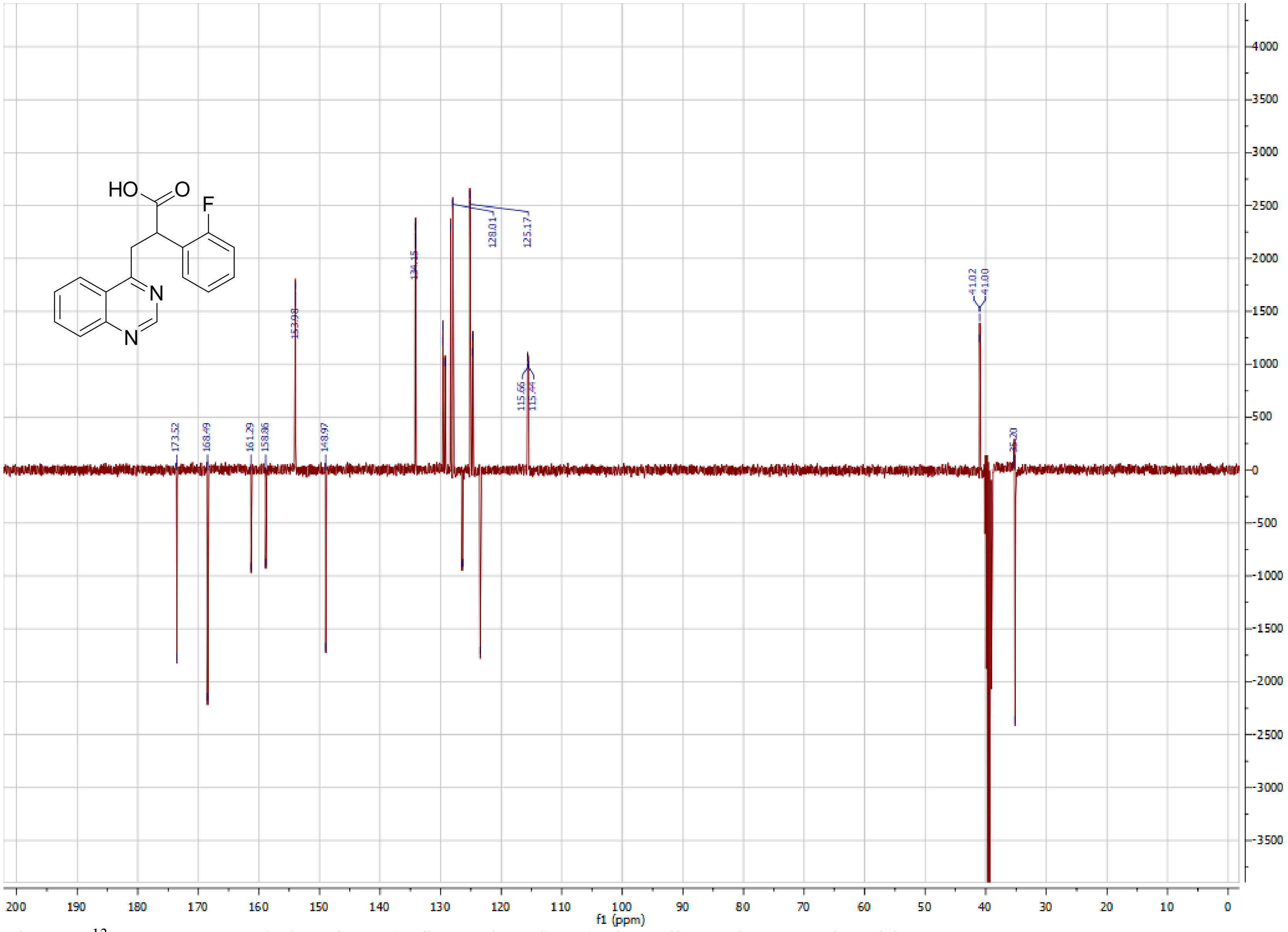

Fig S59. ^{13}C NMR spectral chart for 2-(2-fluorophenyl)-3-(quinazolin-4-yl)propanoic acid **11aj**

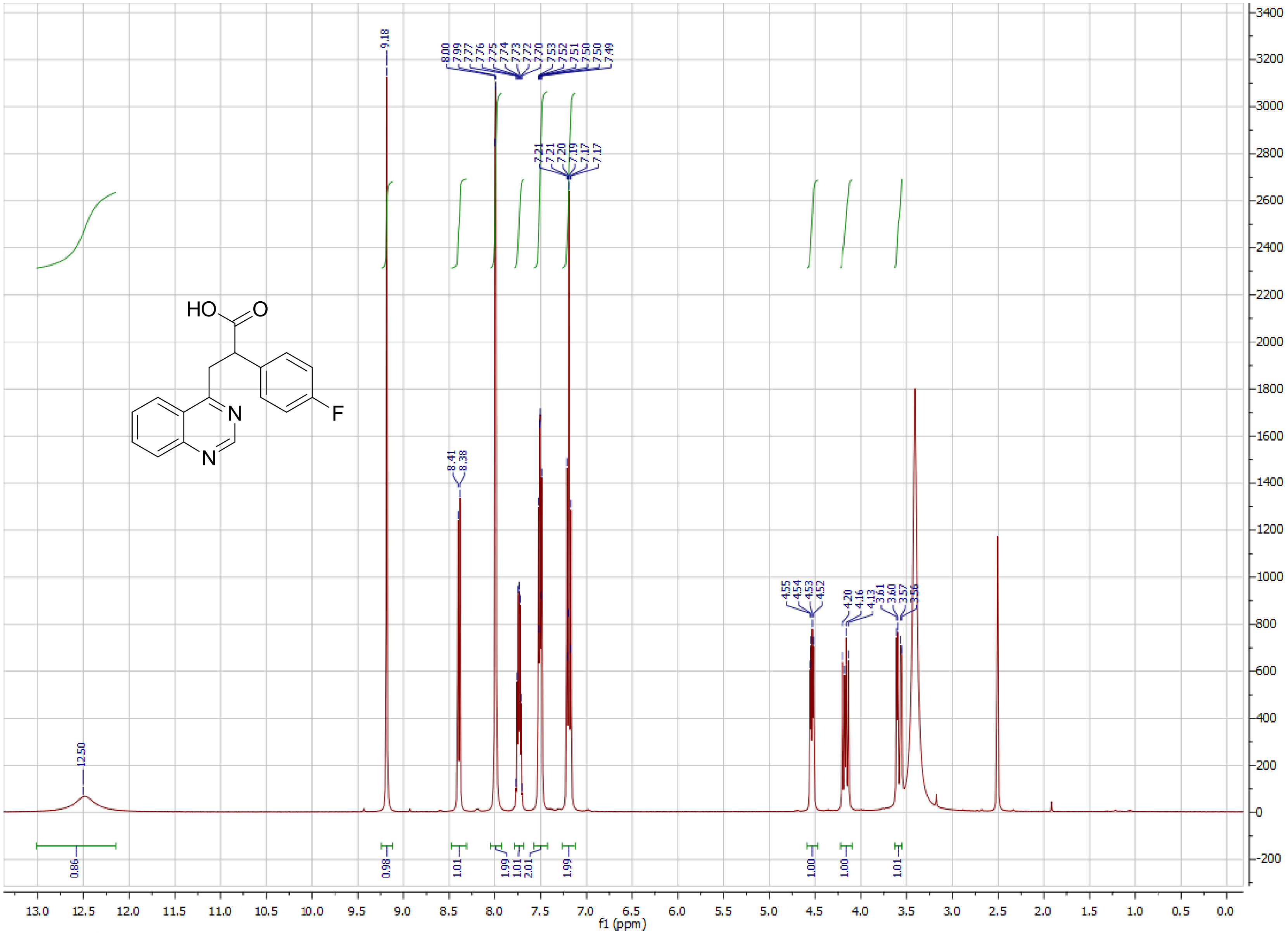

Fig S60. ^{1}H NMR spectral chart for 2-(4-fluorophenyl)-3-(quinazolin-4-yl)propanoic acid **11ak**

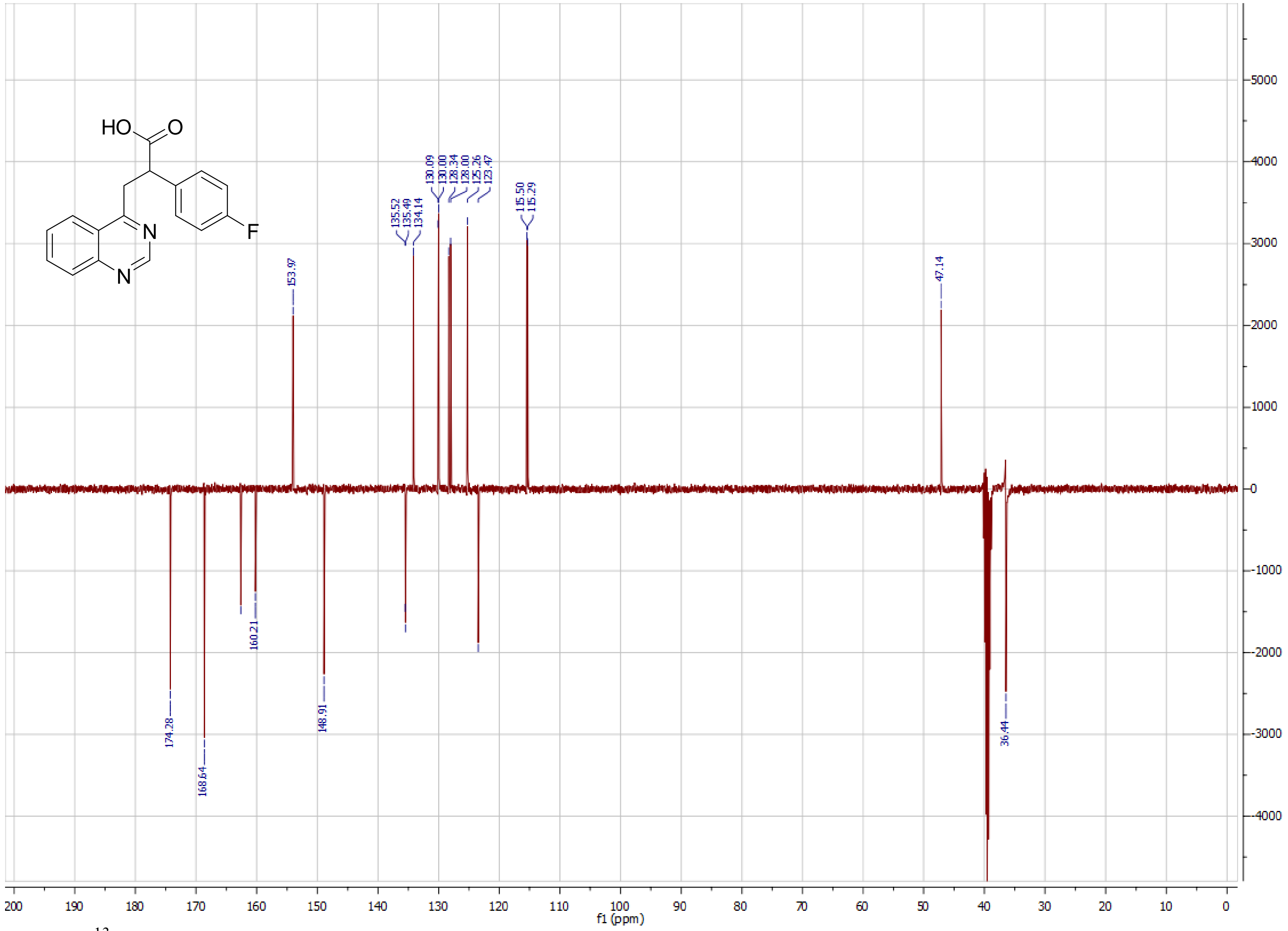

Fig S61. ^{13}C NMR spectral chart for 2-(4-fluorophenyl)-3-(quinazolin-4-yl)propanoic acid **11ak**

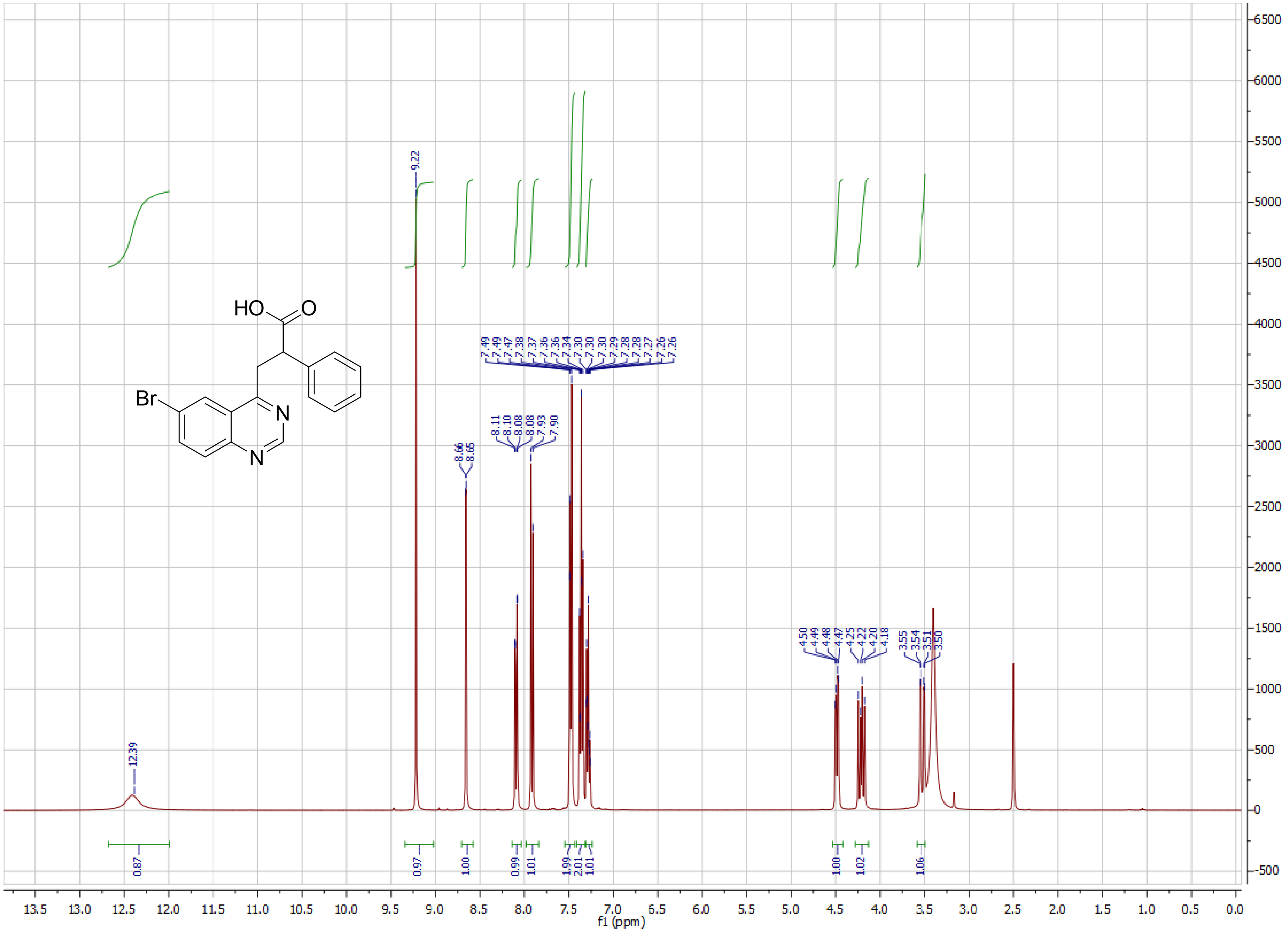

Fig S62. ^{1}H NMR spectral chart for 3-(6-bromoquinazolin-4-yl)-2-phenylpropanoic acid **11al**

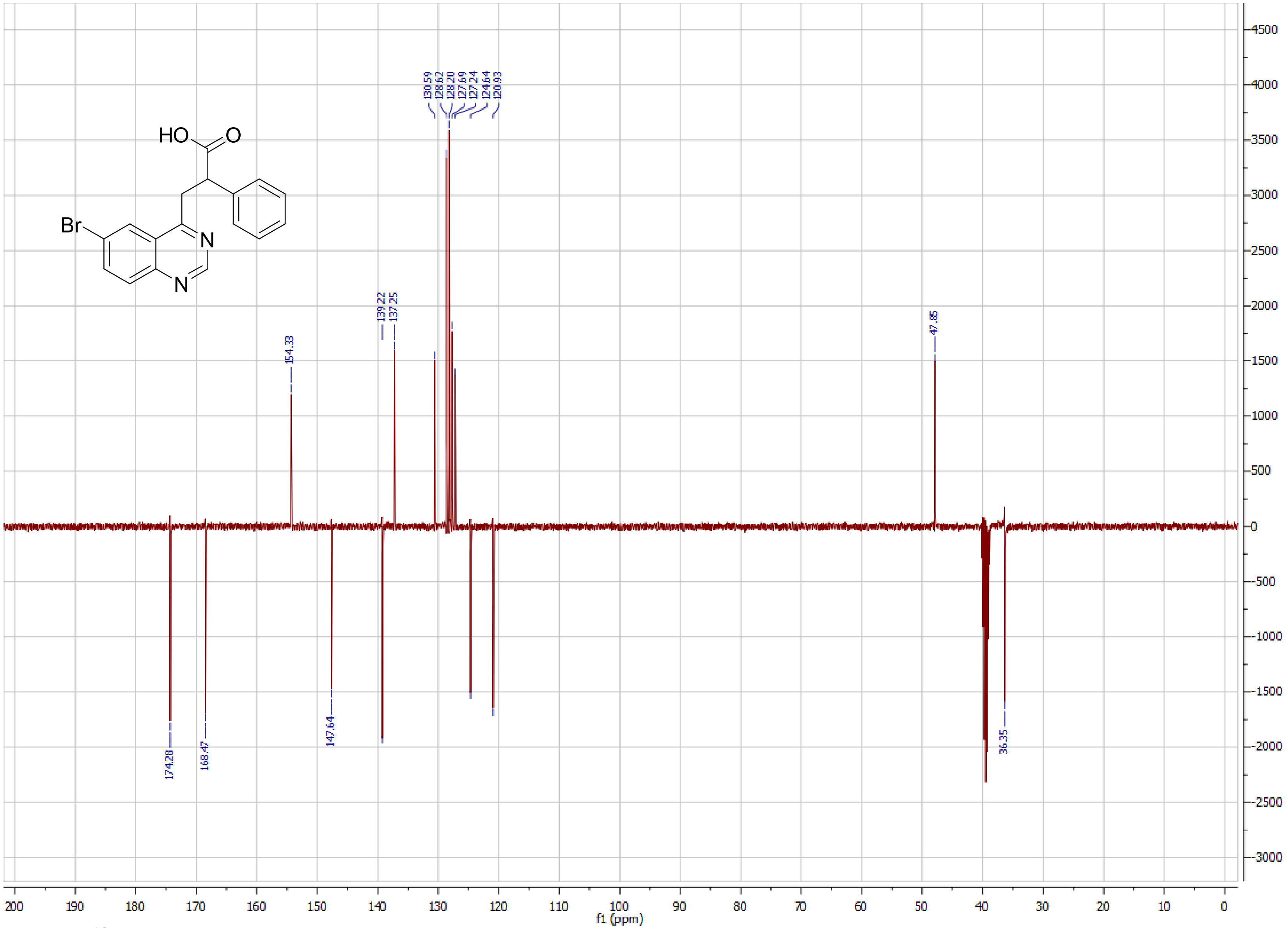

Fig S63. ^{13}C NMR spectral chart for 3-(6-bromoquinazolin-4-yl)-2-phenylpropanoic acid **11al**

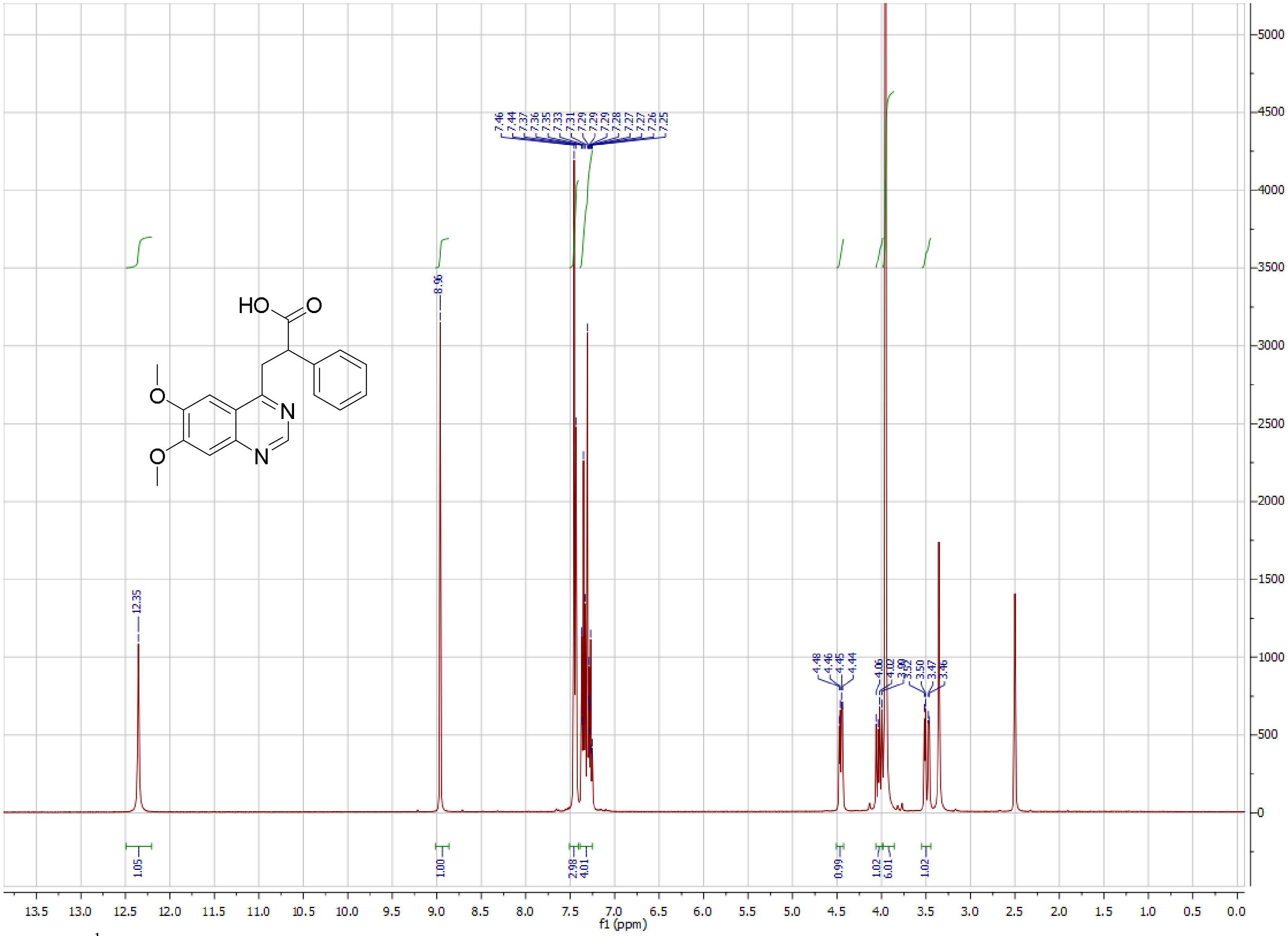

Fig S64. ^{1}H NMR spectral chart for 3-(6,7-dimethoxyquinazolin-4-yl)-2-phenylpropanoic acid **11am**

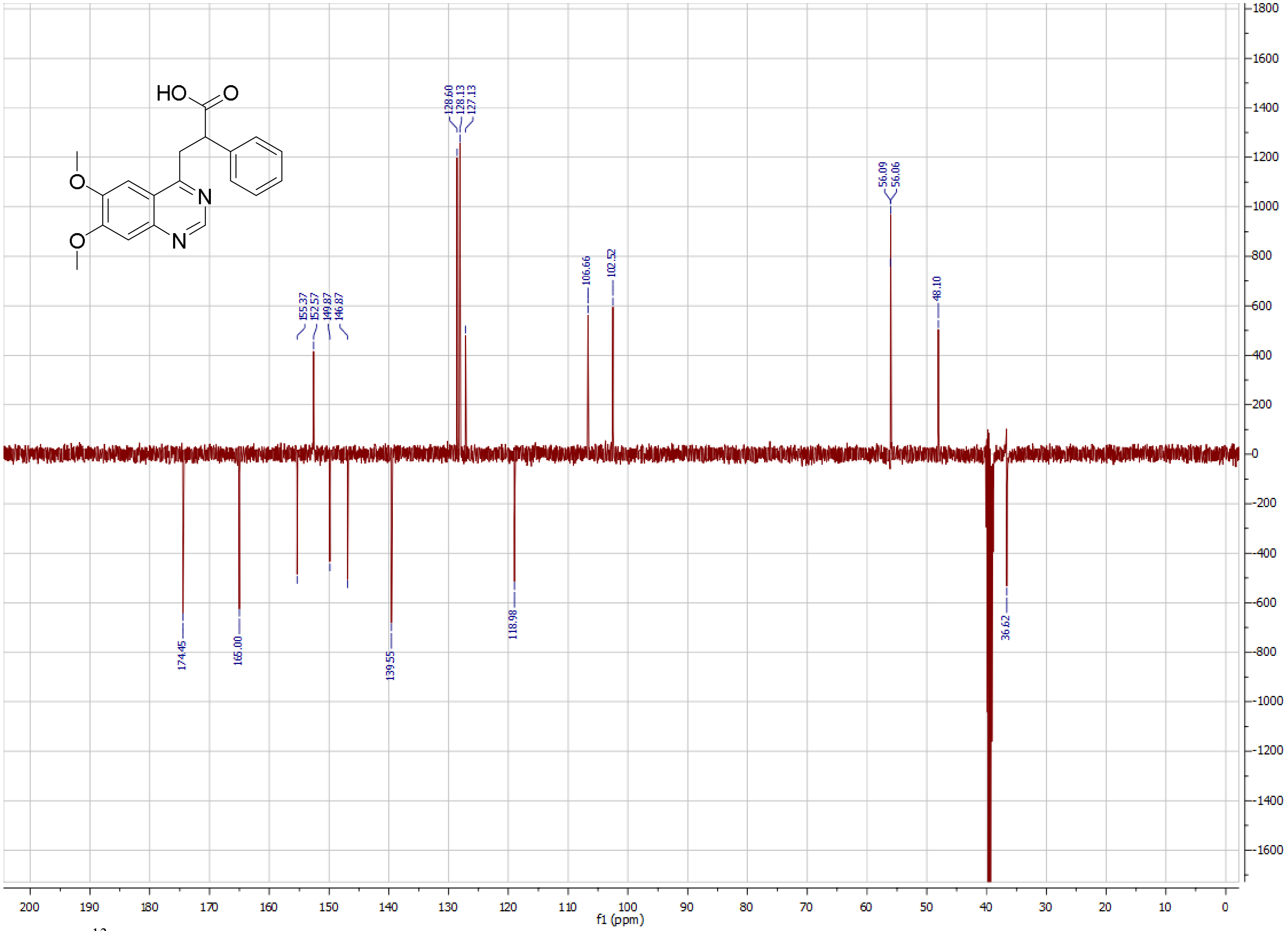

Fig S65. ^{13}C NMR spectral chart for 3-(6,7-dimethoxyquinazolin-4-yl)-2-phenylpropanoic acid **11am**

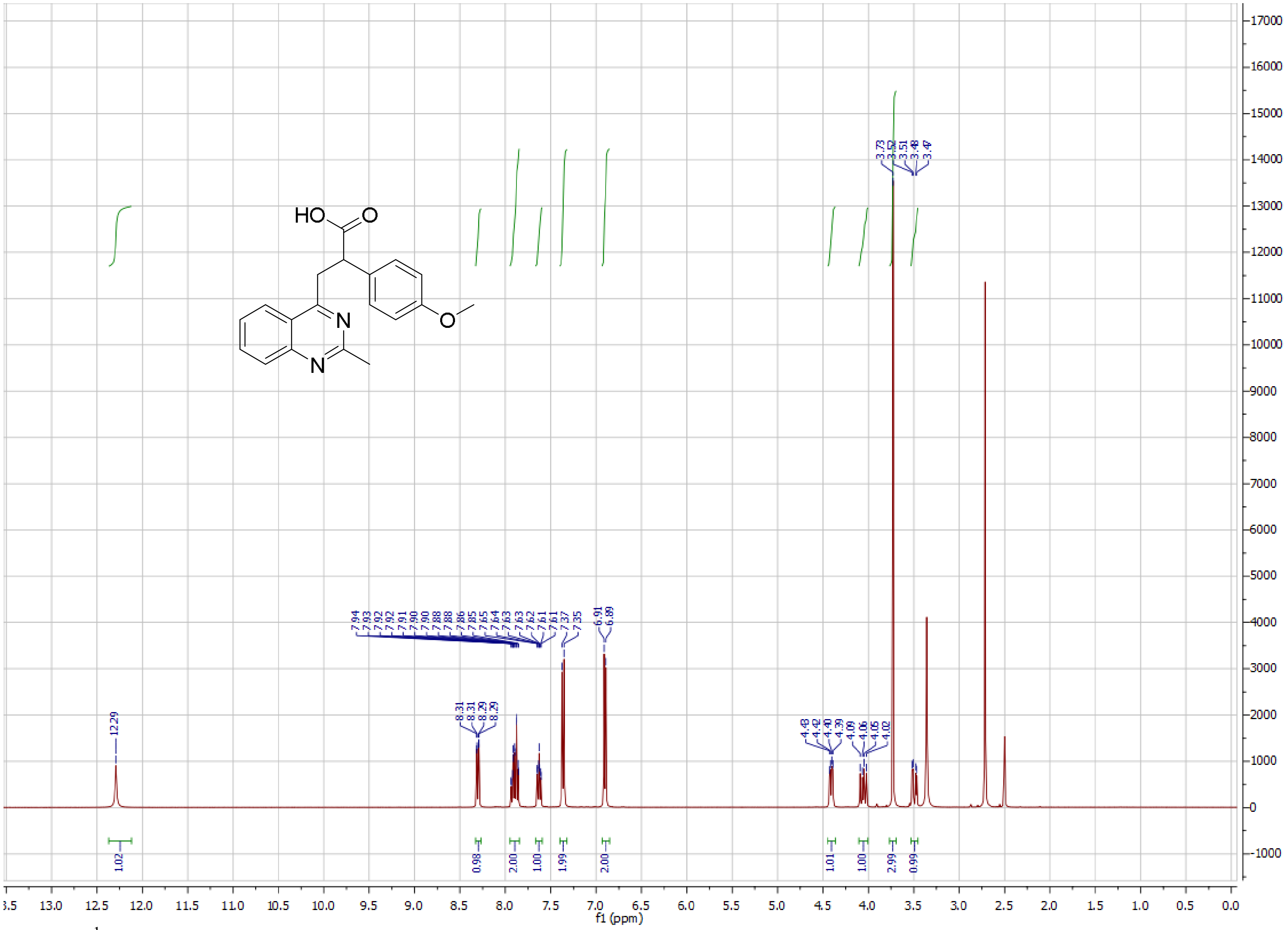

Fig S66. ^{1}H NMR spectral chart for 2-(4-methoxyphenyl) -3-(2-methylquinazolin-4-yl)propanoic acid **11ba**

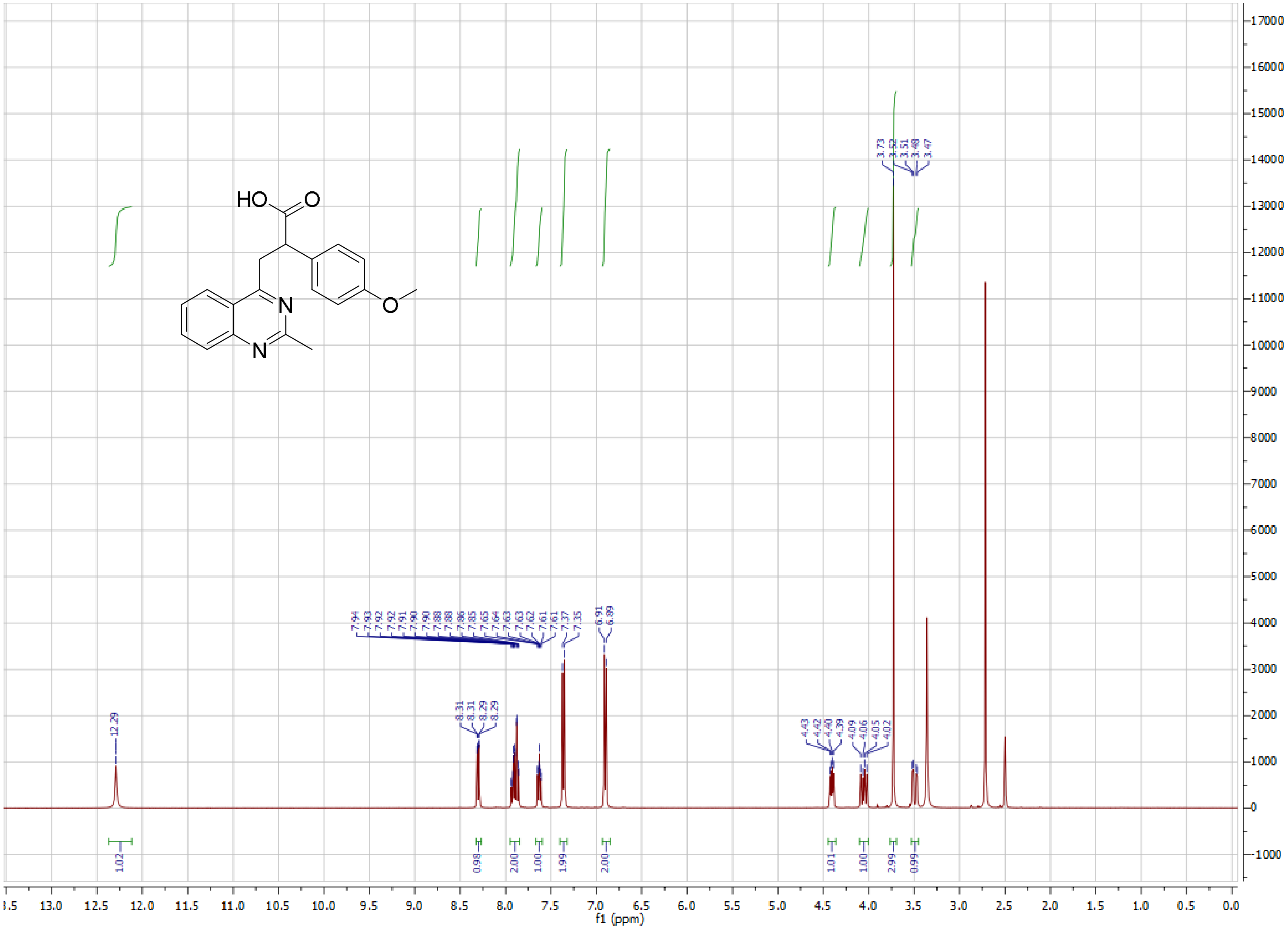

Fig S67. ^{13}C NMR spectral chart for 2-(4-methoxyphenyl)-3-(2-methylquinazolin-4-yl)propanoic acid **11ba**

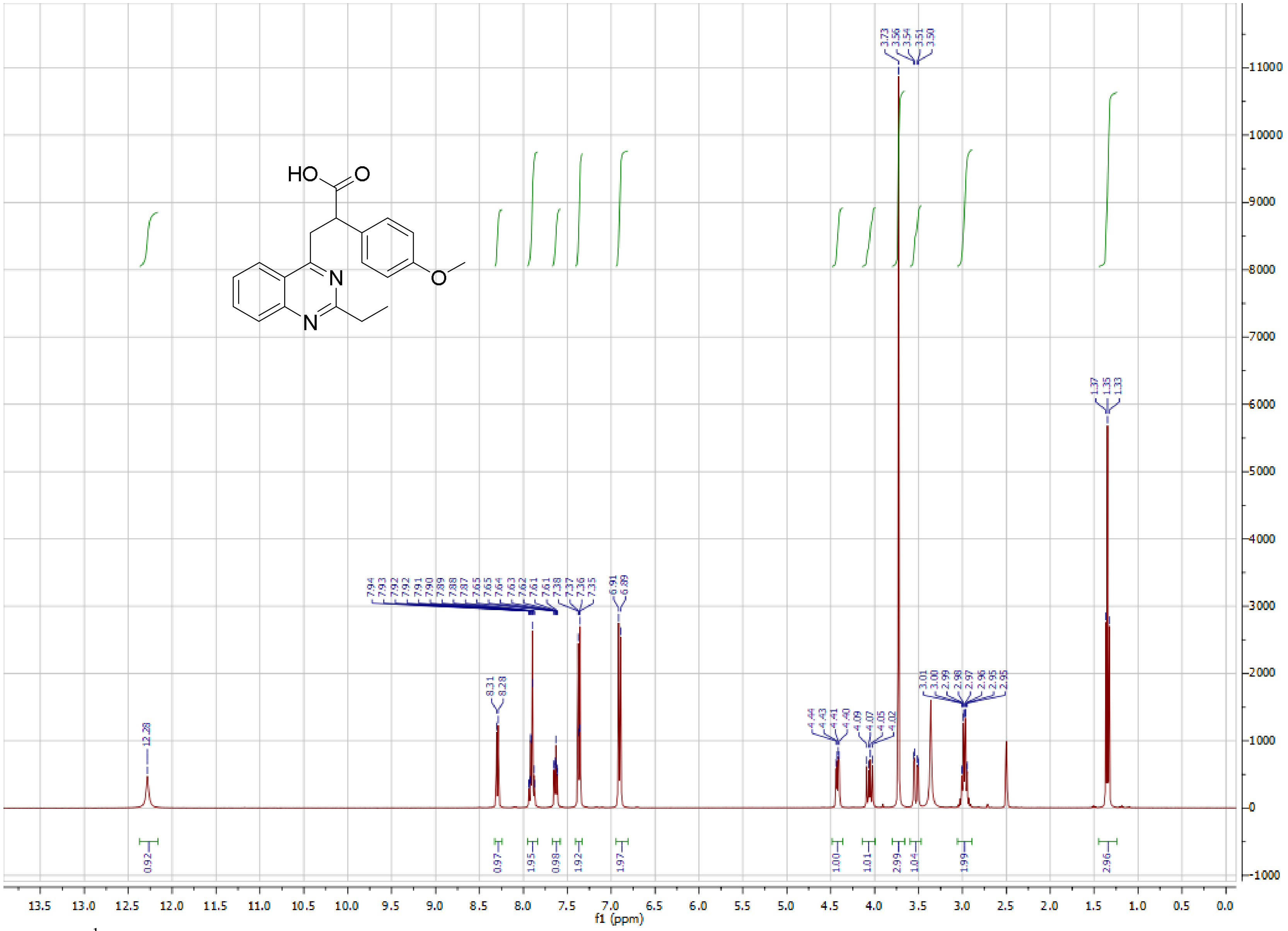

Fig S68. ^{1}H NMR spectral chart for 3-(2-ethylquinazolin-4-yl)-2-(4-methoxyphenyl)propanoic acid **11ca**

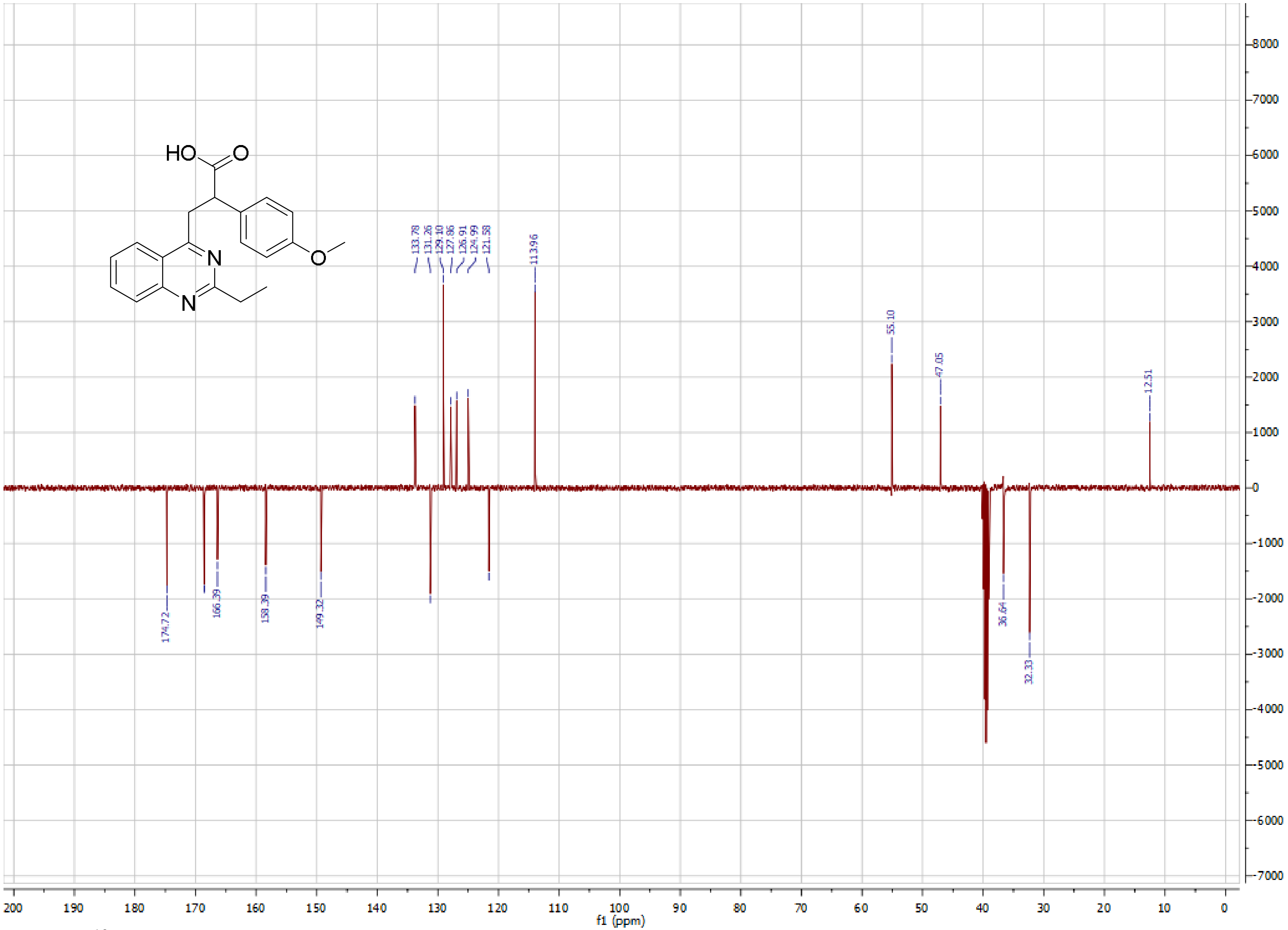

Fig S69. ^{13}C NMR spectral chart for 3-(2-ethylquinazolin-4-yl)-2-(4-methoxyphenyl)propanoic acid **11ca**

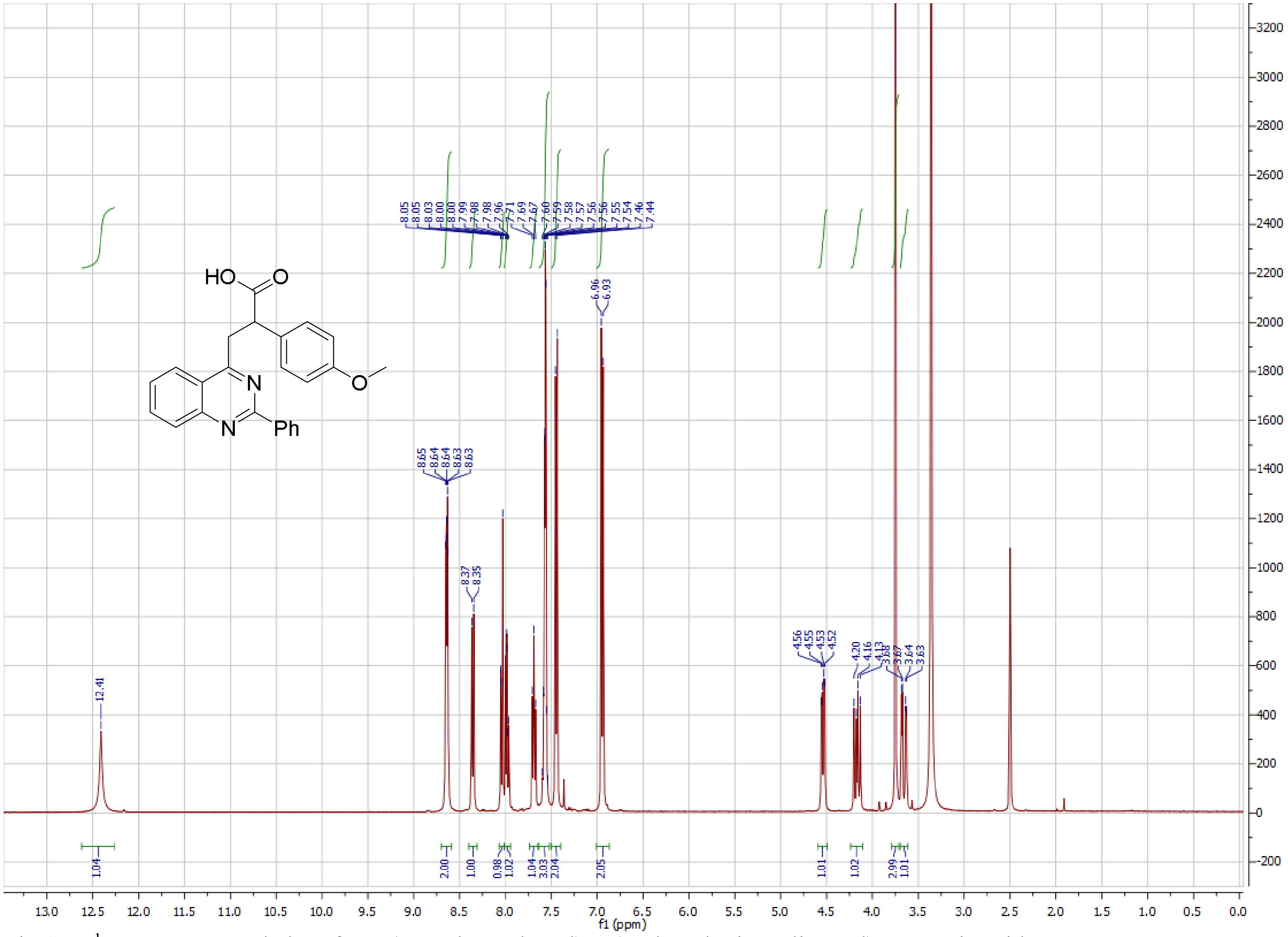

Fig S70. ^{1}H NMR spectral chart for 2-(4-methoxyphenyl)-3-(2-phenylquinazolin-4-yl)propanoic acid **11da**

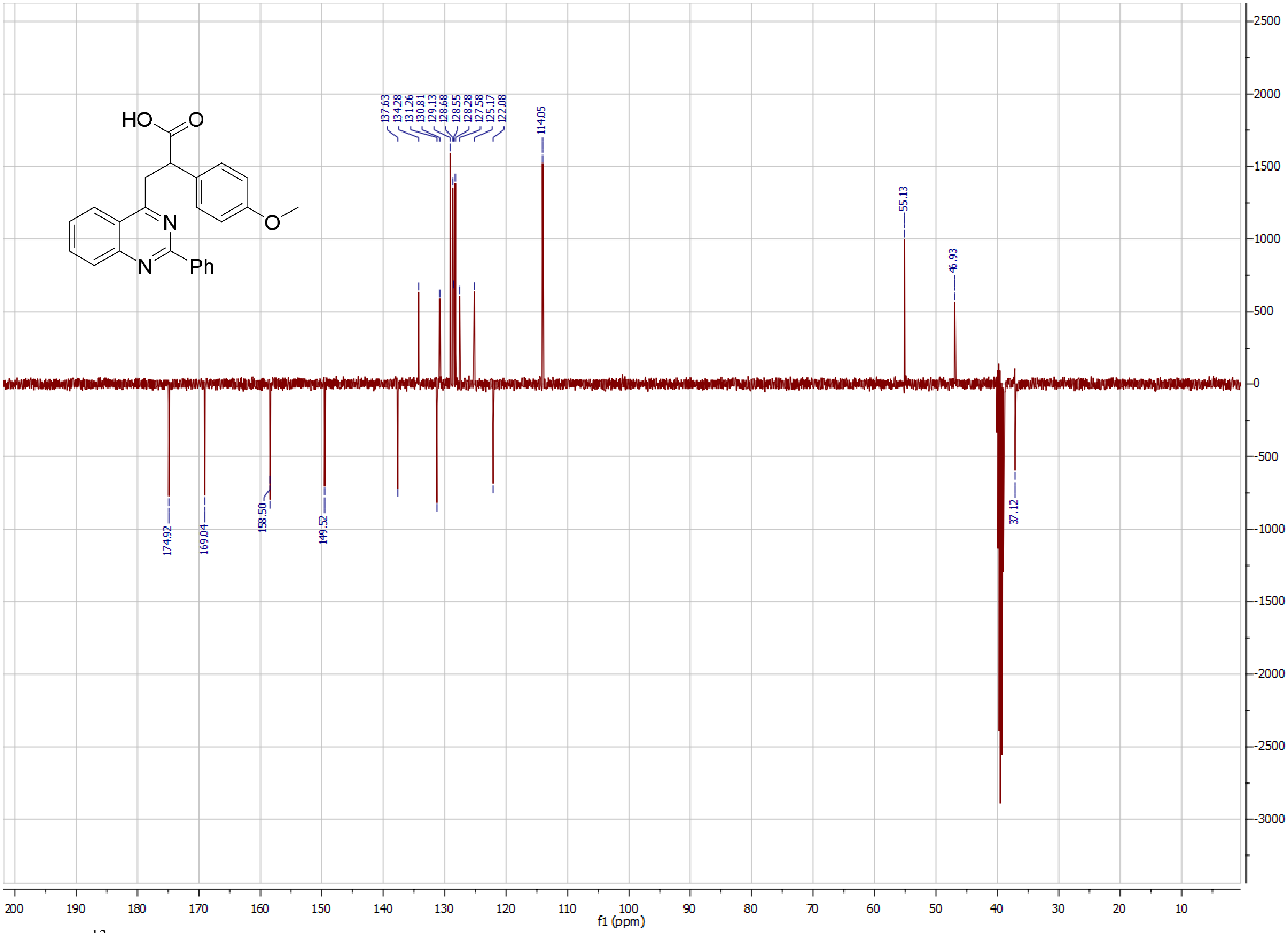

Fig S71. ^{13}C NMR spectral chart for 2-(4-methoxyphenyl)-3-(2-phenylquinazolin-4-yl)propanoic acid **11da**

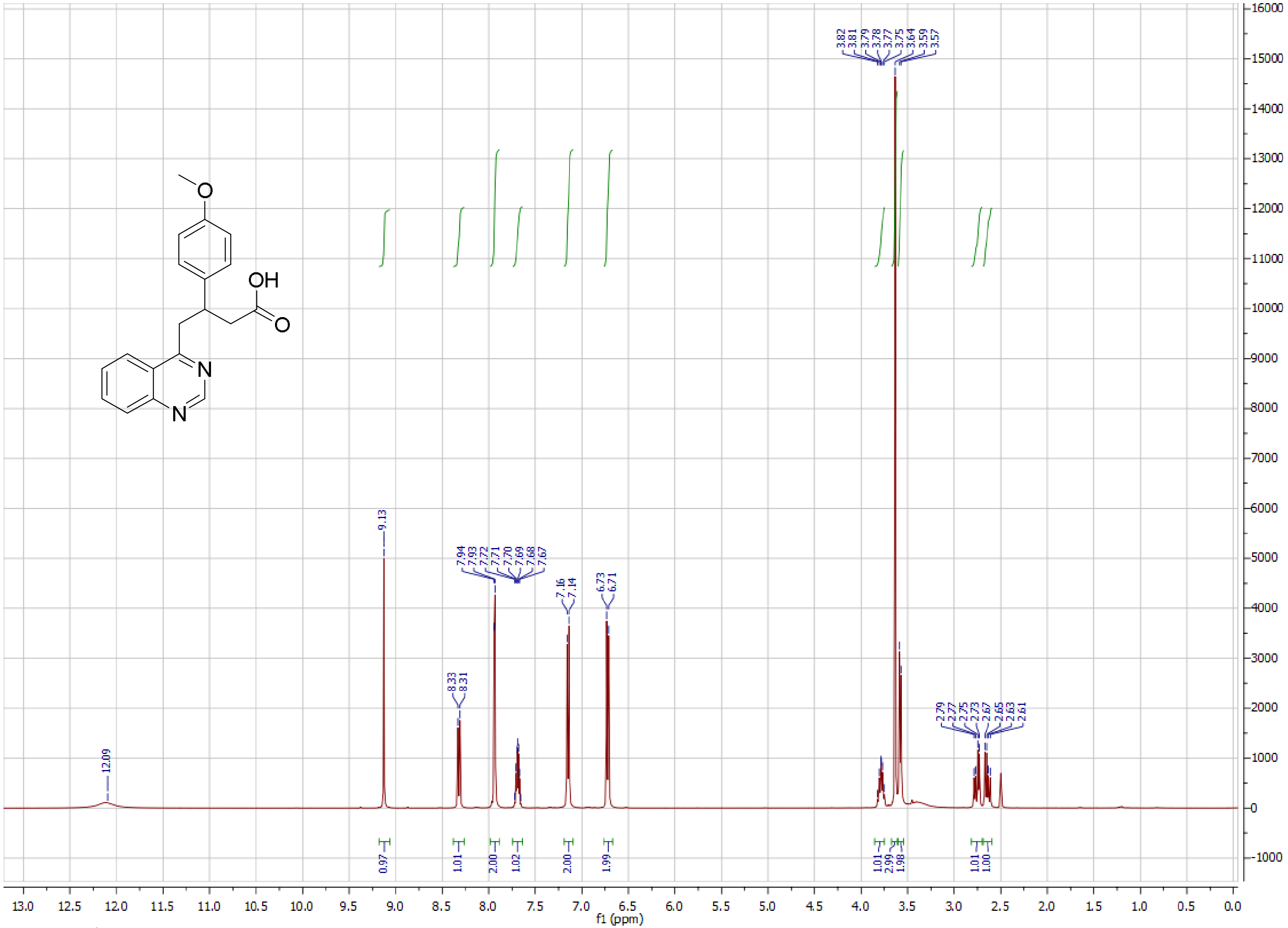

Fig S72. ^{1}H NMR spectral chart for 3-(4-methoxyphenyl)-4-(quinazolin-4-yl)butanoic acid **23aa**

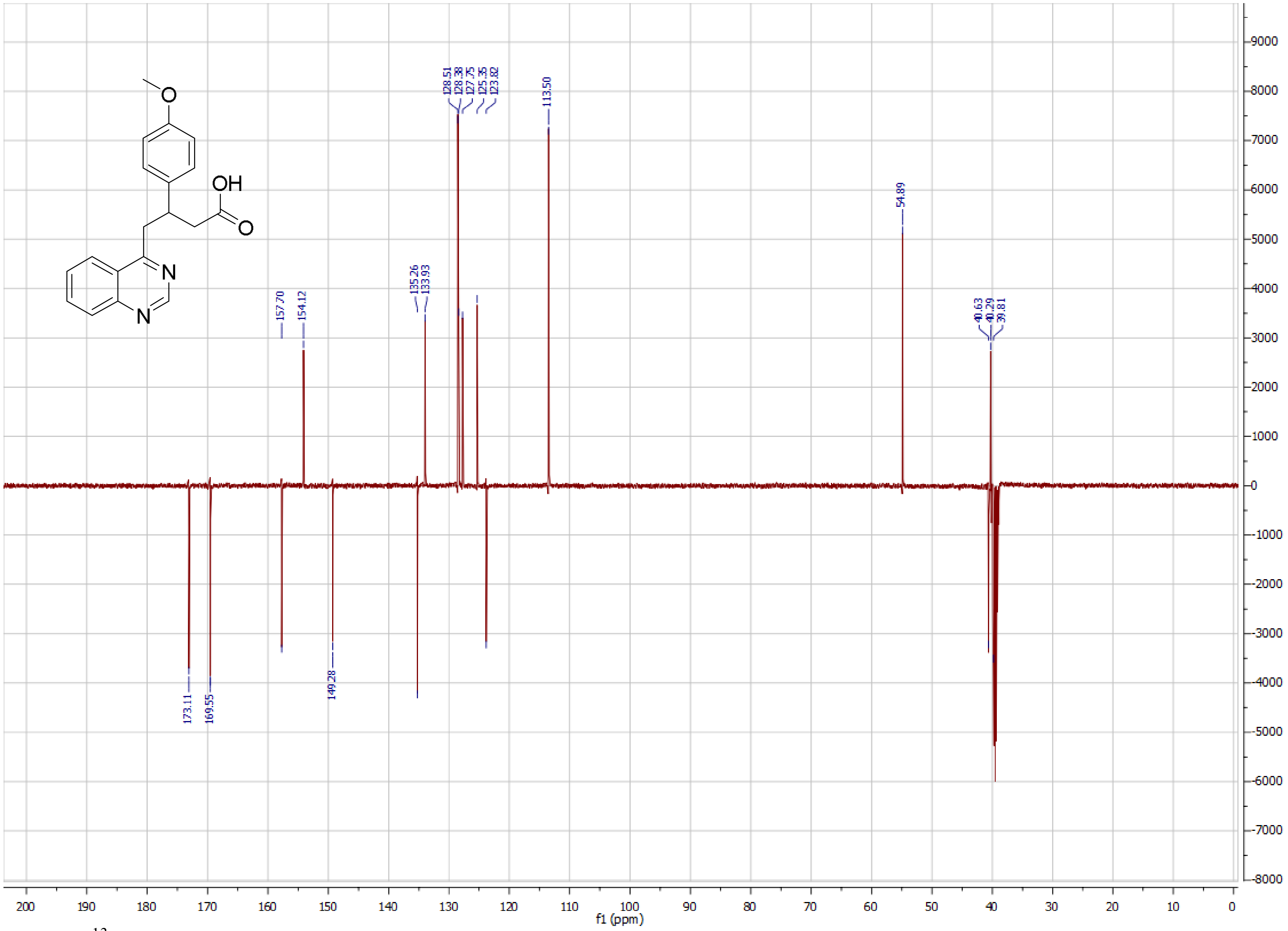

Fig S73. ^{13}C NMR spectral chart for 3-(4-methoxyphenyl)-4-(quinazolin-4-yl)butanoic acid **23aa**

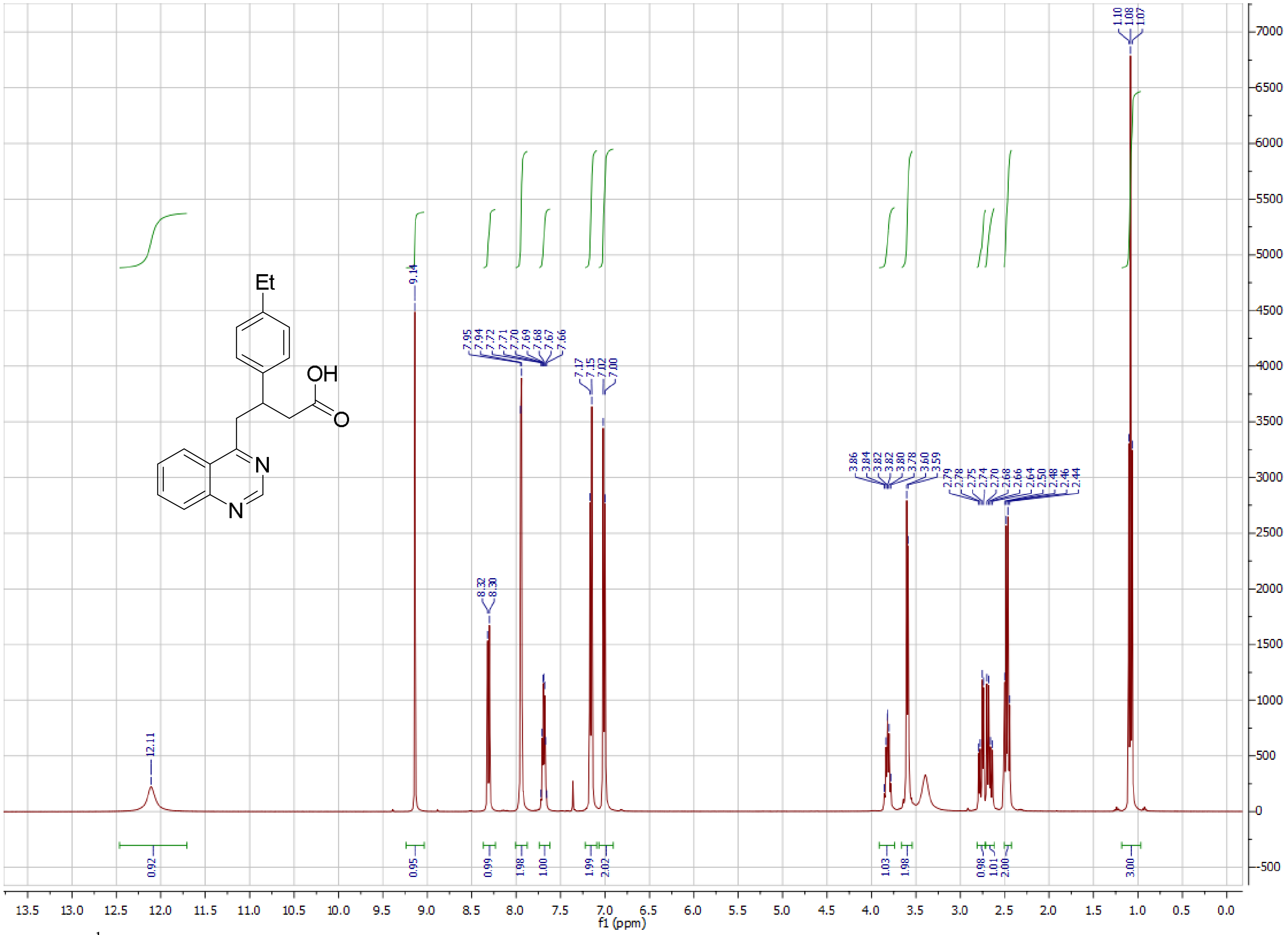

Fig S74. ^{1}H NMR spectral chart for 3-(4-ethylphenyl)-4-(quinazolin-4-yl)butanoic acid **23ae**

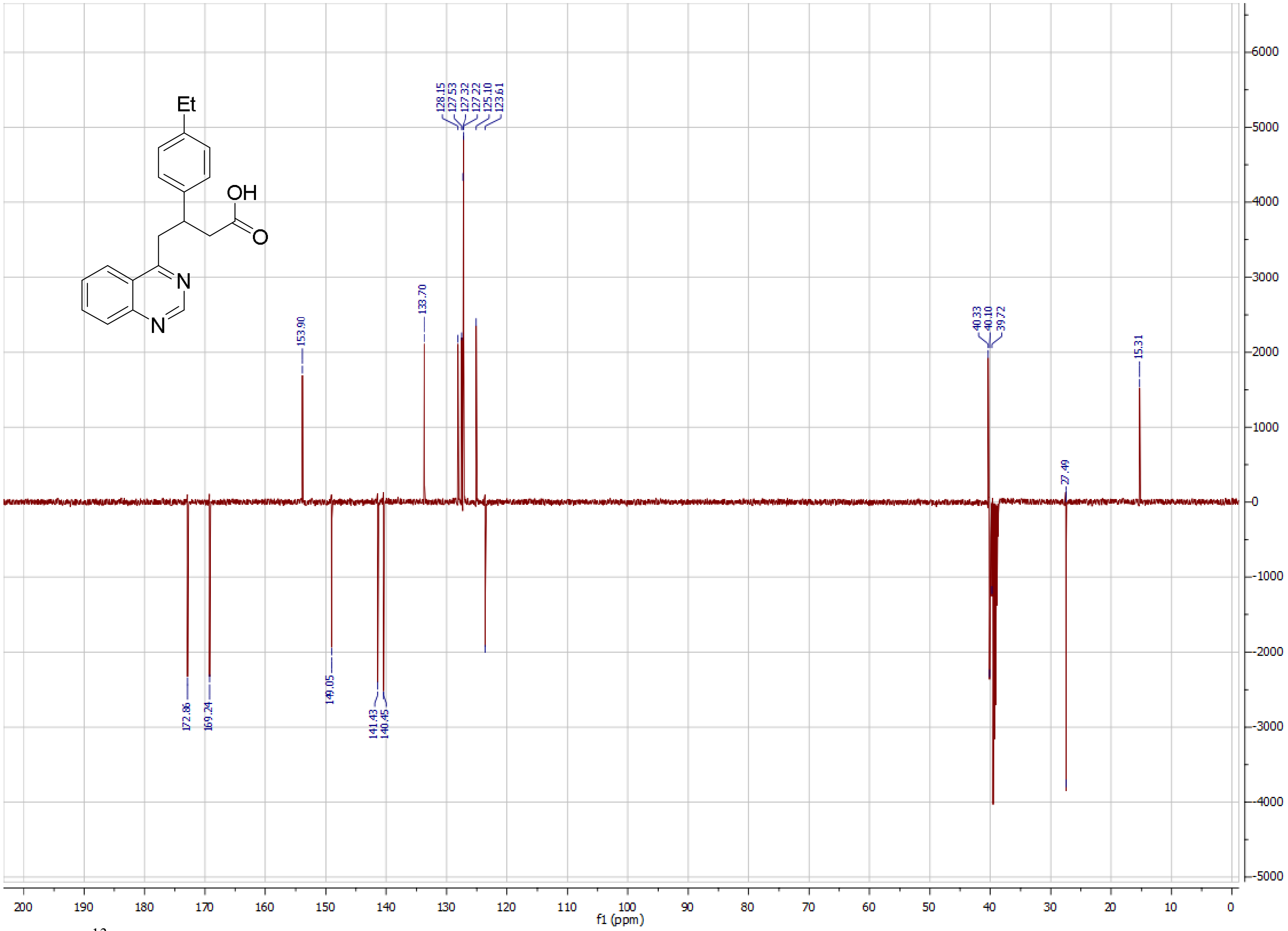

Fig S75. ^{13}C NMR spectral chart for 3-(4-ethylphenyl)-4-(quinazolin-4-yl)butanoic acid **23ae**

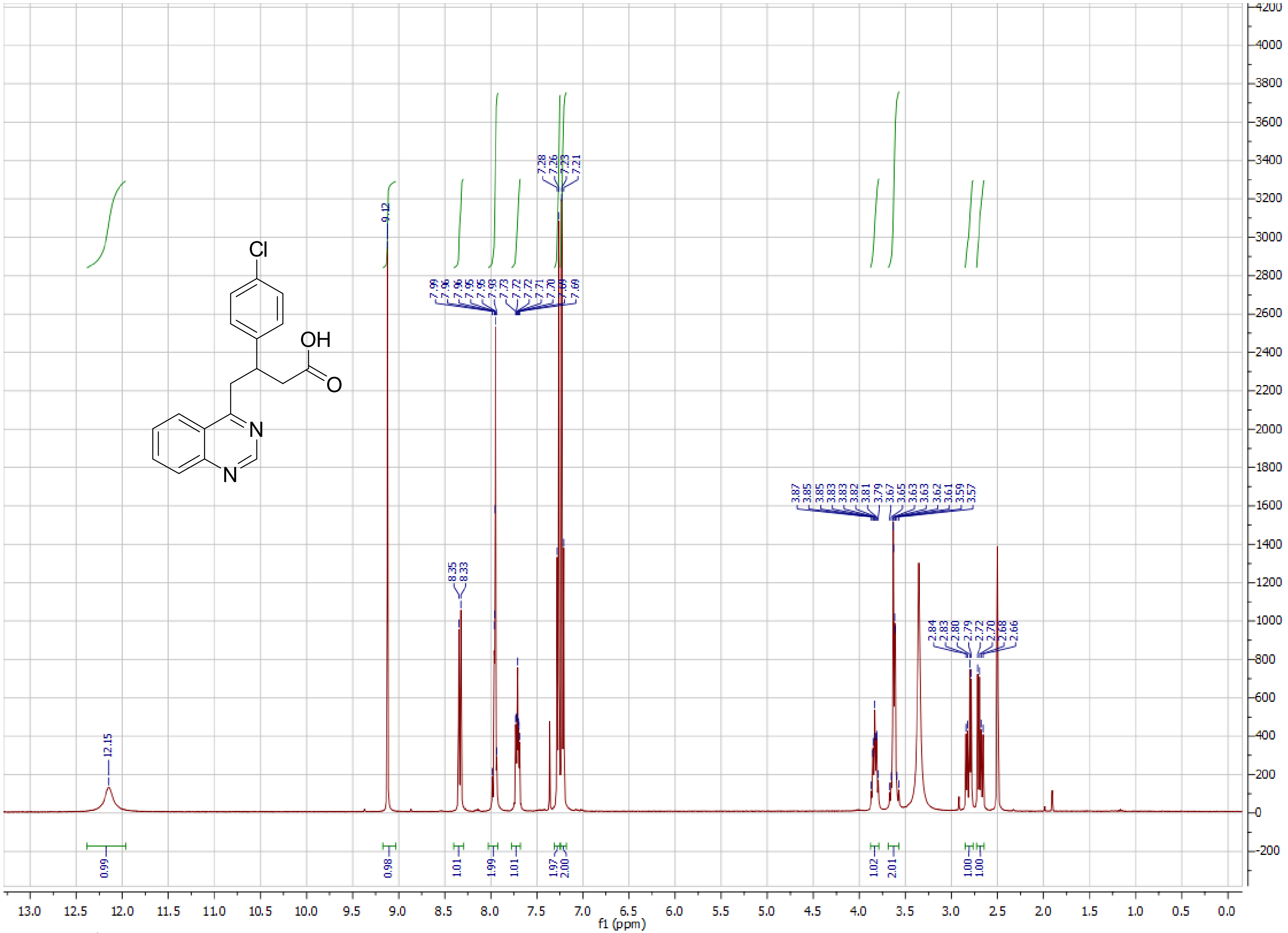

Fig S76. ^{1}H NMR spectral chart for 3-(4-chlorophenyl)-4-(quinazolin-4-yl)butanoic acid **23ah**

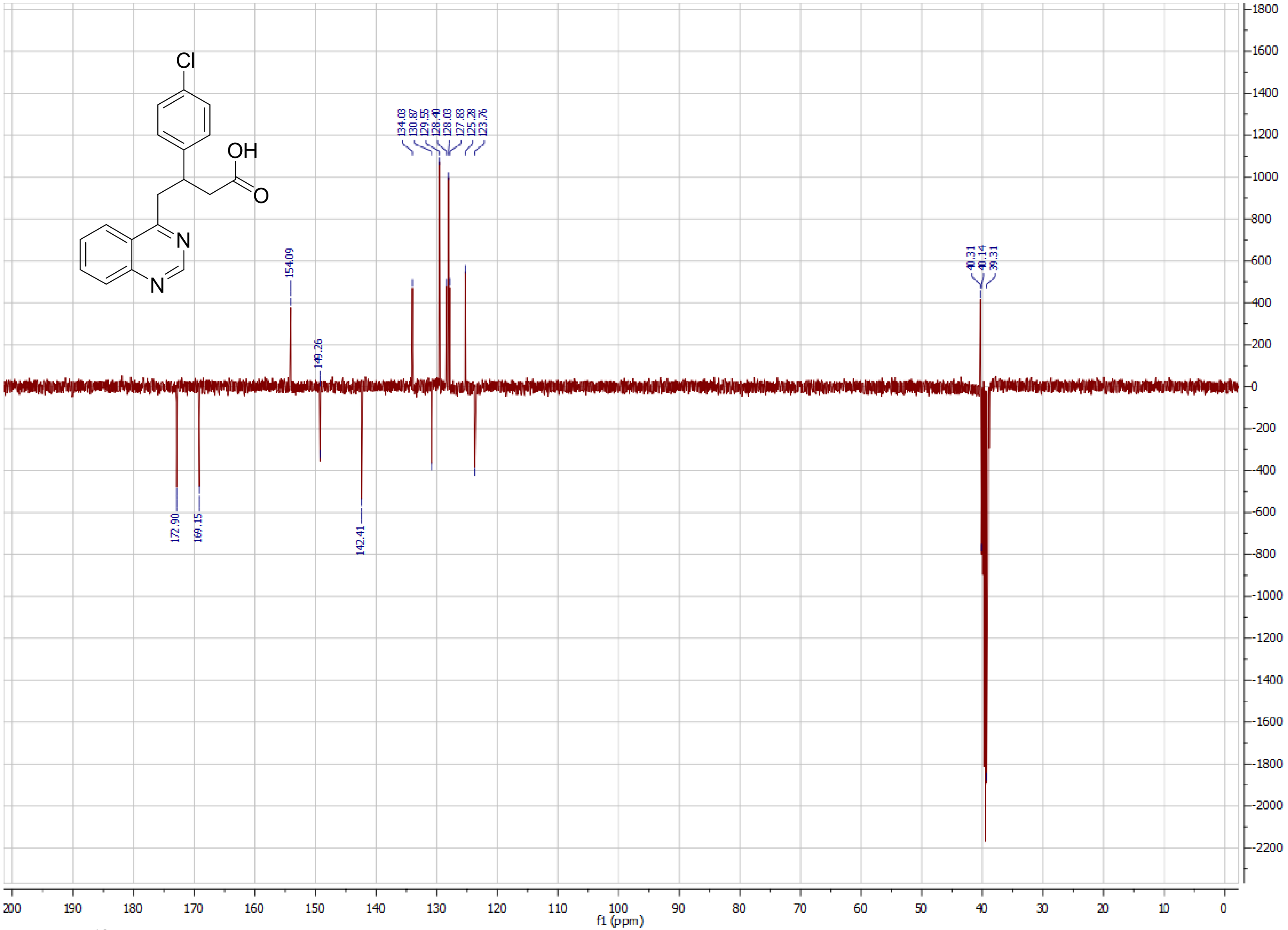

Fig S77. ^{13}C NMR spectral chart for 3-(4-chlorophenyl)-4-(quinazolin-4-yl)butanoic acid **23ah**

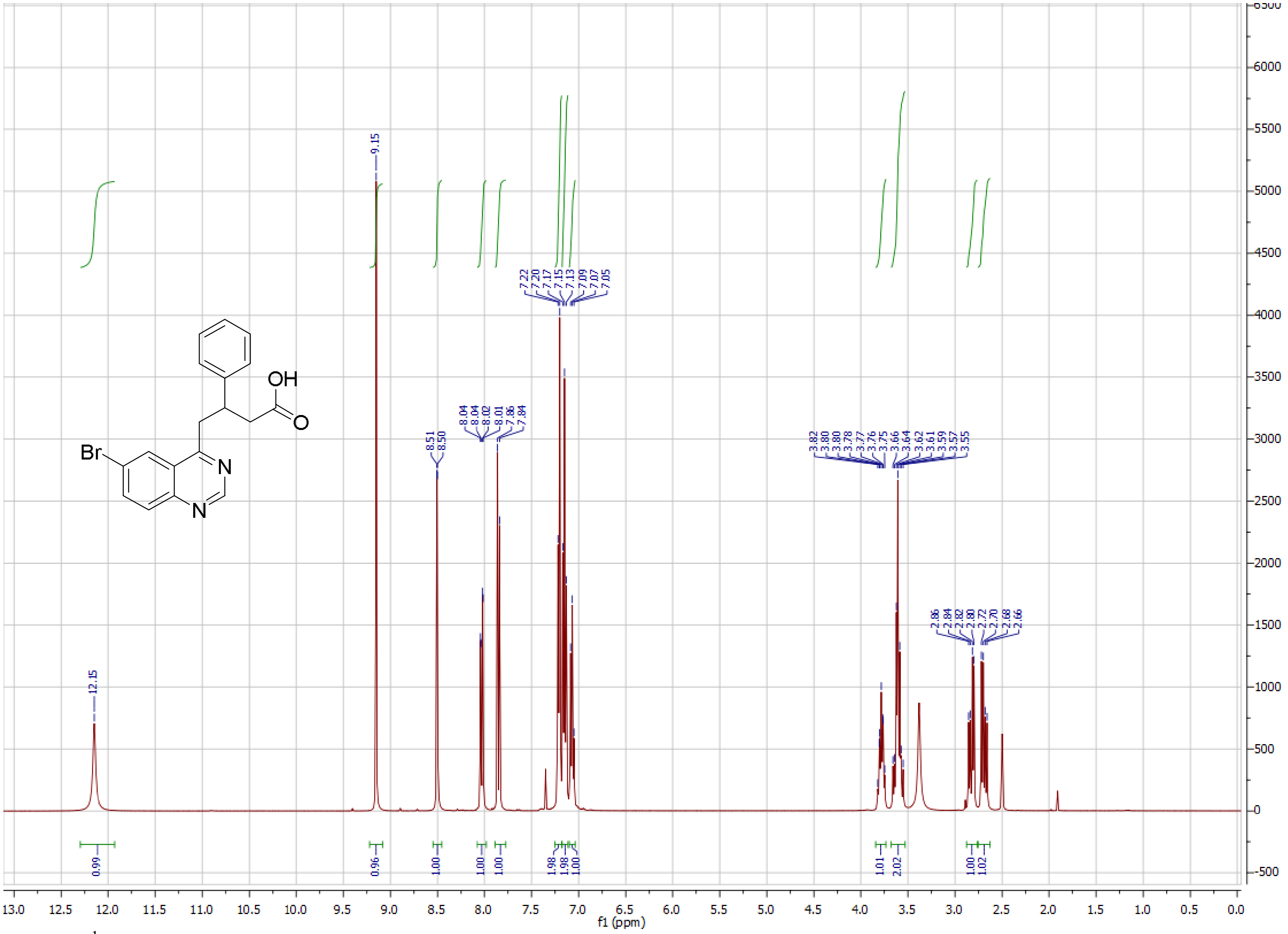

Fig S78. ^{1}H NMR spectral chart for 4-(6-bromoquinazolin-4-yl)-3-phenylbutanoic acid **23al**

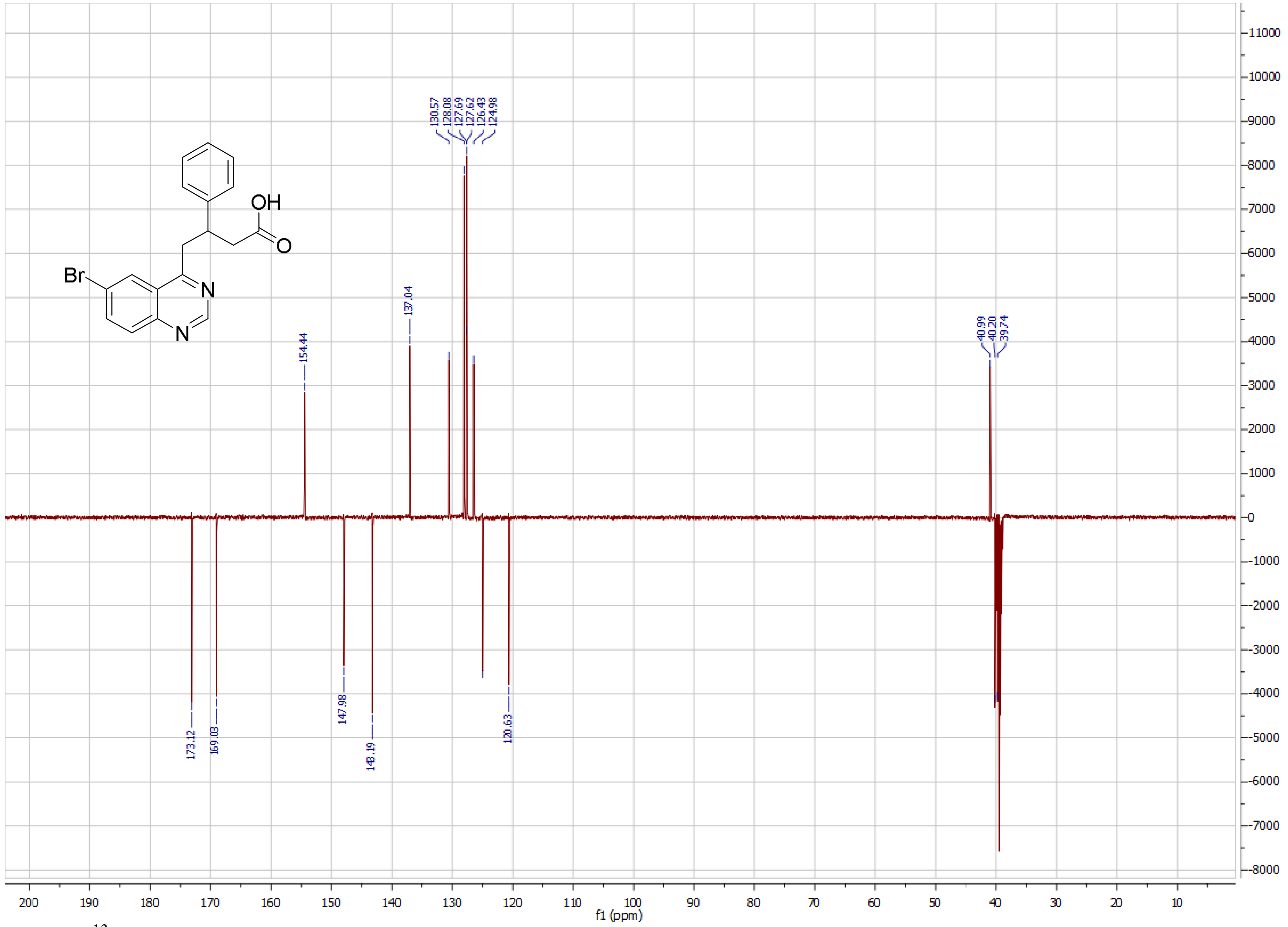

Fig S79. ^{13}C NMR spectral chart for 4- (6-bromoquinazolin-4-yl)-3-phenylbutanoic acid **23al**

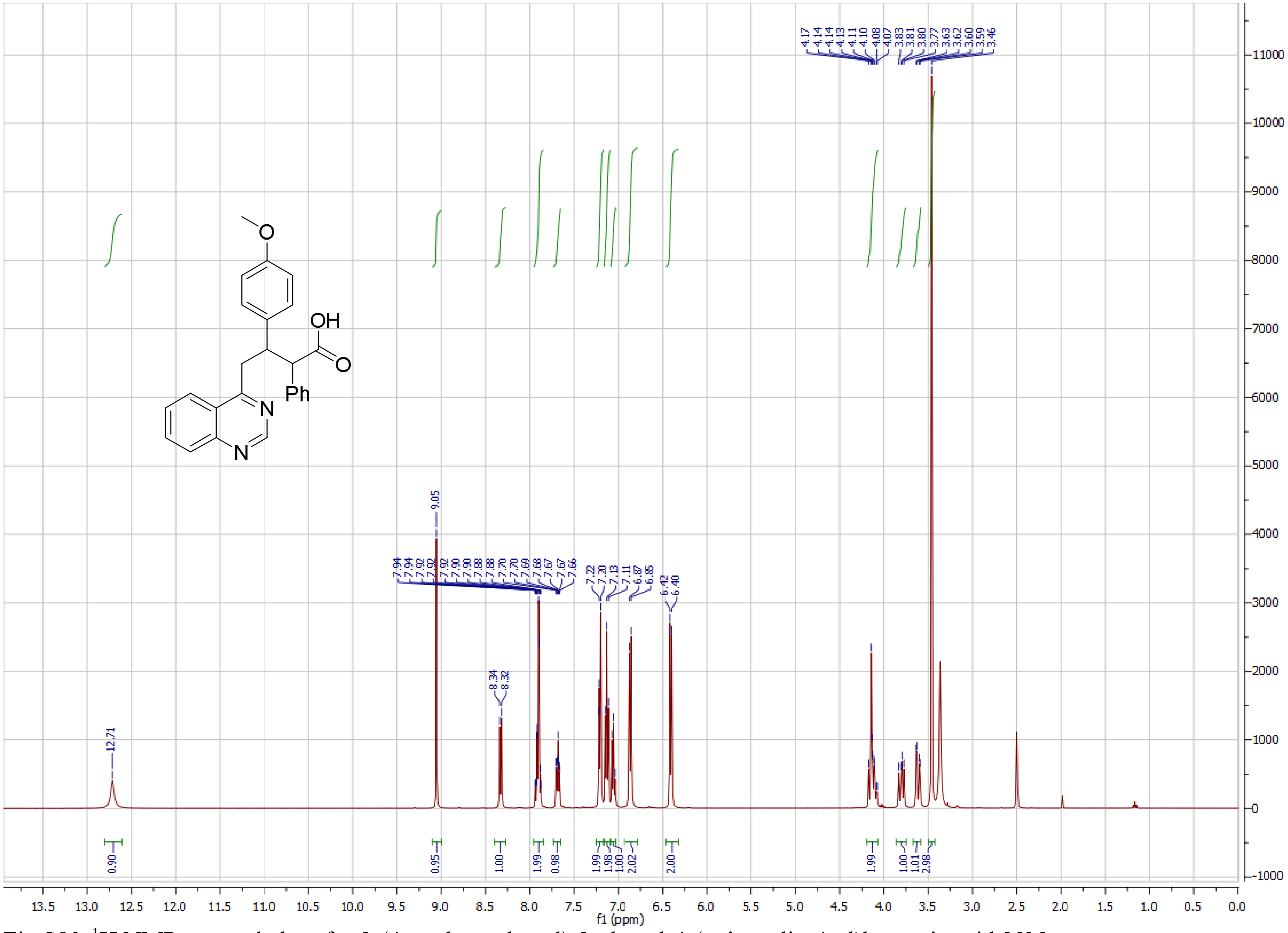

Fig S80. ^{1}H NMR spectral chart for 3-(4-methoxyphenyl)-2-phenyl-4-(quinazolin-4-yl)butanoic acid **23bb**

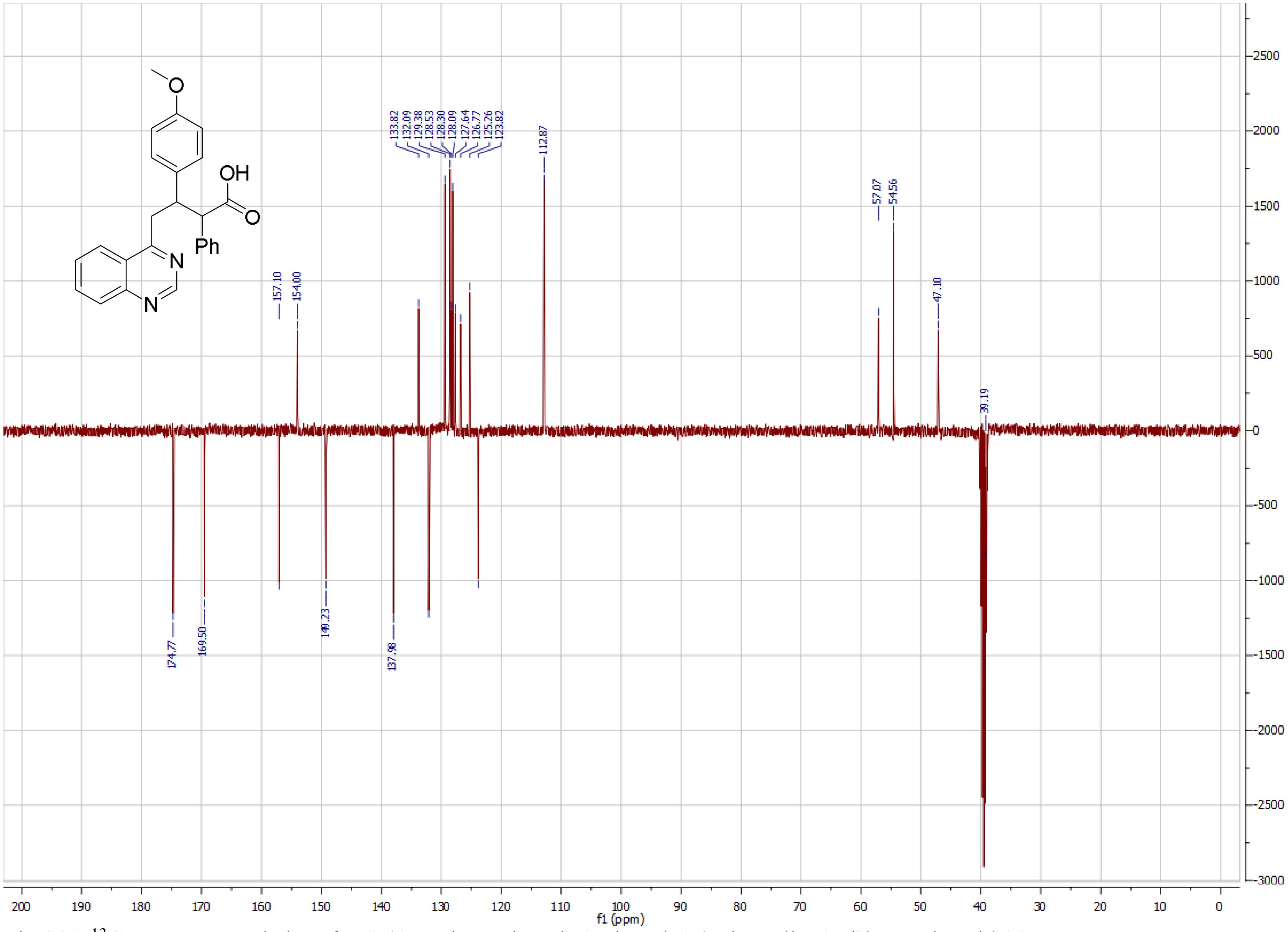

Fig S81. ^{13}C NMR spectral chart for 3-(4-methoxyphenyl)-2-phenyl-4-(quinazolin-4-yl)butanoic acid **23bb**

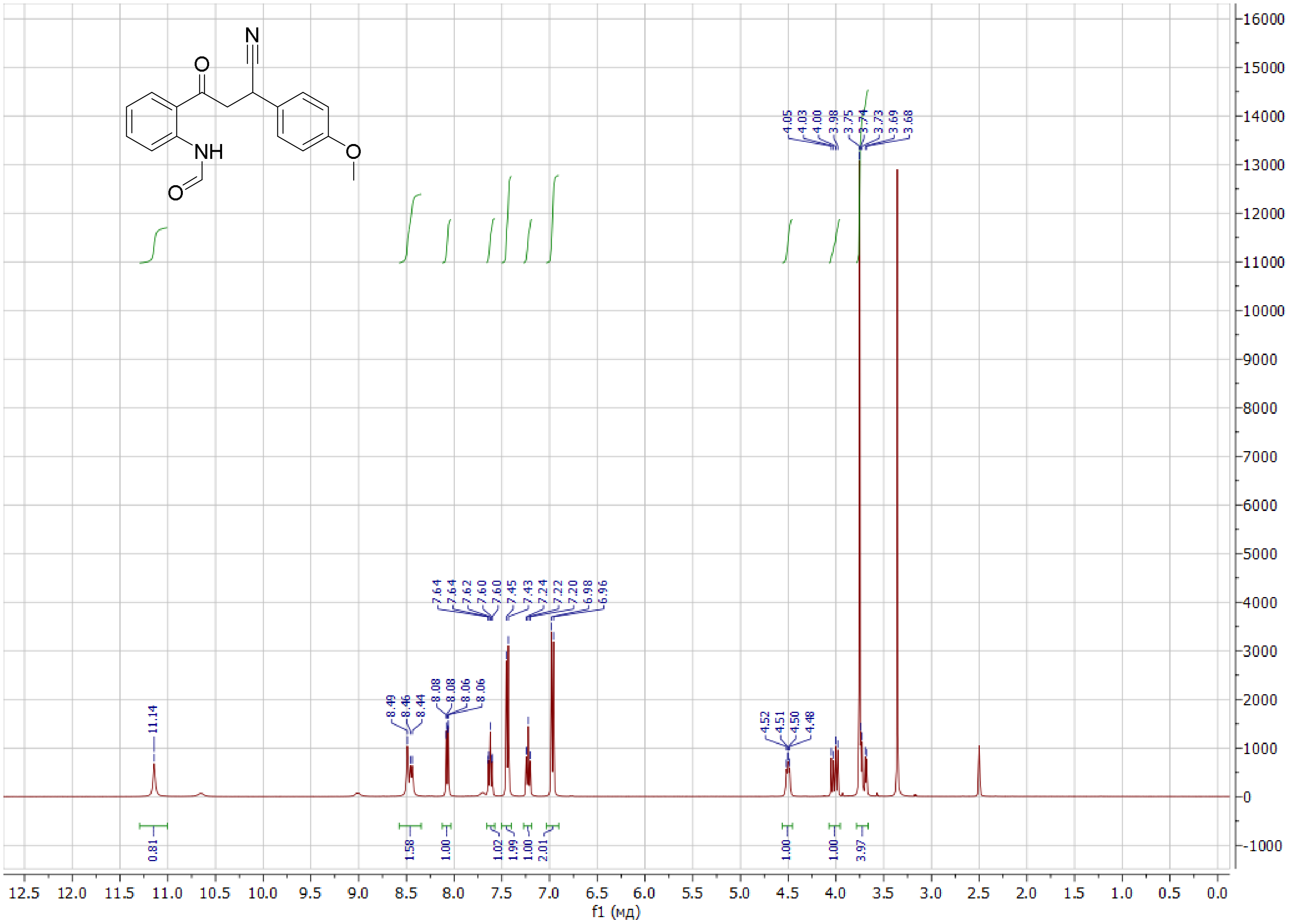

Fig S82. ^{1}H NMR spectral chart for *N*-(2-(3-Cyano-3-(4-methoxyphenyl)propanoyl)phenyl)formamide **9aa**

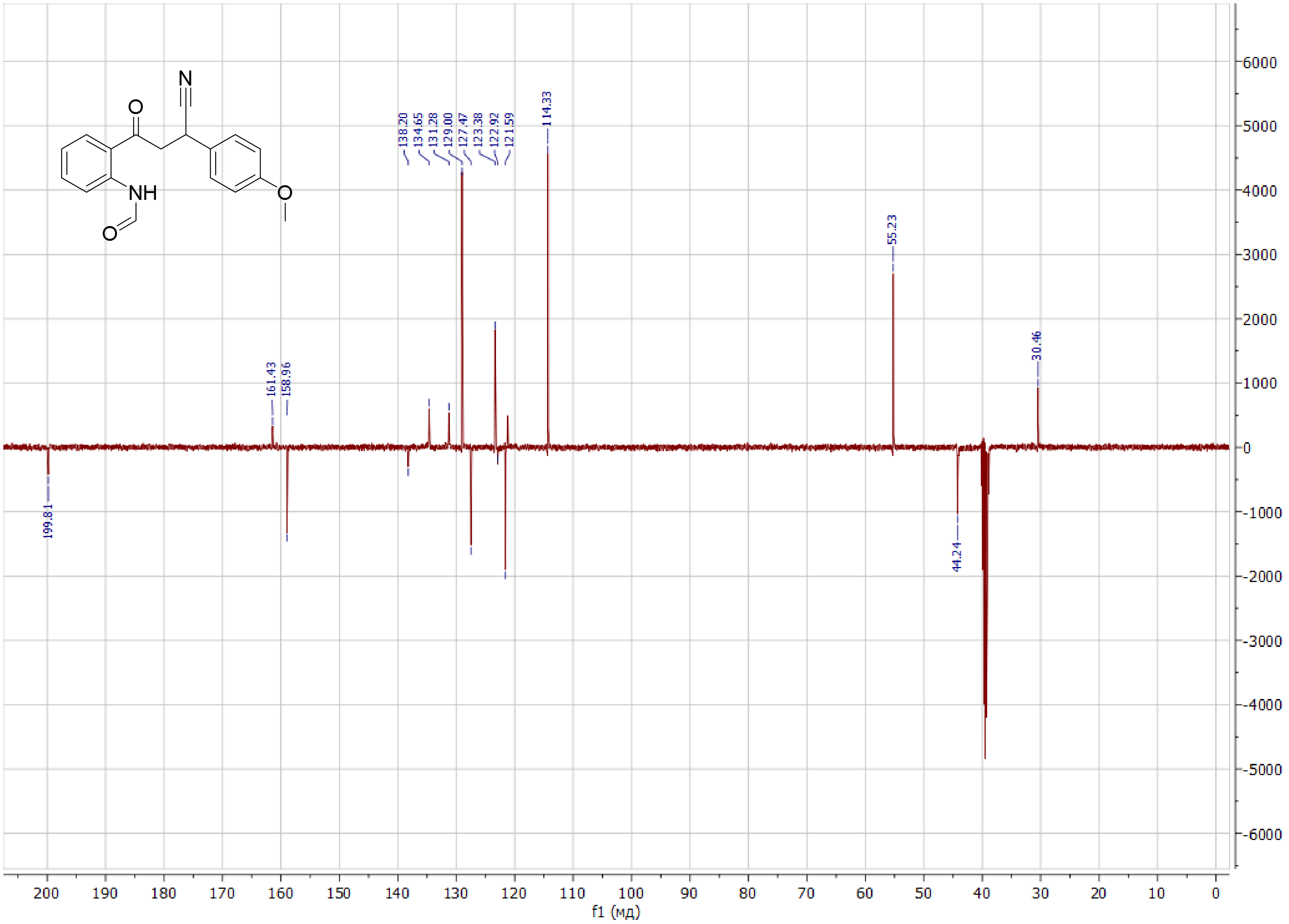

Fig S83. ^{13}C NMR spectral chart for *N*-(2-(3-Cyano-3-(4-methoxyphenyl)propanoyl)phenyl)formamide **9aa**

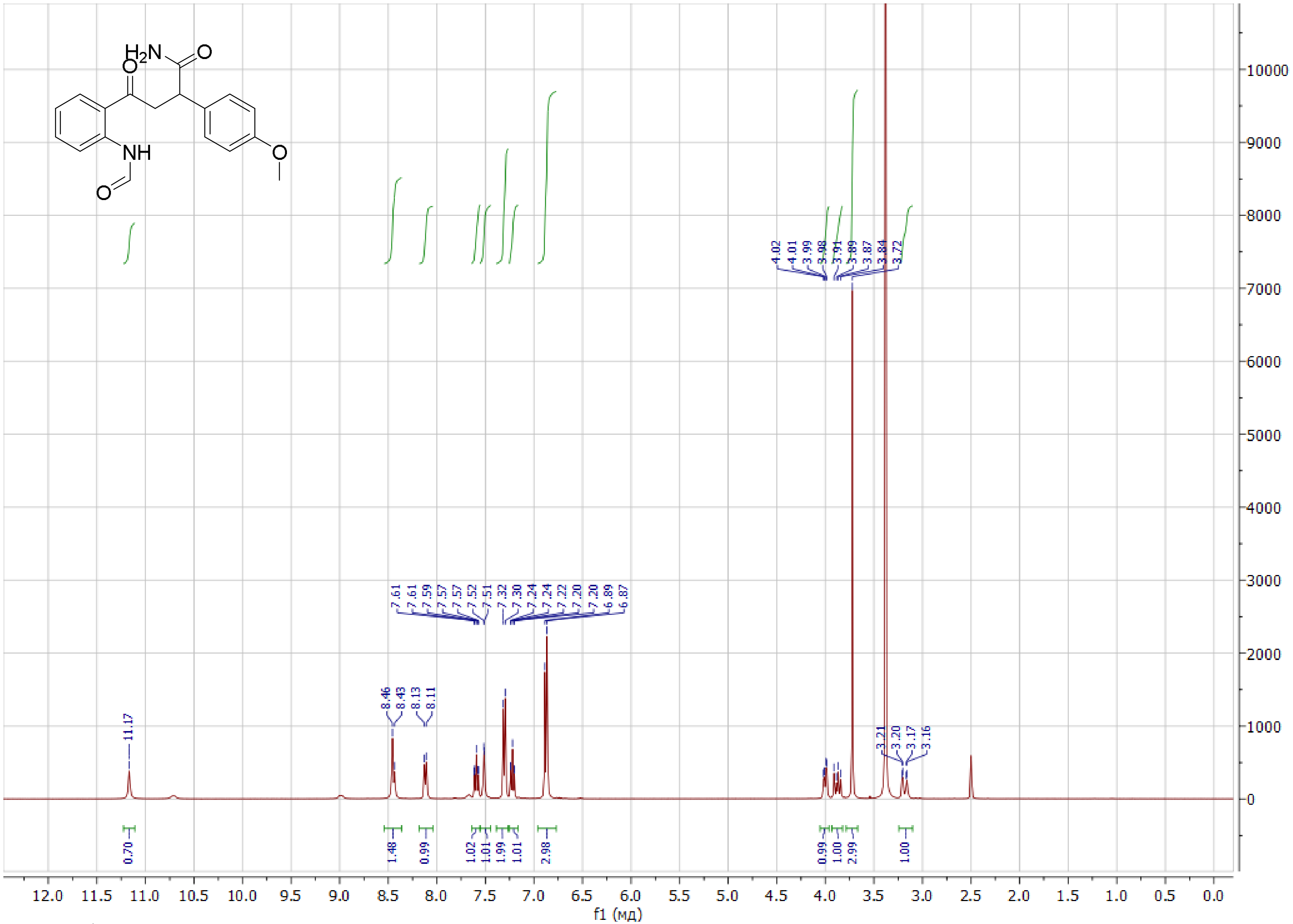

Fig S84. ^{1}H NMR spectral chart for 4-(2-Formamidophenyl)-2-(4-methoxyphenyl)-4-oxobutanamide **16aa**

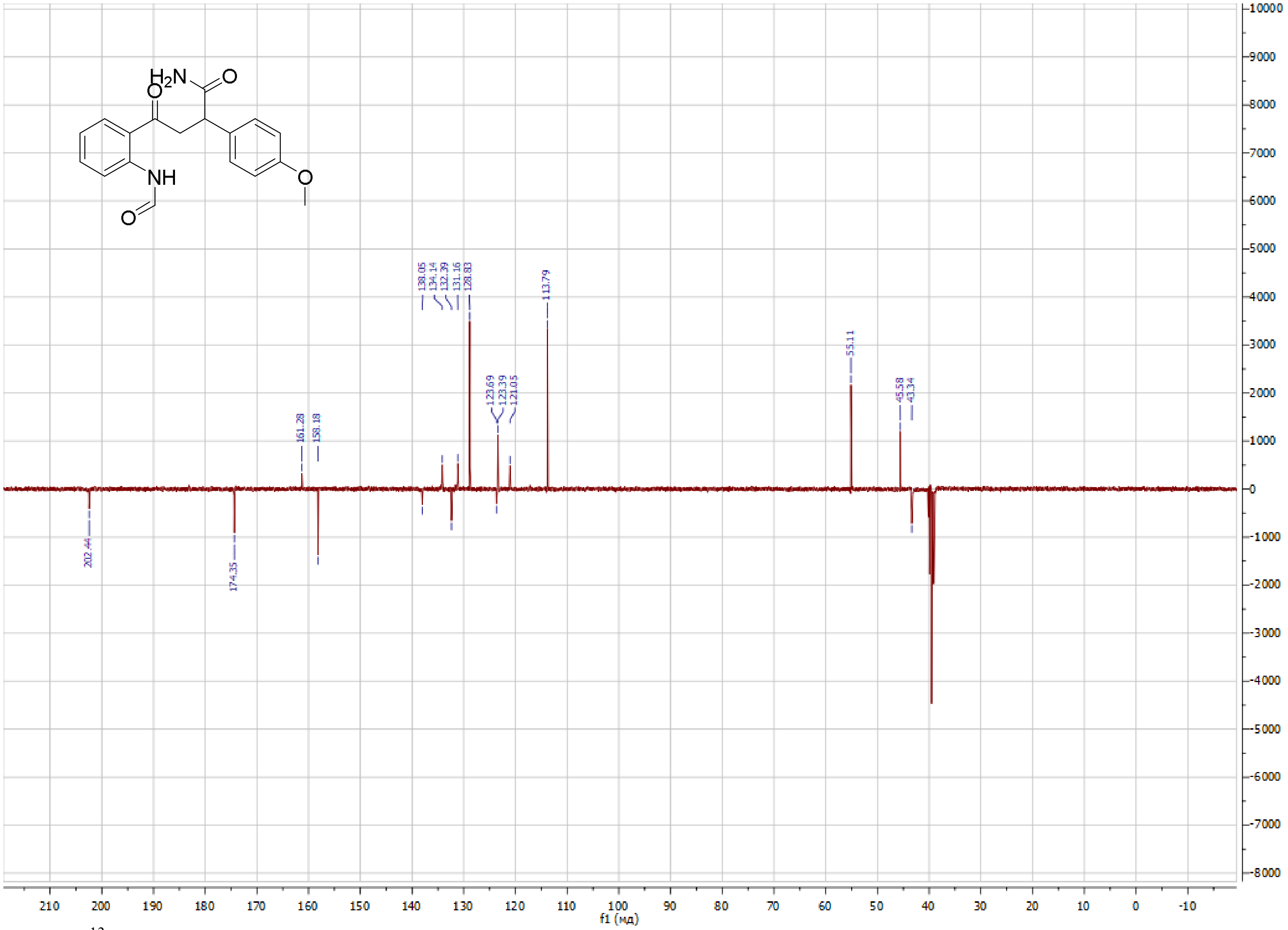

Fig S85. ^{13}C NMR spectral chart for 4-(2-Formamidophenyl)-2-(4-methoxyphenyl)-4-oxobutanamide **16aa**

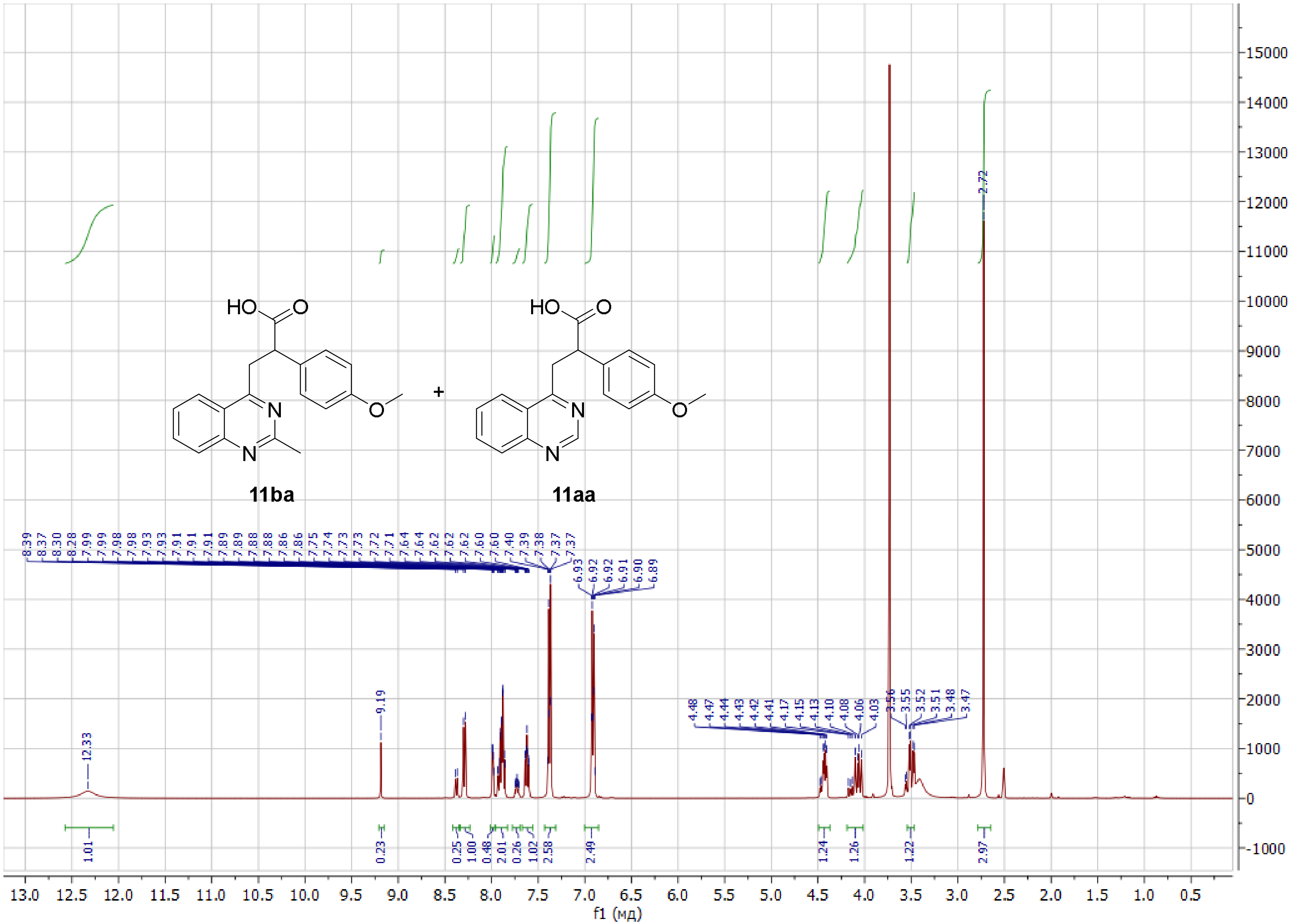

Fig S86. ^{1}H NMR spectral chart for mixture of **11ba** and **11aa**

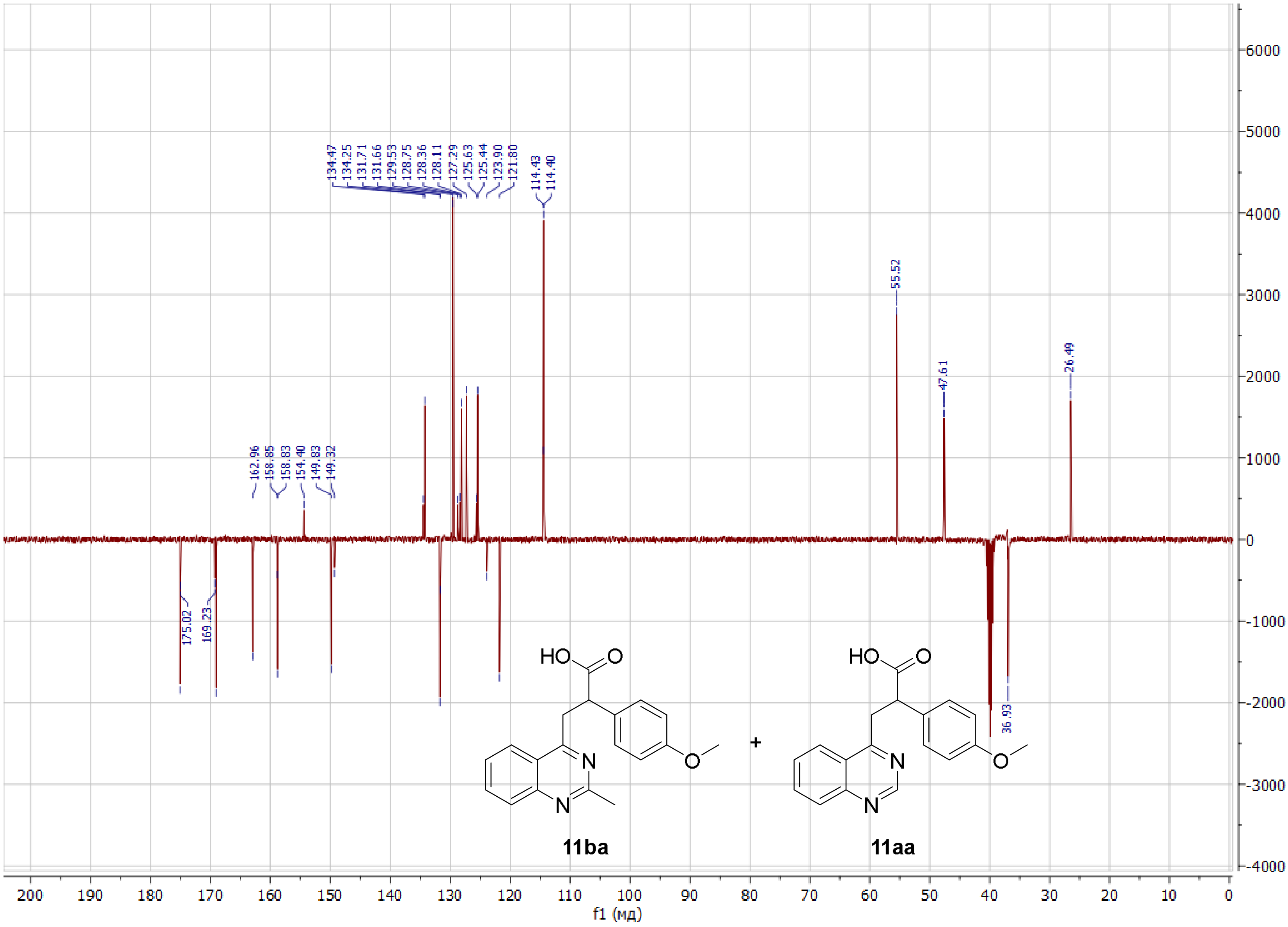

Fig S87. ^{13}C NMR spectral chart for mixture of **11ba** and **11aa**

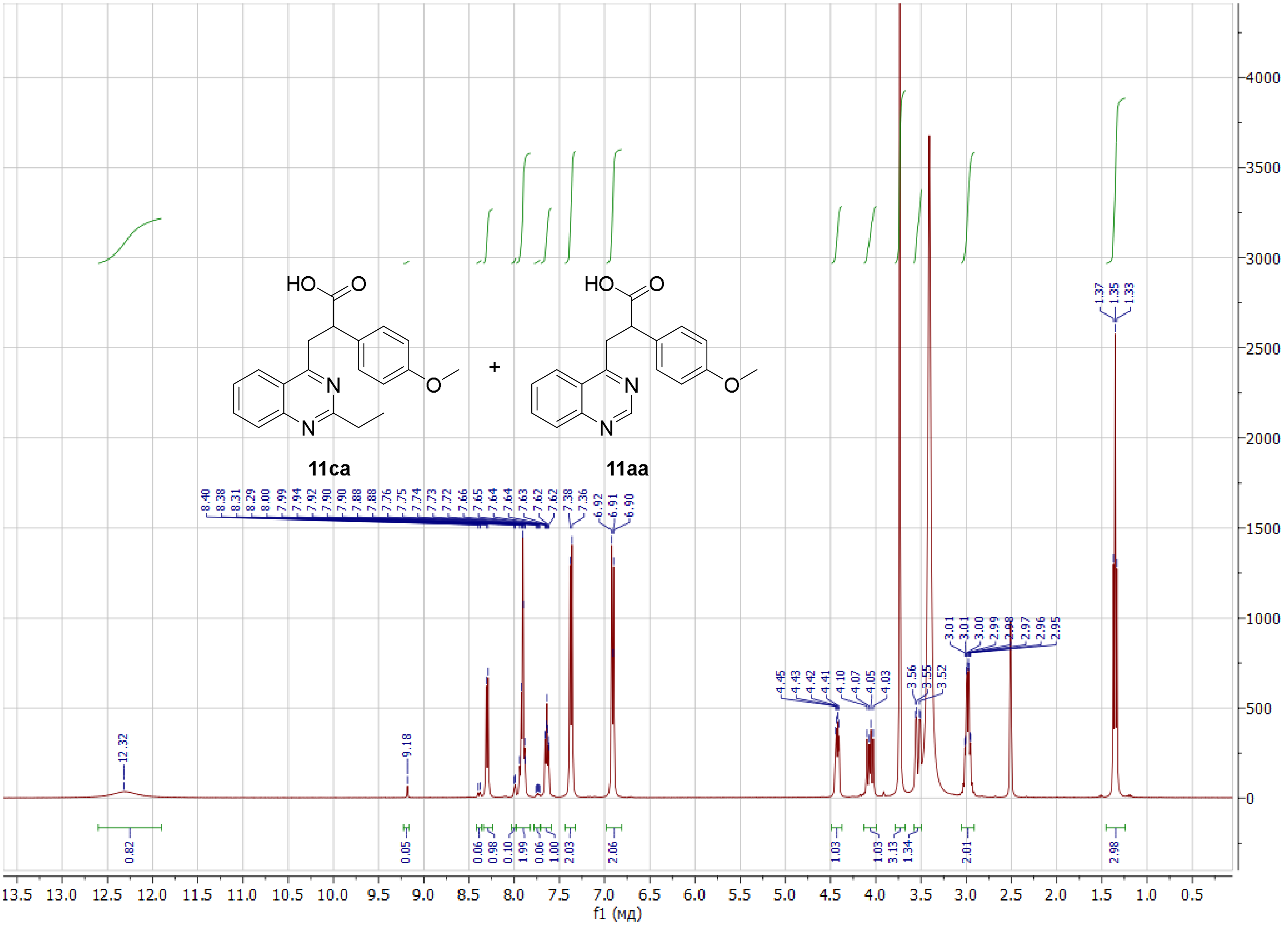

Fig S88. ^{1}H NMR spectral chart for mixture of **11ca** and **11aa**

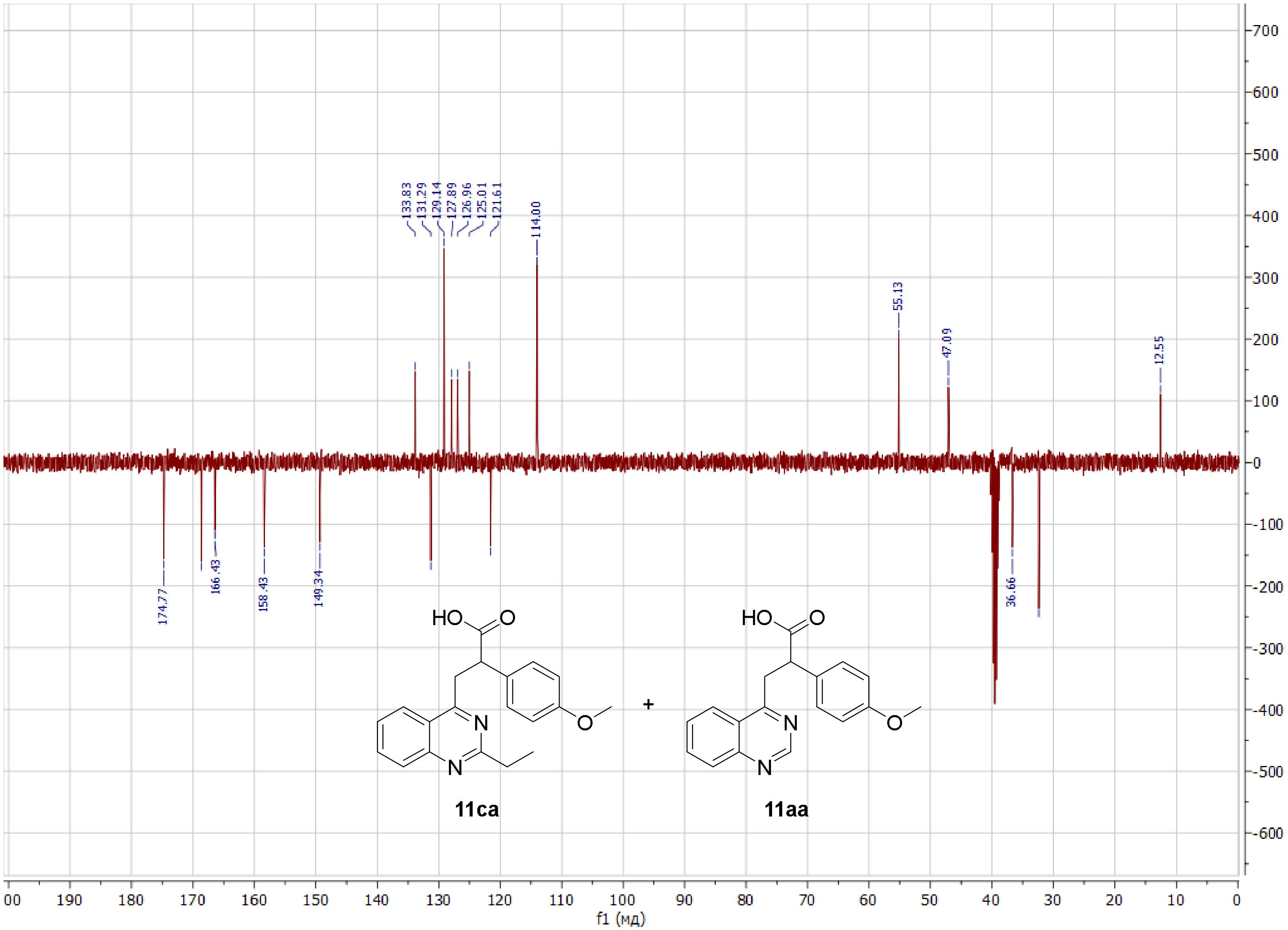

Fig S89. ^{13}C NMR spectral chart for mixture of **11ca** and **11aa**

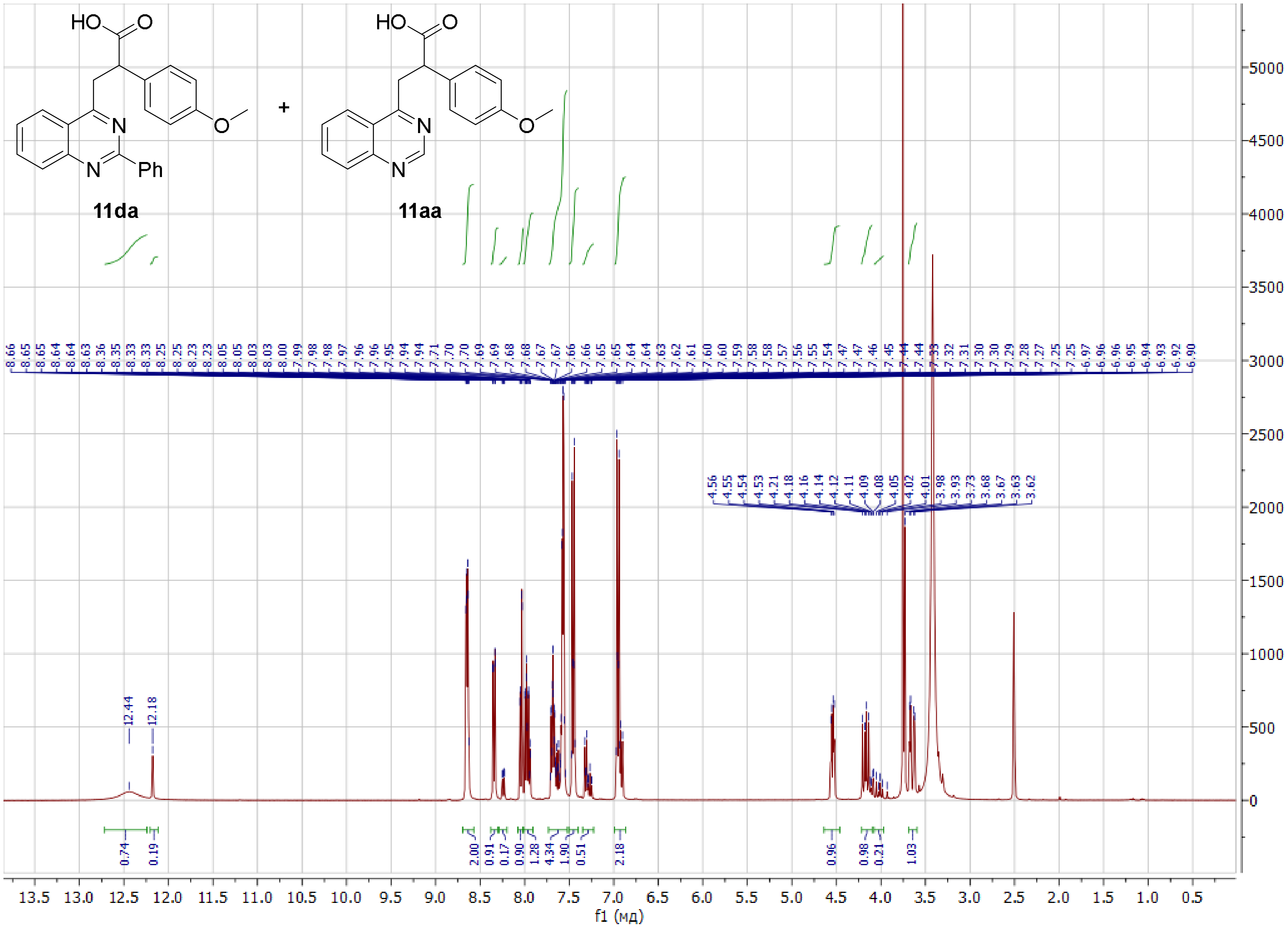

Fig S90. ^{1}H NMR spectral chart for mixture of **11da** and **11aa**

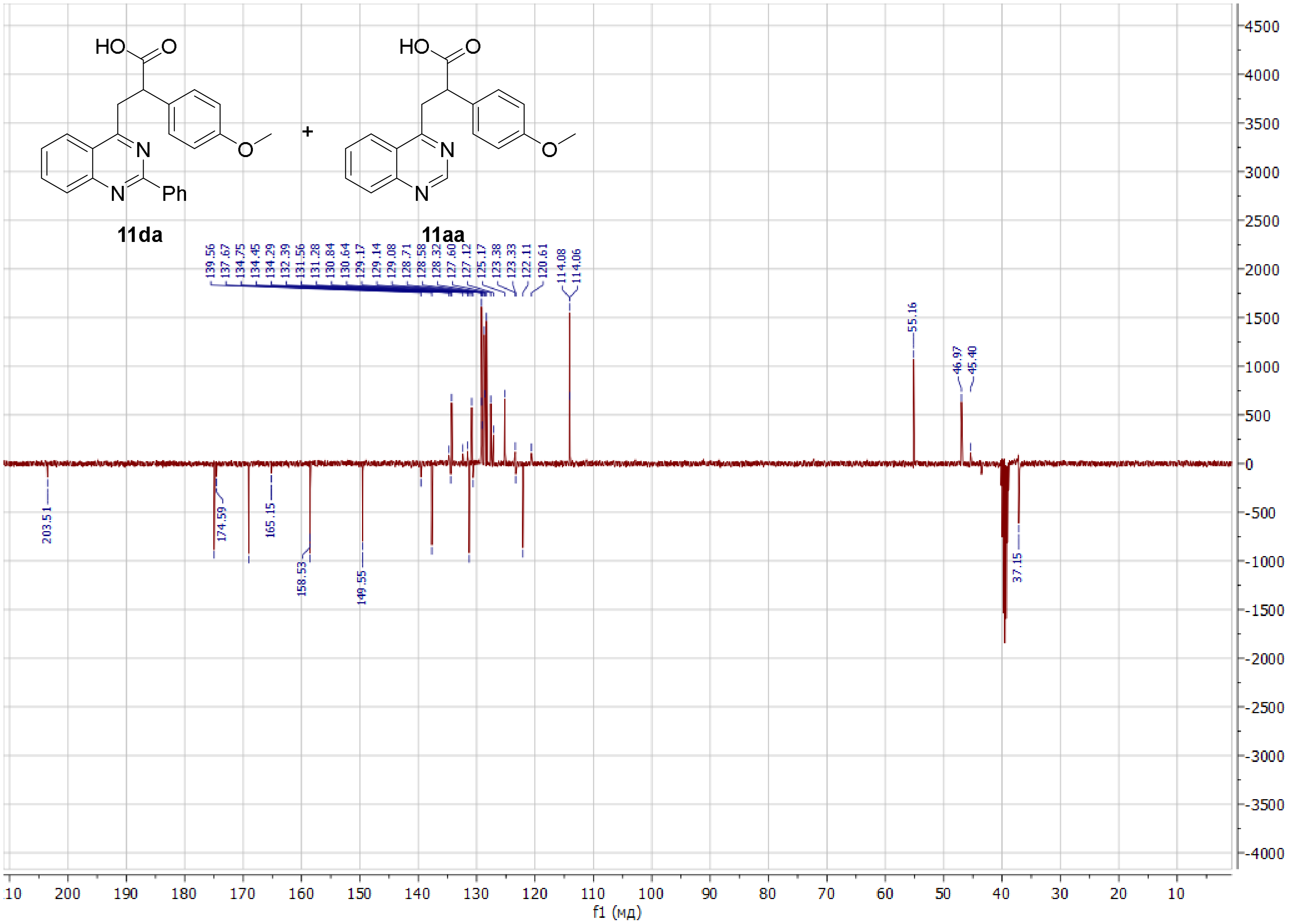

Fig S91. ^{13}C NMR spectral chart for mixture of **11da** and **11aa**

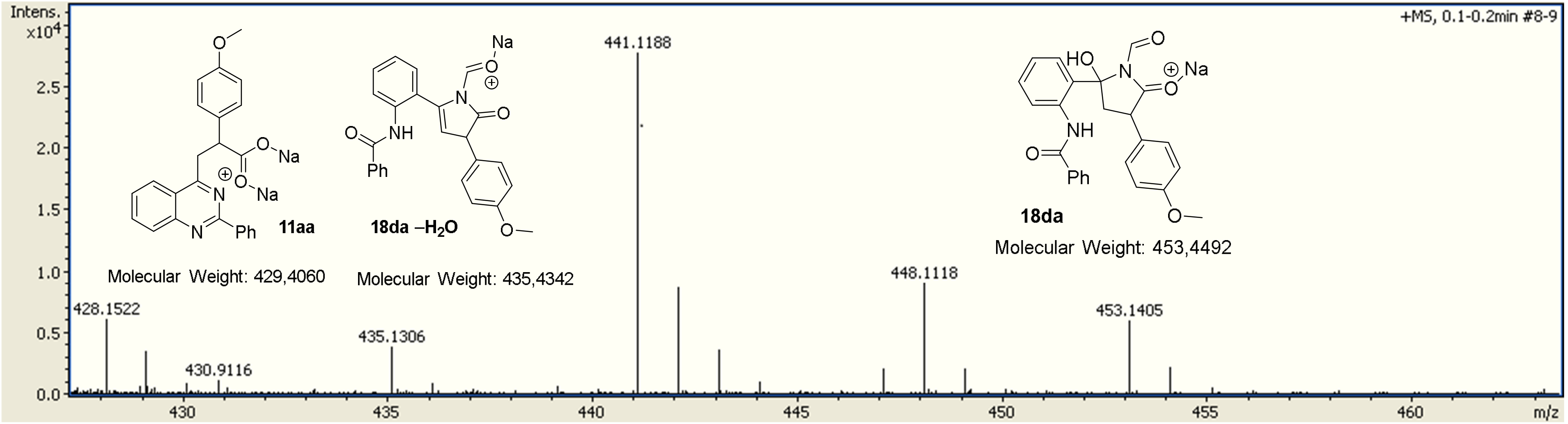

Fig S92. HRMS spectral chart for reaction mixture of reaction of **9da** in presence of HCOOH at 110 °C.

Xray diffraction data for 11ae

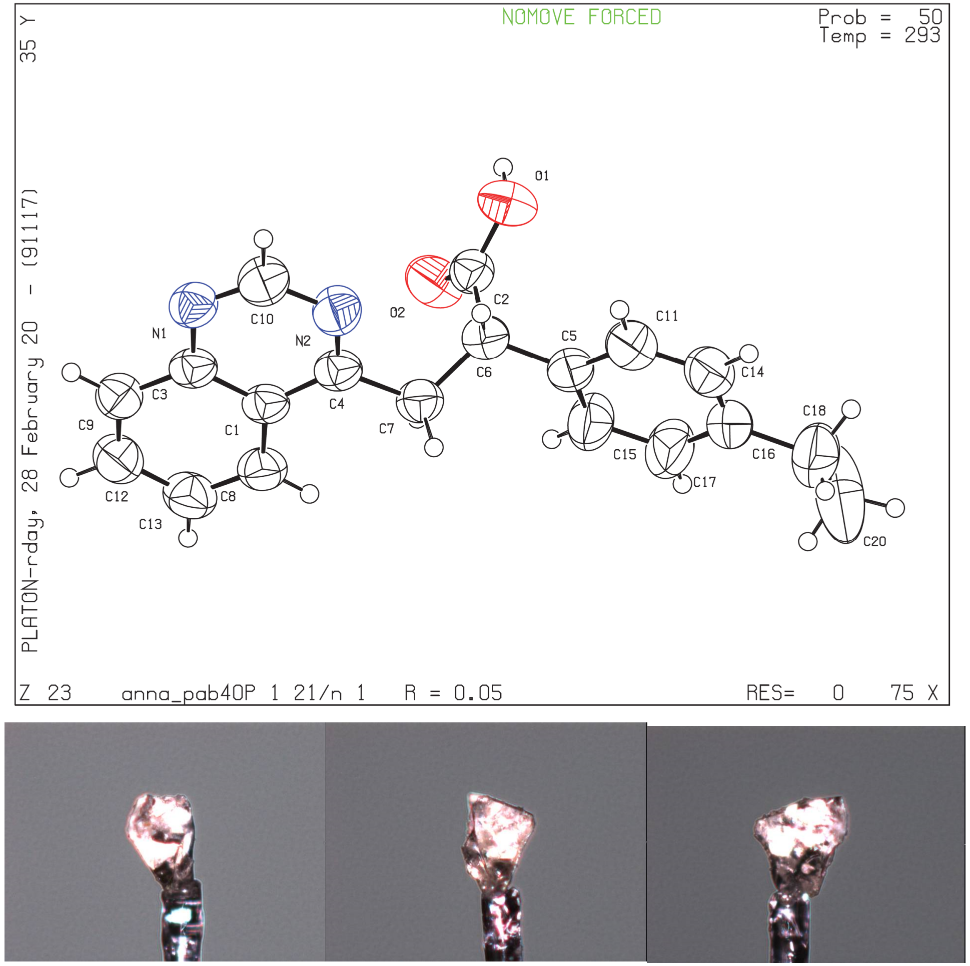

Fig S93. Anisotropic structure representation and crystal photos for **11ae**

Table 1 Crystal data and structure refinement for 11ae.

Identification code	ANNA_PAB40_1
Empirical formula	$C_{19}H_{18}N_2O_2$
Formula weight	306.35

Temperature/K	293(2)
Crystal system	monoclinic
Space group	$P2_1/n$
a/Å	11.1491(2)
b/Å	12.5064(2)
c/Å	12.2173(2)
α/°	90
β/°	109.579(2)
γ/°	90
Volume/Å^3	1605.02(5)
Z	4
ρ_{calc}g/cm^3	1.268
μ/mm^{-1}	0.666
F(000)	648.0
Crystal size/mm^3	0.47 × 0.35 × 0.2
Radiation	Cu Kα (λ = 1.54184)
2Θ range for data collection/°	9.304 to 152.554
Index ranges	-14 ≤ h ≤ 14, -15 ≤ k ≤ 13, -15 ≤ l ≤ 15
Reflections collected	17081
Independent reflections	3340 [R_{int} = 0.0160, R_{sigma} = 0.0104]
Data/restraints/parameters	3340/0/224
Goodness-of-fit on F^2	1.056
Final R indexes [I>=2σ (I)]	R_1 = 0.0493, wR_2 = 0.1332
Final R indexes [all data]	R_1 = 0.0545, wR_2 = 0.1395
Largest diff. peak/hole / e Å^{-3}	0.20/-0.29

Table 2 Fractional Atomic Coordinates (×11^4) and Equivalent Isotropic Displacement Parameters (Å^2×18) for 11ae. U_{eq} is defined as 1/3 of of the trace of the orthogonalised U_{IJ} tensor.

Atom	*x*	*y*	*z*	U(eq)
O1	4611.8(12)	7130.4(11)	8095.8(10)	77.4(3)

Table 2 Fractional Atomic Coordinates (×11⁴) and Equivalent Isotropic Displacement Parameters (Å²×18) for 11ae. U_{eq} is defined as 1/3 of of the trace of the orthogonalised U_{IJ} tensor.

Atom	*x*	*y*	*z*	U(eq)
O2	2913.9(11)	7342.1(12)	6529.6(11)	85.9(4)
N1	1733.6(13)	3406.5(10)	5931.5(11)	65.3(3)
N2	3564.6(13)	4529.9(11)	6374.8(12)	72.1(4)
C1	1850.6(14)	4796.9(11)	4594.1(12)	55.1(3)
C2	3975.0(14)	7015.4(11)	6985.4(13)	58.3(3)
C3	1200.3(14)	3944.9(11)	4906.1(12)	56.7(3)
C4	3061.3(14)	5072.3(11)	5404.0(12)	57.4(3)
C5	5694.5(14)	7251.2(12)	6148.6(13)	60.3(3)
C6	4737.7(14)	6462.5(12)	6322.1(13)	58.5(3)
C7	3825.4(15)	5997.7(12)	5195.4(13)	61.6(4)
C8	1258.2(16)	5327.5(13)	3529.7(13)	67.0(4)
C9	-21.0(16)	3649.0(13)	4166.8(14)	66.1(4)
C10	2861.4(18)	3716.2(14)	6582.2(15)	75.3(4)
C11	6957.9(15)	7219.9(15)	6856.2(15)	71.8(4)
C12	-561.1(17)	4179.7(15)	3150.9(15)	75.5(4)
C13	79.8(18)	5019.9(15)	2830.9(14)	77.6(5)
C14	7834.3(16)	7945.3(17)	6738.4(17)	79.7(5)
C15	5343.4(17)	8043.5(18)	5326.1(17)	88.9(6)
C16	7501.2(18)	8718.1(19)	5913.0(16)	86.3(6)
C17	6241(2)	8768(2)	5212.6(19)	107.9(8)
C18	8499(3)	9486(3)	5777(3)	141.5(13)
C19	8275(12)	10025(15)	4697(13)	137(5)
C20	8152(12)	10353(11)	5141(18)	174(6)

Table 3 Anisotropic Displacement Parameters (Å²×18) for 11ae. The Anisotropic displacement factor exponent takes the form: -$2\pi^2[h^2a^{*2}U_{11}+2hka^*b^*U_{12}+\ldots]$.

**Atom**	**U_{11}**	**U_{22}**	**U_{33}**	**U_{23}**	**U_{8}**	**U_{12}**
O1	87.4(8)	84.6(8)	57.6(6)	-6.0(5)	21.0(6)	17.2(6)
O2	63.5(7)	114.0(10)	77.4(8)	-20.4(7)	19.8(6)	14.0(7)
N1	78.4(8)	57.5(7)	62.5(7)	5.7(6)	27.0(6)	-1.3(6)
N2	76.5(8)	66.8(8)	68.1(8)	12.0(6)	17.9(6)	-4.0(6)
C1	69.9(8)	50.2(7)	50.6(7)	-4.6(5)	27.5(6)	3.7(6)
C2	63.5(8)	55.2(7)	58.8(8)	-0.5(6)	23.8(6)	-2.9(6)
C3	71.7(9)	51.2(7)	53.2(7)	-3.2(5)	29.0(6)	4.1(6)
C4	70.6(8)	51.4(7)	55.2(7)	-2.1(6)	27.8(6)	4.4(6)
C5	60.4(8)	64.3(8)	60.6(8)	-5.1(6)	26.1(6)	1.2(6)
C6	63.5(8)	55.4(7)	58.4(7)	1.2(6)	22.8(6)	4.7(6)
C7	72.7(9)	58.0(8)	58.3(8)	-1.6(6)	27.3(7)	-2.1(6)
C8	85.4(10)	62.3(8)	55.4(8)	2.1(6)	26.3(7)	-4.6(7)
C9	74.1(9)	62.4(8)	65.7(9)	-6.0(7)	28.3(7)	-4.8(7)
C10	85.2(11)	68.1(9)	67.4(9)	16.7(8)	18.8(8)	-1.4(8)
C11	62.9(9)	79.3(10)	73.1(10)	-5.1(8)	22.5(7)	9.2(8)
C12	77.5(10)	77.4(11)	66.3(9)	-3.8(8)	17.3(8)	-3.9(8)
C13	91.8(12)	77.5(11)	56.1(8)	4.7(7)	14.9(8)	-2.0(9)
C14	58.5(9)	104.9(14)	76.9(11)	-19.4(10)	24.1(8)	-3.0(9)
C15	66.7(10)	104.8(14)	84.0(12)	25.0(11)	10.4(8)	-19.6(9)
C16	71.8(10)	119.1(16)	69.1(10)	-12.0(10)	25.2(8)	-29.1(10)
C17	95.2(14)	127.4(19)	87.7(13)	35.2(13)	13.2(11)	-38.0(13)
C18	104.7(18)	200(4)	108.7(19)	10(2)	21.5(15)	-76(2)
C19	97(5)	181(12)	130(8)	44(7)	32(5)	-77(7)
C20	118(6)	111(5)	220(13)	33(7)	-39(6)	-44(4)

Table 4 Bond Lengths for 11ae.

**Atom**	**Atom**	**Length/Å**	**Atom**	**Atom**	**Length/Å**
O1	C2	1.3113(18)	C5	C11	1.384(2)

Table 4 Bond Lengths for 11ae.

Atom	Atom	Length/Å	Atom	Atom	Length/Å
O2	C2	1.1980(18)	C5	C15	1.372(2)
N1	C3	1.3701(18)	C6	C7	1.526(2)
N1	C10	1.301(2)	C8	C13	1.361(2)
N2	C4	1.3174(19)	C9	C12	1.358(2)
N2	C10	1.359(2)	C11	C14	1.376(3)
C1	C3	1.411(2)	C12	C13	1.398(3)
C1	C4	1.423(2)	C14	C16	1.356(3)
C1	C8	1.411(2)	C15	C17	1.391(3)
C2	C6	1.523(2)	C16	C17	1.380(3)
C3	C9	1.408(2)	C16	C18	1.521(3)
C4	C7	1.509(2)	C18	C19	1.426(12)
C5	C6	1.519(2)	C18	C20	1.314(11)

Table 5 Bond Angles for 11ae.

Atom	Atom	Atom	Angle/˚	Atom	Atom	Atom	Angle/˚
C10	N1	C3	116.54(13)	C5	C6	C2	108.83(12)
C4	N2	C10	117.07(14)	C5	C6	C7	114.11(12)
C3	C1	C4	116.65(13)	C4	C7	C6	112.53(12)
C3	C1	C8	118.67(14)	C13	C8	C1	120.03(15)
C8	C1	C4	124.67(14)	C12	C9	C3	119.69(15)
O1	C2	C6	113.27(13)	N1	C10	N2	127.49(15)
O2	C2	O1	123.47(14)	C14	C11	C5	121.71(17)
O2	C2	C6	123.23(14)	C9	C12	C13	120.76(16)
N1	C3	C1	120.88(13)	C8	C13	C12	120.90(16)
N1	C3	C9	119.17(14)	C16	C14	C11	121.39(17)
C9	C3	C1	119.94(13)	C5	C15	C17	120.38(17)
N2	C4	C1	121.33(14)	C14	C16	C17	117.50(18)

Table 5 Bond Angles for 11ae.

Atom	Atom	Atom	Angle/°	Atom	Atom	Atom	Angle/°
N2	C4	C7	116.93(13)	C14	C16	C18	120.2(2)
C1	C4	C7	121.74(13)	C17	C16	C18	122.3(2)
C11	C5	C6	120.64(14)	C16	C17	C15	121.7(2)
C15	C5	C6	122.00(14)	C19	C18	C16	119.4(5)
C15	C5	C11	117.31(15)	C20	C18	C16	120.3(6)
C2	C6	C7	109.31(12)				

Table 6 Hydrogen Bonds for 11ae.

D	H	A	d(D-H)/Å	d(H-A)/Å	d(D-A)/Å	D-H-A/°
O1	H1	N1[1]	0.99(3)	1.73(3)	2.7210(17)	178(2)

[1]1/2-X,1/2+Y,3/2-Z

Table 7 Torsion Angles for 11ae.

A	B	C	D	Angle/°	A	B	C	D	Angle/°
O1	C2	C6	C5	-75.58(16)	C8	C1	C3	N1	-179.83(13)
O1	C2	C6	C7	159.19(13)	C8	C1	C3	C9	-0.8(2)
O2	C2	C6	C5	102.19(18)	C8	C1	C4	N2	-178.98(15)
O2	C2	C6	C7	-23.0(2)	C8	C1	C4	C7	1.2(2)
N1	C3	C9	C12	179.72(15)	C9	C12	C13	C8	-0.1(3)
N2	C4	C7	C6	-25.47(19)	C10	N1	C3	C1	-0.7(2)
C1	C3	C9	C12	0.6(2)	C10	N1	C3	C9	-179.76(14)
C1	C4	C7	C6	154.34(13)	C10	N2	C4	C1	-1.8(2)
C1	C8	C13	C12	0.0(3)	C10	N2	C4	C7	178.04(14)
C2	C6	C7	C4	-68.75(15)	C11	C5	C6	C2	100.52(16)
C3	N1	C10	N2	1.5(3)	C11	C5	C6	C7	-137.11(15)

Table 7 Torsion Angles for 11ae.

A	B	C	D	Angle/°	A	B	C	D	Angle/°
C3	C1	C4	N2	2.4(2)	C11	C5	C15	C17	0.9(3)
C3	C1	C4	C7	-177.36(12)	C11	C14	C16	C17	1.7(3)
C3	C1	C8	C13	0.4(2)	C11	C14	C16	C18	-178.0(2)
C3	C9	C12	C13	-0.2(3)	C14	C16	C17	C15	-1.3(4)
C4	N2	C10	N1	-0.3(3)	C14	C16	C18	C19	157.7(11)
C4	C1	C3	N1	-1.16(19)	C14	C16	C18	C20	-167.0(13)
C4	C1	C3	C9	177.91(12)	C15	C5	C6	C2	-76.9(2)
C4	C1	C8	C13	-178.11(15)	C15	C5	C6	C7	45.5(2)
C5	C6	C7	C4	169.14(12)	C15	C5	C11	C14	-0.5(3)
C5	C11	C14	C16	-0.8(3)	C17	C16	C18	C19	-21.9(12)
C5	C15	C17	C16	0.1(4)	C17	C16	C18	C20	13.4(14)
C6	C5	C11	C14	-178.04(14)	C18	C16	C17	C15	178.3(3)
C6	C5	C15	C17	178.3(2)					

Table 8 Hydrogen Atom Coordinates (Å×11[4]) and Isotropic Displacement Parameters (Å[2]×18) for 11ae.

Atom	*x*	*y*	*z*	U(eq)
H6	5210.02	5870.06	6799.19	70
H7A	3247.47	6554.31	4776	74
H7B	4309.33	5758.9	4712.95	74
H8	1673.53	5887.72	3305.84	80
H9	-455.57	3092.54	4373.56	79
H10	3228.74	3337.09	7268.22	90
H11	7220.96	6694.76	7425.86	86
H12	-1366.77	3982.21	2662.1	91
H13	-304.63	5373.3	2130.79	93
H14	8672.81	7904.71	7235.54	96
H15	4500.68	8096.23	4841.41	107

Table 8 Hydrogen Atom Coordinates (Å×11⁴) and Isotropic Displacement Parameters (Å²×18) for 11ae.

Atom	*x*	*y*	*z*	U(eq)
H17	5984.48	9300.72	4650.52	129
H18A	8641.4	10028.61	6374.46	170
H18B	9288.26	9090.25	5942.39	170
H18C	9017.73	9712.57	6550	170
H18D	9049.3	9080.85	5460.72	170
H19A	8234.47	9510.6	4102.36	206
H19B	8954.9	10518.94	4766.94	206
H19C	7483.2	10406.36	4495.87	206
H20A	7681.11	10155.41	4354.58	260
H20B	8894.27	10750.73	5158.13	260
H20C	7626.11	10785.83	5445.82	260
H1	4100(30)	7600(20)	8430(20)	140(10)

Table 9 Atomic Occupancy for 11ae.

Atom	*Occupancy*	Atom	*Occupancy*	Atom	*Occupancy*
H18A	0.43(3)	H18B	0.43(3)	H18C	0.57(3)
H18D	0.57(3)	C19	0.43(3)	H19A	0.43(3)
H19B	0.43(3)	H19C	0.43(3)	C20	0.57(3)
H20A	0.57(3)	H20B	0.57(3)	H20C	0.57(3)

Experimental

Single crystals of $C_{19}H_{18}N_2O_2$ **11ae** were **Crystallized by slow evaporation of saturated solution in EtOAc**. A suitable crystal was selected and **Mounted on the glass stick by acrylic glue.** on a **SuperNova, Dual, Cu at home/near, AtlasS2** diffractometer. The crystal was kept at 293(2) K during data collection. Using Olex2 [39], the structure was solved with the SHELXT [40] structure solution program using Intrinsic Phasing and refined with the SHELXL [41] refinement package using Least Squares minimisation.

Crystal structure determination of 11ae

Crystal Data for $C_{19}H_{18}N_2O_2$ (M =306.35 g/mol): monoclinic, space group P2$_1$/n (no. 14), a = 11.1491(2) Å, b = 12.5064(2) Å, c = 12.2173(2) Å, β = 109.579(2)°, V = 1605.02(5) Å^3, Z = 4, T = 293(2) K, μ(Cu Kα) = 0.666 mm^{-1}, $Dcalc$ = 1.268 g/cm^3, 17081 reflections measured (9.304° ≤ 2Θ ≤ 152.554°), 3340 unique (R_{int} = 0.0160, R_{sigma} = 0.0104) which were used in all calculations. The final R_1 was 0.0493 (I > 2σ(I)) and wR_2 was 0.1395 (all data).

Refinement model description

Number of restraints - 0, number of constraints - unknown.

Details:

1. Fixed Uiso

At 1.2 times of:

All C(H) groups, All C(H,H) groups, All C(H,H,H,H) groups

At 1.5 times of:

All C(H,H,H) groups

2. Others

Sof(H18C)=Sof(H18D)=Sof(C20)=Sof(H20A)=Sof(H20B)=Sof(H20C)=1-FVAR(1)

Sof(H18A)=Sof(H18B)=Sof(C19)=Sof(H19A)=Sof(H19B)=Sof(H19C)=FVAR(1)

3.a Ternary CH refined with riding coordinates:

C6(H6)

3.b Secondary CH2 refined with riding coordinates:

C7(H7A,H7B), C18(H18A,H18B), C18(H18C,H18D)

3.c Aromatic/amide H refined with riding coordinates:

C8(H8), C9(H9), C10(H10), C11(H11), C12(H12), C13(H13), C14(H14), C15(H15), C17(H17)

3.d Idealised Me refined as rotating group:

C19(H19A,H19B,H19C), C20(H20A,H20B,H20C)

This report has been created with Olex2, compiled on 2020.11.12 svn.r5f609507 for OlexSys. Please let us know if there are any errors or if you would like to have additional features.

Xray diffraction data for 11ca

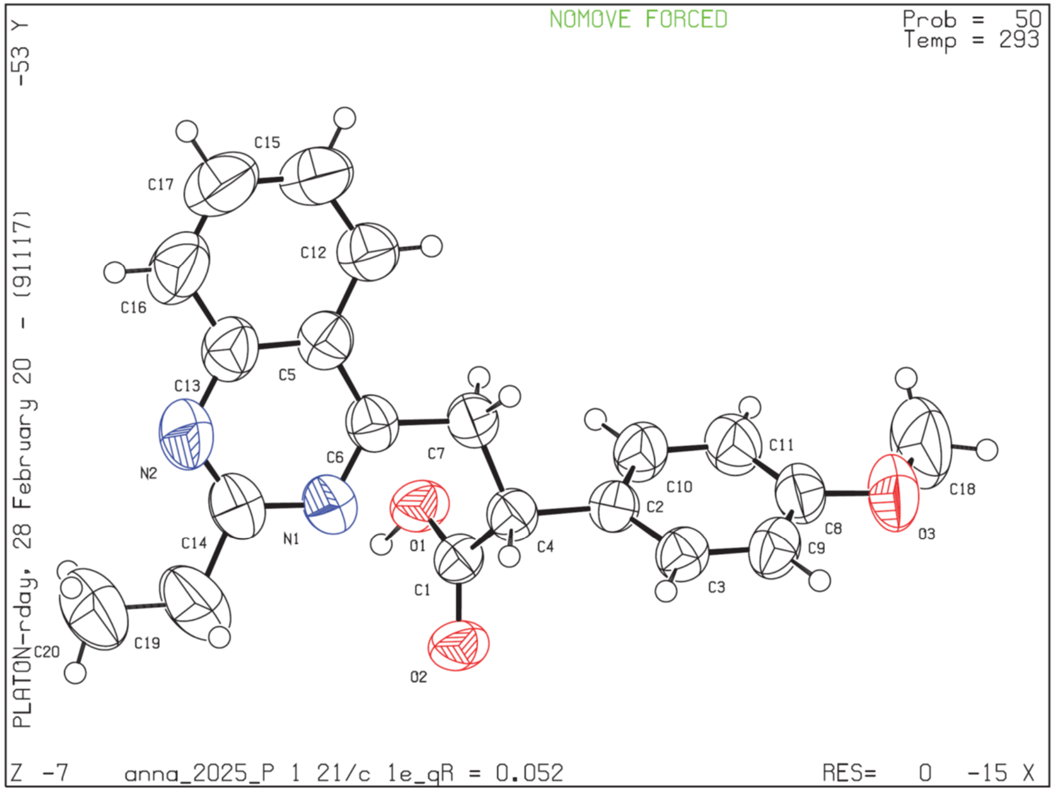

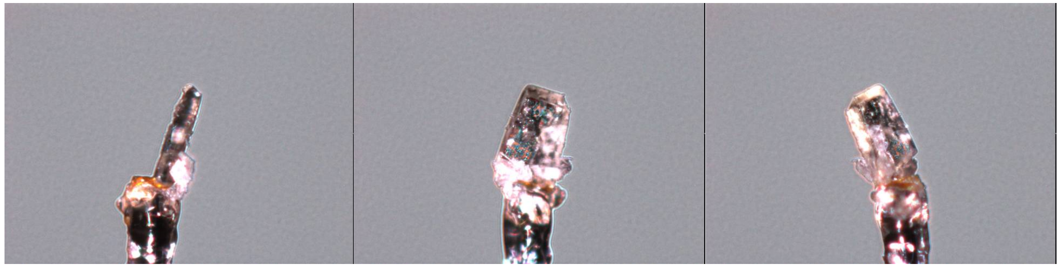

Fig S94. Anisotropic structure representation and crystal photos for **11ca**

Table 11 Crystal data and structure refinement for 11ca.

Identification code	ANNA_2025_QUINAZOLINE_Q21SUBST_2
Empirical formula	$C_{20}H_{19.52}N_2O_3$
Formula weight	335.89
Temperature/K	293(2)
Crystal system	monoclinic
Space group	$P2_1/c$
a/Å	13.5630(2)
b/Å	15.3850(3)
c/Å	8.93170(10)
α/°	90
β/°	100.075(2)
γ/°	90
Volume/Å^3	1835.01(5)
Z	4
ρ_{calc}g/cm^3	1.216
μ/mm^{-1}	0.669
F(000)	710.0
Crystal size/mm^3	0.4 × 0.24 × 0.09
Radiation	Cu Kα (λ = 1.54184)
2Θ range for data collection/°	6.62 to 152.388
Index ranges	$-17 \leq h \leq 17$, $-19 \leq k \leq 18$, $-11 \leq l \leq 11$
Reflections collected	19511
Independent reflections	3823 [R_{int} = 0.0202, R_{sigma} = 0.0128]
Data/restraints/parameters	3823/0/243
Goodness-of-fit on F^2	1.058
Final R indexes [I>=2σ (I)]	R_1 = 0.0465, wR_2 = 0.1326
Final R indexes [all data]	R_1 = 0.0527, wR_2 = 0.1409
Largest diff. peak/hole / e Å^{-3}	0.14/-0.21

Table 11 Fractional Atomic Coordinates (×11[4]) and Equivalent Isotropic Displacement Parameters (Å^2×18) for 11ca. U_{eq} is defined as 1/3 of of the trace of the orthogonalised U_{IJ} tensor.

Atom	*x*	*y*	*z*	U(eq)
O1	4970.5(8)	6120.2(6)	401.2(10)	68.5(3)
O2	4816.5(8)	4943.5(6)	1786.9(10)	72.8(3)
O3	8427.5(9)	6675.7(10)	6857.3(15)	98.9(4)
N1	2834.0(10)	6344.3(9)	1074.4(17)	79.5(4)
C1	4811.5(9)	5752.4(8)	1617.0(13)	57.0(3)
C2	5687.4(9)	6433.2(8)	3965.1(13)	57.2(3)
C3	5849.3(10)	6117.7(9)	5435.1(14)	61.8(3)
C4	4670.0(10)	6316.5(8)	2945.2(14)	58.8(3)
C5	2668.5(10)	7886.3(10)	851.7(17)	67.5(3)
C6	3187.4(10)	7125.2(9)	1435.0(15)	64.5(3)
C7	4191.8(10)	7191.8(9)	2456.2(16)	63.6(3)
N2	1416.3(11)	6934.6(12)	-554(2)	101.4(5)
C8	7541.5(11)	6626.9(10)	5850.5(17)	71.0(4)
C9	6764.0(12)	6212.1(10)	6370.8(15)	72.0(4)
C10	6469.9(11)	6846.0(11)	3458.9(17)	74.4(4)
C11	7395.4(12)	6949.0(11)	4389(2)	79.0(4)
C12	3003.9(12)	8740.8(10)	1220(2)	78.1(4)
C13	1781.6(11)	7754.6(12)	-188(2)	82.6(4)
C14	1941.9(14)	6283.8(14)	88(2)	96.6(6)
C15	2472.2(14)	9430.0(13)	555(3)	98.1(6)
C16	1259.1(15)	8488.7(17)	-875(3)	110.1(7)
C17	1606.9(17)	9301.8(15)	-502(3)	113.4(7)
C18	9258.7(17)	7040(2)	6320(4)	144.4(11)
C19	1580(2)	5356(2)	-215(5)	156.8(13)
C20	768(5)	5219(4)	-1495(8)	152(3)
C21	2152(7)	4762(4)	-634(9)	170(4)

Table 12 Anisotropic Displacement Parameters (Å^2×18) for 11ca. The Anisotropic displacement factor exponent takes the form: -2π^2[h^{2}a*^{2}U$_{11}$+2hka*b*U$_{12}$+…].

**Atom**	**U$_{11}$**	**U$_{22}$**	**U$_{33}$**	**U$_{23}$**	**U$_{8}$**	**U$_{12}$**
O1	92.6(7)	60.9(5)	53.8(5)	8.6(4)	17.3(4)	-1.9(4)
O2	108.1(8)	56.3(5)	54.6(5)	7.0(4)	15.8(5)	-1.3(5)
O3	72.6(7)	123.1(10)	90.2(8)	-16.3(7)	-15.8(6)	0.7(6)
N1	69.8(7)	75.0(8)	87.8(8)	-3.3(6)	-2.3(6)	-9.5(6)
C1	63.1(6)	57.8(7)	48.8(6)	6.4(5)	6.3(5)	-4.3(5)
C2	62.8(7)	57.6(6)	50.6(6)	0.2(5)	8.3(5)	-1.1(5)
C3	68.6(7)	66.4(7)	51.3(6)	0.4(5)	12.8(5)	2.4(6)
C4	62.7(7)	62.3(7)	51.6(6)	1.6(5)	10.2(5)	-6.1(5)
C5	55.9(7)	76.9(8)	70.8(8)	5.6(6)	14.2(6)	-1.5(6)
C6	58.4(7)	70.8(8)	64.2(7)	-2.4(6)	10.4(5)	-5.8(6)
C7	61.8(7)	63.5(7)	63.4(7)	-4.8(5)	4.8(5)	-1.6(5)
N2	71.4(8)	112.3(12)	108.4(12)	5.4(9)	-17.4(8)	-15.5(8)
C8	65.8(7)	73.4(8)	68.7(8)	-13.8(7)	-2.3(6)	4.1(6)
C9	78.4(8)	83.6(9)	51.4(6)	0.3(6)	3.7(6)	8.4(7)
C10	73.8(8)	84.9(9)	62.4(7)	15.0(7)	5.9(6)	-11.7(7)
C11	68.0(8)	85.8(10)	81.6(9)	5.0(8)	8.2(7)	-15.4(7)
C12	66.6(8)	76.1(9)	92.7(10)	5.6(8)	16.7(7)	-2.1(6)
C13	61.4(8)	96.1(11)	87.1(10)	12.0(8)	4.7(7)	-4.7(7)
C14	76.2(10)	95.4(12)	108.4(14)	-3.0(10)	-10.6(9)	-19.3(9)
C15	86.8(11)	78.7(10)	130.7(16)	22.4(11)	24.3(11)	6.4(9)
C16	77.3(10)	126.1(17)	118.0(16)	32.5(13)	-7.8(10)	7.6(11)
C17	95.4(13)	101.5(15)	140.3(19)	42.9(14)	12.9(12)	13.9(11)
C18	75.3(12)	186(3)	156(2)	1(2)	-22.3(13)	-30.3(14)
C19	126(2)	116(2)	205(4)	-21(2)	-34(2)	-42.0(17)
C20	136(5)	148(5)	156(5)	-10(4)	-20(4)	-55(4)
C21	252(10)	100(4)	145(5)	-13(4)	-1(6)	-50(5)

Table 8 Bond Lengths for 11ca.

Atom	Atom	Length/Å	Atom	Atom	Length/Å
O1	C1	1.2758(14)	C5	C13	1.400(2)
O2	C1	1.2536(16)	C6	C7	1.5045(18)
O3	C8	1.3712(17)	N2	C13	1.374(2)
O3	C18	1.416(3)	N2	C14	1.302(3)
N1	C6	1.3126(18)	C8	C9	1.381(2)
N1	C14	1.370(2)	C8	C11	1.377(2)
C1	C4	1.5089(17)	C10	C11	1.388(2)
C2	C3	1.3808(17)	C12	C15	1.359(2)
C2	C4	1.5252(17)	C13	C16	1.415(3)
C2	C10	1.3792(19)	C14	C19	1.518(3)
C3	C9	1.3770(19)	C15	C17	1.385(3)
C4	C7	1.5251(18)	C16	C17	1.358(3)
C5	C6	1.418(2)	C19	C20	1.457(6)
C5	C12	1.411(2)	C19	C21	1.296(9)

Table 14 Bond Angles for 11ca.

Atom	Atom	Atom	Angle/˚	Atom	Atom	Atom	Angle/˚
C8	O3	C18	117.24(17)	C14	N2	C13	117.02(14)
C6	N1	C14	117.62(14)	O3	C8	C9	115.97(14)
O1	C1	C4	118.53(11)	O3	C8	C11	124.47(15)
O2	C1	O1	123.04(12)	C11	C8	C9	119.56(13)
O2	C1	C4	118.33(10)	C3	C9	C8	120.30(13)
C3	C2	C4	120.22(11)	C2	C10	C11	121.56(13)
C10	C2	C3	118.04(12)	C8	C11	C10	119.44(14)
C10	C2	C4	121.74(11)	C15	C12	C5	120.00(17)

Table 14 Bond Angles for 11ca.

Atom	Atom	Atom	Angle/°	Atom	Atom	Atom	Angle/°
C9	C3	C2	121.10(13)	C5	C13	C16	118.64(17)
C1	C4	C2	108.12(10)	N2	C13	C5	121.54(15)
C1	C4	C7	112.89(10)	N2	C13	C16	119.82(16)
C7	C4	C2	111.23(10)	N1	C14	C19	113.59(19)
C12	C5	C6	124.38(13)	N2	C14	N1	125.79(17)
C13	C5	C6	115.98(14)	N2	C14	C19	120.62(18)
C13	C5	C12	119.62(14)	C12	C15	C17	120.54(19)
N1	C6	C5	121.96(13)	C17	C16	C13	120.17(19)
N1	C6	C7	117.66(13)	C16	C17	C15	121.00(18)
C5	C6	C7	120.35(12)	C20	C19	C14	116.9(3)
C6	C7	C4	114.09(11)	C21	C19	C14	121.5(4)

Table 15 Hydrogen Bonds for 11ca.

D	H	A	d(D-H)/Å	d(H-A)/Å	d(D-A)/Å	D-H-A/°
O1	H1	O2[1]	1.15(3)	1.45(4)	2.6037(13)	178(3)

[1]1-X,1-Y,-Z

Table 16 Torsion Angles for 11ca.

A	B	C	D	Angle/°	A	B	C	D	Angle/°
O1	C1	C4	C2	-92.58(13)	N2	C13	C16	C17	179.0(2)
O1	C1	C4	C7	30.91(16)	N2	C14	C19	C20	14.1(6)
O2	C1	C4	C2	83.95(14)	N2	C14	C19	C21	128.6(5)
O2	C1	C4	C7	-152.56(12)	C9	C8	C11	C10	-0.5(2)
O3	C8	C9	C3	-178.94(13)	C10	C2	C3	C9	0.0(2)
O3	C8	C11	C10	178.65(15)	C10	C2	C4	C1	62.96(16)

Table 16 Torsion Angles for 11ca.

A	B	C	D	Angle/°	A	B	C	D	Angle/°
N1	C6	C7	C4	-0.14(19)	C10	C2	C4	C7	-61.53(17)
N1	C14	C19	C20	-166.5(4)	C11	C8	C9	C3	0.2(2)
N1	C14	C19	C21	-51.9(6)	C12	C5	C6	N1	178.85(14)
C1	C4	C7	C6	57.79(15)	C12	C5	C6	C7	-3.4(2)
C2	C3	C9	C8	0.0(2)	C12	C5	C13	N2	-178.55(17)
C2	C4	C7	C6	179.54(11)	C12	C5	C13	C16	1.8(3)
C2	C10	C11	C8	0.5(3)	C12	C15	C17	C16	1.3(4)
C3	C2	C4	C1	-116.61(13)	C13	C5	C6	N1	-3.0(2)
C3	C2	C4	C7	118.90(13)	C13	C5	C6	C7	174.81(13)
C3	C2	C10	C11	-0.3(2)	C13	C5	C12	C15	-0.7(2)
C4	C2	C3	C9	179.63(12)	C13	N2	C14	N1	-1.2(4)
C4	C2	C10	C11	-179.84(14)	C13	N2	C14	C19	178.2(3)
C5	C6	C7	C4	-178.01(12)	C13	C16	C17	C15	-0.2(4)
C5	C12	C15	C17	-0.8(3)	C14	N1	C6	C5	0.8(2)
C5	C13	C16	C17	-1.3(3)	C14	N1	C6	C7	-176.99(15)
C6	N1	C14	N2	1.4(3)	C14	N2	C13	C5	-1.2(3)
C6	N1	C14	C19	-178.0(2)	C14	N2	C13	C16	178.4(2)
C6	C5	C12	C15	177.41(16)	C18	O3	C8	C9	175.4(2)
C6	C5	C13	N2	3.2(2)	C18	O3	C8	C11	-3.7(3)
C6	C5	C13	C16	-176.50(17)					

Table 17 Hydrogen Atom Coordinates (Å×11[4]) and Isotropic Displacement Parameters (Å[2]×18) for 11ca.

Atom	*x*	*y*	*z*	U(eq)
H3	5331.84	5836.79	5799.24	74
H4	4229.92	6008.02	3527.18	71
H7A	4114.93	7518	3357.98	76
H7B	4641.39	7516.37	1931.29	76

Table 17 Hydrogen Atom Coordinates (Å×11[4]) and Isotropic Displacement Parameters (Å[2]×18) for 11ca.

Atom	*x*	*y*	*z*	U(eq)
H9	6858.64	5995.52	7357.68	86
H10	6375.28	7060.48	2470.56	89
H11	7912.38	7233.13	4029.69	95
H12	3588.64	8832.4	1918.84	94
H15	2691.06	9992.21	811.2	118
H16	675.85	8414.02	-1584.33	132
H17	1259.14	9779.88	-962.33	136
H18A	9386.73	6714.84	5456.04	217
H18B	9837.2	7017.61	7110.32	217
H18C	9115.9	7633.16	6027.93	217
H19A	2145.67	5005.06	-378.28	188
H19B	1364.07	5138.85	695.13	188
H19	932.38	5210.46	-96.48	188
H20A	167.3	5484.19	-1281.3	228
H20B	659.58	4606.52	-1653.92	228
H20C	942.57	5475.05	-2393.27	228
H21A	2377.59	4945.6	-1544.54	255
H21B	1783.16	4229.5	-825.37	255
H21C	2719.86	4671.74	157.67	255
H1	5080(20)	5650(20)	-550(40)	183(12)

Table 18 Atomic Occupancy for 11ca.

Atom	*Occupancy*	Atom	*Occupancy*	Atom	*Occupancy*
H19A	0.520(6)	H19B	0.520(6)	H19	0.480(6)
C20	0.520(6)	H20A	0.520(6)	H20B	0.520(6)
H20C	0.520(6)	C21	0.480(6)	H21A	0.480(6)
H21B	0.480(6)	H21C	0.480(6)		

Experimental

Single crystals of $C_{20}H_{19.52}N_2O_3$ **11ca** were **Crystallized by slow evaporation of saturated solution in EtOAc**. A suitable crystal was selected and **Mounted on the glass stick by acrylic glue** on a **SuperNova, Dual, Cu at home/near, AtlasS2** diffractometer. The crystal was kept at 293(2) K during data collection. Using Olex2 [39], the structure was solved with the SHELXT [40] structure solution program using Intrinsic Phasing and refined with the SHELXL [41] refinement package using Least Squares minimisation.

Crystal structure determination of 11ca

Crystal Data for $C_{20}H_{19.52}N_2O_3$ (M =335.89 g/mol): monoclinic, space group P2$_1$/c (no. 14), a = 13.5630(2) Å, b = 15.3850(3) Å, c = 8.93170(10) Å, β = 100.075(2)°, V = 1835.01(5) Å^3, Z = 4, T = 293(2) K, μ(Cu Kα) = 0.669 mm^{-1}, $Dcalc$ = 1.216 g/cm^3, 19511 reflections measured (6.62° ≤ 2Θ ≤ 152.388°), 3823 unique (R_{int} = 0.0202, R_{sigma} = 0.0128) which were used in all calculations. The final R_1 was 0.0465 (I > 2σ(I)) and wR_2 was 0.1409 (all data).

Refinement model description

Number of restraints - 0, number of constraints - unknown.

Details:

1. Fixed Uiso

At 1.2 times of:

All C(H) groups, {H19A,H19B,H19} of C19, All C(H,H) groups

At 1.5 times of:

{H21A,H21B,H21C} of C21, {H20A,H20B,H20C} of C20, {H18A,H18B,H18C} of C18

2. Others

Sof(H19)=Sof(C21)=Sof(H21A)=Sof(H21B)=Sof(H21C)=1-FVAR(1)

Sof(H19A)=Sof(H19B)=Sof(C20)=Sof(H20A)=Sof(H20B)=Sof(H20C)=FVAR(1)

3.a Ternary CH refined with riding coordinates:

C4(H4)

3.b Secondary CH2 refined with riding coordinates:

C7(H7A,H7B), C19(H19A,H19B)

3.c Aromatic/amide H refined with riding coordinates:

C3(H3), C9(H9), C10(H10), C11(H11), C12(H12), C15(H15), C16(H16), C17(H17), C19(H19)

3.d Idealised Me refined as rotating group:

C18(H18A,H18B,H18C), C20(H20A,H20B,H20C), C21(H21A,H21B,H21C)

Xray diffraction data for 23al

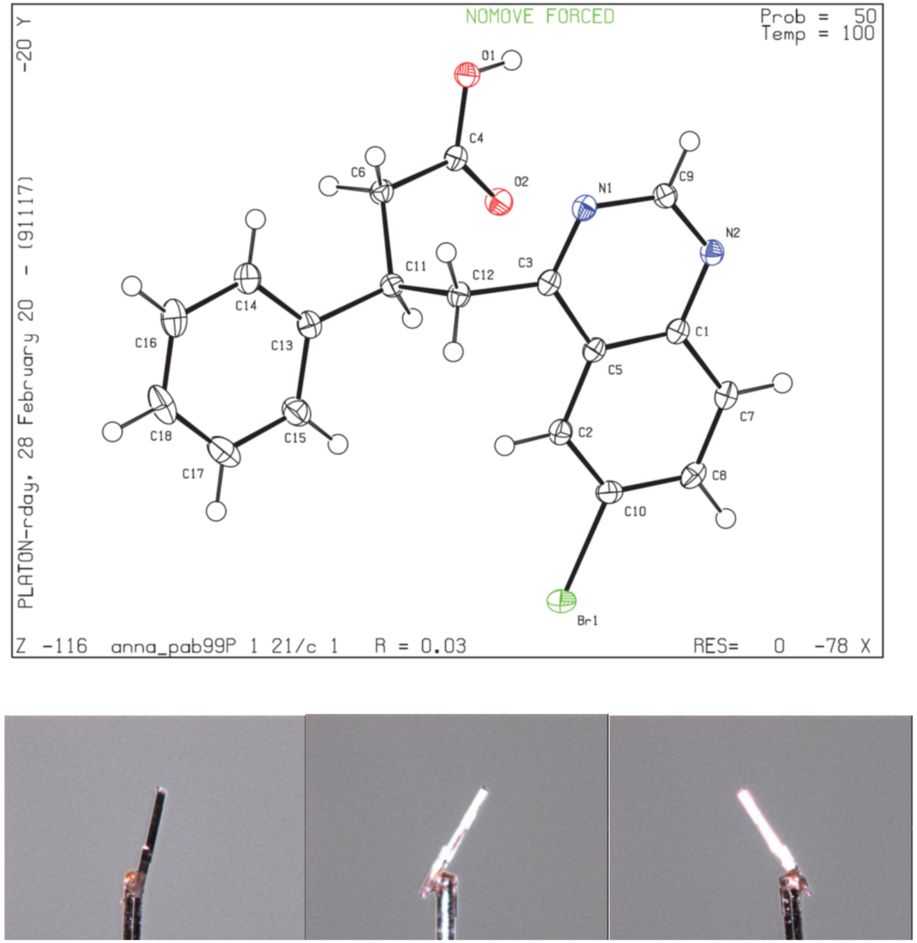

Fig S95. Anisotropic structure representation and crystal photos for **23al**

Table 19 Crystal data and structure refinement for 23al.

Identification code	ANNA_PAB99_1
Empirical formula	$C_{18}H_{15}BrN_2O_2$
Formula weight	371.23

Temperature/K	100.00(10)
Crystal system	monoclinic
Space group	$P2_1/c$
a/Å	9.12480(10)
b/Å	5.30060(10)
c/Å	32.5036(4)
α/°	90
β/°	97.0770(10)
γ/°	90
Volume/Å^3	1560.12(4)
Z	4
ρ_{calc}g/cm^3	1.580
μ/mm^{-1}	3.687
F(000)	752.0
Crystal size/mm^3	0.56 × 0.07 × 0.04
Radiation	Cu Kα (λ = 1.54184)
2Θ range for data collection/°	5.48 to 152.352
Index ranges	$-11 \le h \le 11$, $-5 \le k \le 6$, $-40 \le l \le 40$
Reflections collected	16994
Independent reflections	3269 [R_{int} = 0.0426, R_{sigma} = 0.0319]
Data/restraints/parameters	3269/0/212
Goodness-of-fit on F^2	1.050
Final R indexes [I>=2σ (I)]	R_1 = 0.0259, wR_2 = 0.0643
Final R indexes [all data]	R_1 = 0.0280, wR_2 = 0.0659
Largest diff. peak/hole / e Å^{-3}	0.34/-0.61

Table 20 Fractional Atomic Coordinates (×11^4) and Equivalent Isotropic Displacement Parameters (Å^2×18) for 23al. U_{eq} is defined as 1/3 of of the trace of the orthogonalised U_{IJ} tensor.

**Atom**	***x***	***y***	***z***	**U(eq)**
Br1	-1677.1(2)	-1755.7(3)	3479.8(2)	24.55(8)

Table 20 Fractional Atomic Coordinates (×11[4]) and Equivalent Isotropic Displacement Parameters (Å^2×18) for 23al. U_{eq} is defined as 1/3 of of the trace of the orthogonalised U_{IJ} tensor.

Atom	*x*	*y*	*z*	U(eq)
O1	6996.3(12)	2639(2)	4351.1(4)	18.0(2)
O2	5168.4(12)	-24(2)	4123.4(3)	18.3(2)
N1	3623.2(14)	3967(2)	4639.1(4)	15.5(2)
N2	2659.9(14)	488(2)	4990.5(4)	15.2(2)
C1	1671.7(15)	15(3)	4647.5(5)	14.1(3)
C2	617.6(16)	977(3)	3938.2(5)	15.8(3)
C3	2709.6(16)	3519(3)	4302.3(5)	13.9(3)
C4	5812.6(17)	1964(3)	4095.3(5)	14.1(3)
C5	1649.9(16)	1508(3)	4286.7(5)	13.6(3)
C6	5380.5(16)	3898(3)	3759.8(5)	15.5(3)
C7	645.3(17)	-1991(3)	4655.3(5)	16.3(3)
C8	-358.2(17)	-2487(3)	4315.7(5)	17.0(3)
C9	3563.1(17)	2408(3)	4971.3(5)	15.5(3)
C10	-346.2(16)	-986(3)	3957.3(5)	16.5(3)
C11	3720.5(16)	3924(3)	3604.6(4)	13.9(3)
C12	2821.6(16)	5152(3)	3929.6(5)	14.6(3)
C13	3361.4(17)	5338(3)	3199.4(5)	16.1(3)
C14	4159.5(18)	7445(3)	3097.6(5)	19.6(3)
C15	2134.6(19)	4592(3)	2925.9(5)	22.4(3)
C16	3751(2)	8749(3)	2728.1(6)	26.2(4)
C17	1716(2)	5911(4)	2560.6(6)	28.5(4)
C18	2527(2)	7986(4)	2461.0(6)	29.2(4)

Table 21 Anisotropic Displacement Parameters (Å^2×18) for 23al. The Anisotropic displacement factor exponent takes the form: -2π^2[$h^2a^{*2}U_{11}$+2hka*b*U_{12}+…].

Atom	U_{11}	U_{22}	U_{33}	U_{23}	U_8	U_{12}

Table 21 Anisotropic Displacement Parameters (Å^2×18) for 23al. The Anisotropic displacement factor exponent takes the form: -2π^2[h^{2}a*^{2}U$_{11}$+2hka*b*U$_{12}$+…].

Atom	U_{11}	U_{22}	U_{33}	U_{23}	U_8	U_{12}
Br1	20.23(10)	33.32(11)	19.15(10)	-1.94(6)	-1.31(7)	-10.83(6)
O1	17.8(5)	17.9(5)	17.7(5)	4.4(4)	-0.7(4)	-3.3(4)
O2	21.4(5)	13.5(5)	19.4(5)	0.8(4)	0.4(4)	-2.3(4)
N1	16.6(6)	13.9(5)	16.5(6)	-1.0(5)	3.8(5)	-1.0(4)
N2	15.6(6)	15.5(6)	14.9(6)	0.4(5)	3.3(5)	0.9(4)
C1	14.2(6)	13.1(6)	15.8(7)	-1.0(5)	4.9(5)	2.0(5)
C2	15.7(7)	16.7(7)	15.3(7)	0.5(5)	3.7(5)	0.1(5)
C3	13.9(6)	12.1(6)	16.6(7)	-0.9(5)	5.3(5)	2.0(5)
C4	15.1(7)	12.7(6)	15.1(7)	-0.7(5)	4.2(5)	0.7(5)
C5	12.8(6)	12.8(6)	15.8(7)	-0.2(5)	4.0(5)	1.2(5)
C6	14.6(7)	15.4(6)	16.7(7)	3.4(5)	2.2(5)	0.0(5)
C7	17.9(7)	14.5(6)	17.4(7)	1.5(5)	5.9(6)	0.1(5)
C8	16.0(7)	14.8(6)	21.1(7)	-2.5(6)	6.4(6)	-3.3(5)
C9	15.7(7)	16.6(6)	14.2(7)	-1.2(5)	2.1(5)	-0.5(5)
C10	13.3(6)	19.3(7)	16.6(7)	-3.6(6)	1.4(5)	-0.7(5)
C11	15.5(7)	11.8(6)	14.1(6)	-0.3(5)	1.5(5)	-1.8(5)
C12	16.6(6)	11.8(6)	15.8(7)	0.6(5)	3.5(5)	-1.0(5)
C13	18.9(7)	15.2(6)	14.7(7)	-0.6(5)	3.7(5)	3.1(5)
C14	23.2(8)	16.4(7)	19.7(7)	1.5(6)	4.2(6)	1.6(6)
C15	25.1(8)	23.8(7)	17.7(7)	-0.5(6)	-0.1(6)	-1.5(6)
C16	36.8(10)	20.8(7)	22.4(8)	5.7(6)	8.9(7)	2.3(7)
C17	29.5(9)	35.9(9)	18.2(8)	0.0(7)	-4.9(7)	3.4(7)
C18	40.4(10)	31.5(9)	15.3(7)	6.0(6)	1.7(7)	10.2(7)

Table 22 Bond Lengths for 23al.

Atom	Atom	Length/Å	Atom	Atom	Length/Å

Table 22 Bond Lengths for 23al.

Atom	Atom	Length/Å	Atom	Atom	Length/Å
Br1	C10	1.8935(16)	C4	C6	1.513(2)
O1	C4	1.328(2)	C6	C11	1.536(2)
O2	C4	1.2158(19)	C7	C8	1.370(2)
N1	C3	1.313(2)	C8	C10	1.412(2)
N1	C9	1.366(2)	C11	C12	1.5573(19)
N2	C1	1.368(2)	C11	C13	1.516(2)
N2	C9	1.316(2)	C13	C14	1.395(2)
C1	C5	1.413(2)	C13	C15	1.398(2)
C1	C7	1.419(2)	C14	C16	1.396(2)
C2	C5	1.410(2)	C15	C17	1.390(2)
C2	C10	1.369(2)	C16	C18	1.387(3)
C3	C5	1.436(2)	C17	C18	1.386(3)
C3	C12	1.5030(19)			

Table 23 Bond Angles for 23al.

Atom	Atom	Atom	Angle/°	Atom	Atom	Atom	Angle/°
C3	N1	C9	117.80(13)	C7	C8	C10	118.96(14)
C9	N2	C1	117.19(13)	N2	C9	N1	126.27(15)
N2	C1	C5	120.95(13)	C2	C10	Br1	118.52(12)
N2	C1	C7	119.54(14)	C2	C10	C8	122.42(15)
C5	C1	C7	119.51(14)	C8	C10	Br1	119.05(11)
C10	C2	C5	119.02(14)	C6	C11	C12	111.19(12)
N1	C3	C5	121.29(14)	C13	C11	C6	112.86(12)
N1	C3	C12	117.94(13)	C13	C11	C12	107.89(11)
C5	C3	C12	120.76(14)	C3	C12	C11	113.56(12)
O1	C4	C6	112.85(12)	C14	C13	C11	122.58(14)
O2	C4	O1	123.35(14)	C14	C13	C15	118.44(15)

Table 23 Bond Angles for 23al.

Atom	Atom	Atom	Angle/°	Atom	Atom	Atom	Angle/°
O2	C4	C6	123.78(14)	C15	C13	C11	118.89(13)
C1	C5	C3	116.48(14)	C13	C14	C16	120.55(16)
C2	C5	C1	119.65(13)	C17	C15	C13	120.99(16)
C2	C5	C3	123.87(14)	C18	C16	C14	120.23(16)
C4	C6	C11	113.67(12)	C18	C17	C15	120.02(18)
C8	C7	C1	120.44(14)	C17	C18	C16	119.77(16)

Table 24 Hydrogen Bonds for 23al.

D	H	A	d(D-H)/Å	d(H-A)/Å	d(D-A)/Å	D-H-A/°
O1	H1	N2[1]	0.85(3)	1.85(3)	2.6943(17)	170(3)

[1]1-X,-Y,1-Z

Table 25 Torsion Angles for 23al.

A	B	C	D	Angle/°	A	B	C	D	Angle/°
O1	C4	C6	C11	-151.23(12)	C7	C8	C10	Br1	177.42(11)
O2	C4	C6	C11	30.3(2)	C7	C8	C10	C2	-1.0(2)
N1	C3	C5	C1	-1.5(2)	C9	N1	C3	C5	1.9(2)
N1	C3	C5	C2	178.34(13)	C9	N1	C3	C12	-177.14(12)
N1	C3	C12	C11	98.54(15)	C9	N2	C1	C5	1.2(2)
N2	C1	C5	C2	-179.94(13)	C9	N2	C1	C7	-178.31(13)
N2	C1	C5	C3	-0.06(19)	C10	C2	C5	C1	0.0(2)
N2	C1	C7	C8	179.68(13)	C10	C2	C5	C3	-179.88(13)
C1	N2	C9	N1	-0.8(2)	C11	C13	C14	C16	-177.38(14)
C1	C7	C8	C10	0.5(2)	C11	C13	C15	C17	176.64(15)
C3	N1	C9	N2	-0.7(2)	C12	C3	C5	C1	177.49(12)

Table 25 Torsion Angles for 23al.

A	B	C	D	Angle/°	A	B	C	D	Angle/°
C4	C6	C11	C12	73.78(15)	C12	C3	C5	C2	-2.6(2)
C4	C6	C11	C13	-164.81(12)	C12	C11	C13	C14	89.62(16)
C5	C1	C7	C8	0.2(2)	C12	C11	C13	C15	-86.80(16)
C5	C2	C10	Br1	-177.69(10)	C13	C11	C12	C3	162.89(12)
C5	C2	C10	C8	0.8(2)	C13	C14	C16	C18	1.2(2)
C5	C3	C12	C11	-80.50(16)	C13	C15	C17	C18	0.6(3)
C6	C11	C12	C3	-72.85(15)	C14	C13	C15	C17	0.1(2)
C6	C11	C13	C14	-33.64(19)	C14	C16	C18	C17	-0.5(3)
C6	C11	C13	C15	149.94(14)	C15	C13	C14	C16	-0.9(2)
C7	C1	C5	C2	-0.5(2)	C15	C17	C18	C16	-0.4(3)
C7	C1	C5	C3	179.41(13)					

Table 26 Hydrogen Atom Coordinates (Å×11[4]) and Isotropic Displacement Parameters (Å[2]×18) for 23al.

Atom	*x*	*y*	*z*	U(eq)
H2	591.8	1945.54	3698.84	19
H6A	5922.64	3557.27	3527.22	19
H6B	5669.82	5558.54	3866	19
H7	653.9	-2974.76	4892.45	20
H8	-1036.59	-3792.49	4321.19	20
H9	4224.13	2735.5	5206.52	19
H11	3388.6	2175.44	3561.44	17
H12A	3284.31	6737.19	4020.66	18
H12B	1833.81	5526.19	3797.32	18
H14	4970.44	7986.19	3277.49	24
H15	1590.12	3192.59	2989.35	27
H16	4300.79	10132.63	2660.81	31
H17	892.03	5401.39	2383.06	34

Table 26 Hydrogen Atom Coordinates (Å×11[4]) and Isotropic Displacement Parameters (Å[2]×18) for 23al.

Atom	*x*	*y*	*z*	U(eq)
H18	2252.5	8864.45	2215.9	35
H1	7150(30)	1530(60)	4541(10)	47(8)

Experimental

Single crystals of $C_{18}H_{15}BrN_2O_2$ **23a** were **Crystallized by slow evaporation of saturated solution in EtOAc**. A suitable crystal was selected and **Mounted on the glass stick by acrylic glue** on a **SuperNova, Dual, Cu at home/near, AtlasS2** diffractometer. The crystal was kept at 100.00(10) K during data collection. Using Olex2 [39], the structure was solved with the SHELXT [40] structure solution program using Intrinsic Phasing and refined with the SHELXL [41] refinement package using Least Squares minimisation.

Crystal structure determination of 23al

Crystal Data for $C_{18}H_{15}BrN_2O_2$ (M =371.23 g/mol): monoclinic, space group $P2_1/c$ (no. 14), a = 9.12480(10) Å, b = 5.30060(10) Å, c = 32.5036(4) Å, β = 97.0770(10)°, V = 1560.12(4) Å^3, Z = 4, T = 100.00(10) K, μ(Cu Kα) = 3.687 mm^{-1}, $Dcalc$ = 1.580 g/cm^3, 16994 reflections measured (5.48° ≤ 2Θ ≤ 152.352°), 3269 unique (R_{int} = 0.0426, R_{sigma} = 0.0319) which were used in all calculations. The final R_1 was 0.0259 (I > 2σ(I)) and wR_2 was 0.0659 (all data).

Refinement model description

Number of restraints - 0, number of constraints - unknown.
Details:
1. Fixed Uiso
At 1.2 times of:
All C(H) groups, All C(H,H) groups
2.a Ternary CH refined with riding coordinates:
C11(H11)
2.b Secondary CH2 refined with riding coordinates:
C6(H6A,H6B), C12(H12A,H12B)
2.c Aromatic/amide H refined with riding coordinates:
C2(H2), C7(H7), C8(H8), C9(H9), C14(H14), C15(H15), C16(H16), C17(H17),
C18(H18)
